# Canine Mammary Tumor Histopathological Image Classification via Computer-Aided Pathology: An Available Dataset for Imaging Analysis

**DOI:** 10.3390/ani13091563

**Published:** 2023-05-06

**Authors:** Giovanni P. Burrai, Andrea Gabrieli, Marta Polinas, Claudio Murgia, Maria Paola Becchere, Pierfranco Demontis, Elisabetta Antuofermo

**Affiliations:** 1 Department of Veterinary Medicine, University of Sassari, Via Vienna 2, 07100 Sassari, Italy; gburrai@uniss.it (G.P.B.); claudio97murgia@gmail.com (C.M.);; 2 Mediterranean Center for Disease Control (MCDC), University of Sassari, Via Vienna 2, 07100 Sassari, Italy; 3 Independent Researcher, 07100 Sassari, Italy; 4 Department of Chemical, Physical, Mathematical and Natural Sciences, University of Sassari, Via Vienna 2, 07100 Sassari, Italy; demontis@uniss.it

**Keywords:** breast cancer, canine mammary tumor (CMTs), CMT dataset, deep learning, machine learning, histological classification

## Abstract

**Simple Summary:**

Digital pathology (DP) and computer-aided diagnosis (CAD) are rapidly evolving fields that have great potential for improving the accuracy and efficiency of cancer diagnosis, including that of canine mammary tumors (CMTs), the most common neoplasm in female dogs. The work presents a study on the development of CAD systems for the automated classification of CMTs utilizing convolutional neural networks (CNNs) to extract features from histopathological images of CMTs and classify them into benign or malignant tumors. The study shows that the proposed framework can accurately distinguish between benign and malignant CMTs, with testing accuracies ranging from 0.63 to 0.85. The study emphasizes how digital pathology and CAD could help veterinarians and pathologists in accurately diagnosing the tumor type, which is crucial in determining the optimal course of treatment. Overall, digital pathology and CAD are promising tools that could improve the accuracy and efficiency of cancer diagnosis, including that of canine mammary tumors.

**Abstract:**

Histopathology, the gold-standard technique in classifying canine mammary tumors (CMTs), is a time-consuming process, affected by high inter-observer variability. Digital (DP) and Computer-aided pathology (CAD) are emergent fields that will improve overall classification accuracy. In this study, the ability of the CAD systems to distinguish benign from malignant CMTs has been explored on a dataset—namely *CMTD*—of 1056 hematoxylin and eosin JPEG images from 20 benign and 24 malignant CMTs, with three different CAD systems based on the combination of a convolutional neural network (VGG16, Inception v3, EfficientNet), which acts as a feature extractor, and a classifier (support vector machines (SVM) or stochastic gradient boosting (SGB)), placed on top of the neural net. Based on a human breast cancer dataset (i.e., BreakHis) (accuracy from 0.86 to 0.91), our models were applied to the CMT dataset, showing accuracy from 0.63 to 0.85 across all architectures. The EfficientNet framework coupled with SVM resulted in the best performances with an accuracy from 0.82 to 0.85. The encouraging results obtained by the use of DP and CAD systems in CMTs provide an interesting perspective on the integration of artificial intelligence and machine learning technologies in cancer-related research.

## 1. Introduction

Cancer is the leading cause of death in companion animals, and canine mammary tumor (CMTs), the most common neoplasm in female dogs, represents a serious issue in worldwide veterinary practice [1,2,3,4]. Therefore, an increased number of studies in this area have been published in the last decades. As in animals, human breast cancer (HBC) is the most common malignancy among women worldwide, sharing several clinical and molecular similarities with canine lesions [5,6,7]. Consequentially, dogs have attracted considerable attention as potential animal models to study human cancer [8].

Detection and diagnosis of mammary tumors, alongside a clinical examination, can be accomplished via imaging procedures such as diagnostic mammograms, ultrasound, and magnetic resonance imaging [1,9], although histopathological analysis remains the gold standard for differentiating between benign and malignant neoplasms [1,3].

Histopathological analysis is a time-consuming process, requiring highly trained specialists, and could be influenced by several intrinsic and extrinsic factors, including adequate specimen fixation, laboratory handling, and the pathologists’ experience [10]. In histopathology, a high percentage of cancer can be diagnosed by pathologists using hematoxylin and eosin (H&E)-stained slides. Furthermore, diagnosis based upon manual analysis of slides is prone to inter-observer variability, with approximately 75% diagnostic concordance between specialists [3,11,12,13]. Digital pathology (DP) is a significant modernization that changes the paradigm of microscope-based pathology, replacing the microscope with the computer screen and changing storage media from glass slides to digitalized image files [14]. Digitalized images stored in computer servers or cloud systems can be easily transmitted, thus changing the temporal and spatial domain of pathologic diagnosis [15]. Moreover, digitalized images can be further analyzed by the so-called computer-aided pathology (CAP), referred to as a computational diagnosis system or a set of methodologies that utilize computers or software to interpret pathologic images [14,15,16,17]. CAD systems using machine learning algorithms have been demonstrated to improve classification accuracy and reduce variability in interpretations, increasing the level of inter-observer agreement. Several validation studies have compared the diagnostic accuracy of DP and conventional microscopic diagnosis in the last decade [18,19]. In addition, these techniques are also useful for assisting pathologists and reducing their effort in localizing and identifying abnormalities in cancer tissue images.

In recent years, the increase in computing power due to the spread of parallel architectures based on graphical processing units (GPU) has boosted the emergence of deep learning algorithms. In particular, convolutional neural networks (CNNs) have become the elected method in the field of image analysis [20,21,22,23,24,25] and a powerful tool in the automated classification of human cancer histopathology images [26,27,28,29,30,31,32,33]. CNNs are particularly well-suited and efficient at processing data that manifest local spatial correlation with grid-like topologies [21,22]. The fundamental element is the so-called convolutional layer. In its simplest form, such a layer consists of several learnable weight matrices (kernels) of small spatial dimensions (i.e., a typical kernel for the modern architecture has a size of 3 × 3: 3 pixels wide and 3 pixels high). Kernels are convoluted with the input data, thus generating two-dimensional activation maps. This allows the discovery of particular aspects of the input data, and the weights are efficiently reused wherever a particular feature is located in an image. By modifying the weights, the network can learn patterns and pattern hierarchies and then learn to distinguish images.

CNNs automatically learn mid- and high-level abstractions obtained from RGB images [34], and, along with multiple-instance learning, have accomplished high performance in the binary classification of human cancers and have evolved as one method for analyzing histopathological images [35].

Despite canine mammary tumors representing a serious issue in worldwide veterinary practice, no consistent efforts have been made to automate the diagnosis of CMTs. In this study, a canine mammary tumor image database comprising images captured from 44 cases of CMTs explored with three different CNN architectures, namely VGG16, Inception v3, and EfficientNet, associated with support vector machines (SVM) and stochastic gradient boosting (SGB) was used to investigate the ability to distinguish benign and malignant tumors on H&E-stained images, based on histopathological analysis as a gold standard. Furthermore, the models were tested on a standard human breast cancer dataset (BreakHis) and the effects of data augmentation on the performance of the proposed framework were also analyzed. Thus, a complete novel dataset, namely *CMTD*, of the most common benign and malignant mammary canine tumors was provided.

## 2. Materials and Methods

### 2.1. Canine Mammary Tumor Dataset

The canine mammary tumor image dataset (*CMTD*) is comprises 1056 H&E-stained JPEG images of a size of 1024 × 768 (width × height), acquired from 44 canine mammary tumors that were submitted to the Departments of Veterinary Medicine of the University of Sassari (UNISS). Tissue samples were fixed in 10% neutral buffered formalin, paraffin-embedded and H&E-stained for histopathological analysis. The histopathological classification of CMT tissues was performed in accordance with the recent publication of *Surgical Pathology of Tumors of Domestic Animals, Volume 2: Mammary Tumors* [36] by one board-certified and two experienced veterinary pathologists. A recorded video of a mean time of 2 min was performed for each sample. From each video, a pool of frames was programmatically chosen, and from this pool, the pathologists selected the best 24 images at a 400× magnification. Thus, 1024 × 768 high-resolution RGB images with a 24 bit color depth were captured, comprising a total of 1056 images from 20 benign and 24 malignant CMT cases (Supplementary Material: Canine Mammary Tumor Dataset—*CMTD*). Images and videos were obtained from an optical microscope (Optica c-B3) equipped with a digital camera (C-B3 optikamB3 digital camera).

Experimental permission was not required from the University’s Animal Care Ethics Committee since all the samples were retrieved from the archive of the pathology laboratories and were used for diagnostic purposes.

### 2.2. Breast Cancer Dataset

The proposed framework was first evaluated on the standard and challenging BreakHis dataset, which is freely available (https://web.inf.ufpr.br/vri/databases/breast-cancer-histopathological-database-breakhis/ (accessed on 5 April 2021 comprising 9109 images with different magnifying factors from human breast cancer patients. The dataset included 2480 benign (fibroadenoma, tubular adenoma, and phyllodes tumor) and 5429 malignant images of breast tumors (lobular carcinoma, ductal carcinoma, papillary carcinoma, and mucinous carcinoma) [37,38].

### 2.3. Data Processing

To estimate the generalization error in both CMTD and BreakHis datasets, a nested 5-fold cross-validation procedure was employed [39]. The data were partitioned into five different non-overlapping training and test sets (outer-cv), so that all images belonging to the same histological slide (i.e., patient) fell in either the test or the training set but not both, to avoid information leaking [37,39,40]. For each of the aforementioned training sets, further 5-fold cross-validation (inner-cv) was performed for model tuning and selection. The tuning was performed with a random grid search. The final result was obtained by averaging the test sets of the outer loop. For details, the reader can refer to the scikit-learn documentation and the python code accompanying this work available at https://github.com/cpctools/CMTD_classification (accessed on 1 March 2023) [41].

### 2.4. Data Augmentation

The images were subjected to data augmentation to increase the data size and make our model robust for feature transformation. Since we used CNN as a feature extractor, the features were generated and stored before the training of the classifier. Two different strategies of data augmentation were employed. The first one (i.e., the *base* strategy) consisted of resizing the image to 512 pixels (px), then randomly cropping it to 224 px for VGG (see below), 299 px for Inception (see below), and 380 px for EfficientNet (see below) (default crop size accepted by each model) [24], random rotation by 0, 90, 180, and 270 degrees, and random vertical reflection, similarly to what was described by Araujo et al., 2017, and Kumar et al., 2020 [30,42]. The second strategy employed (i.e., the *advanced* strategy) consisted of taking a crop of a random size between 0.08 and 1.0 of the original size and a random aspect ratio of 3/4 or 4/3 and then resizing it to 224 px for VGG, 299 px for Inception, and 380 px for EfficientNet [43]. The same random rotation and vertical reflection methods described for the first strategy were used. Finally, a random change of ± 20% of the brightness, contrast, and saturation was applied. In both cases, the images were normalized using ImageNet statistics [44]. Features extracted with the CNN were then standard-scaled (with statistics from each training set in both cross-validation loops) before the classification. The predictions were performed by using the center crop of the image (i.e., cropping the center portion of an image and using it as a new image for evaluation) or the ten crops [24], where the central crop and the four corners, as well as the horizontal flip of these five, were separately evaluated, and then the decision was made via majority voting.

### 2.5. Convolutional Neural Networks (CNN)

Three different CNN architectures for feature extraction, namely VGG16 [45], Inception v3 [46], and EfficientNet [47] were employed and compared to each other. The VGG16 architecture marked a fundamental step in the evolution of CNNs. By increasing the depth of the network and reducing the size of the filters, its proposers have obtained excellent results in the ImageNet Large Scale Visual Recognition Challenge (ILSVRC, the test bench on which the new architectures proposed are compared) [48]. This architecture has simplicity among its strengths but has a high computational cost. More specifically, for VGG16 architecture features are extracted after each convolutional block just before the max-pooling layer, as reported in the Supplementary Materials (Figure S1).

Inception v3 is an architecture with great success and large performance improvement compared to the state of the art at a relatively low computational cost. It is an evolution of the so-called GoogLeNet [43]. The key to its success is the inception module which replaces a simple convolution with a composition of kernels of different sizes and pooling operations leading to a reduction in the total number of parameters and an increase in performance. For Inception v3, the extraction happens after the first two convolutional blocks and the first two inception blocks as shown in the Supplementary Materials (Figure S2).

The EfficientNet is a very recent architecture showing state-of-the-art performance. The building block of this architecture is a mobile inverted-bottleneck MBConv with added squeeze-and-excitation optimization [49]. It is built starting from a baseline network called EfficientNet-B0 (obtained using a neural architecture search [50]), and then scaled up to find an optimal combination of depth (i.e., number of layers), width (i.e., number of channels), and resolution of the input image. Features are extracted from several intermediate convolutional maps, a global average pooling (GAP) is applied along spatial dimensions, and the obtained feature vectors are concatenated [42,51,52]. This approach allows an exploitation of the hierarchical nature of CNN where each filter becomes sensitive to a different pattern via shifting the attention from global to local structures [51,53,54]. For EfficientNet-B4, the extraction occurs before every channel increase as depicted in the Supplementary Materials (Figure S3).

The extracted features are used as input for machine learning algorithms, such as support vector machines (SVMs) and stochastic gradient boosting (SGB), to perform classification.

### 2.6. Support Vector Machines

SVMs are a class of algorithms that were developed in the 1990s for classification tasks and have seen successful application in many areas, including breast cancer predictions [22,26,28,30,37,54,55,56,57]. The idea behind these kinds of algorithms is to find an optimal separating hyperplane between classes. In the simplest case, when classes are perfectly separable, the hyperplane is chosen to be the farthest from the observations; this leads to the maximal margin classifier, where the margin is the minimal distance from the observations to the hyperplane. The points touching the margin are known as support vectors and are the only points that affect the classifier. In the non-separable case, the choice is performed by maximizing the number of correctly assigned classes. This can be carried out with the use of a soft margin, i.e., letting some observations be misclassified. The tolerance toward violations of the margin can be tuned by using a non-negative hyperparameter, usually named C. This approach is the basis of the support vector classifier. Finally, a non-linear decision boundary is necessary, in the most complex cases, to separate classes. A general approach to cope with this situation consists of enlarging the feature space. This can be achieved using basis expansions such as polynomials, but computations can become quickly prohibitive. In SVMs, the expansion of the feature space is accomplished efficiently with kernels, which are functions that quantify the similarity between two observations. Typical kernels are polynomial or radial. In our work, we employed linear, degree three polynomial, and radial basis functions to support vector classifiers. Adding to this, and to reduce the computational burden, for polynomial and radial basis kernels (RBK), the Nystroem method [55,56], which approximates the kernel by subsampling the data, was employed.

### 2.7. Stochastic Gradient Boosting

The second technique employed was boosting [22,58], which is a powerful learning method based on the combination of many simple models. The basic idea is to sequentially apply a “weak” learner to modified versions of the initial data. In our work, decision trees were used as weak classifiers, but any method better than random guessing can be used. Each time a tree is built, the data were modified by applying weights to increase the influence of misclassified observations. The final classification was performed through a weighted majority vote [59,60]. This basic idea can be improved by using a stagewise gradient descent procedure [61] and by incorporating randomness by subsampling the data at each iteration. This leads to the stochastic gradient boosting methods employed here [58].

## 3. Results

### 3.1. Canine Mammary Tumors

**Figure 1:**
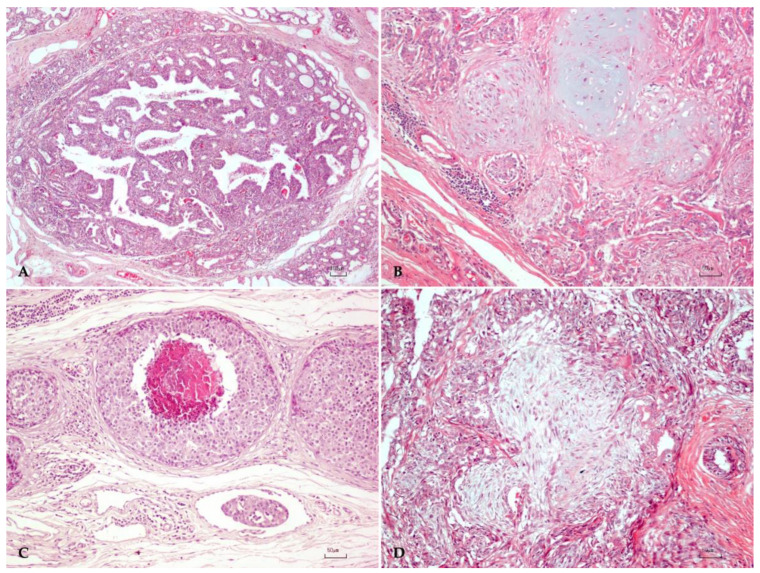
Canine mammary tumor. (**A**) Simple mammary adenoma characterized by a nodular, well-demarcated, focal neoplasm composed of luminal epithelial cells arranged in tubules; H&E, bar: 100 µm. (**B**) Benign mixed tumor characterized by the proliferation of luminal epithelial cells admixed with spindle myoepithelial cells and foci of cartilage; H&E, bar: 50 µm. (**C**) Simple comedocarcinoma characterized by a central necrotic area surrounded by neoplastic luminal epithelial; H&E, bar: 50 µm. (**D**) Complex carcinoma with the proliferation of malignant luminal epithelial cells and benign spindle myoepithelial cells; H&E, bar: 50 µm.

At histopathology, a morphologically heterogeneous group of lesions were classified as follows: 20 benign tumors, including 8 benign mixed tumors, 9 complex adenomas, 3 simple adenomas, and 24 malignant neoplasms, including 11 complex and 13 simple carcinomas (4 tubular, 5 tubulopapillary, 3 solid and 1 comedocarcinoma). The representative H&E-stained images from the different CMTs showing typical benign and malignant CMTs are illustrated in Figure 1. The proposed framework was first evaluated on a standard and challenging BreakHis dataset comprising 9109 images with different magnifying factors from human breast cancer patients.

### 3.2. Performance of the Convolutional Neural Networks Models

The accuracy and the performances of the proposed framework were first validated on the BreakHis dataset comprising a large number of human breast tumor images [37,38]. As a result, test accuracies for distinguishing benign and malignant tumors upon H&E classification of the BreakHis dataset ranged from 0.86 to 0.91, considering all combinations of feature extractors, classifiers, and testing strategies (Table 1 and Table S1 in Supplementary Materials).

As reported in Table 1, the best performance was observed using EfficientNet as a feature extractor followed by a SVM algorithm with radial basis kernels and with the simple augmentation strategy being repeated 6 times, with a testing accuracy of 0.9 to 0.91 for the center-crop and ten-crop testing, respectively. Furthermore, VGG16 and Inception coupled with a linear SVM, both with simple and advanced repeated 6-time augmentation strategies, resulted in a comparable accuracy when ten-crop testing was used.

The application of the same strategies in the CMTD resulted in mean testing accuracies ranging from 0.63 to 0.84 for the single-crop testing and from 0.64 to 0.85 for the ten-crop testing across all architectures (Table 2 and Table S1 in Supplementary Materials).

In particular, the framework using EfficientNet as a feature extractor coupled with a SVM and the simple augmentation strategy being used and repeated six times, resulted in the best performing one, with testing accuracies ranging from 0.83 and 0.84 for the center-crop testing and from 0.84 to 0.85 for the ten-crop testing. The other two tested architectures have similar performances to each other, with VGG16 being slightly ahead but still inferior to EfficientNet, probably as it works with a greater input size that could help to grasp more features.

The data augmentation strategies improve the CNNs’ performance, especially for the more powerful classifiers, while the more complicated approach requires the generation of a higher number of images to become closer to the others. As for the testing strategy, there is a slight advantage of the ten-crop method with respect to the center-crop method.

## 4. Discussion

Considering the importance of a histopathological diagnosis in the management of oncologic patients, considerable efforts have been made for developing robust, precise, and automated CAD systems for humans. More specifically, CNNs are becoming the standard approach for the classification of histological images related to breast cancer [62]. In veterinary medicine, the increase in the incidence of neoplastic disease represents a relentless challenge for veterinary oncology specialists and many efforts have been made in the ongoing research to increase the earliness of diagnosis and the survival time in dogs harboring mammary tumors [1,2,3,4,5,6,7,8,9,63]. However, considering the high incidence of canine mammary tumors, no significant effort has been made in veterinary pathology for the development of CMT-oriented CAD systems.

In this work, three different CNN architectures (VGG16, Inception v3, and EfficientNet), coupled with two different classifiers (support vector machines and stochastic gradient boosting), were tested and used to explore the ability to distinguish between benign and malignant canine mammary tumors on hematoxylin–eosin-stained images.

The application of the abovementioned developed architectures on the public dataset of the Breast Cancer Histopathological Database (BreakHis) comprising microscopic images of human breast tumors, with each sample labeled as either benign or malignant [8], provided a 91% classification accuracy rate. Interestingly, Li and coauthors in 2020 achieved an accuracy rate of 83%, [64], while Kumar in 2020, with a fused framework based on VGG16 CNN, used as a feature extractor for different classifiers, obtained an accuracy of 97% [42]. More recently, Liu in 2021 with concatenated VGG16, based on filtering and denoising the BreakHis images, obtained a 98.89% accuracy [65].

In the present work, the application of the same strategies to the CMT dataset resulted in mean testing accuracies ranging from 0.63 to 0.84 for single-crop testing, and from 0.64 to 0.85 for ten-crop testing across all architectures. Interestingly, Kumar and coauthors proposed a dataset of CMT histopathological images, namely CMTHis, comprising a total of 352 images from 20 benign and 24 malignant CMT cases, evaluated using a framework based on VGGNet-16 coupled with a SVM and Random Forest, with different strategies of data augmentation, obtaining a diagnostic accuracy of 93% [42].

Furthermore, differently from our dataset, the CMTHis dataset consisted only of simple and ductal-associated, except for fibroadenoma, canine mammary tumors, while in our cases 17 benign and 11 malignant neoplasms were of the complex type, and 16 were of the simple type, reflecting the frequencies of histotypes commonly diagnosed in dogs. In particular, mixed neoplasms are the most frequent neoplasia in female dogs and are characterized by the proliferation of both luminal epithelial and interstitial myoepithelial elements admixed with foci of mesenchymal tissues such as cartilage, bone, and fat [36,66]. Thus, the different diagnostic accuracy obtained in our work could be related to the several different morphologies present in our dataset, underlining the importance of considering the complexity of histological images in veterinary medicine, in which mixed neoplasms are common.

However, our study displayed similar results to those of Kumar when analyzing images at the same magnification (i.e., 400×), ranging from 81.63 to 83.35 accuracy [42]. In machine learning, studies with inadequate samples suffer from overfitting of data, while the increase in sample size increases the accuracy of prediction. as suggested by Rajput and coauthors [67]. In our database, a great number of images were evaluated (1056 images from 44 CMT instead of the 88 CMTHis pictures), supporting the validity of our model.

In addition, the lower accuracy using the BreakHis image dataset of the evaluated framework, compared to that using the *CMTD* and CMTHis databases, could be related to the low number of canine mammary tumor images compared to breast images [42].

Several studies have examined the role of data augmentation in deep learning, as this method generates a high amount of data and the building of more generalized models. In particular, Spanhol and collaborators, using CNNs to classify images from the BreakHis database [37,38], employed an approach based on the extraction and the combination of patches of the original high-resolution images [26,37,38].

Moreover, Spanhol and collaborators made further improvements by combining different CNNs, outperforming previous machine learning approaches based on hand-crafted textural descriptors. In subsequent work, the authors employed the CNN as a feature extractor to train a classifier, showing again how classical approaches are outperformed, as are task-specific CNNs sometimes [26,28,37]. Araújo and coauthors in 2017, proposing a custom CNN to access information at different scales, obtained good results for the Bioimaging 2015 challenge dataset, both with the pure CNN and using the CNN as a feature extractor coupled with a SVM classifier [30]. Han and collaborators in 2017 developed a class structure-based deep convolutional neural network, which can be trained end-to-end and does not rely on patches [32]. The applied network dramatically improved the accuracy of previous models in both binary and multi-class tasks for the BreakHis dataset. Alom in 2019 proposed a method based on the inception recurrent residual convolutional neural network model for binary and multi-class classification tasks [33]. They explored different training and analysis strategies, obtaining good results over different benchmark datasets (e.g., accuracies larger than 97% for the BreakHis binary classification task for all magnifications).

In our study, the accuracy and the positive and negative predictive values using the EfficientNet-B4 architecture as a feature extractor had the best performances, albeit presenting only a slight difference, among the three tested options. VGG16 showed a slightly superior accuracy of performance compared to that of InceptionNetV3, but it was still inferior to that of EfficientNet, probably as the latter is more refined and works with a greater input size that could help to grasp more features.

This data are in agreement with what has been recently reported by Kallipolitis, where EfficientNets architectures, and in particular B4, have the best performances compared to InceptionNetV3 and VGG16 [68].

Data augmentation is a useful technique to enhance the performance of CNNs, especially when the available data (i.e., images) are limited or the model’s performance needs to be improved [69]. In our work, the data augmentation strategies discussed improve the CNNs’ performance, especially for the more powerful classifiers. In particular, the simpler data augmentation strategy, consisting only of a random crop, rotations, and reflections, generally leads to slightly better results. The more complicated approach used in our work instead requires the generation of a higher number of images to obtain a similar performance to that of the others. An exception is the case of the linear support vector classifier, which gives good results across all options as previously described by Kumar 2020 [42]. In our study, an aggressive data augmentation strategy did not lead to substantial improvements but rather worsened the results when the number of artificially modified images was small, as in the case of VGG16, a situation that was reversed when considering the InceptionNetV3 architecture.

As for the testing strategy, there is a slight advantage of the ten-crop method compared to the center-crop method. This is tentatively explained by the fact that the ten crops strategy is probably able to acquire more information, as all regions of the image are analyzed.

Overall, our results suggest that CAD systems using deep learning techniques, specifically convolutional neural networks, have great potential for improving the accuracy of histopathological diagnoses in both human and veterinary medicine. Although the accuracy rates were not like those in human breast cancer histopathology, our results are promising, also taking in consideration the limited number of studies regarding CMT. Furthermore, we provide the larger canine mammary tumor benchmark dataset—namely *CMTD*—containing over 1000 high-resolution histological images of the most common benign and malignant tumors for developing and evaluating computer-aided diagnostic systems for testing other state-of-the-art models for histopathological image classification.

## 5. Conclusions

Convolutional neural networks have been used in various applications related to breast cancer, such as in medical imaging for the diagnosis and prognosis of breast cancer. In veterinary medicine, several manuscripts have described the potentiality of this technology, but no consistent efforts have been undertaken regarding canine mammary tumors [69,70,71,72,73,74,75]. Deep learning-based algorithms can assist the pathologist in classifying tumors on standard hematoxylin and eosin images. Therefore, publicly available datasets have become increasingly popular, as they reduce annotation costs for recurring pathological research questions and improve the comparability of computer-aided systems developed on these datasets. Overall, the results of this study demonstrate the potential of CNNs and CAD systems to aid in the diagnosis of canine mammary tumors, while the available canine mammary tumor benchmark dataset will be of great benefit to the veterinary research community. Therefore, further studies with a large number of CMT patients and histopathological images are required to prove the efficacy of the proposed framework in the binary classification of CMTs.

In conclusion, the encouraging results obtained in this study provide an interesting perspective on the integration of artificial intelligence and machine learning technologies in cancer-related research, offering a valuable starting point for further research in this area.

## Figures and Tables

**Table 1:** BreakHis dataset; the mean center-crop and the ten-crop accuracies for the best combination of feature extractor, classifier and augmentation strategy.

Feature Extractor	Classifier	Augmentation	Center Crop Accuracy	Ten Crops Accuracy
VGG16	Linear SVM	Advanced 1×	0.89 ± 0.01	0.91 ± 0.01
Inception	Linear SVM	Advanced 6×	0.88 ± 0.02	0.91 ± 0.02
EfficientNet	RBF SVM	Base 6×	0.9 ± 0.02	0.91 ± 0.02

**Table 2:** CMT dataset: the mean center-crop and the ten-crop accuracies for the best combination of feature extractor, classifier, and augmentation strategy.

Feature Extractor	Classifier	Augmentation	Center Crop Accuracy	Ten Crops Accuracy
VGG16	Linear SVM	Base 6×	0.78 ± 0.03	0.82 ± 0.03
Inception	Linear SVM	Advanced 6×	0.78 ± 0.03	0.81 ± 0.04
EfficientNet	Poly SVM	Base 6×	0.84 ± 0.02	0.84 ± 0.03
	RBF SVM	Base 6×	0.83 ± 0.03	0.85 ± 0.03

## Data Availability

The data are available upon request from the corresponding author.

## Supplementary Materials

The following supporting information can be downloaded at: https://www.mdpi.com/article/10.3390/ani13091563/s1, File S1: Canine Mammary Tumor Dataset; Figure S1: Depiction of the feature extraction process for VGG16 architecture; GAP: global average pooling. ⨁ denotes the concatenation operation. Figure S2. Depiction of the feature extraction process for Inception V3 architecture; BN: batch normalization. ReLu: Rectified linear units (ReLu); GAP: global average pooling; ⨁ denotes the concatenation operation. Figure S3. Depiction of the feature extraction process for the EfficientNet-B4 architecture; GAP: global average pooling; ⨁ denotes the concatenation operation. Table S1: Performances of CNN on BreakHis and *CMTD*; *CMTD*: canine mammary tumor dataset.
