# Supplementary figures and images for: Canine Mammary Tumor Histopathological Image Classification via Computer-Aided Pathology: An Available Dataset for Imaging Analysis (part 2 of 2)
Source: Animals (Basel). 2023 May 6;13(9):1563. doi: 10.3390/ani13091563 (PMC10177203; doi:10.3390/ani13091563)

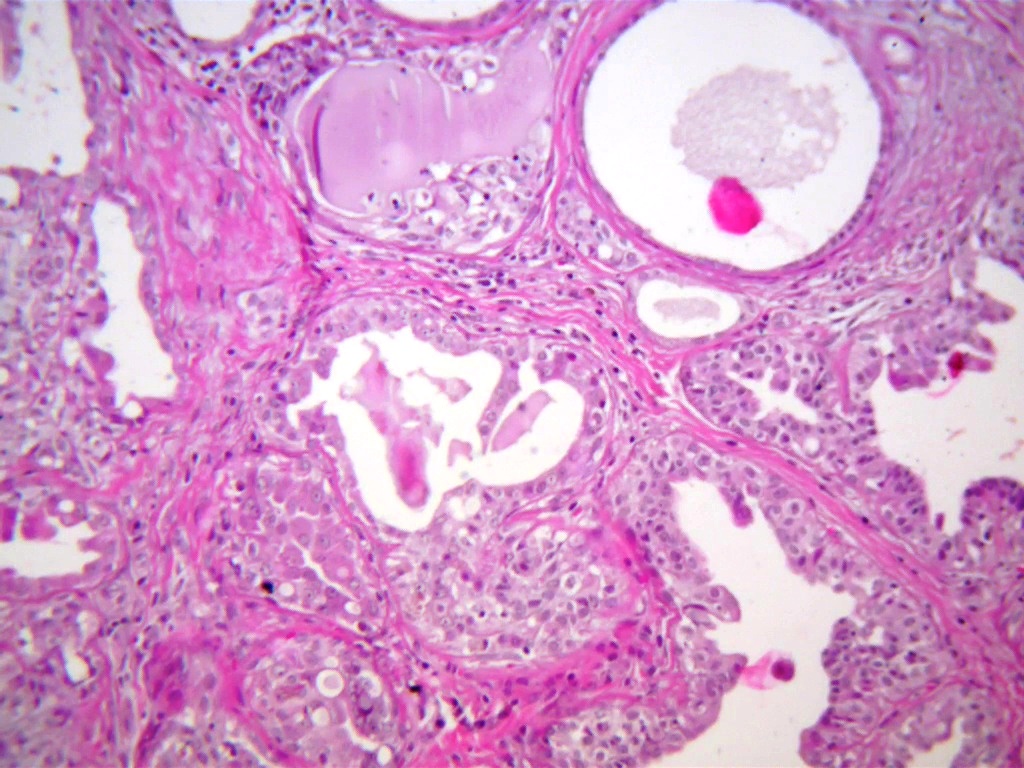

Supplement: Supplementary file 1 [file animals-13-01563-s001.zip › supplementary files/File S1 Canine Mammary Tumor Dataset/benign/Benign mixed tumor_99_369V2_FRM_000 (11).jpg]

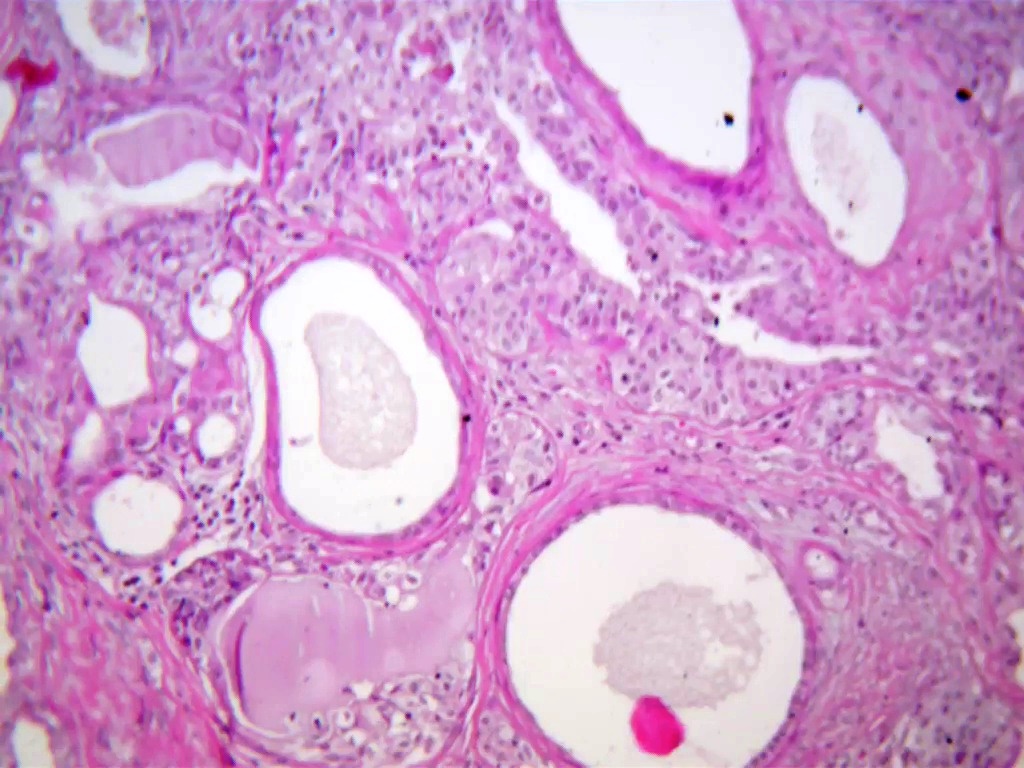

Supplement: Supplementary file 1 [file animals-13-01563-s001.zip › supplementary files/File S1 Canine Mammary Tumor Dataset/benign/Benign mixed tumor_99_369V2_FRM_000 (12).jpg]

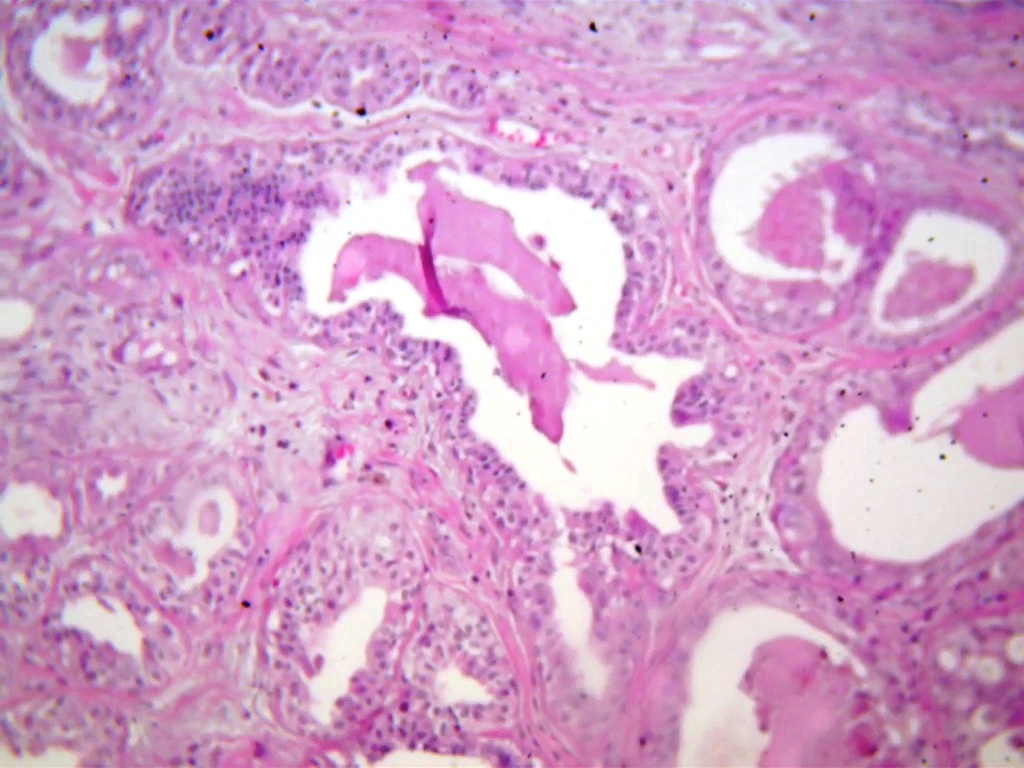

Supplement: Supplementary file 1 [file animals-13-01563-s001.zip › supplementary files/File S1 Canine Mammary Tumor Dataset/benign/Benign mixed tumor_99_369V2_FRM_000 (13).jpg]

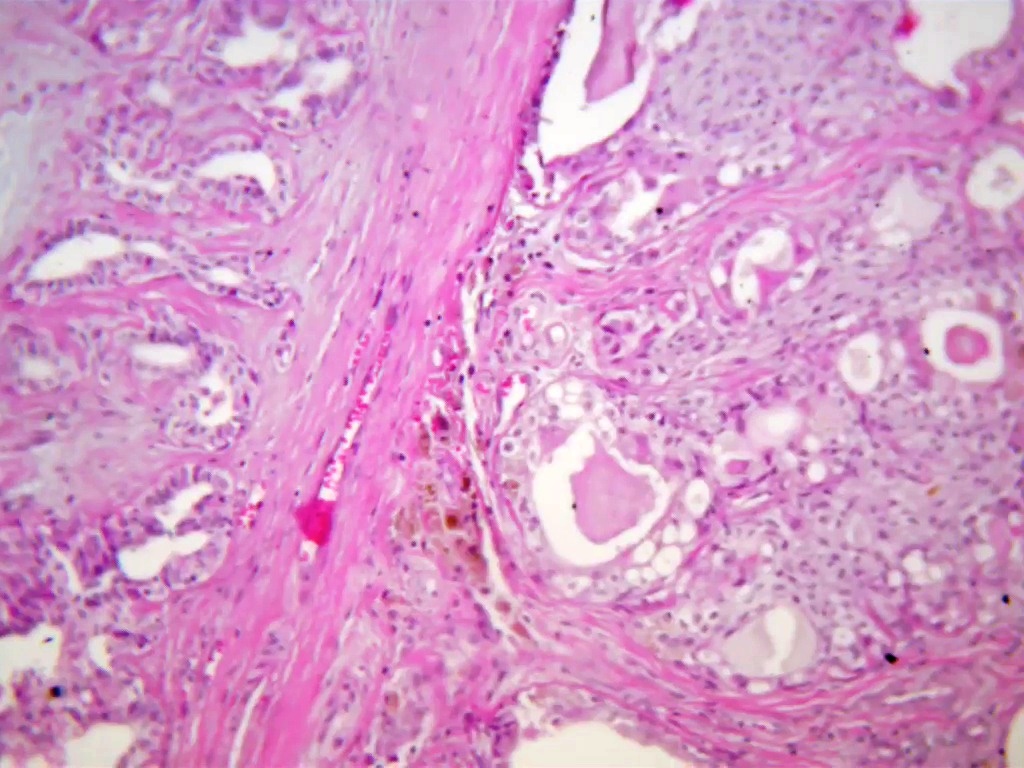

Supplement: Supplementary file 1 [file animals-13-01563-s001.zip › supplementary files/File S1 Canine Mammary Tumor Dataset/benign/Benign mixed tumor_99_369V2_FRM_000 (14).jpg]

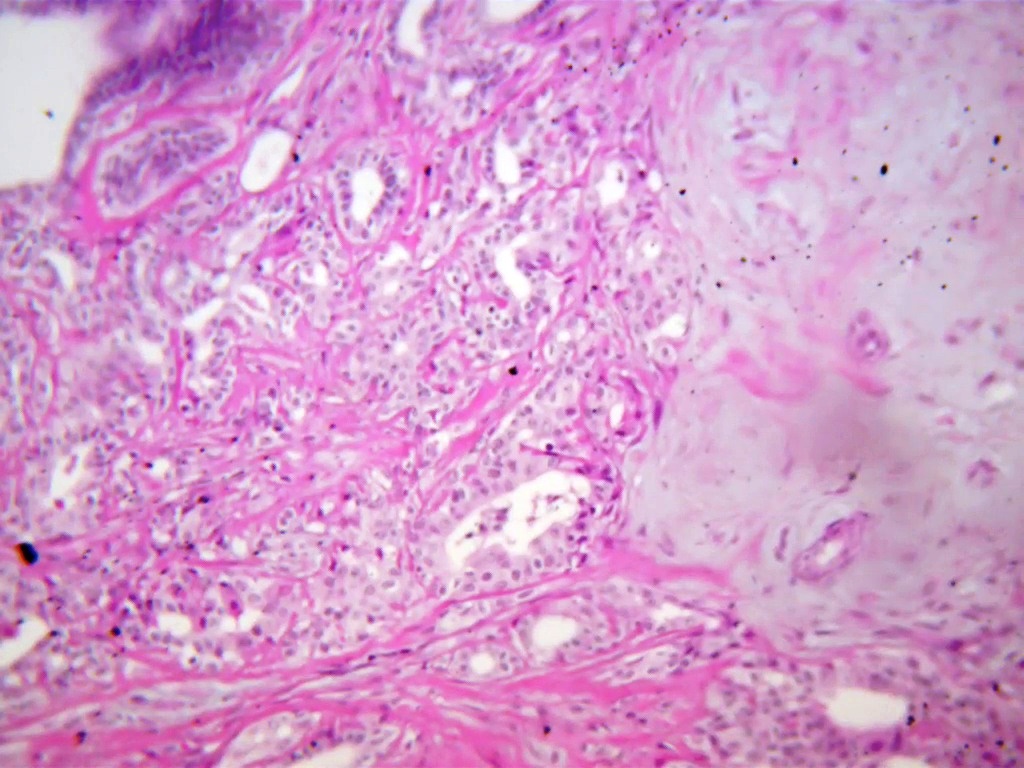

Supplement: Supplementary file 1 [file animals-13-01563-s001.zip › supplementary files/File S1 Canine Mammary Tumor Dataset/benign/Benign mixed tumor_99_369V2_FRM_000 (15).jpg]

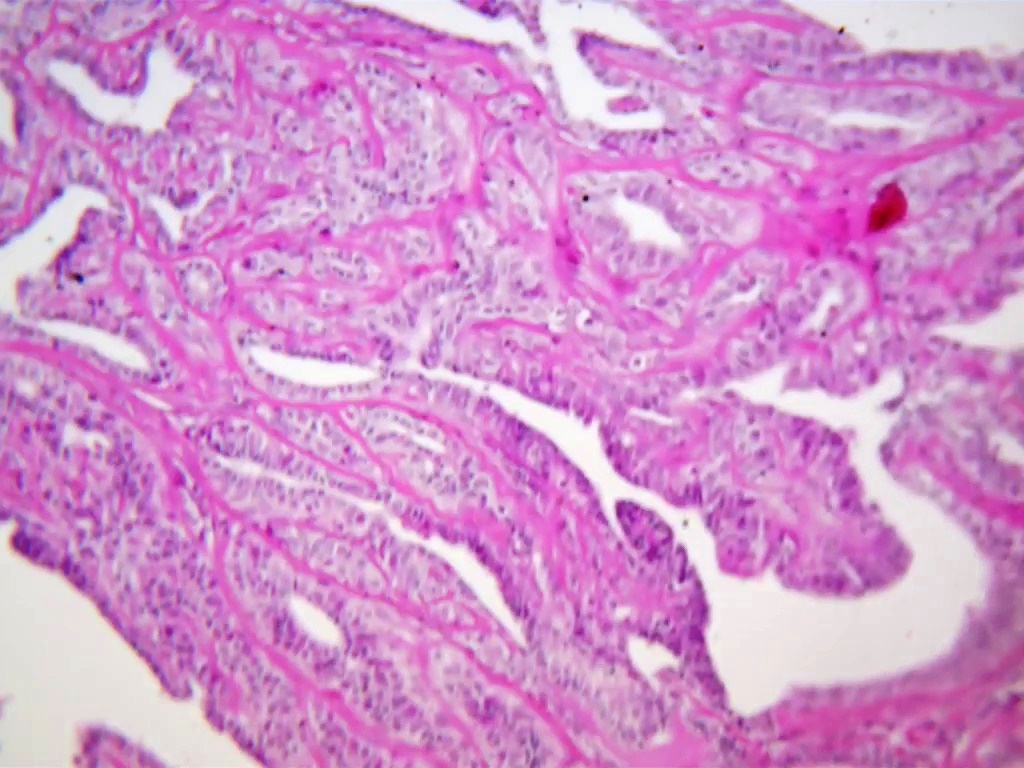

Supplement: Supplementary file 1 [file animals-13-01563-s001.zip › supplementary files/File S1 Canine Mammary Tumor Dataset/benign/Benign mixed tumor_99_369V2_FRM_000 (16).jpg]

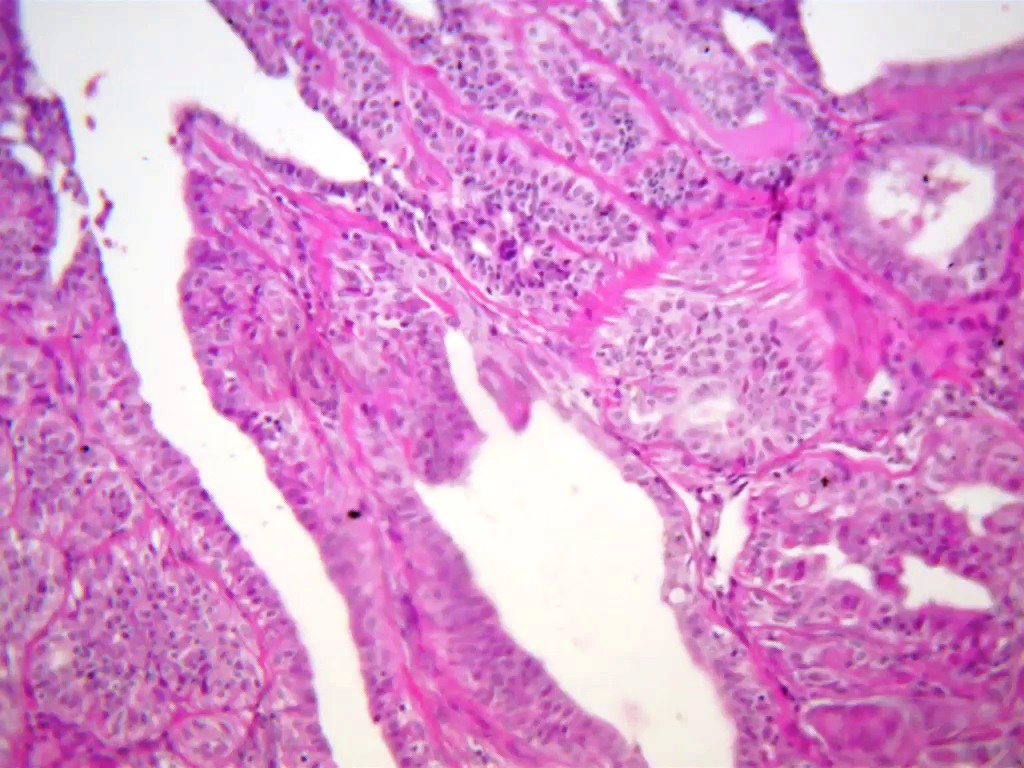

Supplement: Supplementary file 1 [file animals-13-01563-s001.zip › supplementary files/File S1 Canine Mammary Tumor Dataset/benign/Benign mixed tumor_99_369V2_FRM_000 (17).jpg]

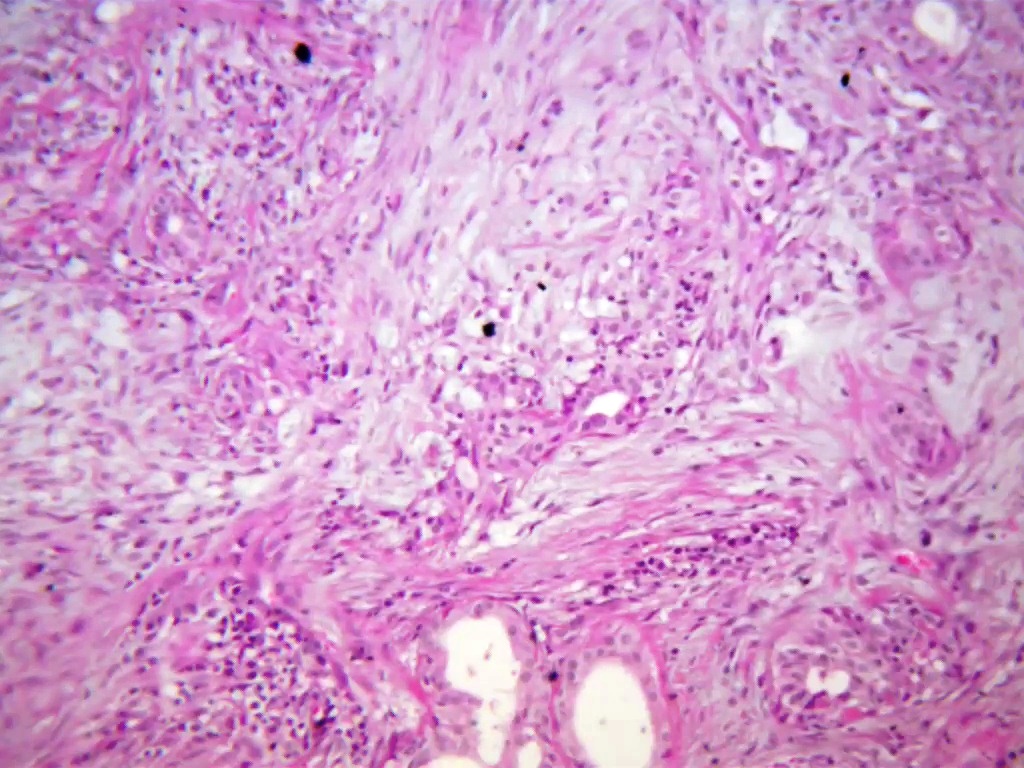

Supplement: Supplementary file 1 [file animals-13-01563-s001.zip › supplementary files/File S1 Canine Mammary Tumor Dataset/benign/Benign mixed tumor_99_369V2_FRM_000 (18).jpg]

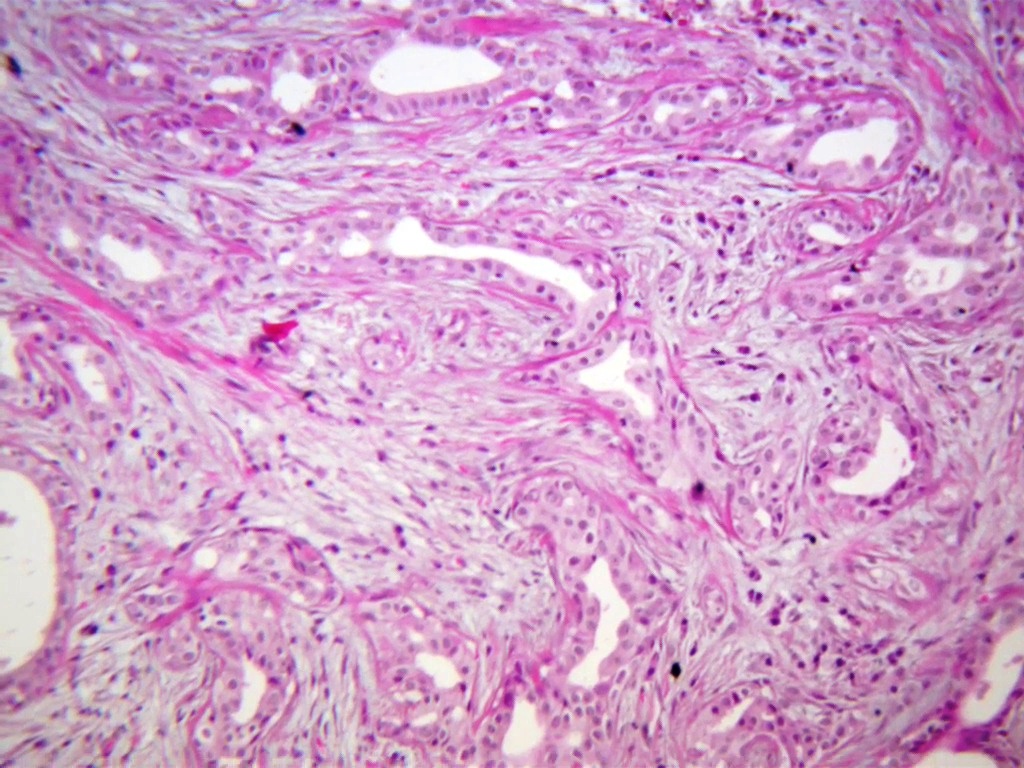

Supplement: Supplementary file 1 [file animals-13-01563-s001.zip › supplementary files/File S1 Canine Mammary Tumor Dataset/benign/Benign mixed tumor_99_369V2_FRM_000 (19).jpg]

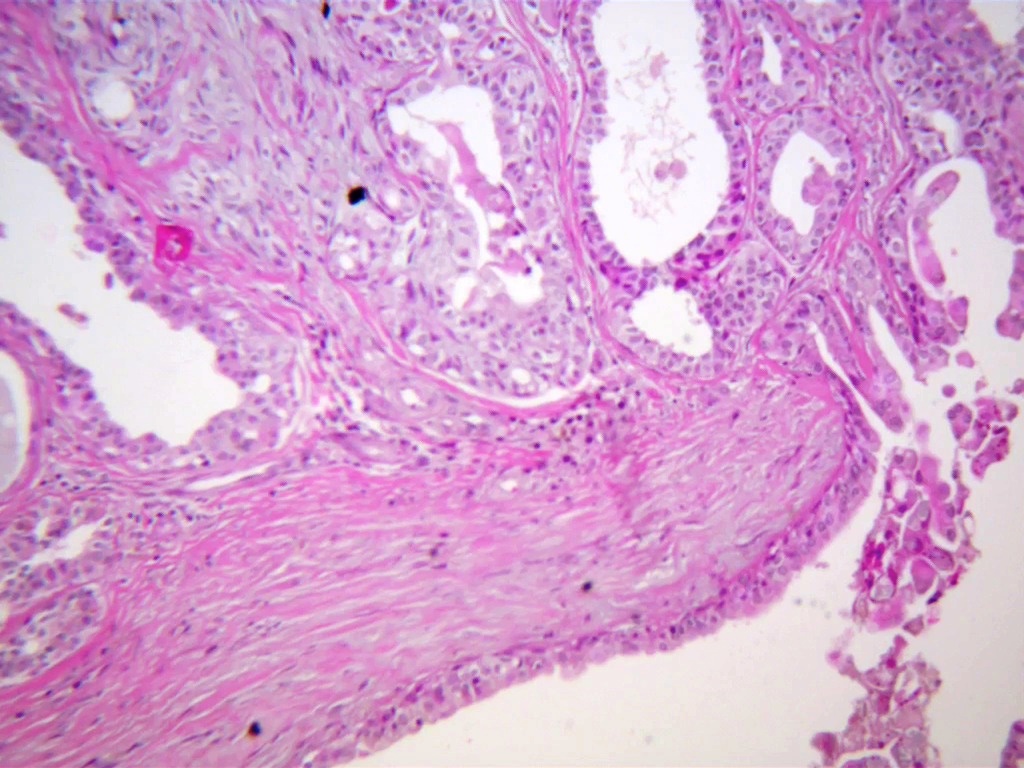

Supplement: Supplementary file 1 [file animals-13-01563-s001.zip › supplementary files/File S1 Canine Mammary Tumor Dataset/benign/Benign mixed tumor_99_369V2_FRM_000 (2).jpg]

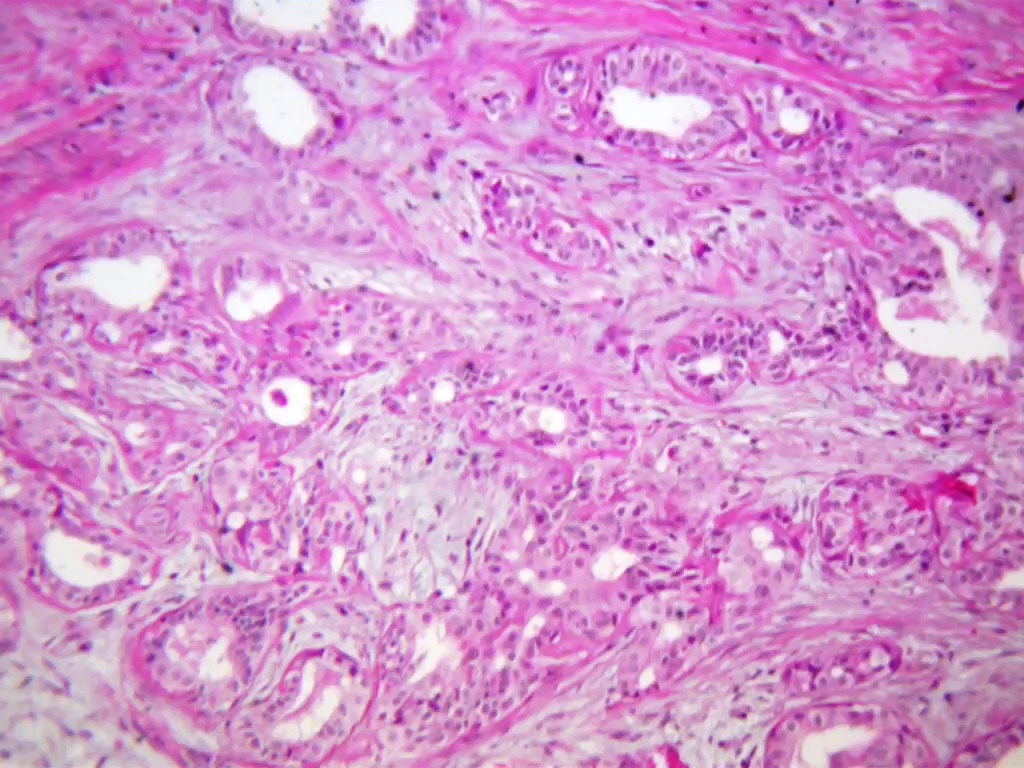

Supplement: Supplementary file 1 [file animals-13-01563-s001.zip › supplementary files/File S1 Canine Mammary Tumor Dataset/benign/Benign mixed tumor_99_369V2_FRM_000 (20).jpg]

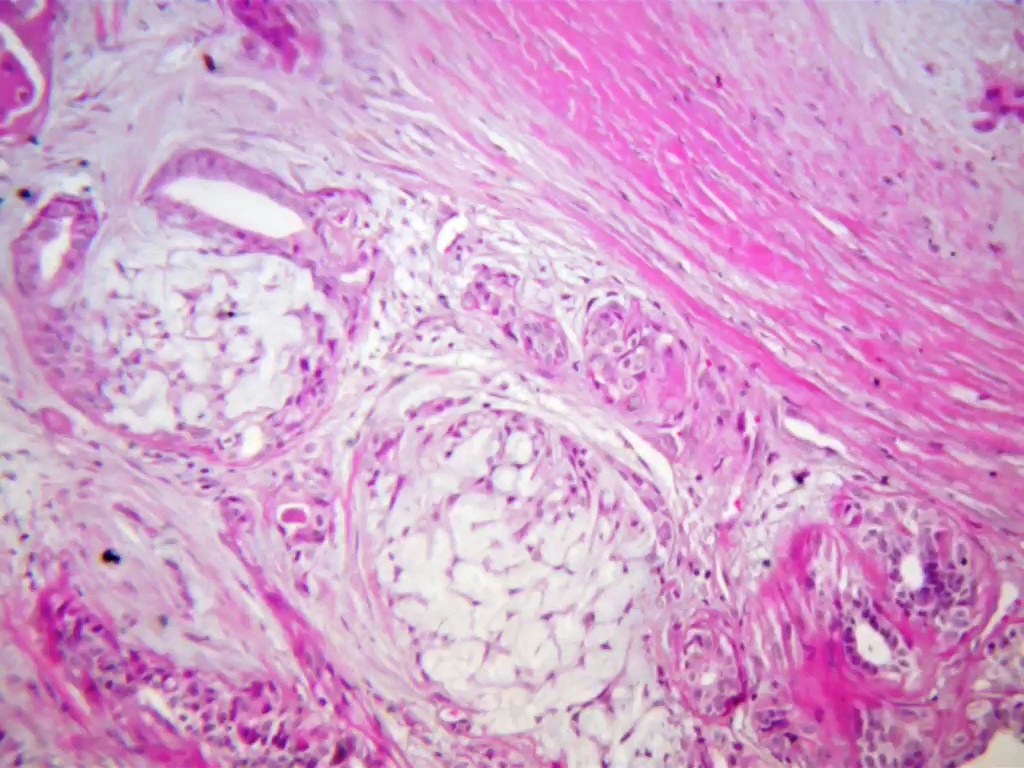

Supplement: Supplementary file 1 [file animals-13-01563-s001.zip › supplementary files/File S1 Canine Mammary Tumor Dataset/benign/Benign mixed tumor_99_369V2_FRM_000 (21).jpg]

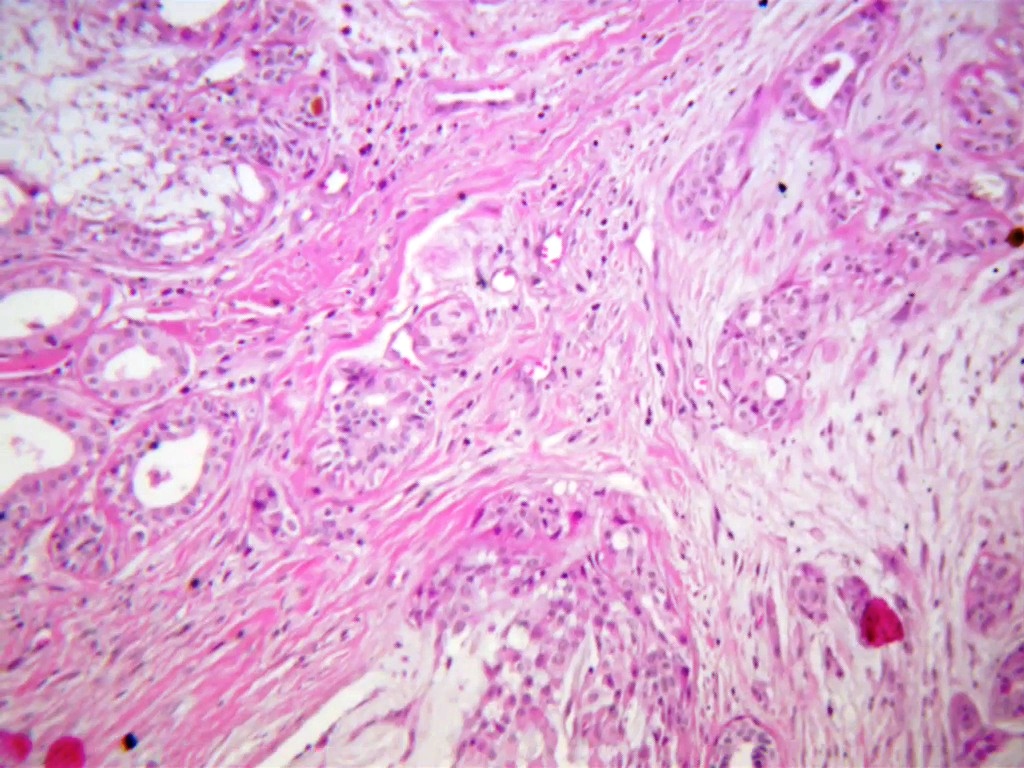

Supplement: Supplementary file 1 [file animals-13-01563-s001.zip › supplementary files/File S1 Canine Mammary Tumor Dataset/benign/Benign mixed tumor_99_369V2_FRM_000 (22).jpg]

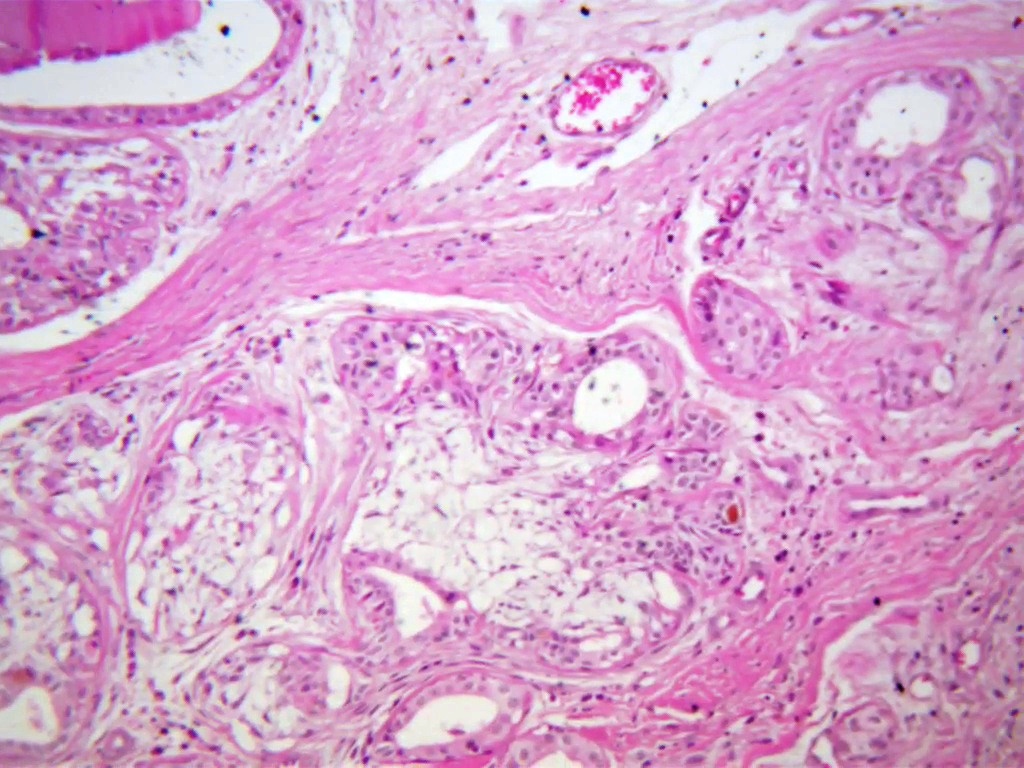

Supplement: Supplementary file 1 [file animals-13-01563-s001.zip › supplementary files/File S1 Canine Mammary Tumor Dataset/benign/Benign mixed tumor_99_369V2_FRM_000 (23).jpg]

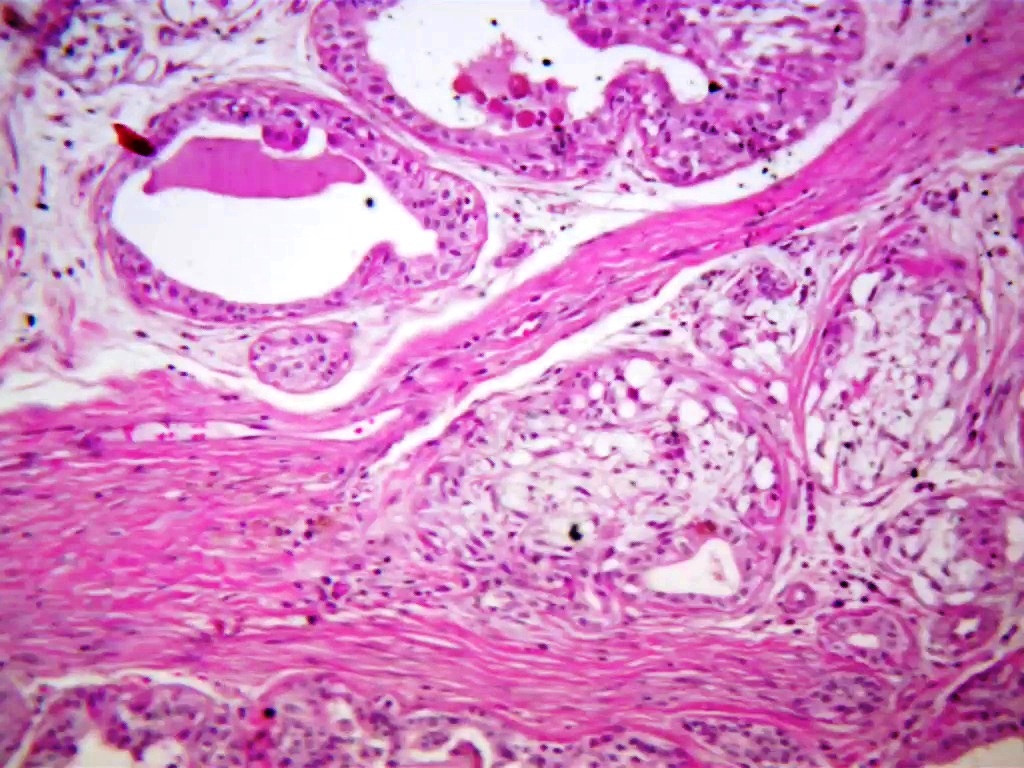

Supplement: Supplementary file 1 [file animals-13-01563-s001.zip › supplementary files/File S1 Canine Mammary Tumor Dataset/benign/Benign mixed tumor_99_369V2_FRM_000 (24).jpg]

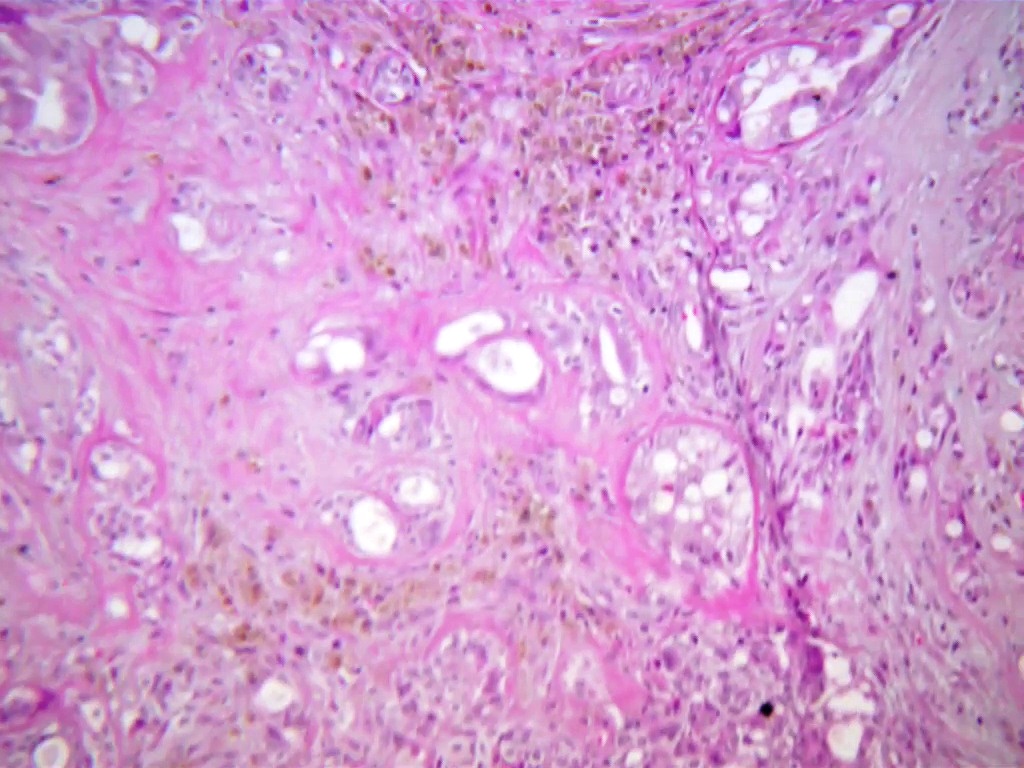

Supplement: Supplementary file 1 [file animals-13-01563-s001.zip › supplementary files/File S1 Canine Mammary Tumor Dataset/benign/Benign mixed tumor_99_369V2_FRM_000 (3).jpg]

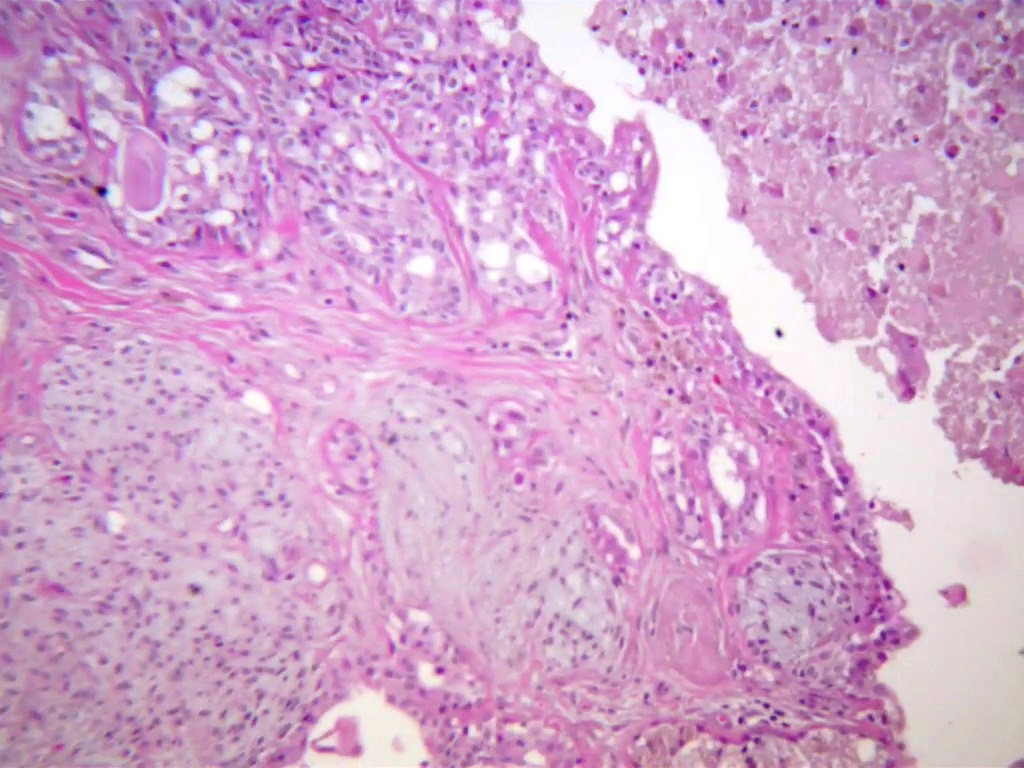

Supplement: Supplementary file 1 [file animals-13-01563-s001.zip › supplementary files/File S1 Canine Mammary Tumor Dataset/benign/Benign mixed tumor_99_369V2_FRM_000 (4).jpg]

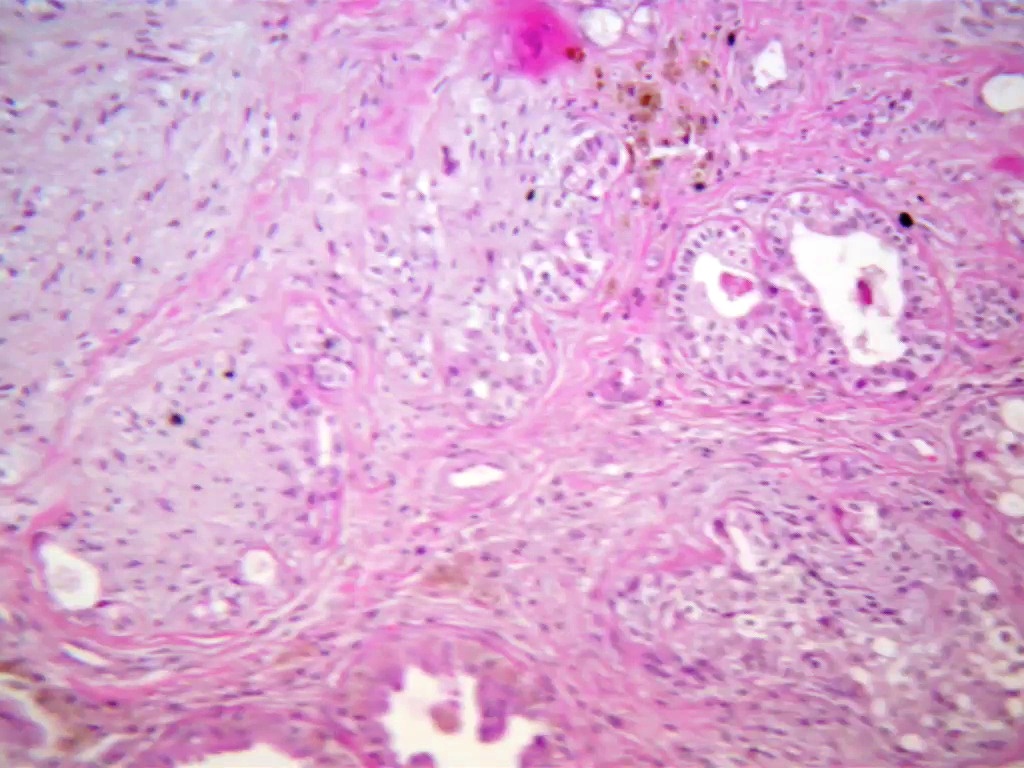

Supplement: Supplementary file 1 [file animals-13-01563-s001.zip › supplementary files/File S1 Canine Mammary Tumor Dataset/benign/Benign mixed tumor_99_369V2_FRM_000 (5).jpg]

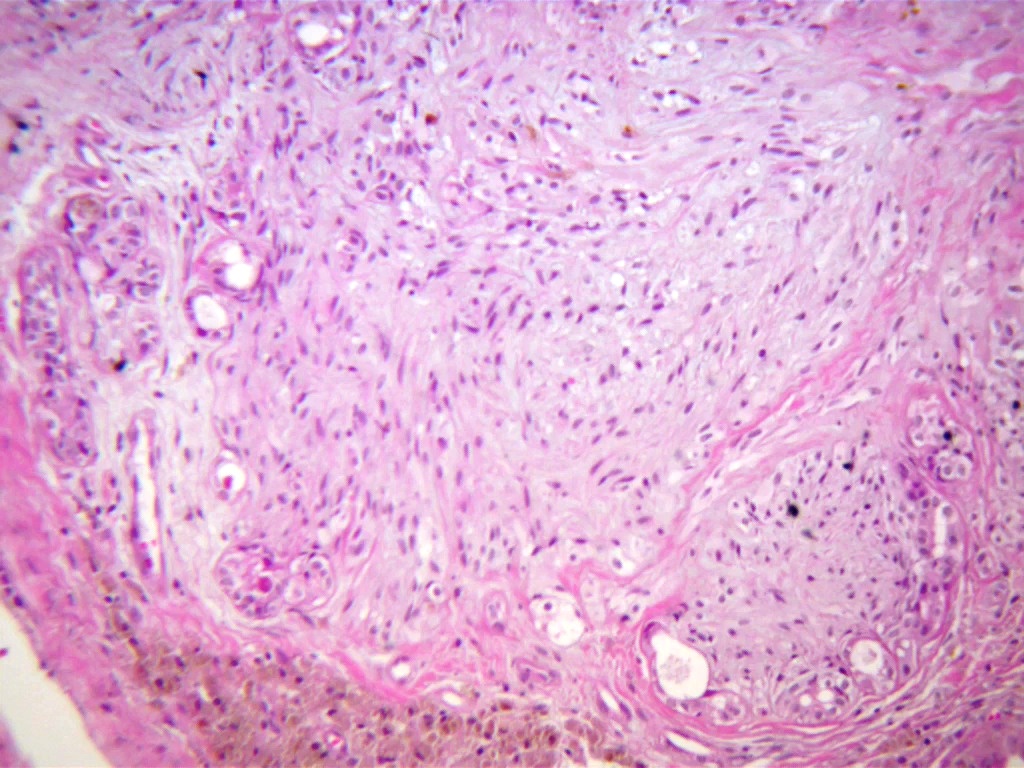

Supplement: Supplementary file 1 [file animals-13-01563-s001.zip › supplementary files/File S1 Canine Mammary Tumor Dataset/benign/Benign mixed tumor_99_369V2_FRM_000 (6).jpg]

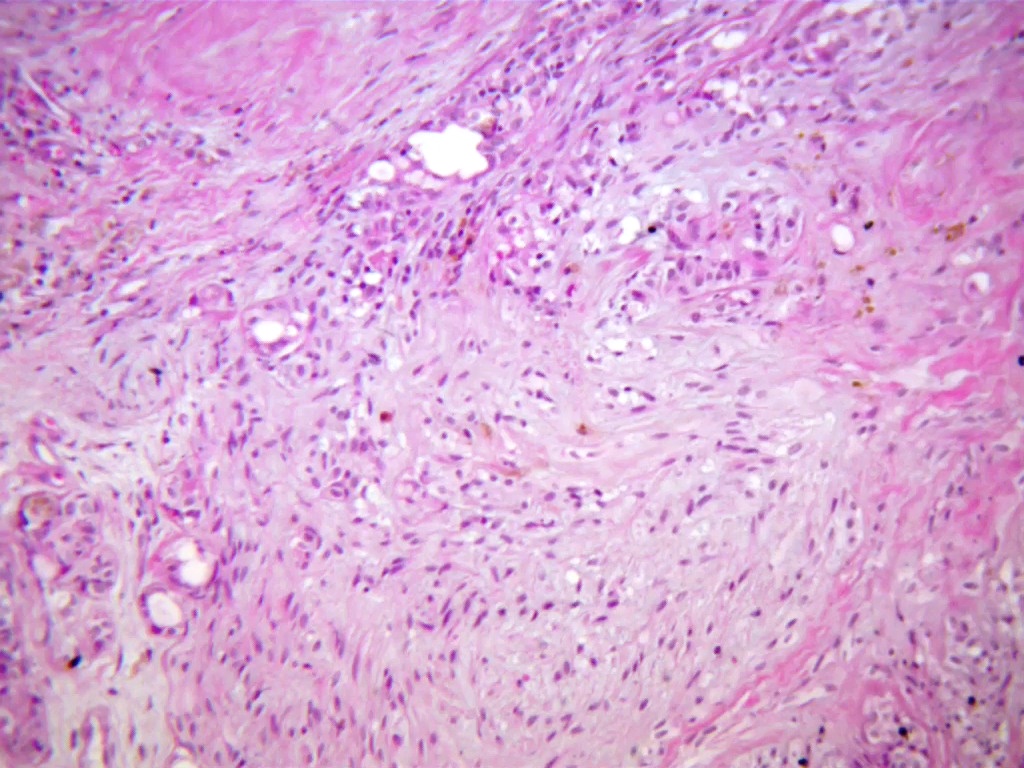

Supplement: Supplementary file 1 [file animals-13-01563-s001.zip › supplementary files/File S1 Canine Mammary Tumor Dataset/benign/Benign mixed tumor_99_369V2_FRM_000 (7).jpg]

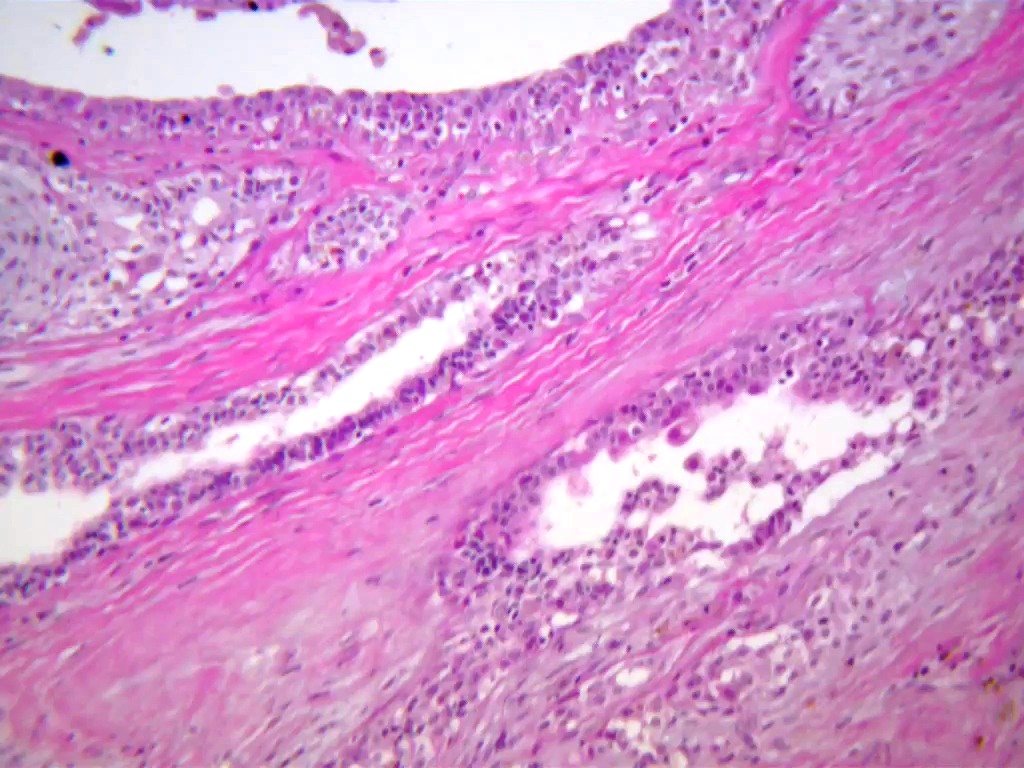

Supplement: Supplementary file 1 [file animals-13-01563-s001.zip › supplementary files/File S1 Canine Mammary Tumor Dataset/benign/Benign mixed tumor_99_369V2_FRM_000 (8).jpg]

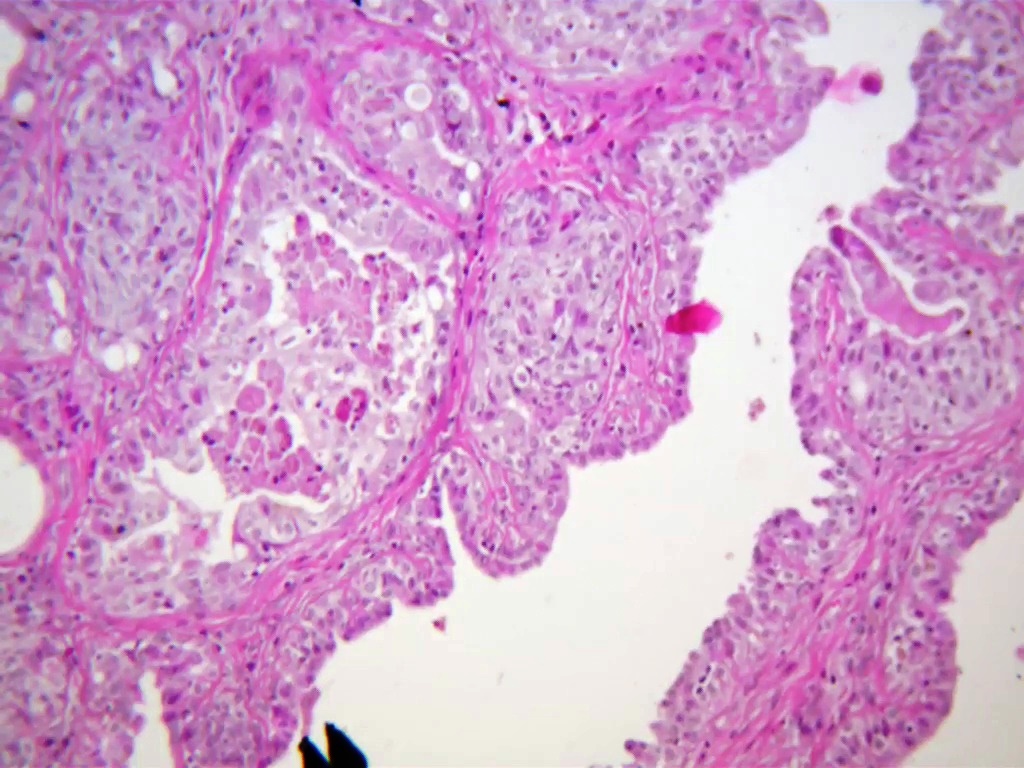

Supplement: Supplementary file 1 [file animals-13-01563-s001.zip › supplementary files/File S1 Canine Mammary Tumor Dataset/benign/Benign mixed tumor_99_369V2_FRM_000 (9).jpg]

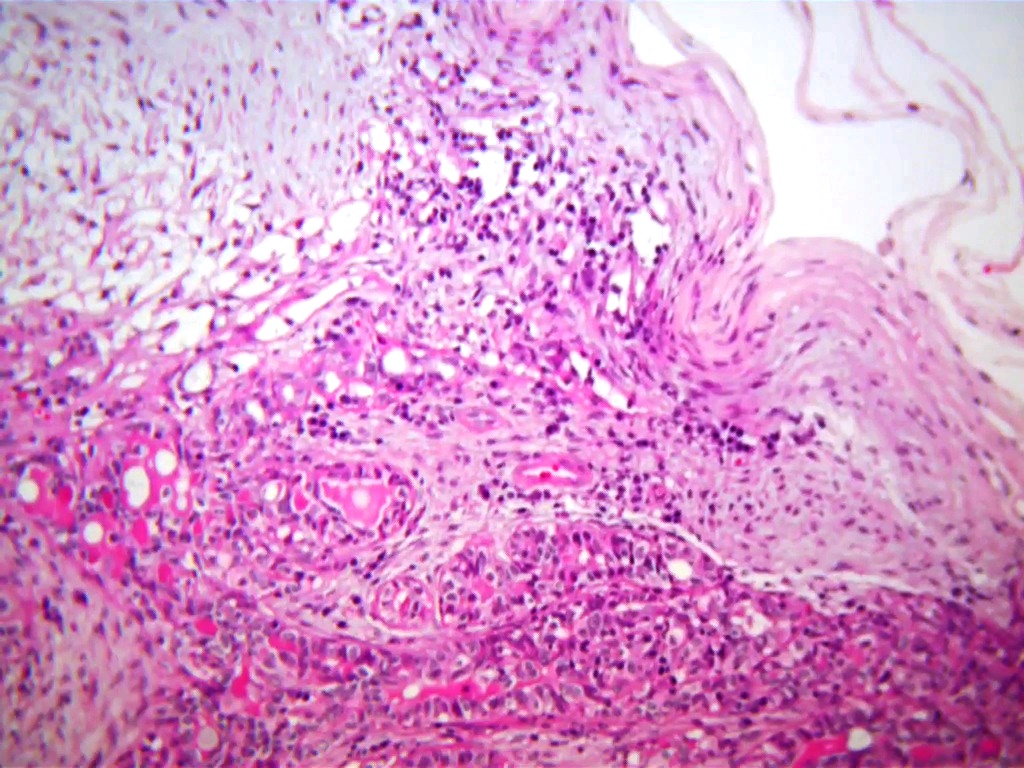

Supplement: Supplementary file 1 [file animals-13-01563-s001.zip › supplementary files/File S1 Canine Mammary Tumor Dataset/benign/Benign mixed tumor_FRM_001 (1).jpg]

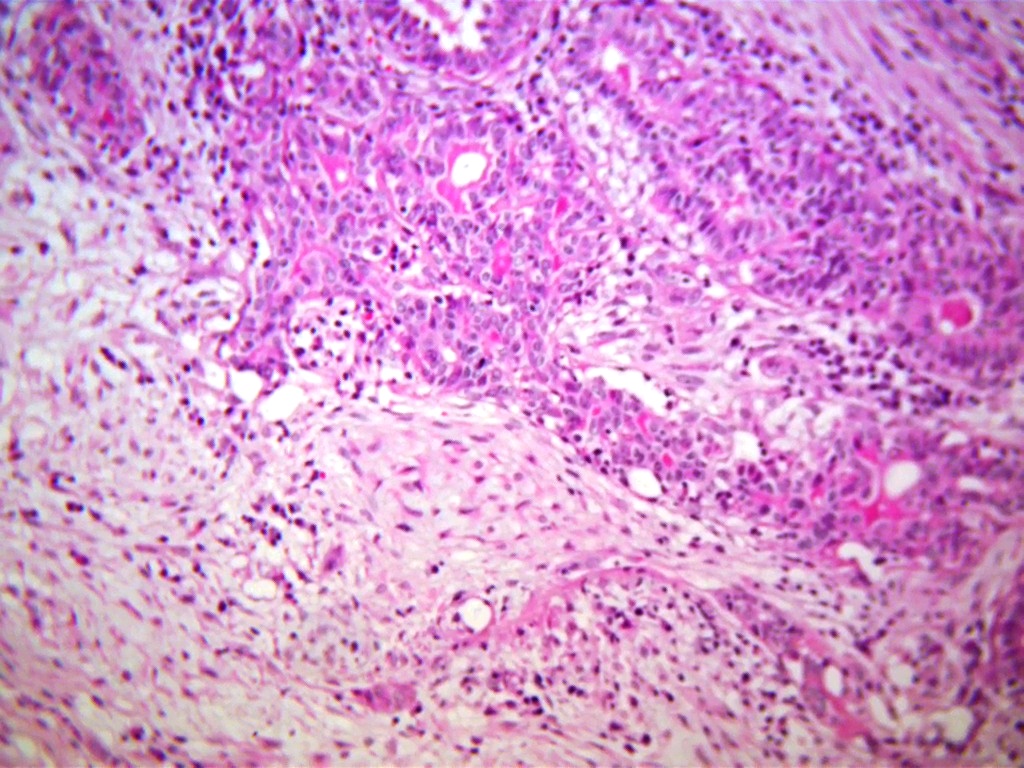

Supplement: Supplementary file 1 [file animals-13-01563-s001.zip › supplementary files/File S1 Canine Mammary Tumor Dataset/benign/Benign mixed tumor_FRM_001 (10).jpg]

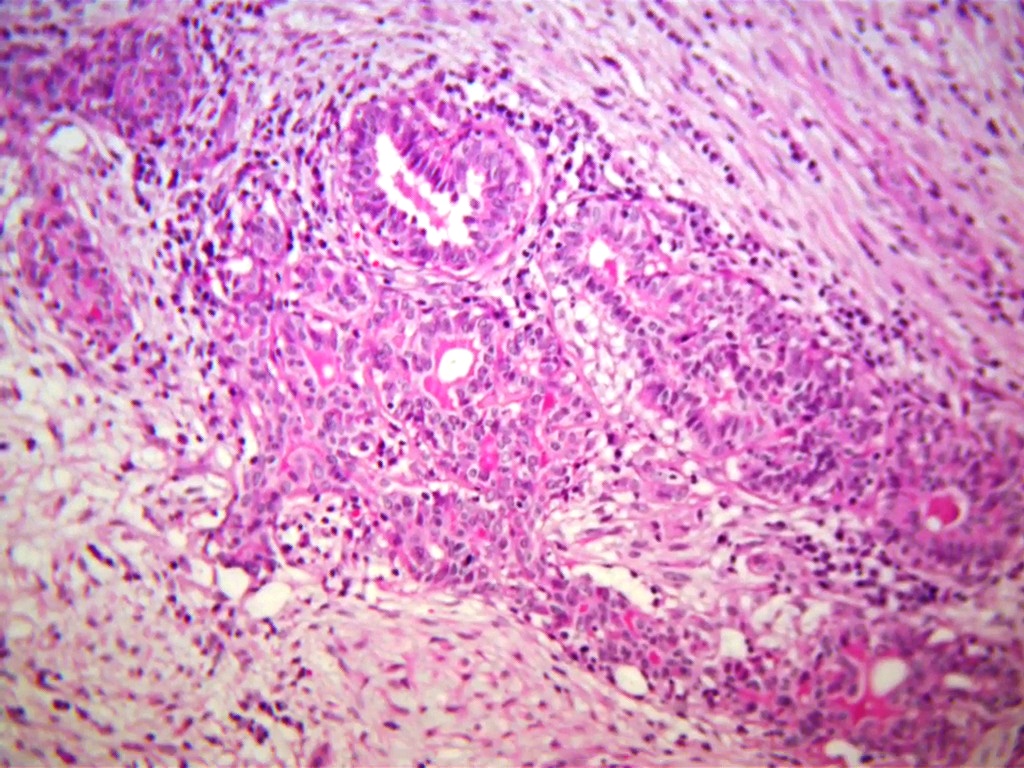

Supplement: Supplementary file 1 [file animals-13-01563-s001.zip › supplementary files/File S1 Canine Mammary Tumor Dataset/benign/Benign mixed tumor_FRM_001 (11).jpg]

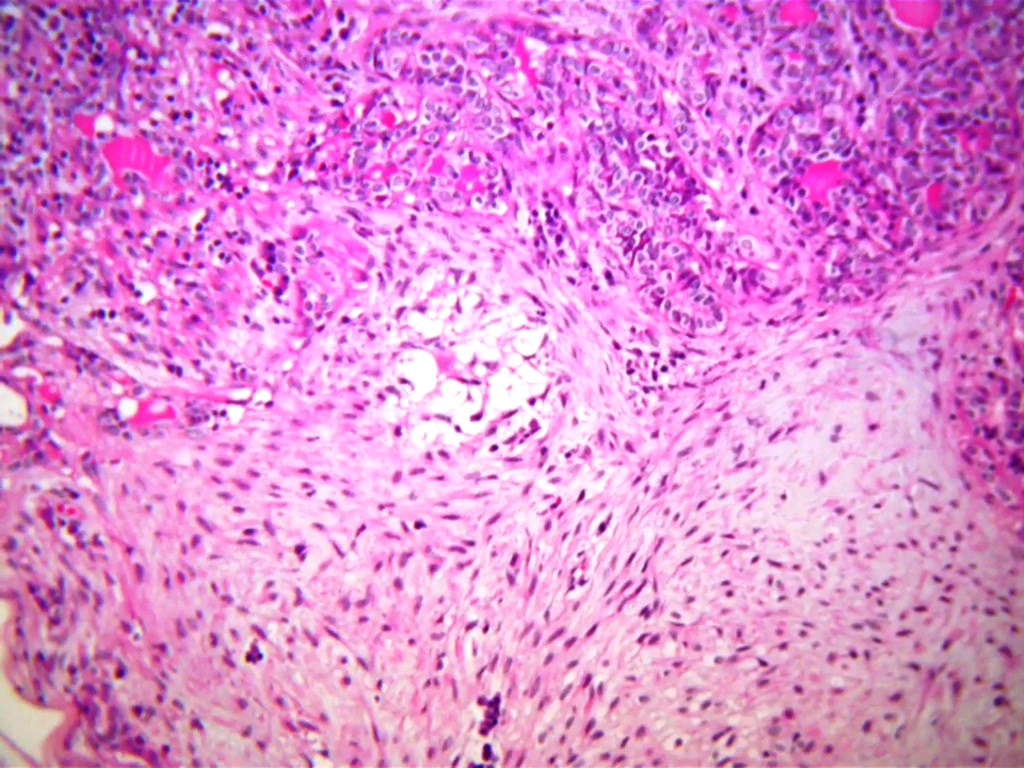

Supplement: Supplementary file 1 [file animals-13-01563-s001.zip › supplementary files/File S1 Canine Mammary Tumor Dataset/benign/Benign mixed tumor_FRM_001 (12).jpg]

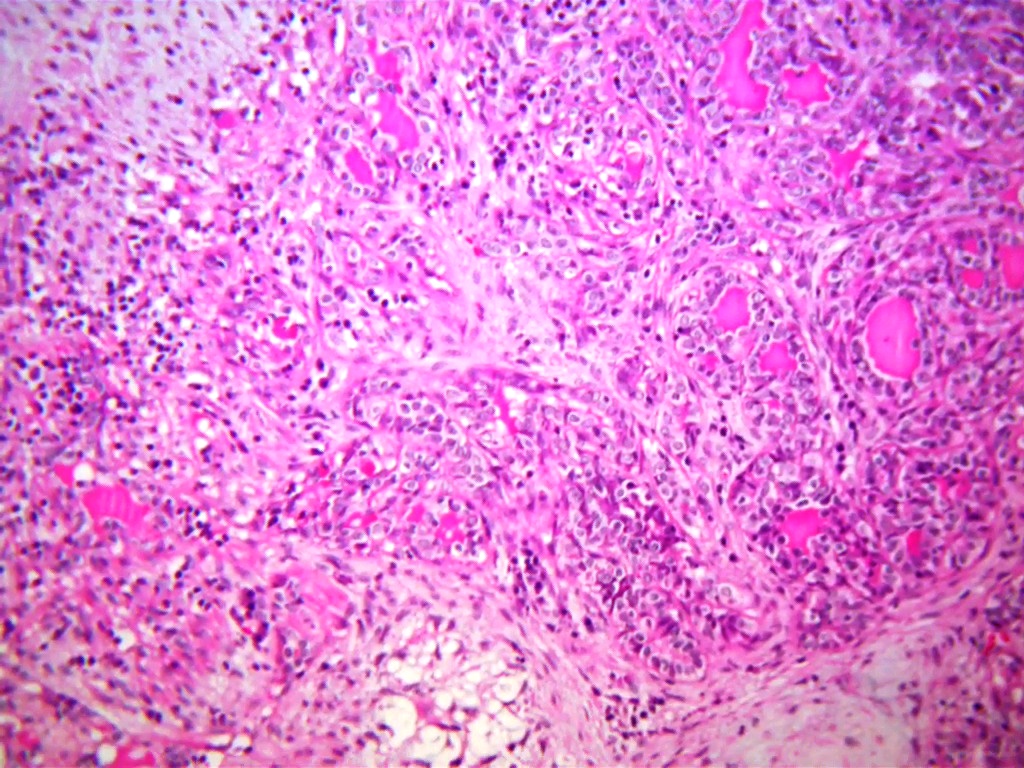

Supplement: Supplementary file 1 [file animals-13-01563-s001.zip › supplementary files/File S1 Canine Mammary Tumor Dataset/benign/Benign mixed tumor_FRM_001 (13).jpg]

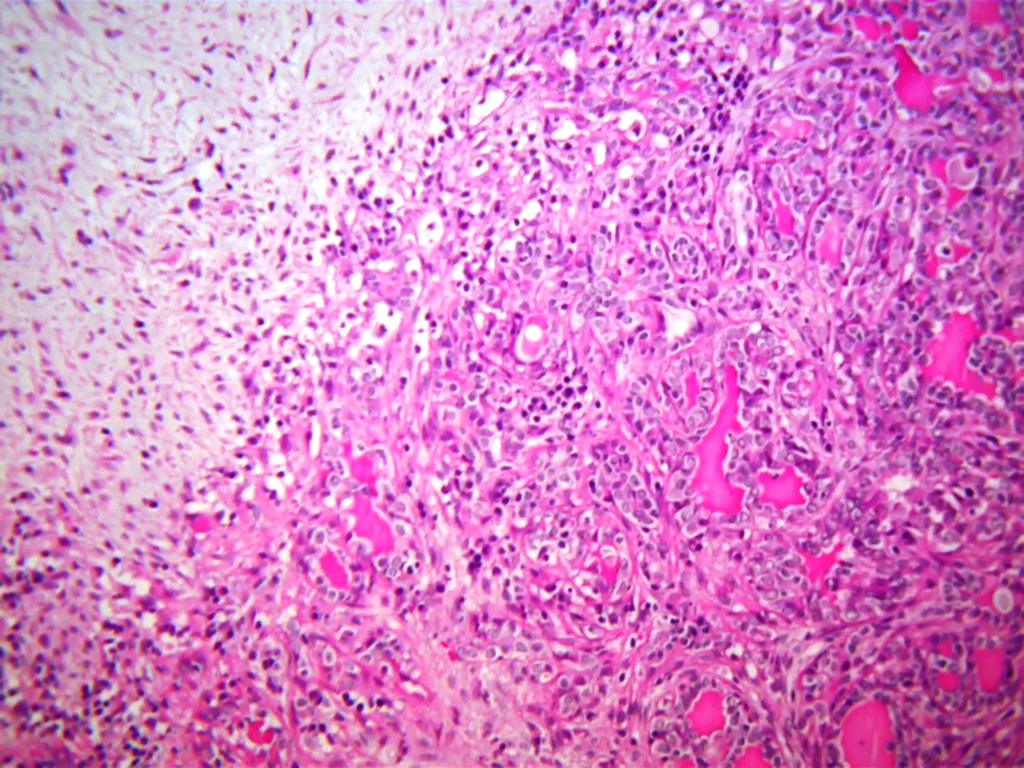

Supplement: Supplementary file 1 [file animals-13-01563-s001.zip › supplementary files/File S1 Canine Mammary Tumor Dataset/benign/Benign mixed tumor_FRM_001 (14).jpg]

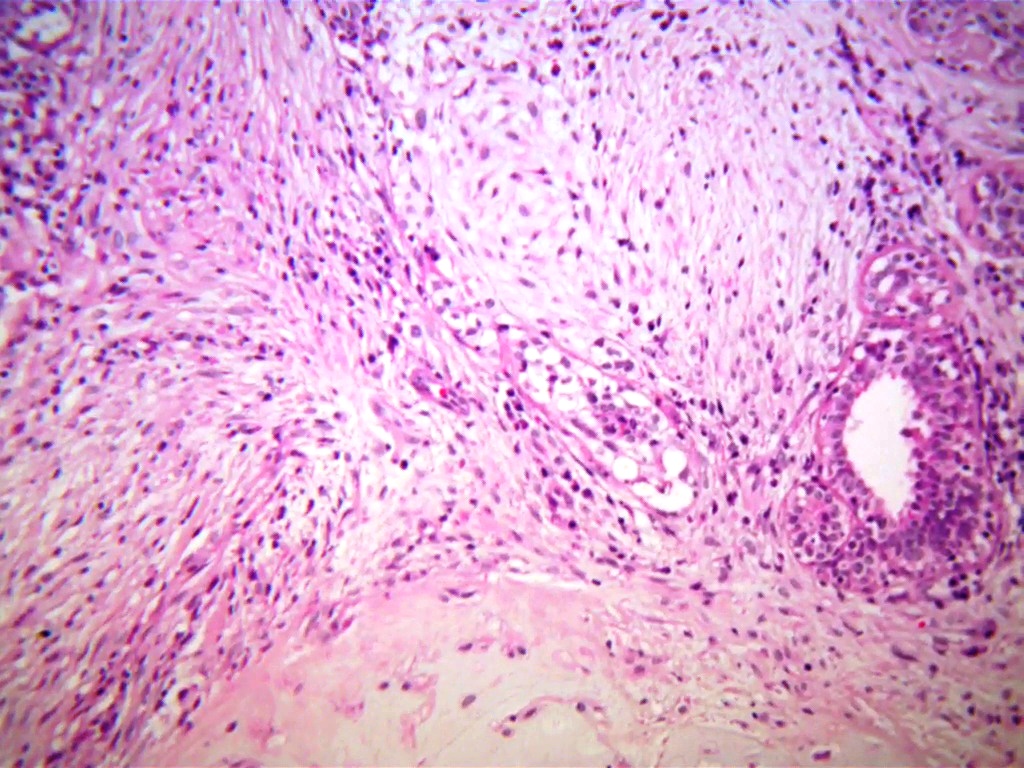

Supplement: Supplementary file 1 [file animals-13-01563-s001.zip › supplementary files/File S1 Canine Mammary Tumor Dataset/benign/Benign mixed tumor_FRM_001 (15).jpg]

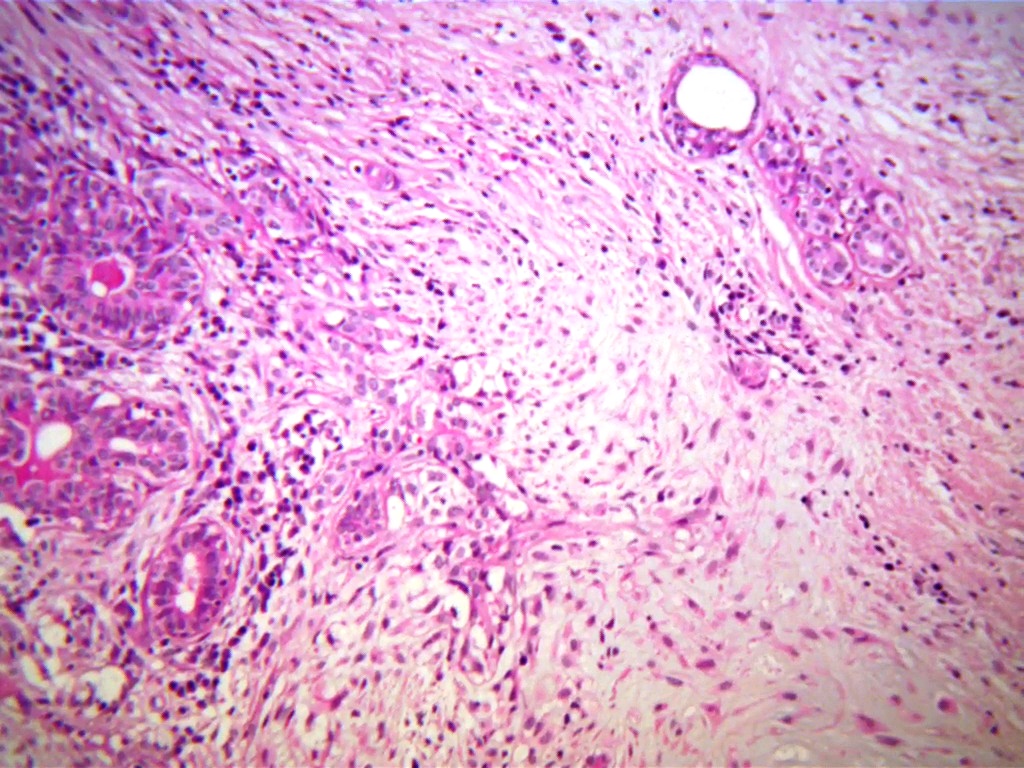

Supplement: Supplementary file 1 [file animals-13-01563-s001.zip › supplementary files/File S1 Canine Mammary Tumor Dataset/benign/Benign mixed tumor_FRM_001 (16).jpg]

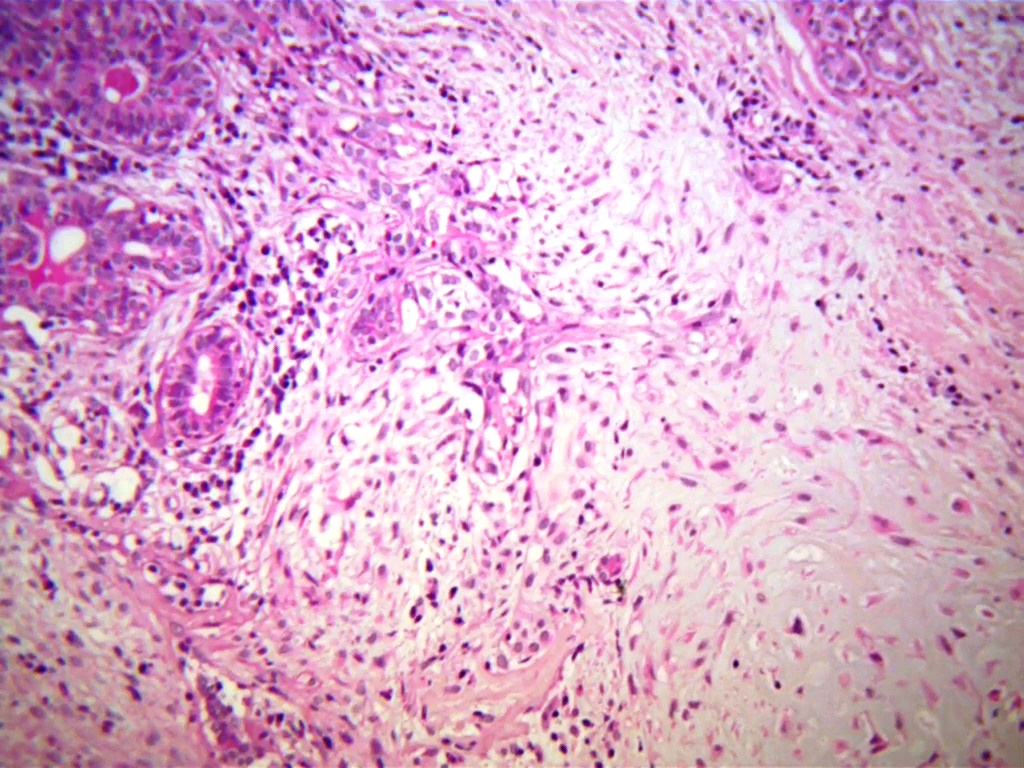

Supplement: Supplementary file 1 [file animals-13-01563-s001.zip › supplementary files/File S1 Canine Mammary Tumor Dataset/benign/Benign mixed tumor_FRM_001 (17).jpg]

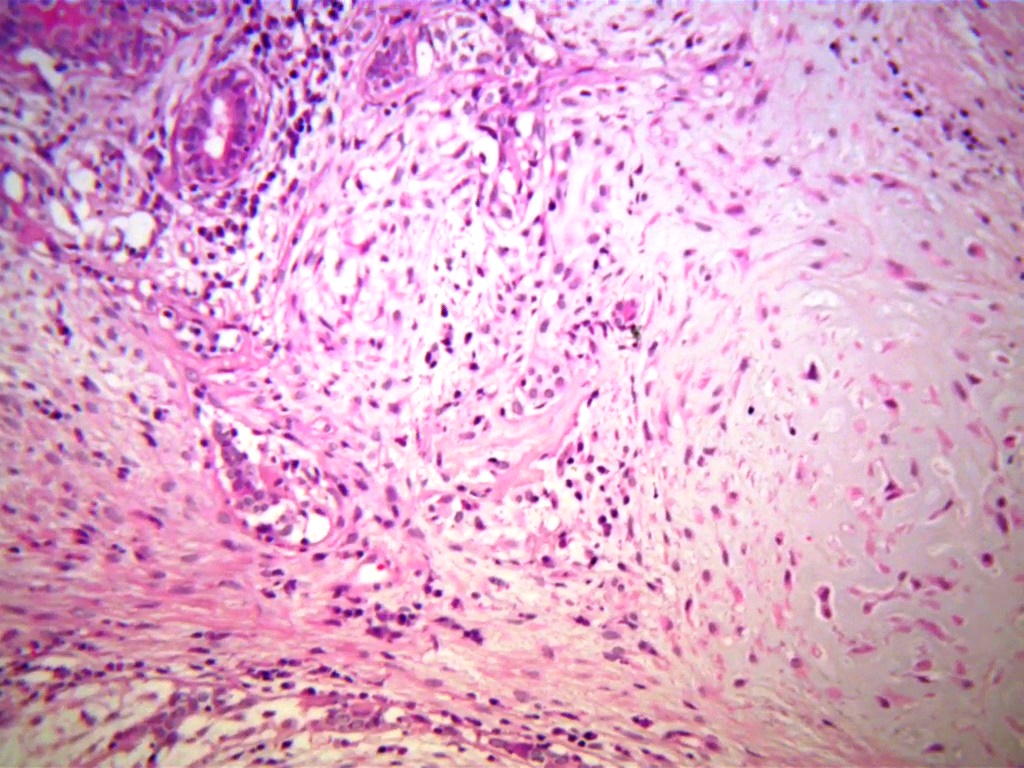

Supplement: Supplementary file 1 [file animals-13-01563-s001.zip › supplementary files/File S1 Canine Mammary Tumor Dataset/benign/Benign mixed tumor_FRM_001 (18).jpg]

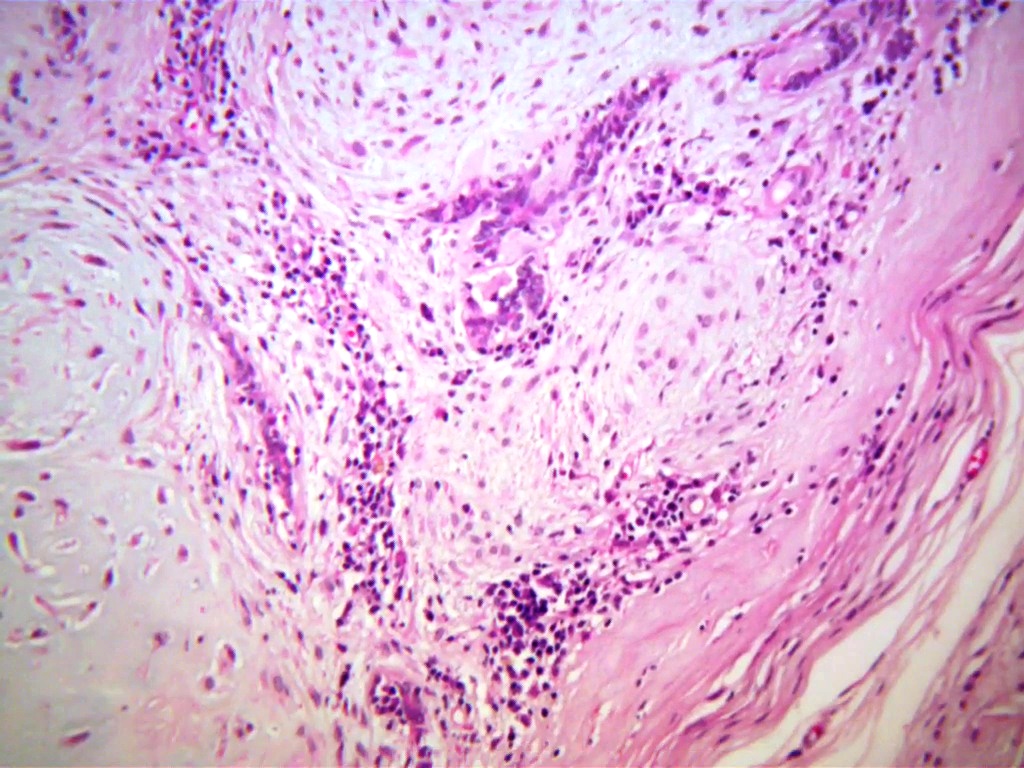

Supplement: Supplementary file 1 [file animals-13-01563-s001.zip › supplementary files/File S1 Canine Mammary Tumor Dataset/benign/Benign mixed tumor_FRM_001 (19).jpg]

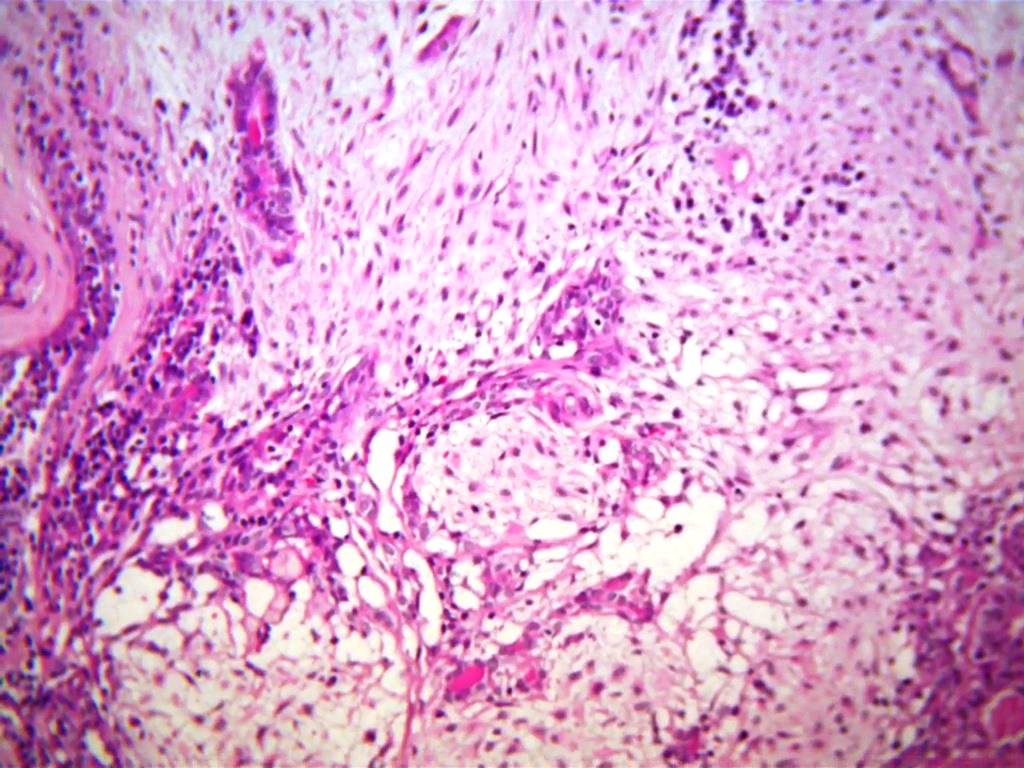

Supplement: Supplementary file 1 [file animals-13-01563-s001.zip › supplementary files/File S1 Canine Mammary Tumor Dataset/benign/Benign mixed tumor_FRM_001 (2).jpg]

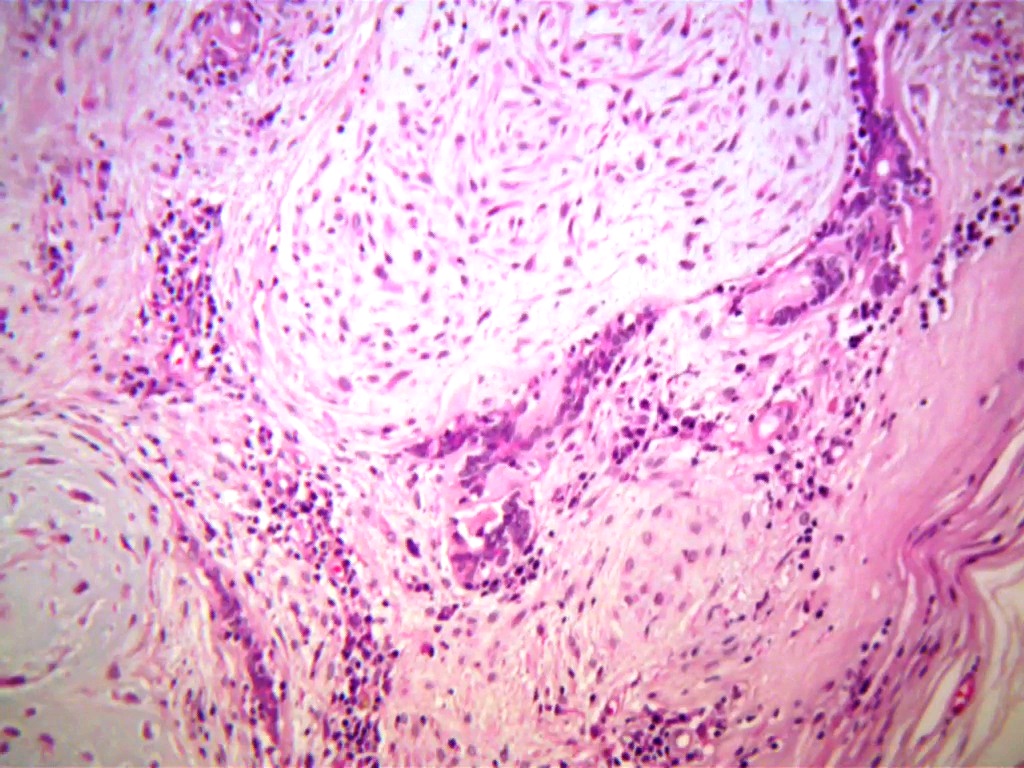

Supplement: Supplementary file 1 [file animals-13-01563-s001.zip › supplementary files/File S1 Canine Mammary Tumor Dataset/benign/Benign mixed tumor_FRM_001 (20).jpg]

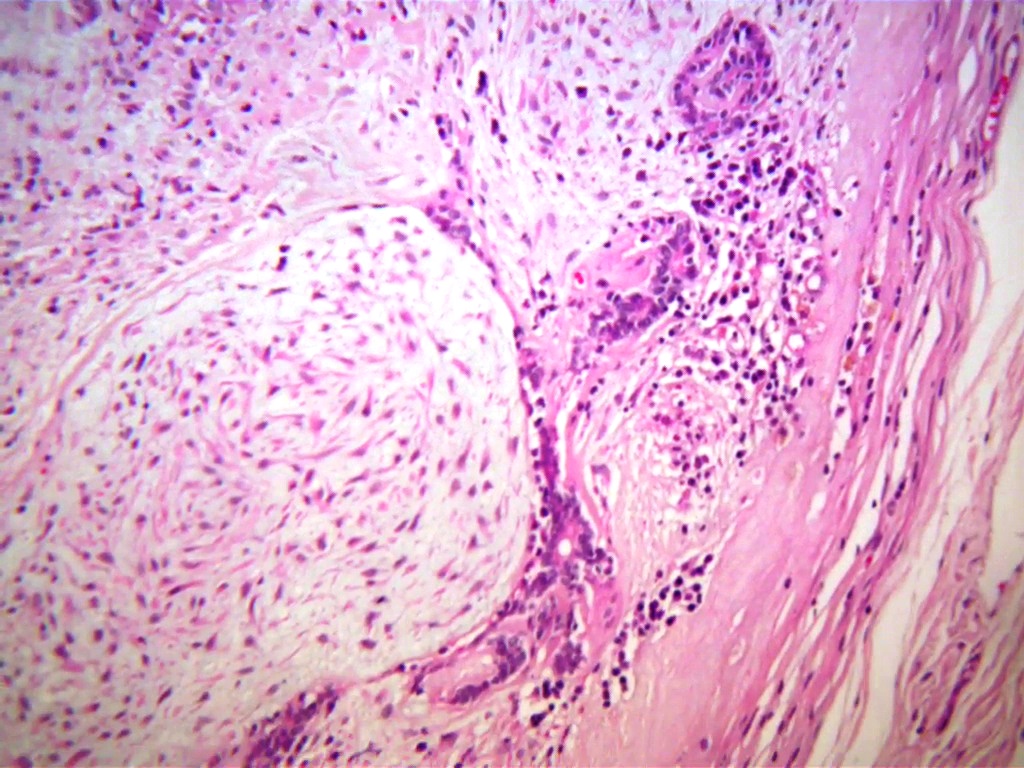

Supplement: Supplementary file 1 [file animals-13-01563-s001.zip › supplementary files/File S1 Canine Mammary Tumor Dataset/benign/Benign mixed tumor_FRM_001 (21).jpg]

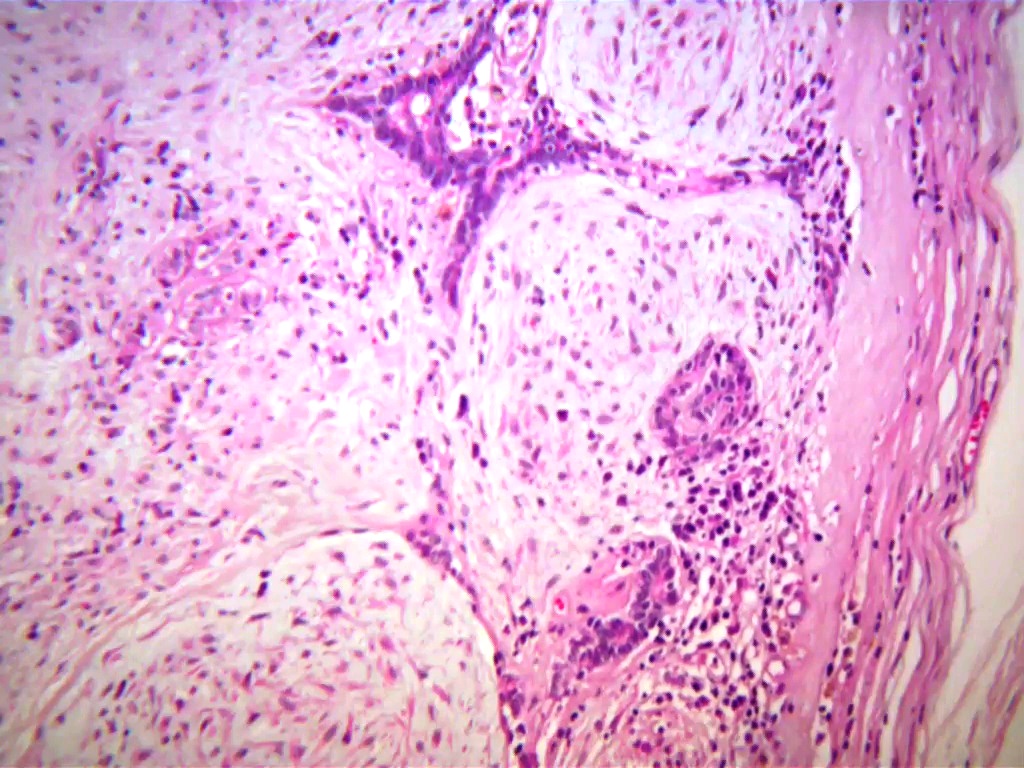

Supplement: Supplementary file 1 [file animals-13-01563-s001.zip › supplementary files/File S1 Canine Mammary Tumor Dataset/benign/Benign mixed tumor_FRM_001 (22).jpg]

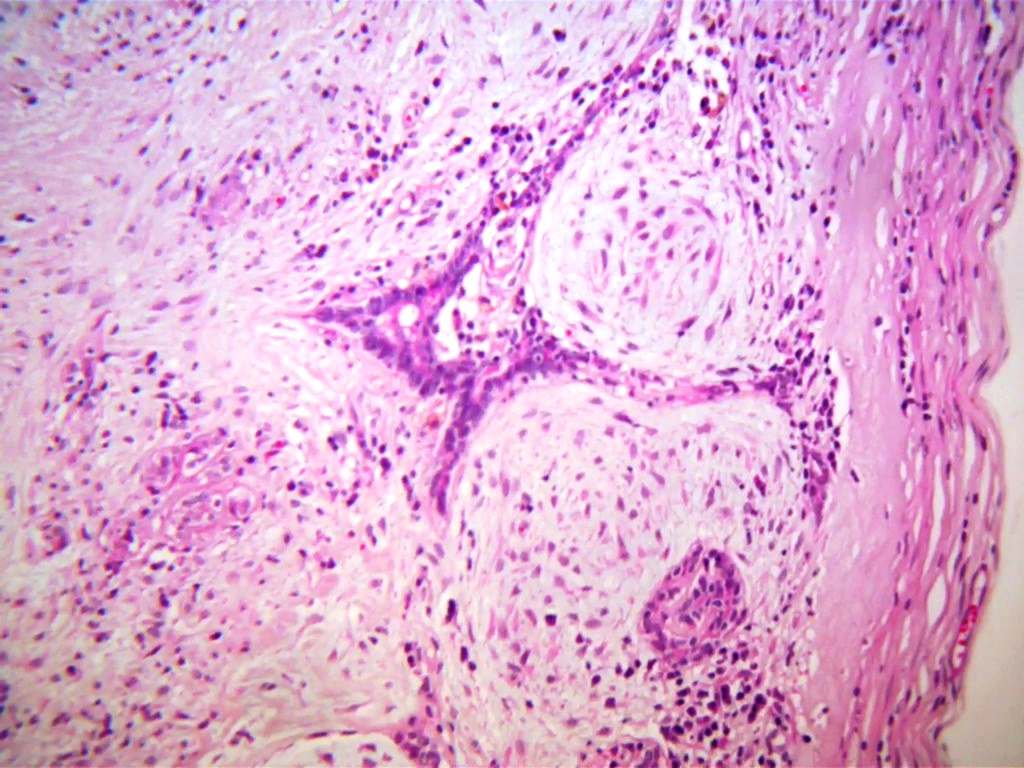

Supplement: Supplementary file 1 [file animals-13-01563-s001.zip › supplementary files/File S1 Canine Mammary Tumor Dataset/benign/Benign mixed tumor_FRM_001 (23).jpg]

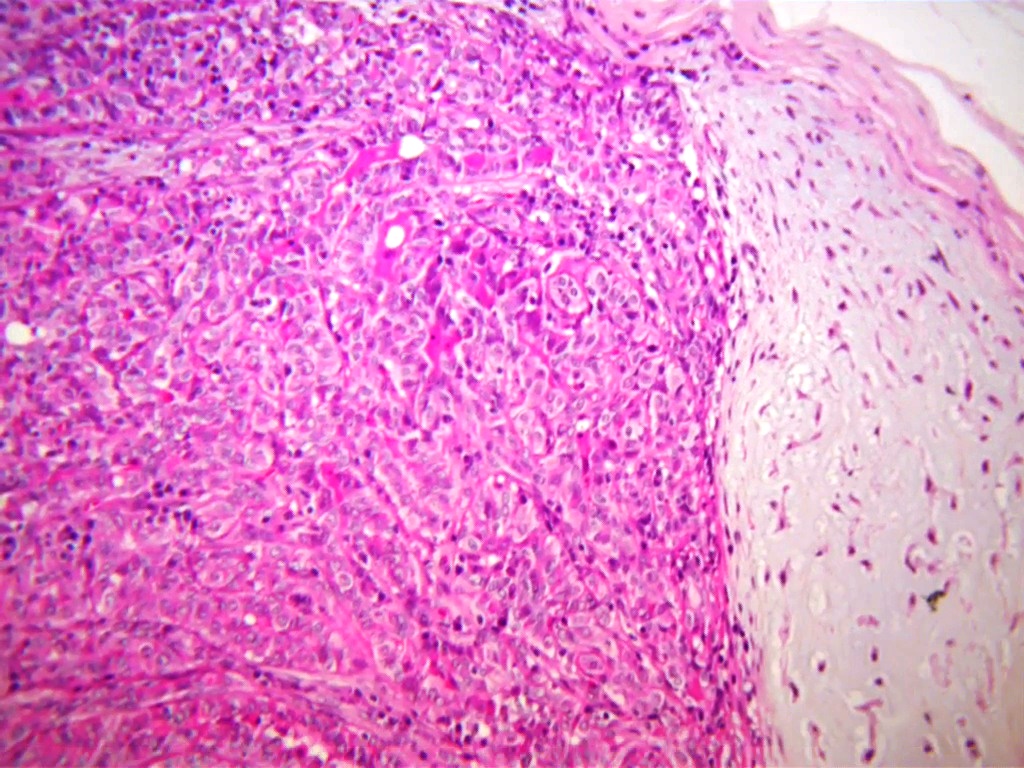

Supplement: Supplementary file 1 [file animals-13-01563-s001.zip › supplementary files/File S1 Canine Mammary Tumor Dataset/benign/Benign mixed tumor_FRM_001 (24).jpg]

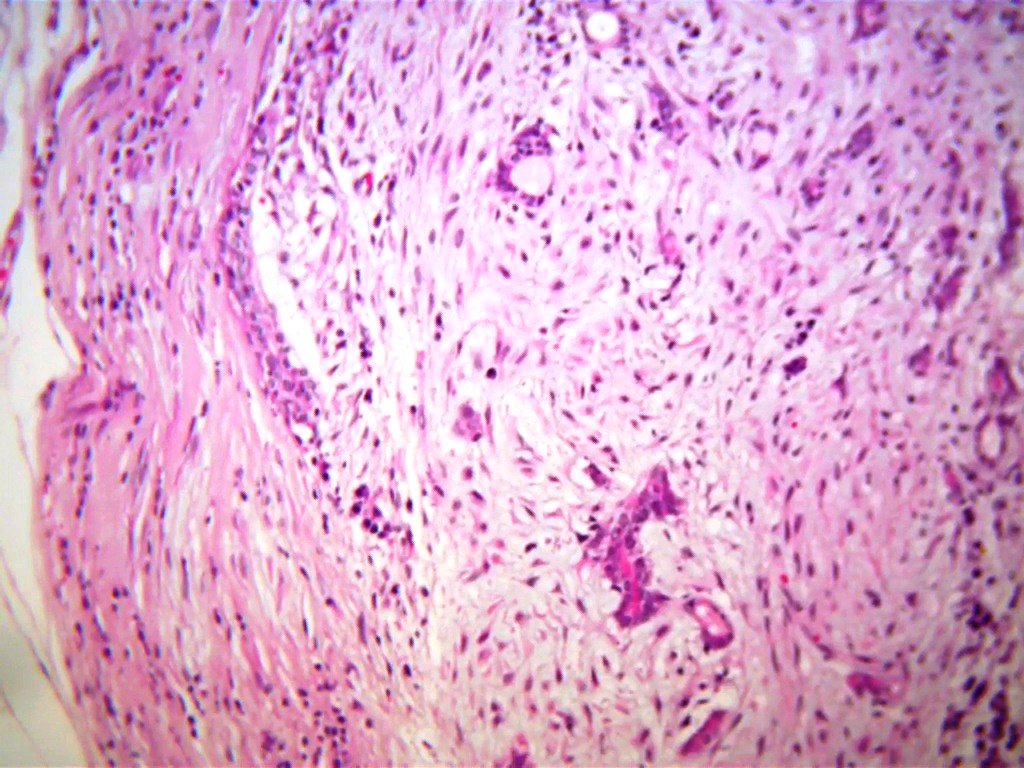

Supplement: Supplementary file 1 [file animals-13-01563-s001.zip › supplementary files/File S1 Canine Mammary Tumor Dataset/benign/Benign mixed tumor_FRM_001 (3).jpg]

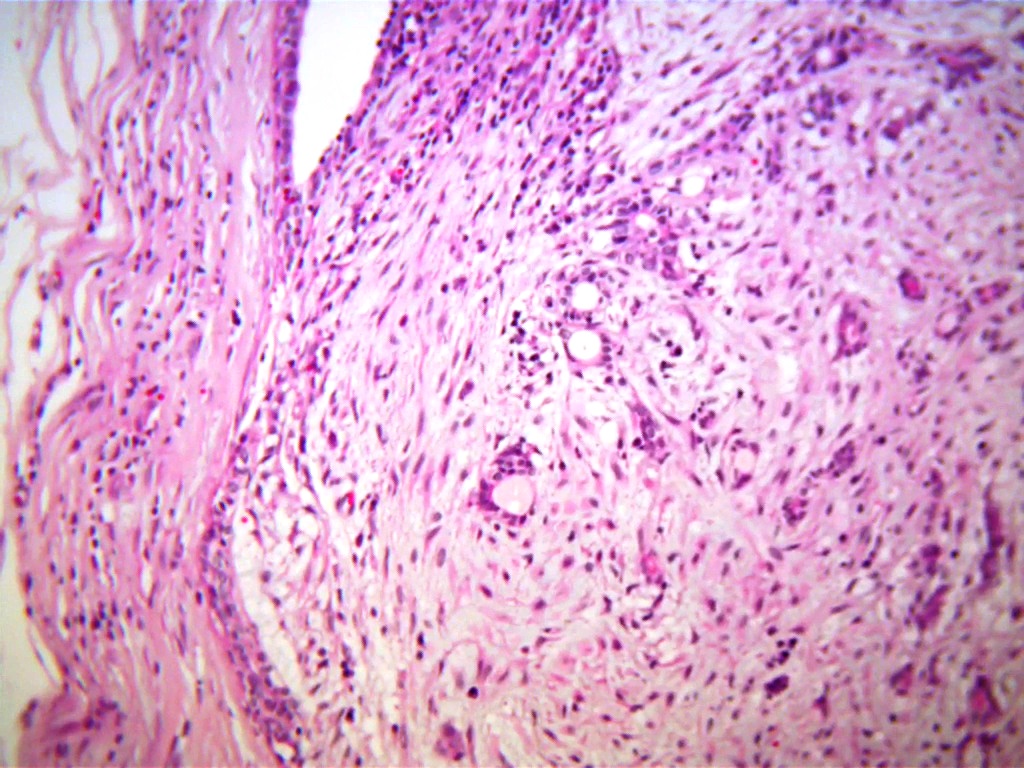

Supplement: Supplementary file 1 [file animals-13-01563-s001.zip › supplementary files/File S1 Canine Mammary Tumor Dataset/benign/Benign mixed tumor_FRM_001 (4).jpg]

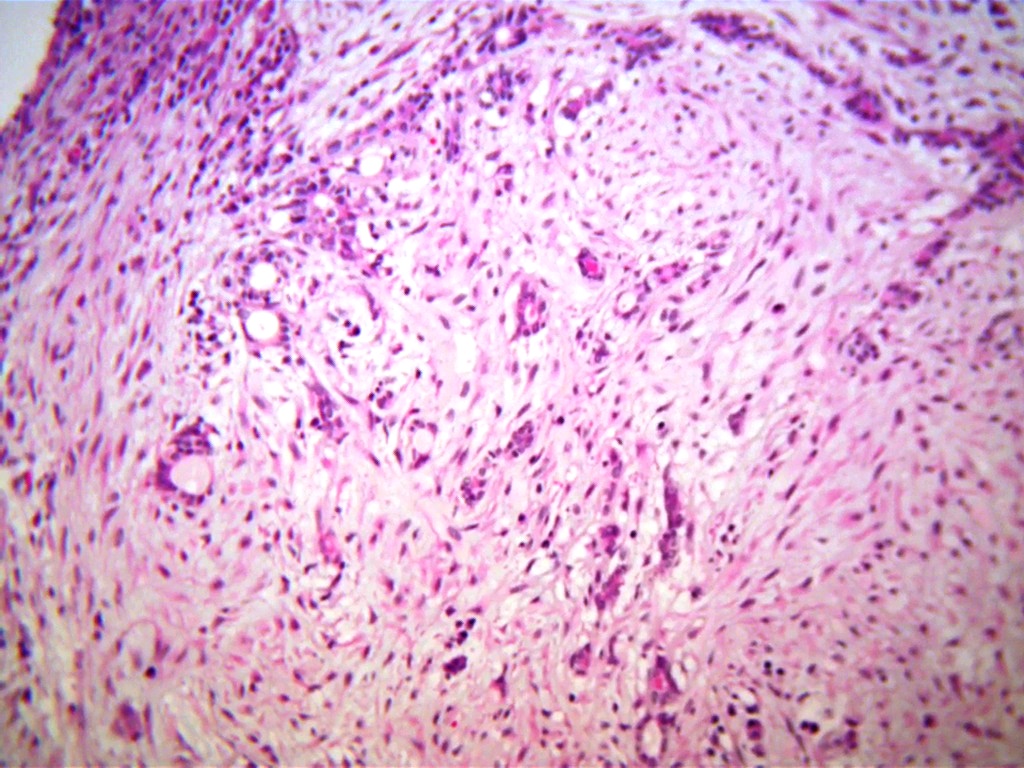

Supplement: Supplementary file 1 [file animals-13-01563-s001.zip › supplementary files/File S1 Canine Mammary Tumor Dataset/benign/Benign mixed tumor_FRM_001 (5).jpg]

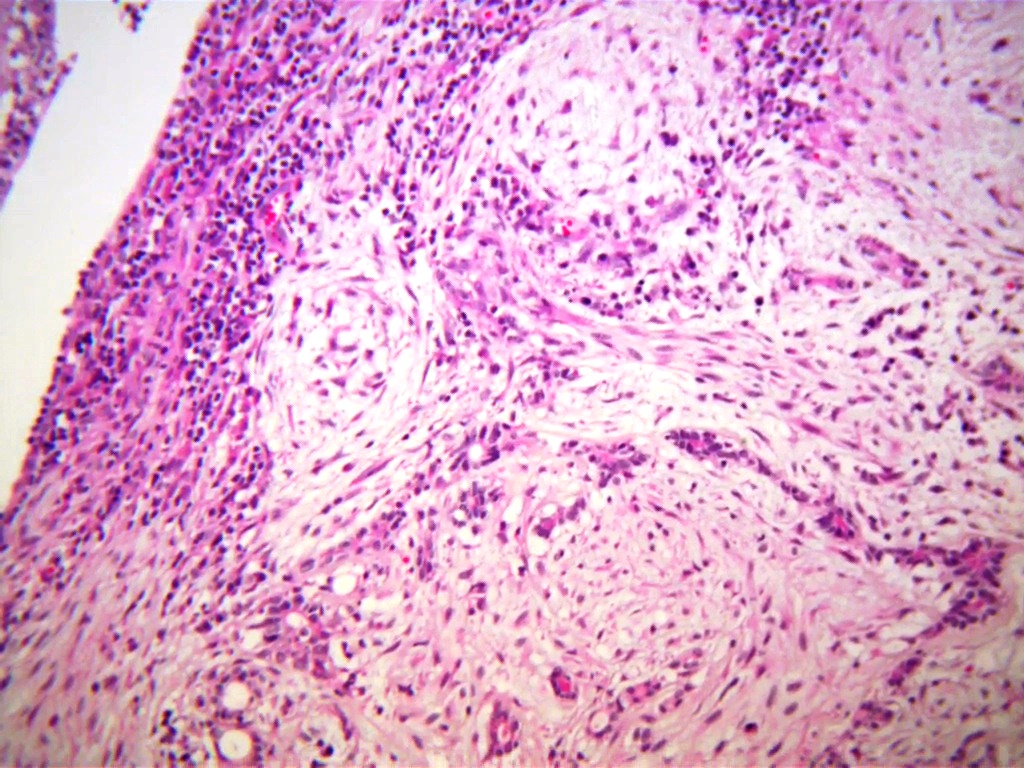

Supplement: Supplementary file 1 [file animals-13-01563-s001.zip › supplementary files/File S1 Canine Mammary Tumor Dataset/benign/Benign mixed tumor_FRM_001 (6).jpg]

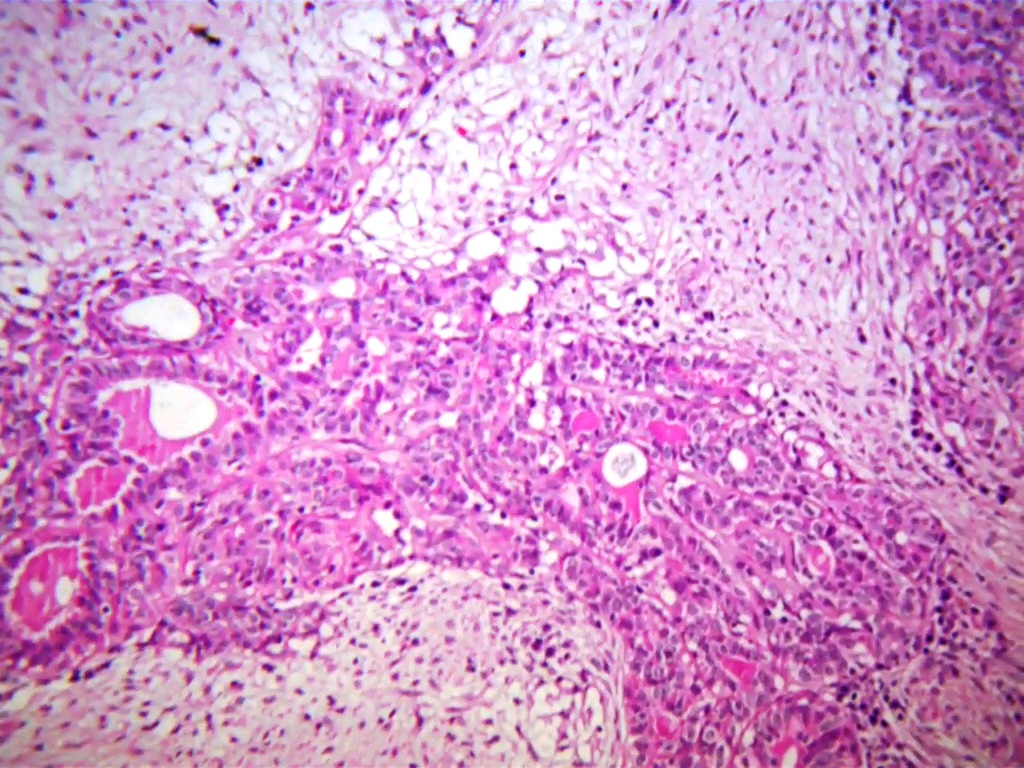

Supplement: Supplementary file 1 [file animals-13-01563-s001.zip › supplementary files/File S1 Canine Mammary Tumor Dataset/benign/Benign mixed tumor_FRM_001 (7).jpg]

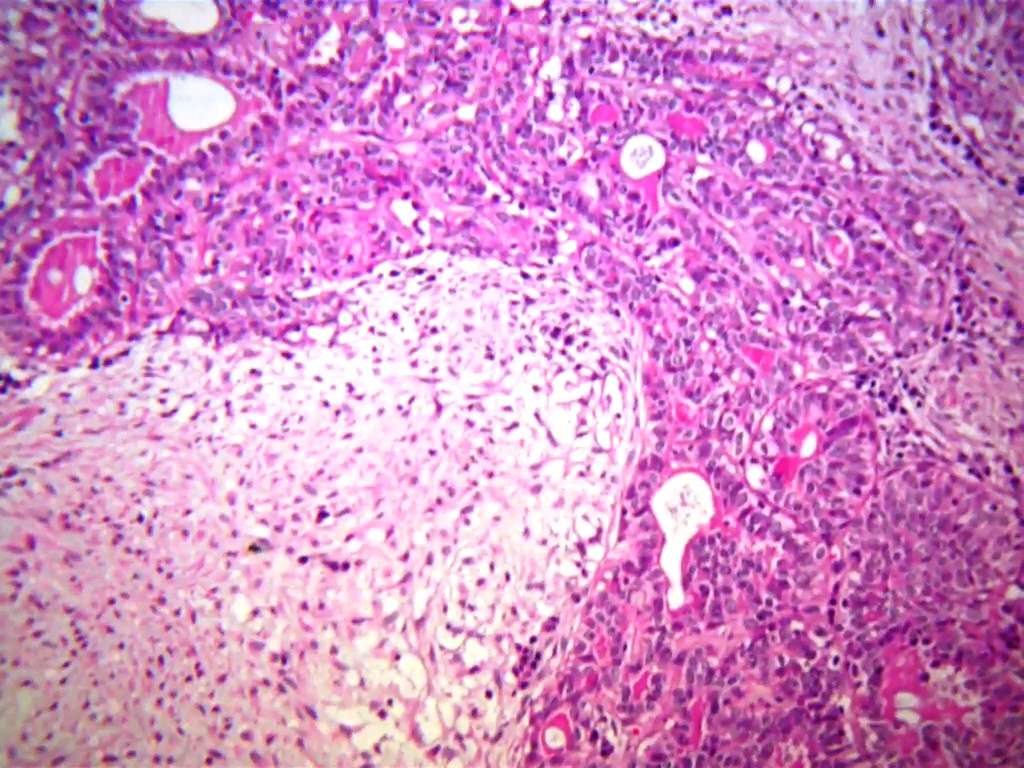

Supplement: Supplementary file 1 [file animals-13-01563-s001.zip › supplementary files/File S1 Canine Mammary Tumor Dataset/benign/Benign mixed tumor_FRM_001 (8).jpg]

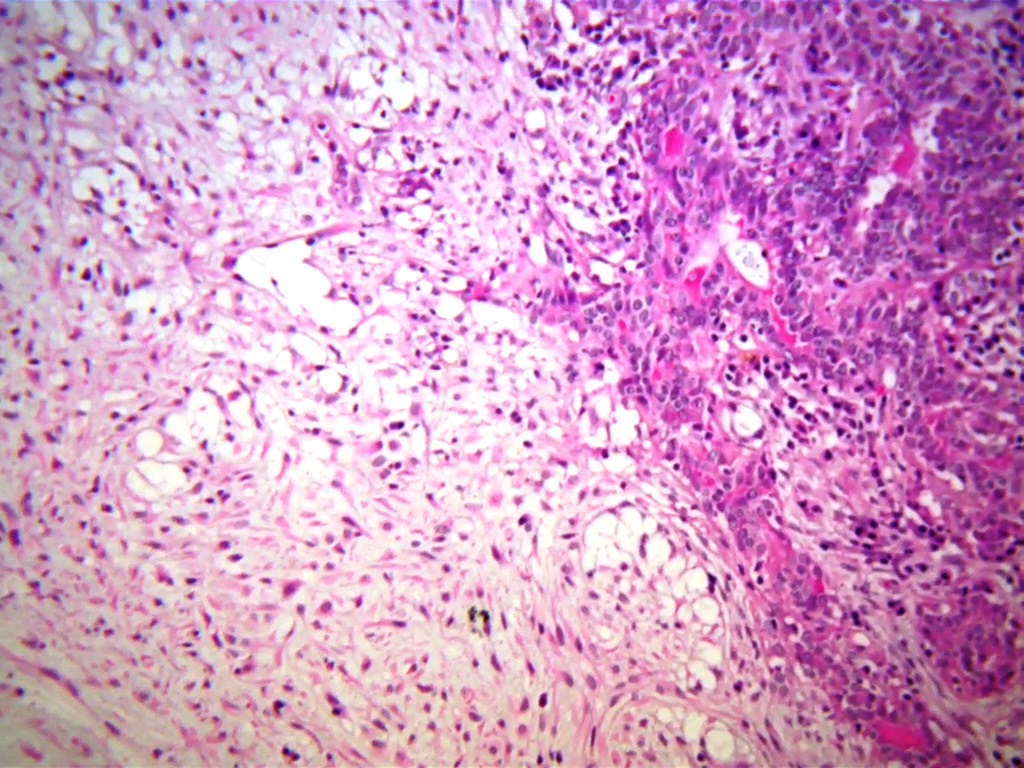

Supplement: Supplementary file 1 [file animals-13-01563-s001.zip › supplementary files/File S1 Canine Mammary Tumor Dataset/benign/Benign mixed tumor_FRM_001 (9).jpg]

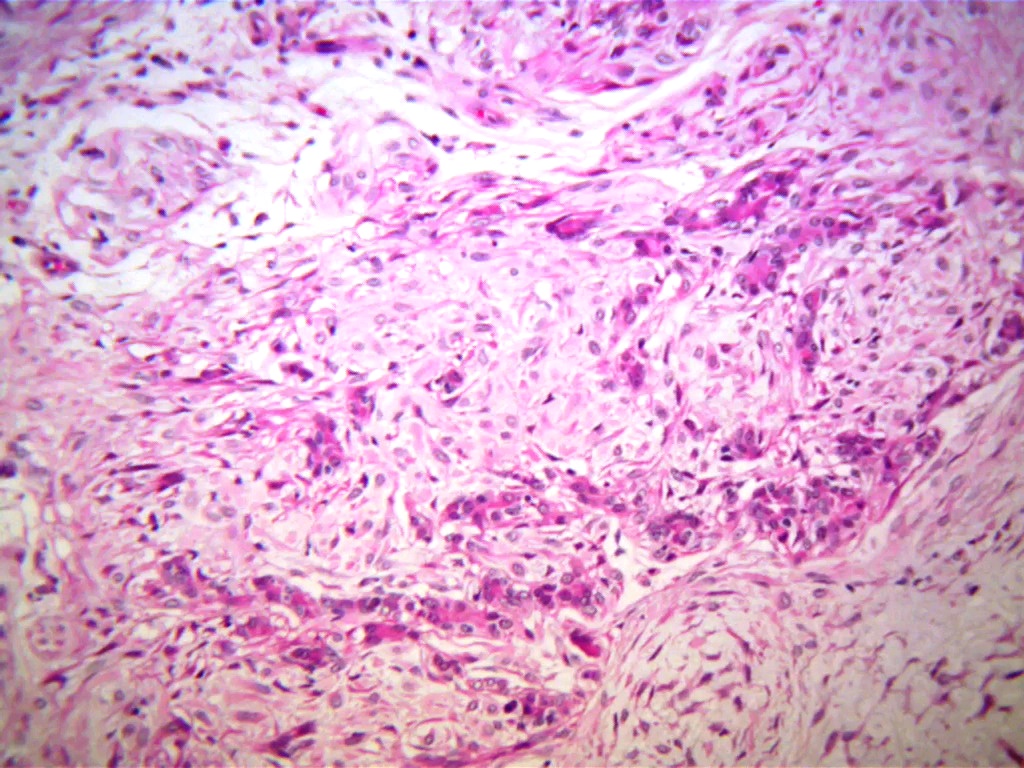

Supplement: Supplementary file 1 [file animals-13-01563-s001.zip › supplementary files/File S1 Canine Mammary Tumor Dataset/benign/Benign mixed tumor-466-11_FRM_008 (1).jpg]

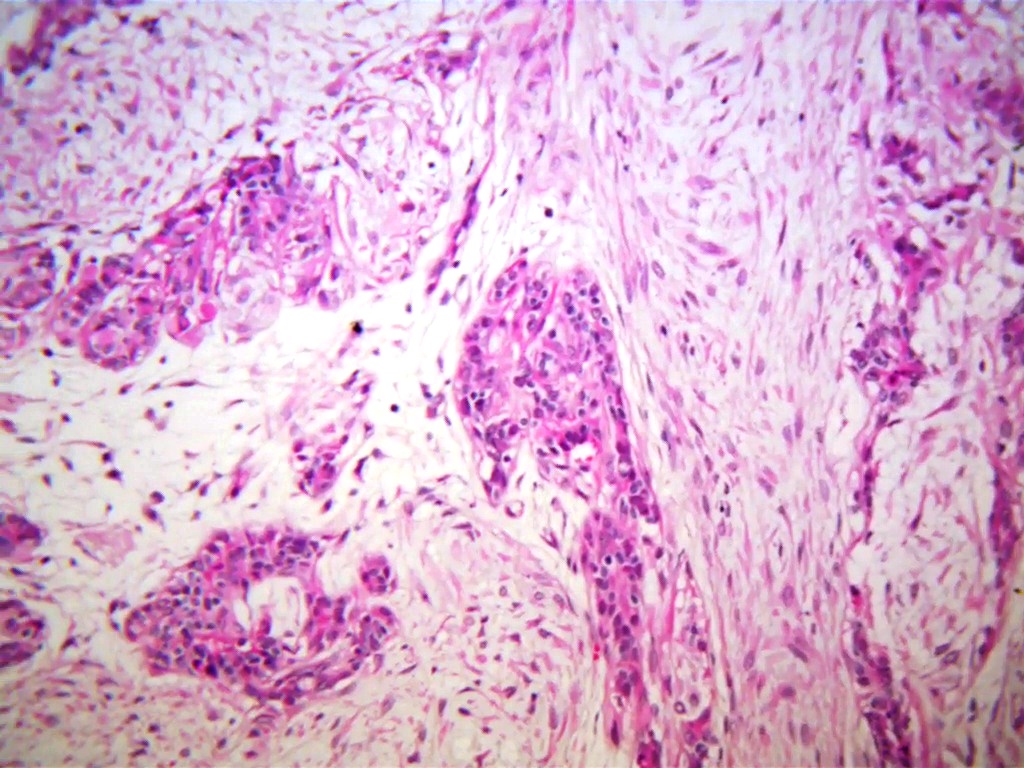

Supplement: Supplementary file 1 [file animals-13-01563-s001.zip › supplementary files/File S1 Canine Mammary Tumor Dataset/benign/Benign mixed tumor-466-11_FRM_008 (10).jpg]

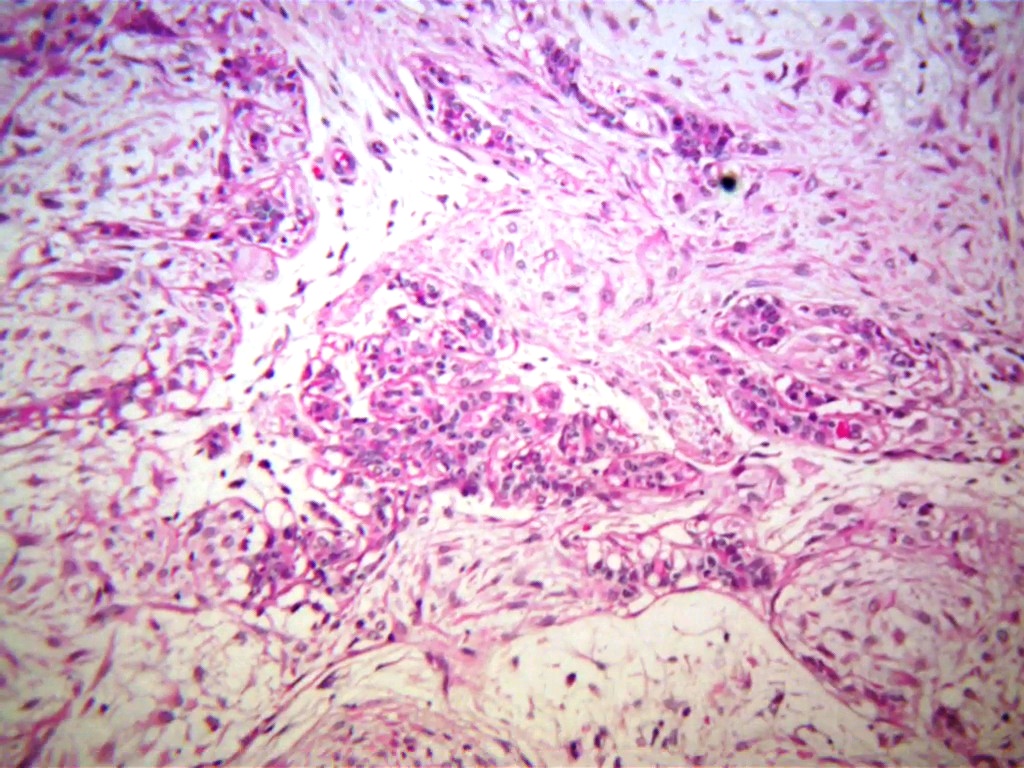

Supplement: Supplementary file 1 [file animals-13-01563-s001.zip › supplementary files/File S1 Canine Mammary Tumor Dataset/benign/Benign mixed tumor-466-11_FRM_008 (11).jpg]

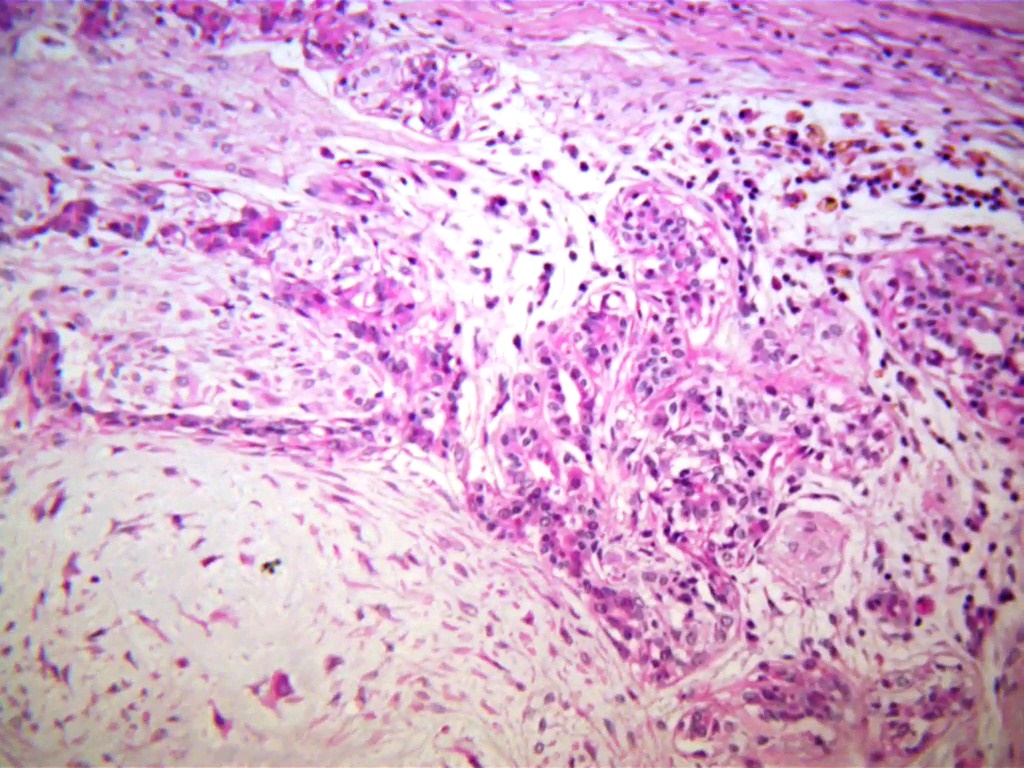

Supplement: Supplementary file 1 [file animals-13-01563-s001.zip › supplementary files/File S1 Canine Mammary Tumor Dataset/benign/Benign mixed tumor-466-11_FRM_008 (12).jpg]

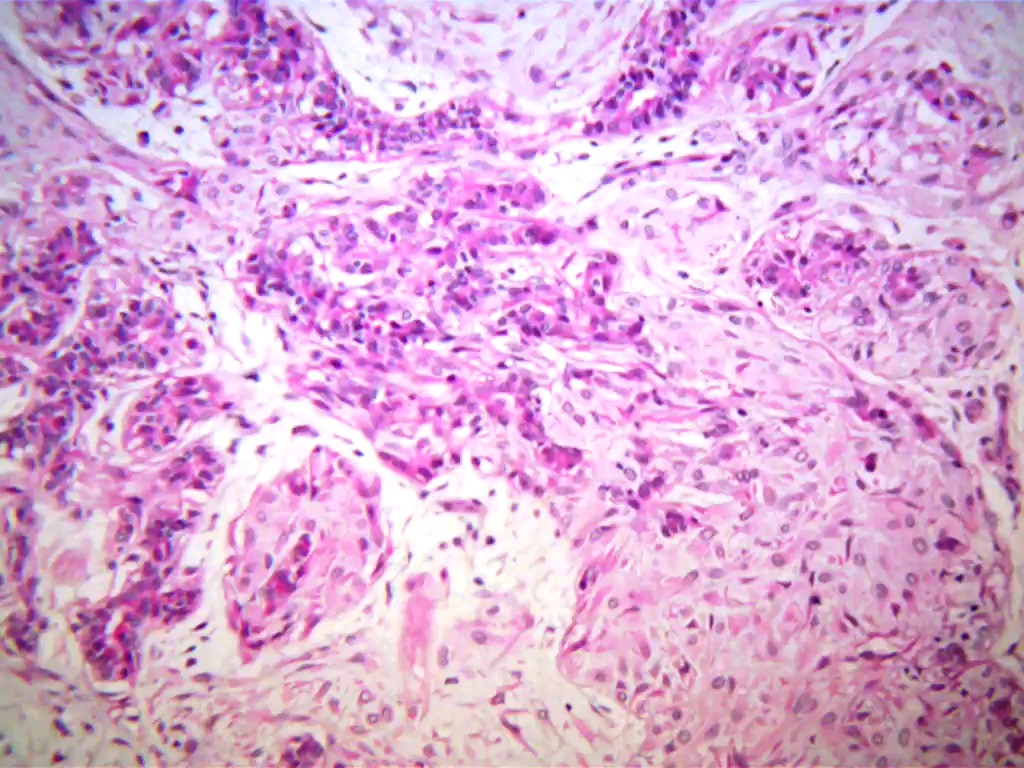

Supplement: Supplementary file 1 [file animals-13-01563-s001.zip › supplementary files/File S1 Canine Mammary Tumor Dataset/benign/Benign mixed tumor-466-11_FRM_008 (13).jpg]

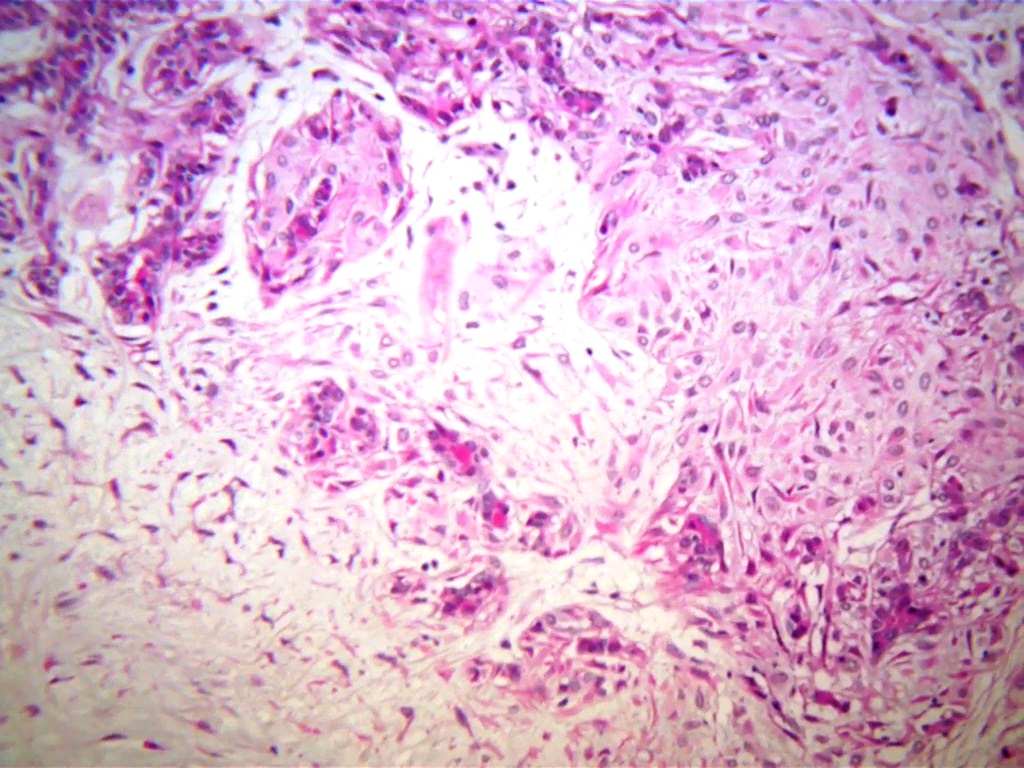

Supplement: Supplementary file 1 [file animals-13-01563-s001.zip › supplementary files/File S1 Canine Mammary Tumor Dataset/benign/Benign mixed tumor-466-11_FRM_008 (14).jpg]

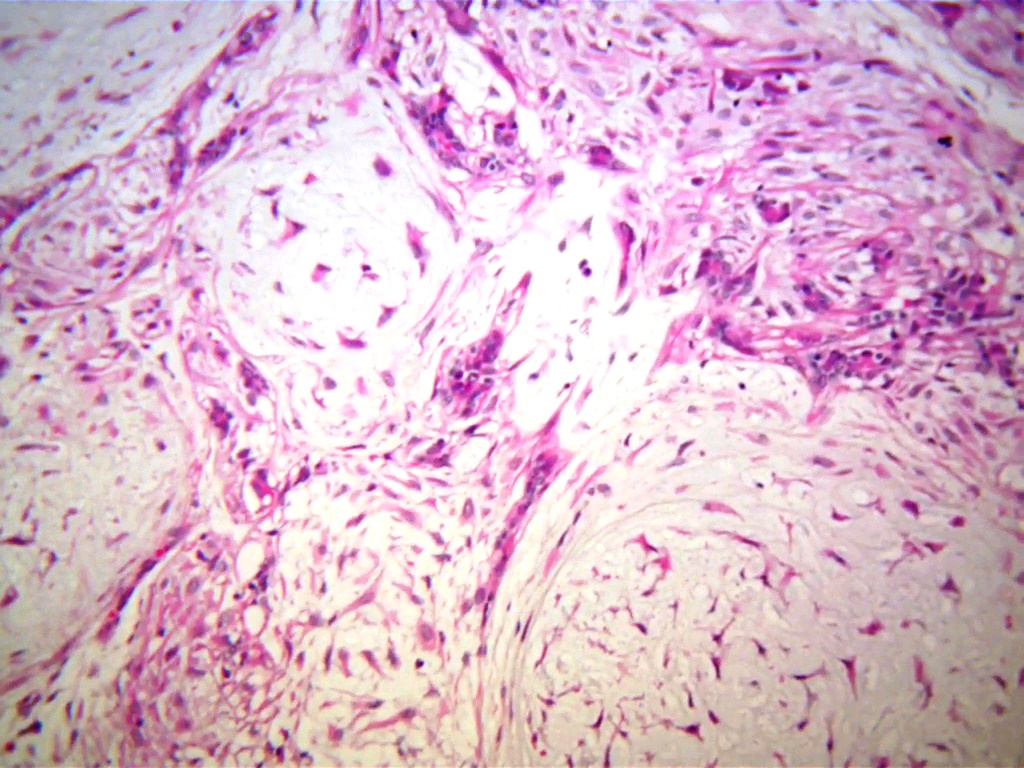

Supplement: Supplementary file 1 [file animals-13-01563-s001.zip › supplementary files/File S1 Canine Mammary Tumor Dataset/benign/Benign mixed tumor-466-11_FRM_008 (15).jpg]

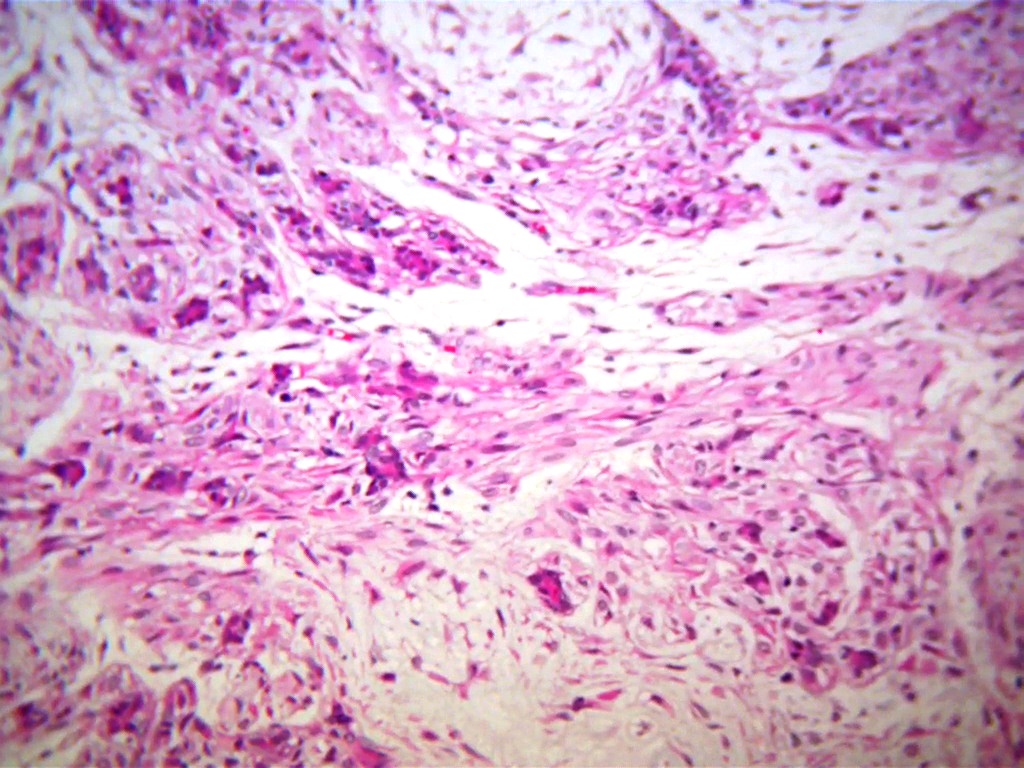

Supplement: Supplementary file 1 [file animals-13-01563-s001.zip › supplementary files/File S1 Canine Mammary Tumor Dataset/benign/Benign mixed tumor-466-11_FRM_008 (16).jpg]

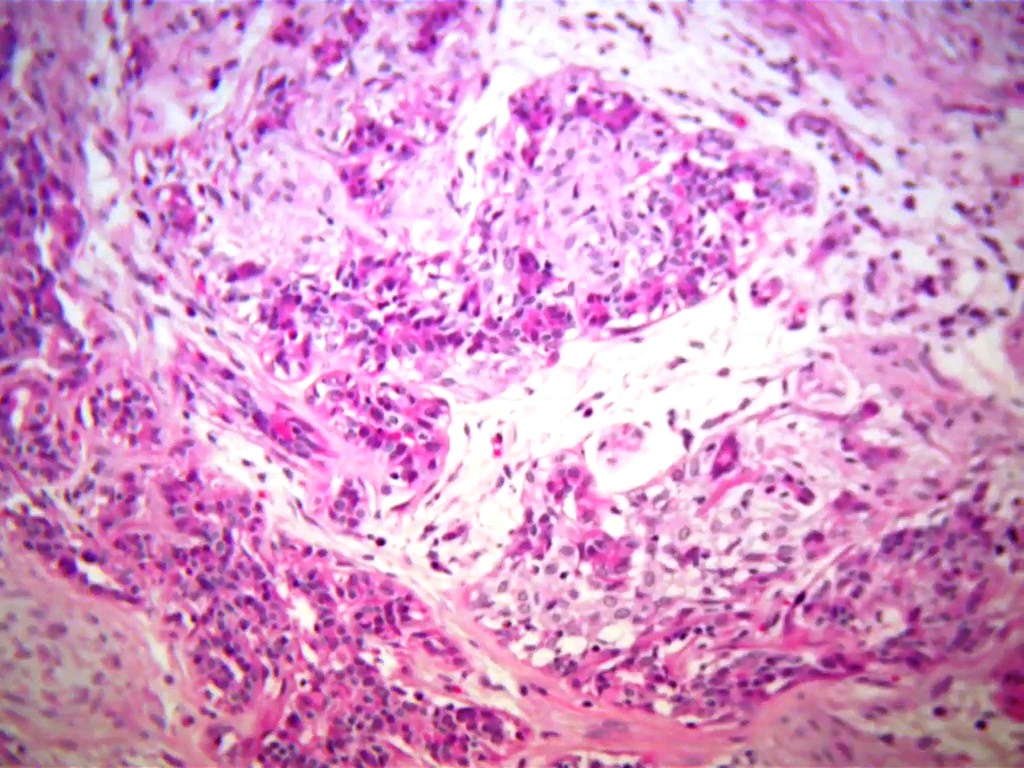

Supplement: Supplementary file 1 [file animals-13-01563-s001.zip › supplementary files/File S1 Canine Mammary Tumor Dataset/benign/Benign mixed tumor-466-11_FRM_008 (17).jpg]

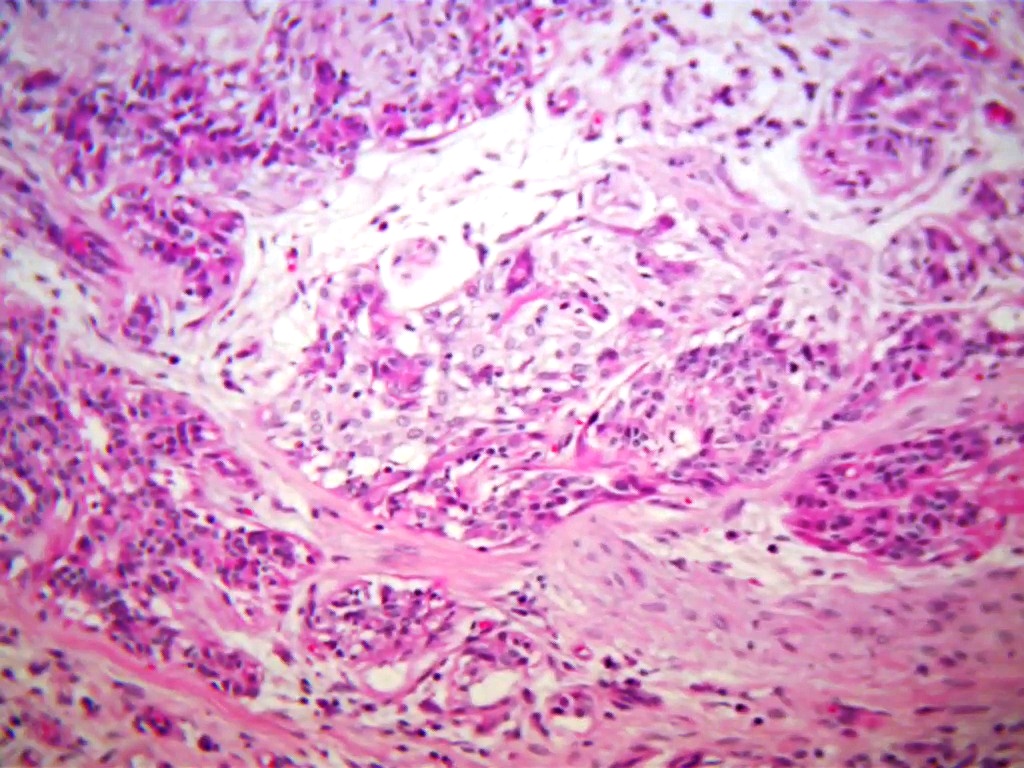

Supplement: Supplementary file 1 [file animals-13-01563-s001.zip › supplementary files/File S1 Canine Mammary Tumor Dataset/benign/Benign mixed tumor-466-11_FRM_008 (18).jpg]

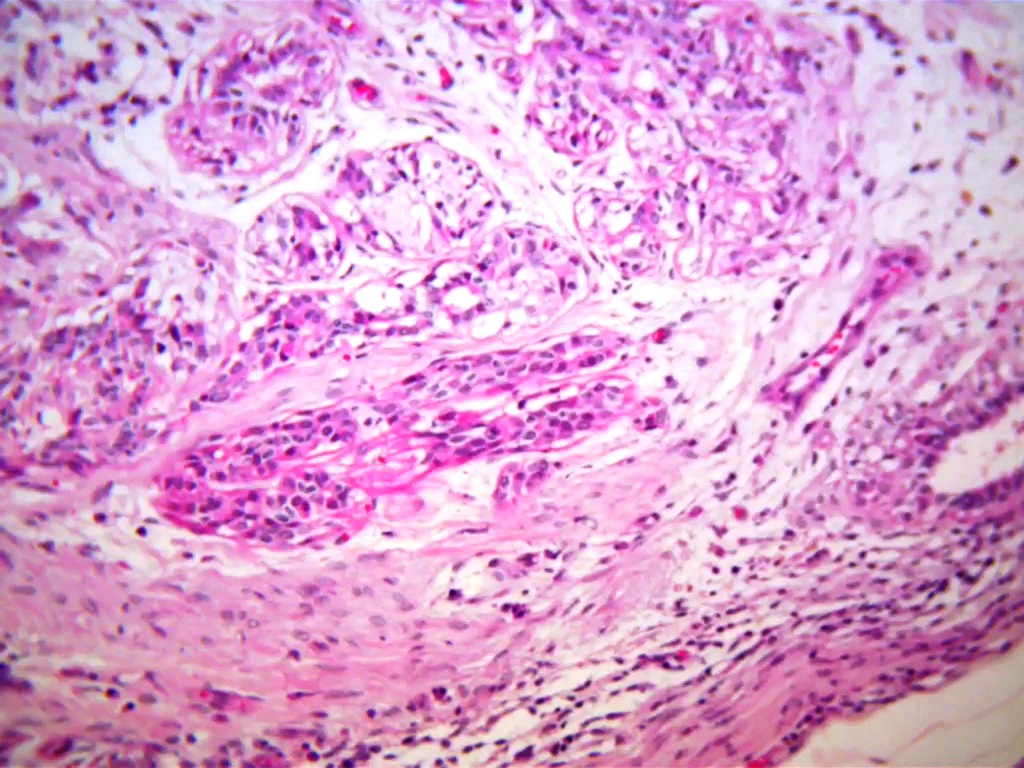

Supplement: Supplementary file 1 [file animals-13-01563-s001.zip › supplementary files/File S1 Canine Mammary Tumor Dataset/benign/Benign mixed tumor-466-11_FRM_008 (19).jpg]

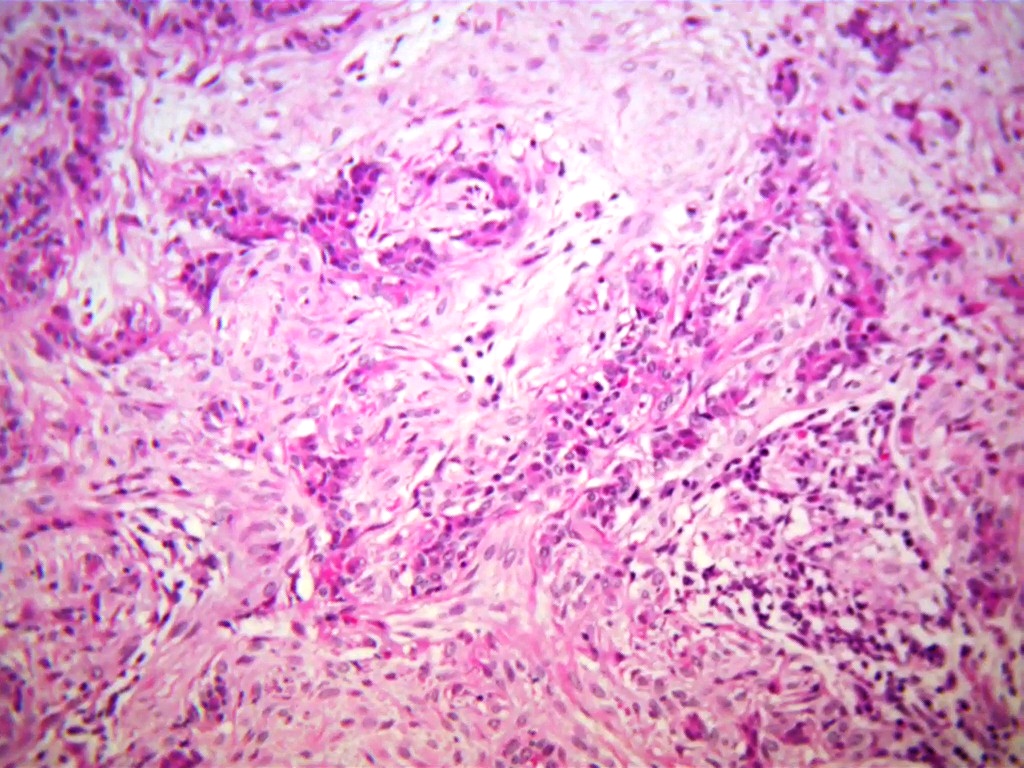

Supplement: Supplementary file 1 [file animals-13-01563-s001.zip › supplementary files/File S1 Canine Mammary Tumor Dataset/benign/Benign mixed tumor-466-11_FRM_008 (2).jpg]

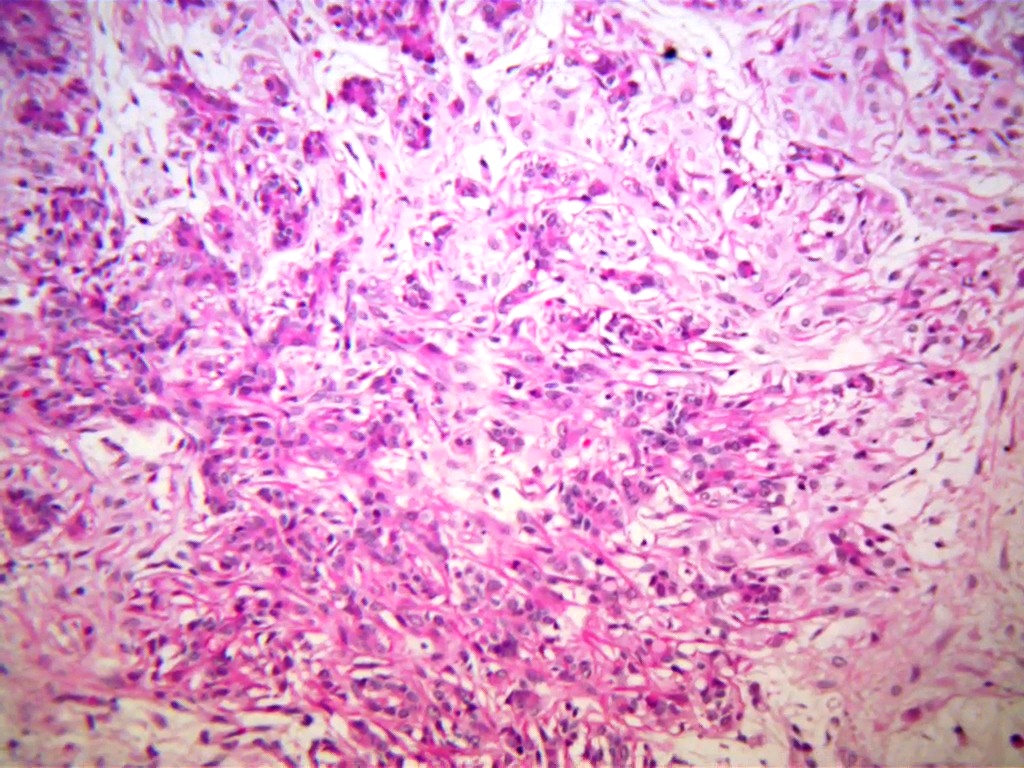

Supplement: Supplementary file 1 [file animals-13-01563-s001.zip › supplementary files/File S1 Canine Mammary Tumor Dataset/benign/Benign mixed tumor-466-11_FRM_008 (20).jpg]

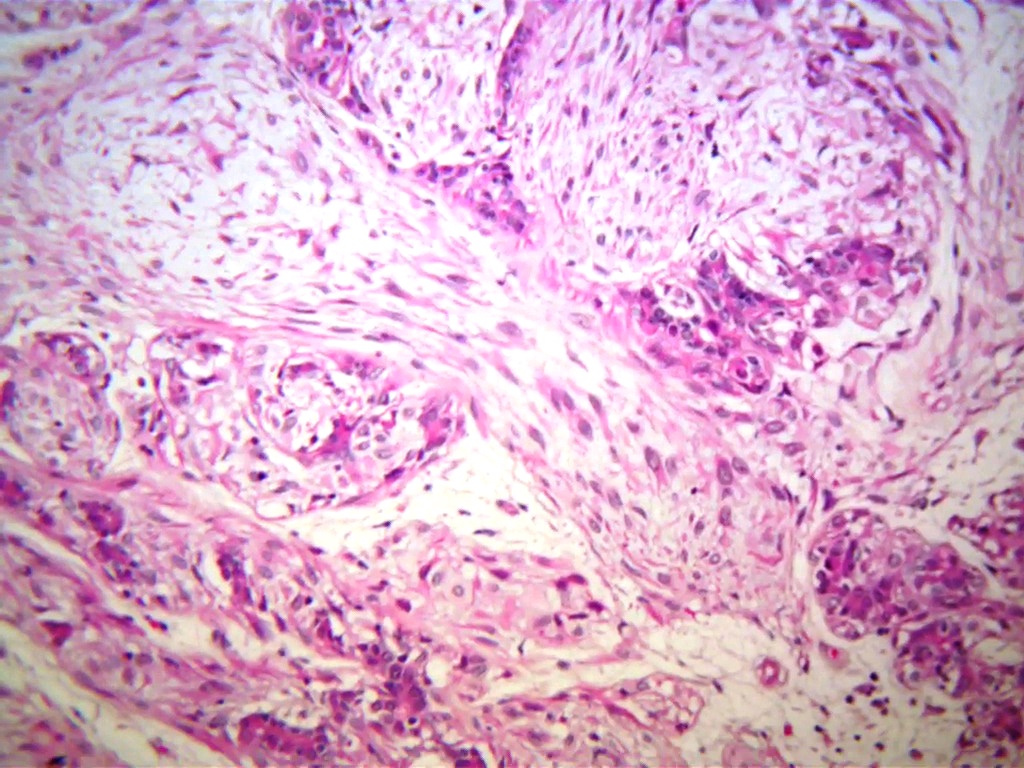

Supplement: Supplementary file 1 [file animals-13-01563-s001.zip › supplementary files/File S1 Canine Mammary Tumor Dataset/benign/Benign mixed tumor-466-11_FRM_008 (21).jpg]

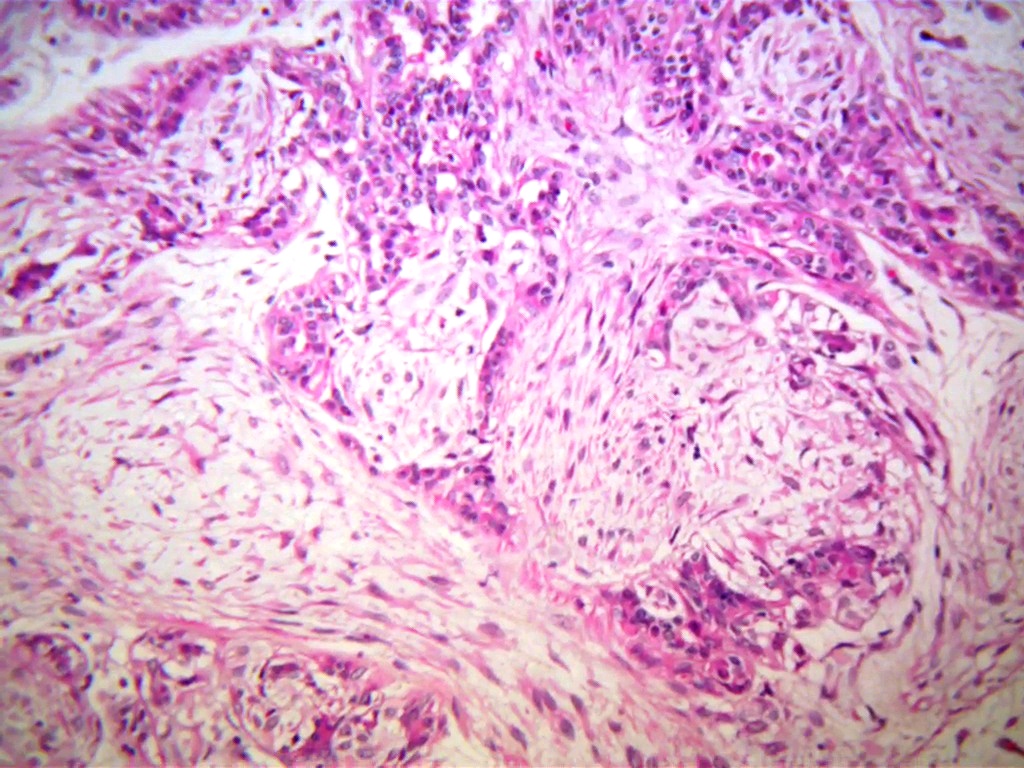

Supplement: Supplementary file 1 [file animals-13-01563-s001.zip › supplementary files/File S1 Canine Mammary Tumor Dataset/benign/Benign mixed tumor-466-11_FRM_008 (22).jpg]

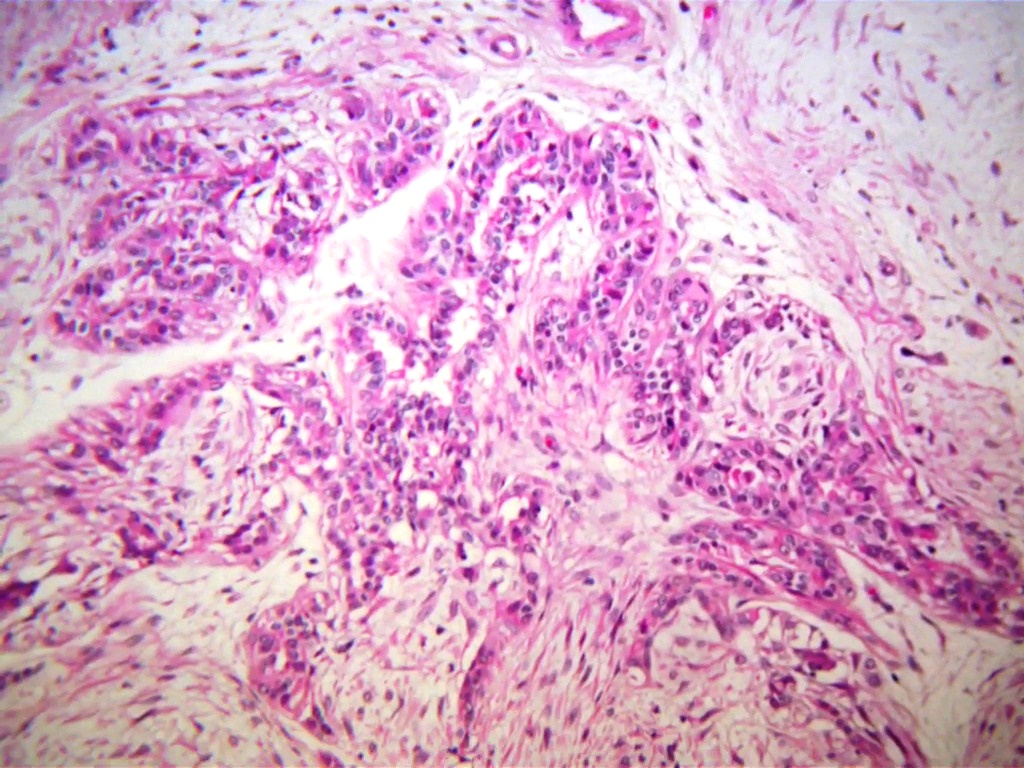

Supplement: Supplementary file 1 [file animals-13-01563-s001.zip › supplementary files/File S1 Canine Mammary Tumor Dataset/benign/Benign mixed tumor-466-11_FRM_008 (23).jpg]

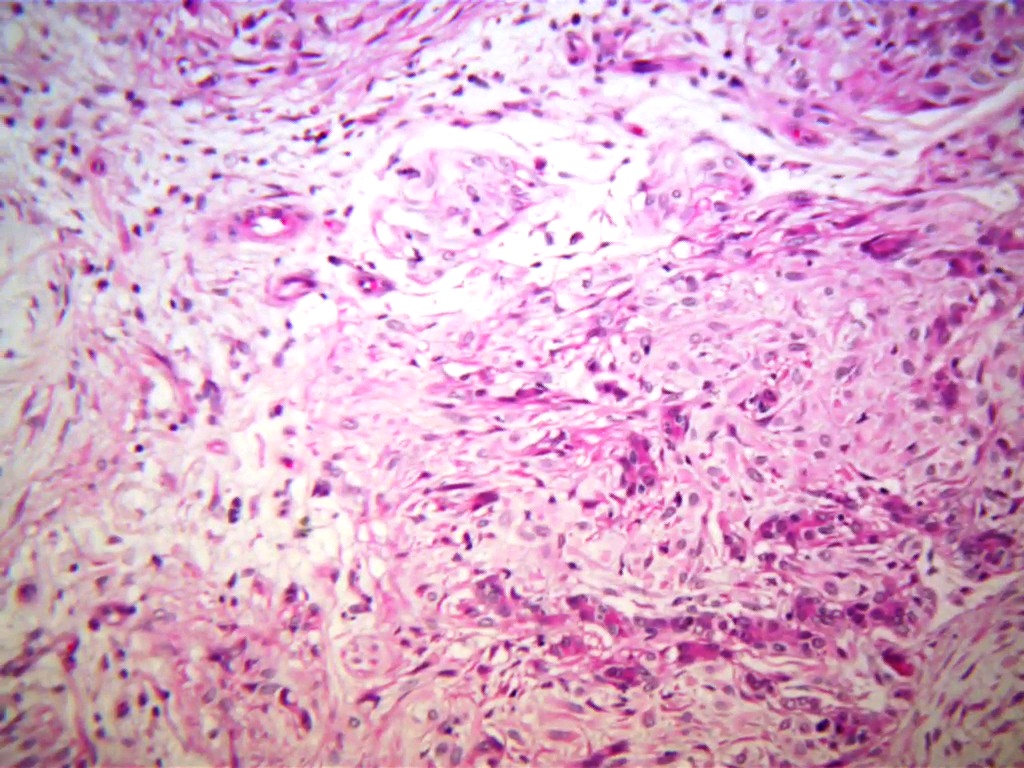

Supplement: Supplementary file 1 [file animals-13-01563-s001.zip › supplementary files/File S1 Canine Mammary Tumor Dataset/benign/Benign mixed tumor-466-11_FRM_008 (24).jpg]

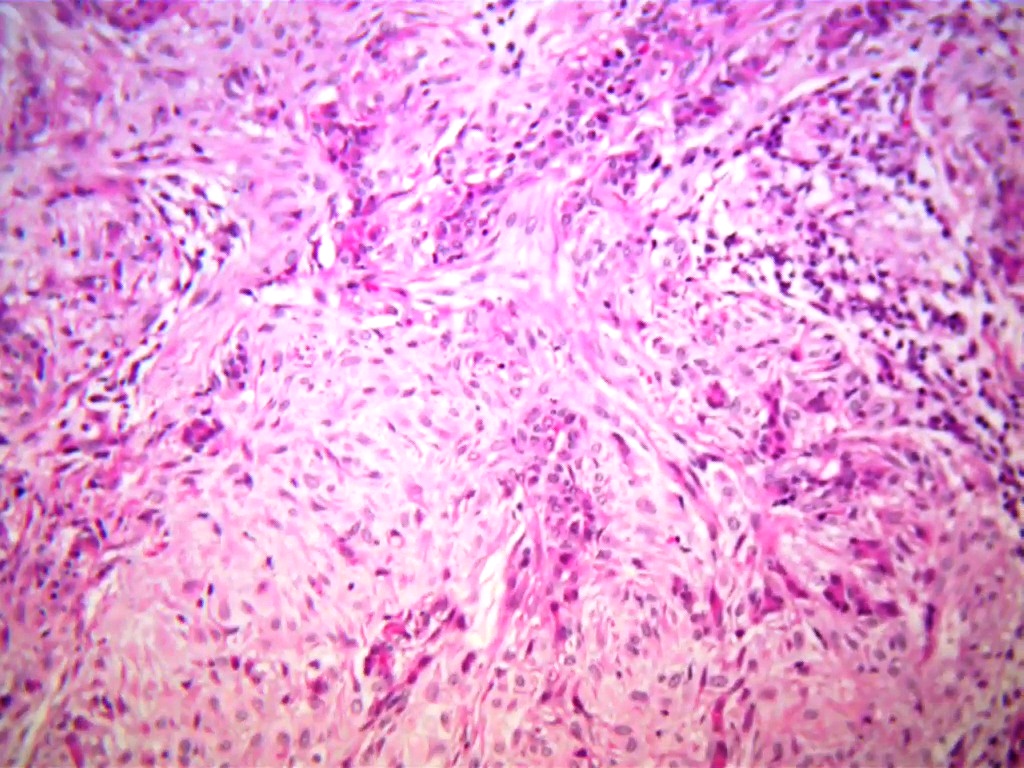

Supplement: Supplementary file 1 [file animals-13-01563-s001.zip › supplementary files/File S1 Canine Mammary Tumor Dataset/benign/Benign mixed tumor-466-11_FRM_008 (3).jpg]

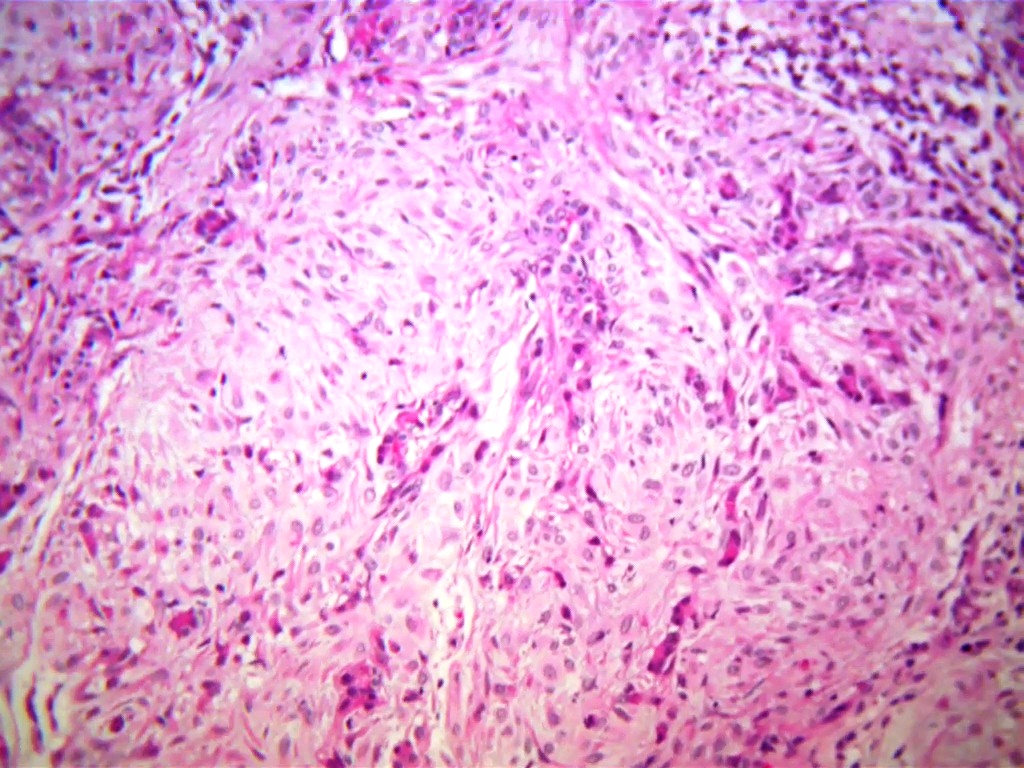

Supplement: Supplementary file 1 [file animals-13-01563-s001.zip › supplementary files/File S1 Canine Mammary Tumor Dataset/benign/Benign mixed tumor-466-11_FRM_008 (4).jpg]

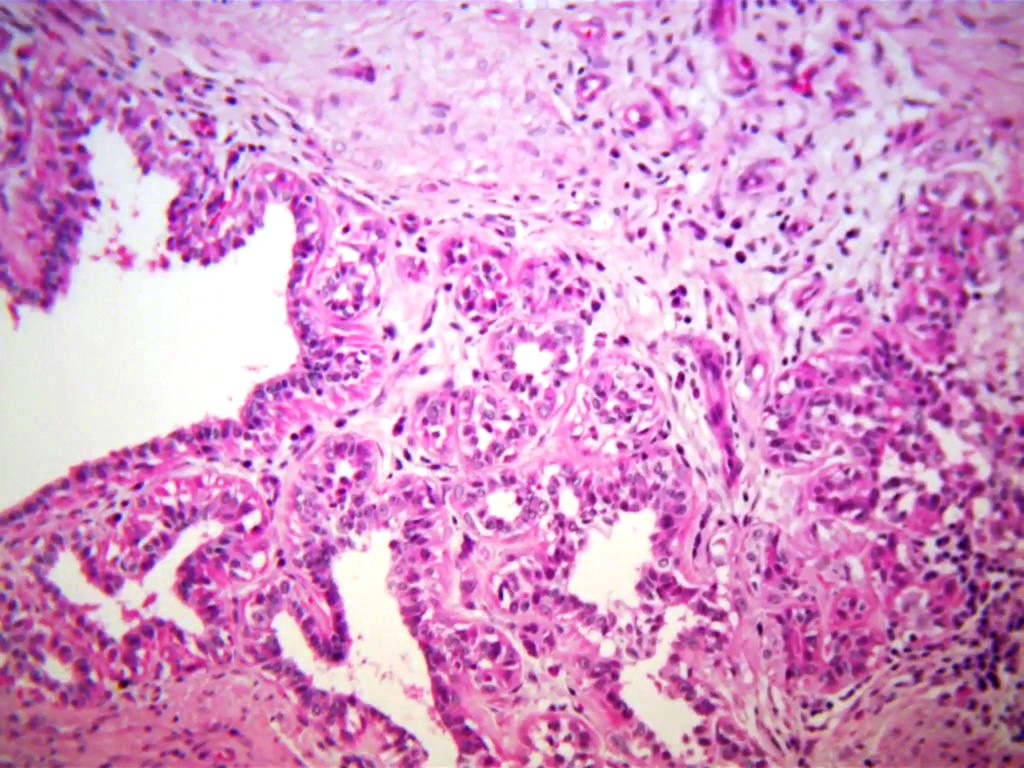

Supplement: Supplementary file 1 [file animals-13-01563-s001.zip › supplementary files/File S1 Canine Mammary Tumor Dataset/benign/Benign mixed tumor-466-11_FRM_008 (5).jpg]

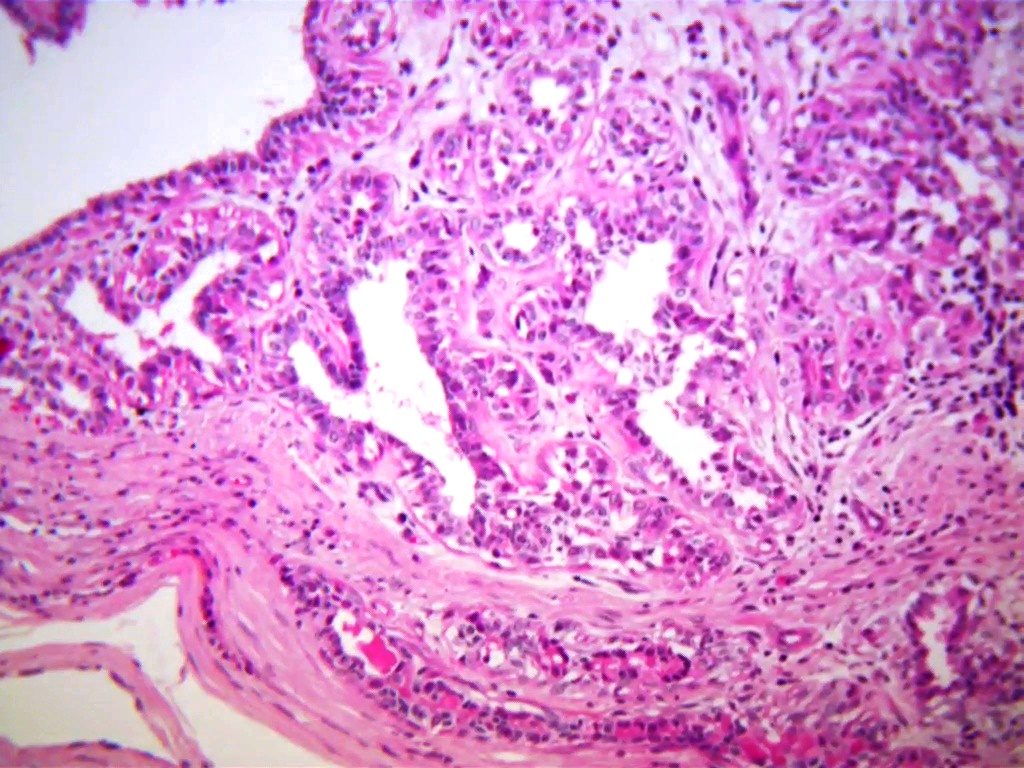

Supplement: Supplementary file 1 [file animals-13-01563-s001.zip › supplementary files/File S1 Canine Mammary Tumor Dataset/benign/Benign mixed tumor-466-11_FRM_008 (6).jpg]

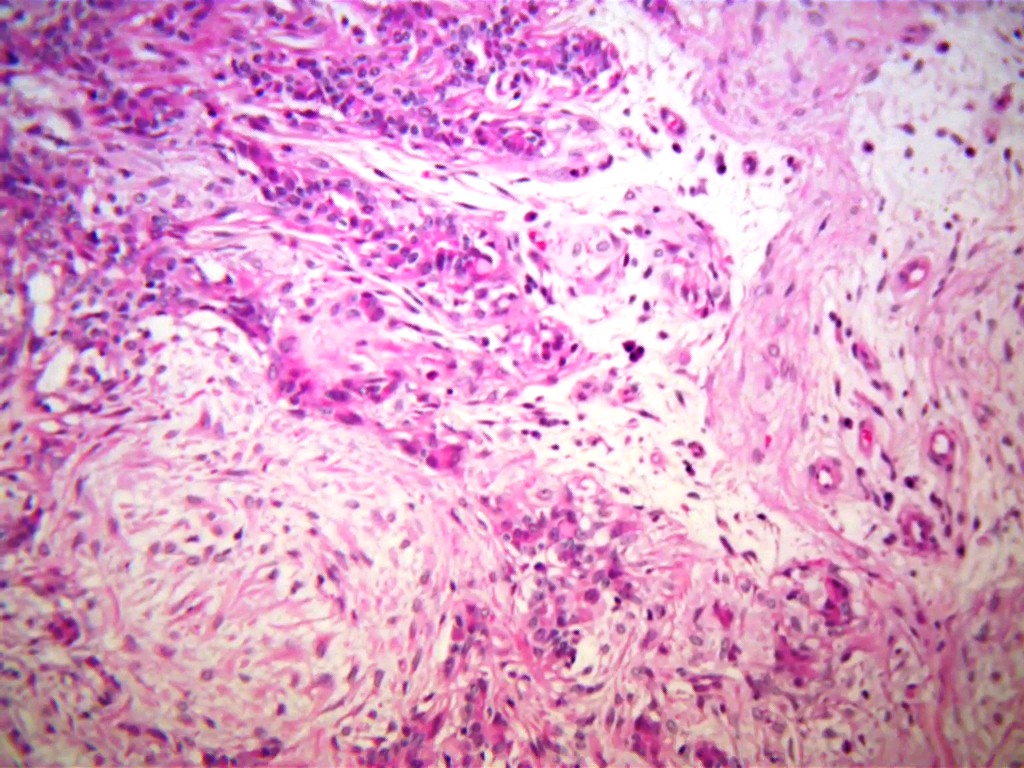

Supplement: Supplementary file 1 [file animals-13-01563-s001.zip › supplementary files/File S1 Canine Mammary Tumor Dataset/benign/Benign mixed tumor-466-11_FRM_008 (7).jpg]

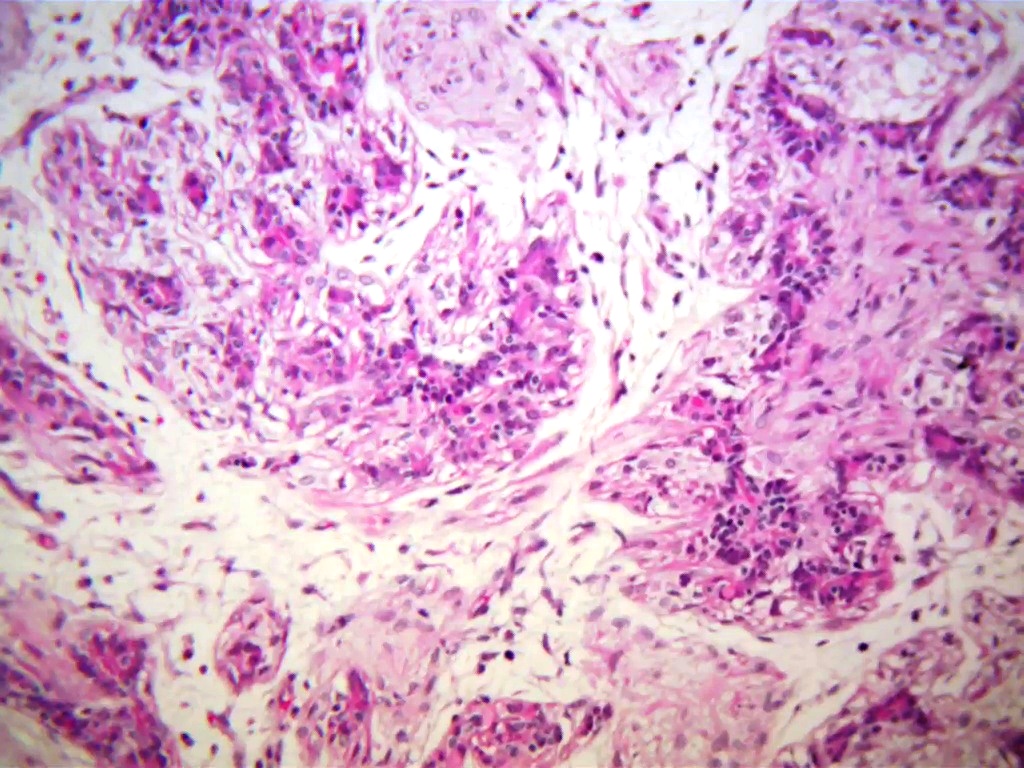

Supplement: Supplementary file 1 [file animals-13-01563-s001.zip › supplementary files/File S1 Canine Mammary Tumor Dataset/benign/Benign mixed tumor-466-11_FRM_008 (8).jpg]

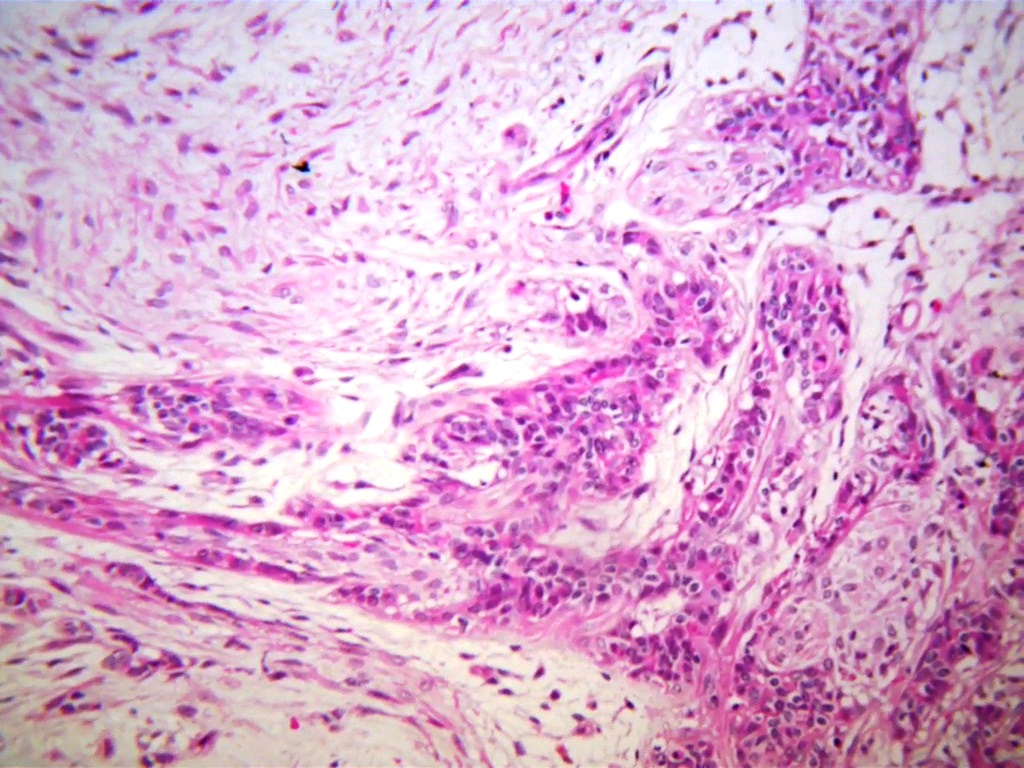

Supplement: Supplementary file 1 [file animals-13-01563-s001.zip › supplementary files/File S1 Canine Mammary Tumor Dataset/benign/Benign mixed tumor-466-11_FRM_008 (9).jpg]

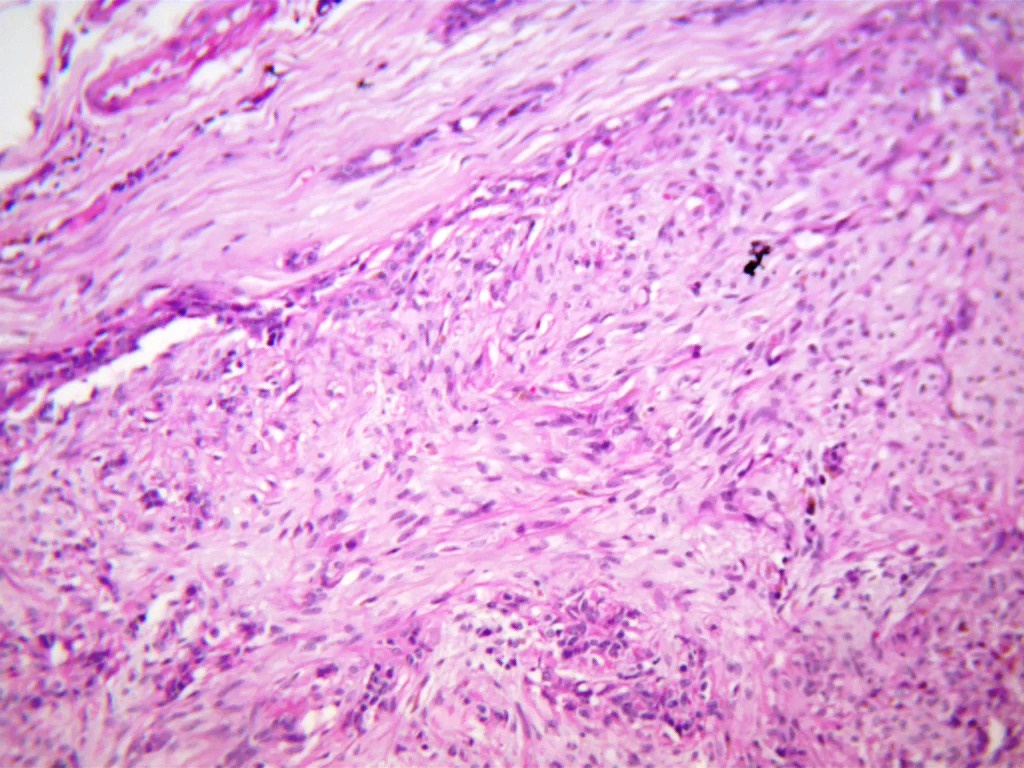

Supplement: Supplementary file 1 [file animals-13-01563-s001.zip › supplementary files/File S1 Canine Mammary Tumor Dataset/benign/Benign mixed tumor-72-13V2_FRM_000 (1).jpg]

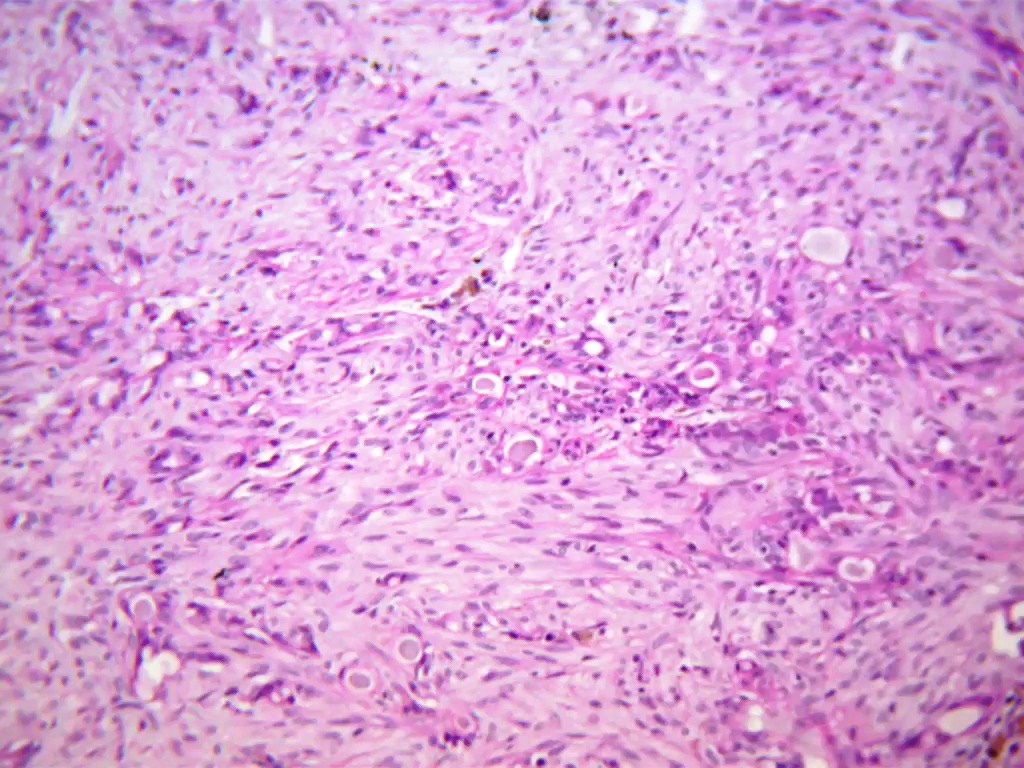

Supplement: Supplementary file 1 [file animals-13-01563-s001.zip › supplementary files/File S1 Canine Mammary Tumor Dataset/benign/Benign mixed tumor-72-13V2_FRM_000 (10).jpg]

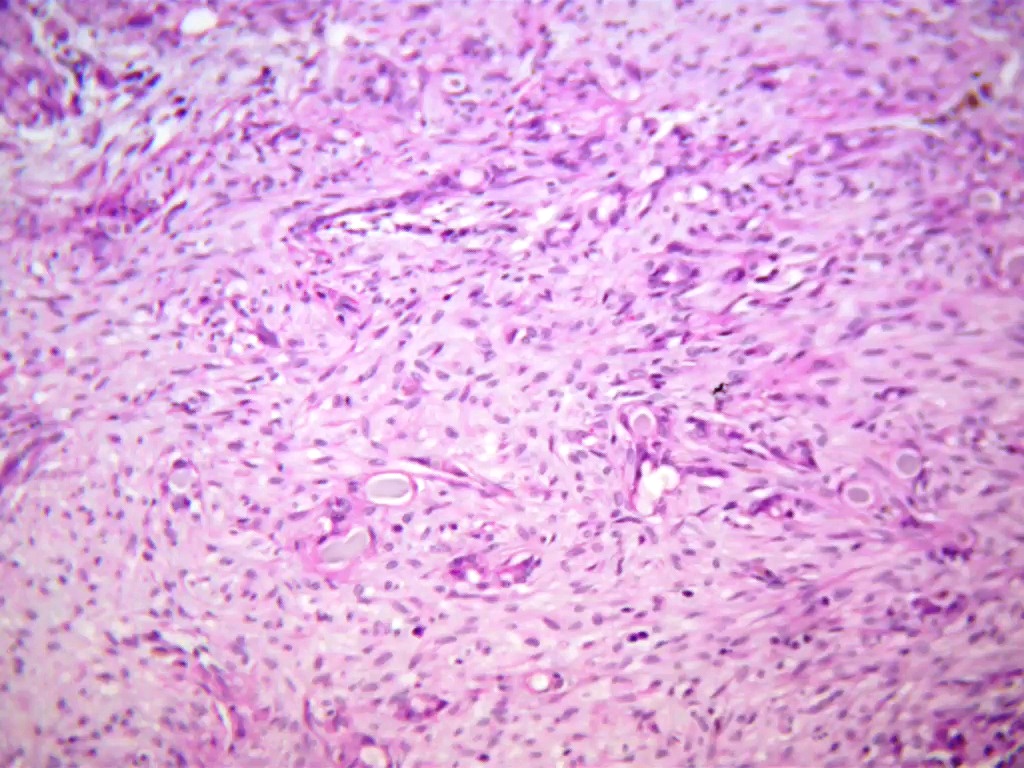

Supplement: Supplementary file 1 [file animals-13-01563-s001.zip › supplementary files/File S1 Canine Mammary Tumor Dataset/benign/Benign mixed tumor-72-13V2_FRM_000 (11).jpg]

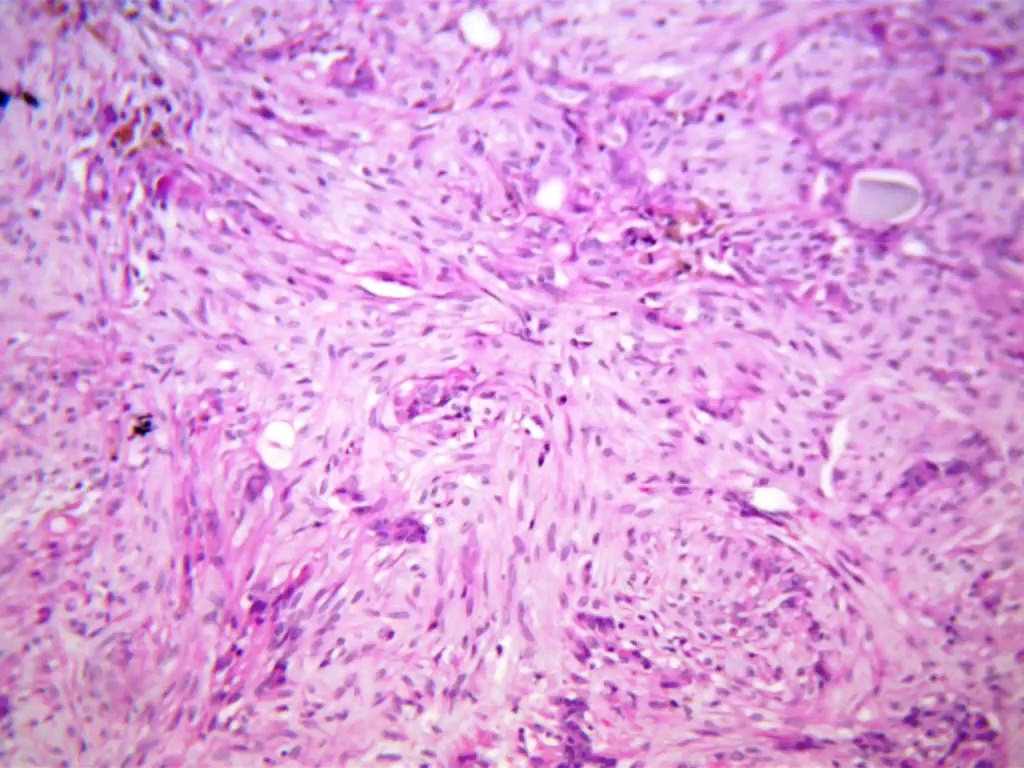

Supplement: Supplementary file 1 [file animals-13-01563-s001.zip › supplementary files/File S1 Canine Mammary Tumor Dataset/benign/Benign mixed tumor-72-13V2_FRM_000 (12).jpg]

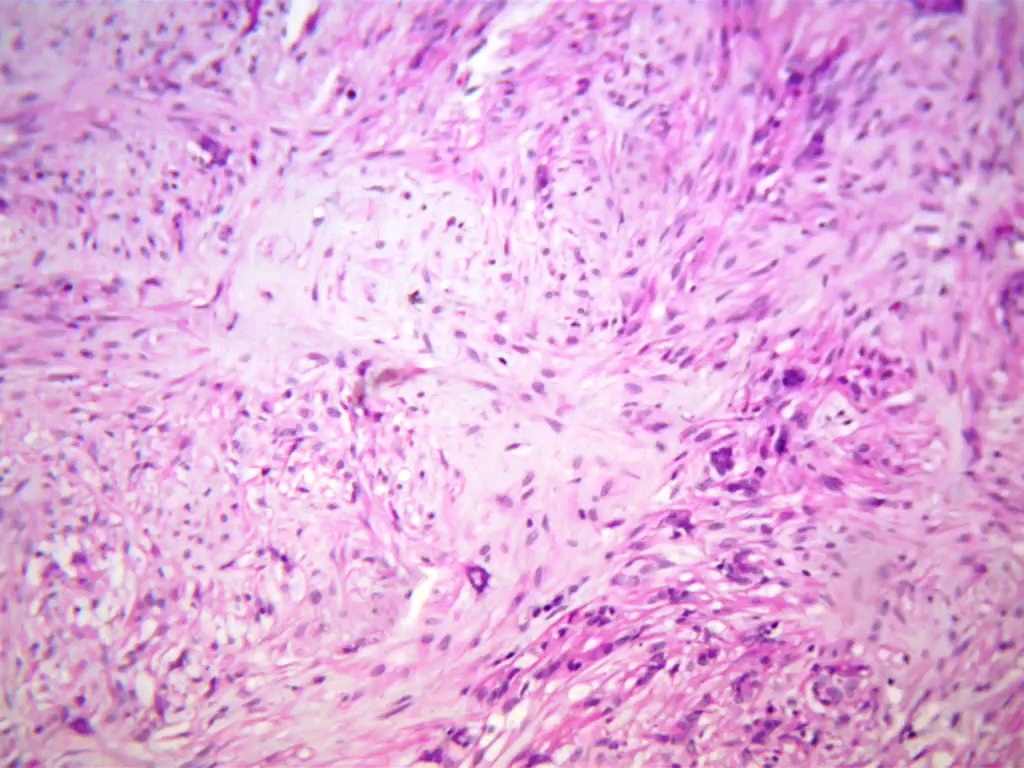

Supplement: Supplementary file 1 [file animals-13-01563-s001.zip › supplementary files/File S1 Canine Mammary Tumor Dataset/benign/Benign mixed tumor-72-13V2_FRM_000 (13).jpg]

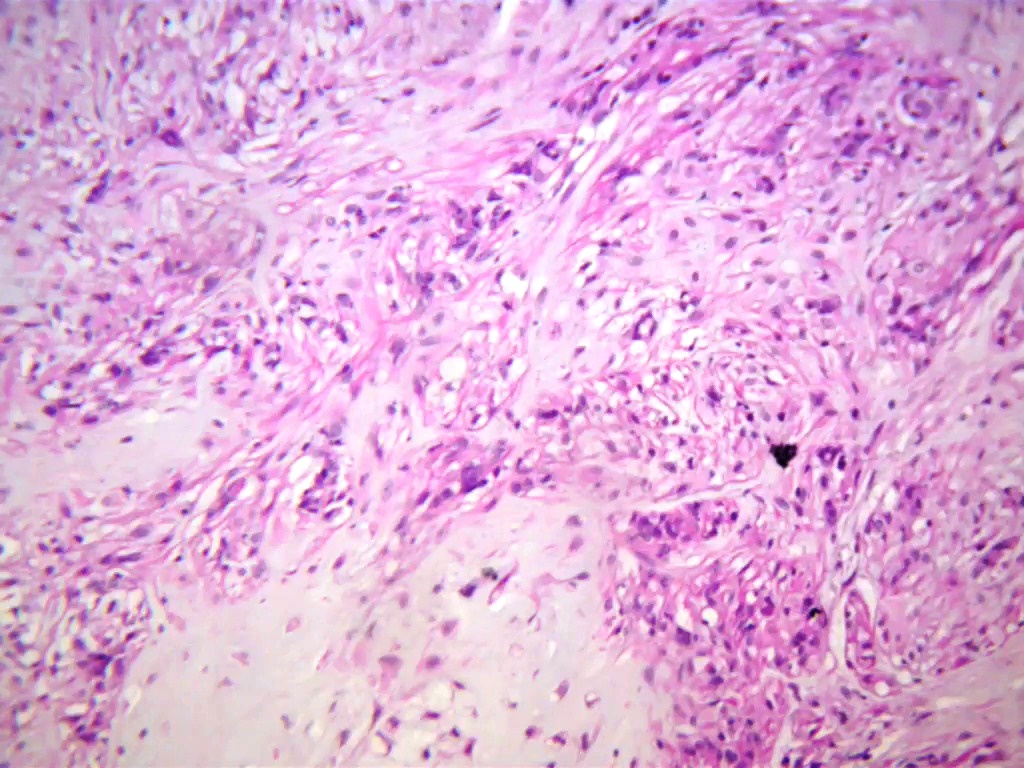

Supplement: Supplementary file 1 [file animals-13-01563-s001.zip › supplementary files/File S1 Canine Mammary Tumor Dataset/benign/Benign mixed tumor-72-13V2_FRM_000 (14).jpg]

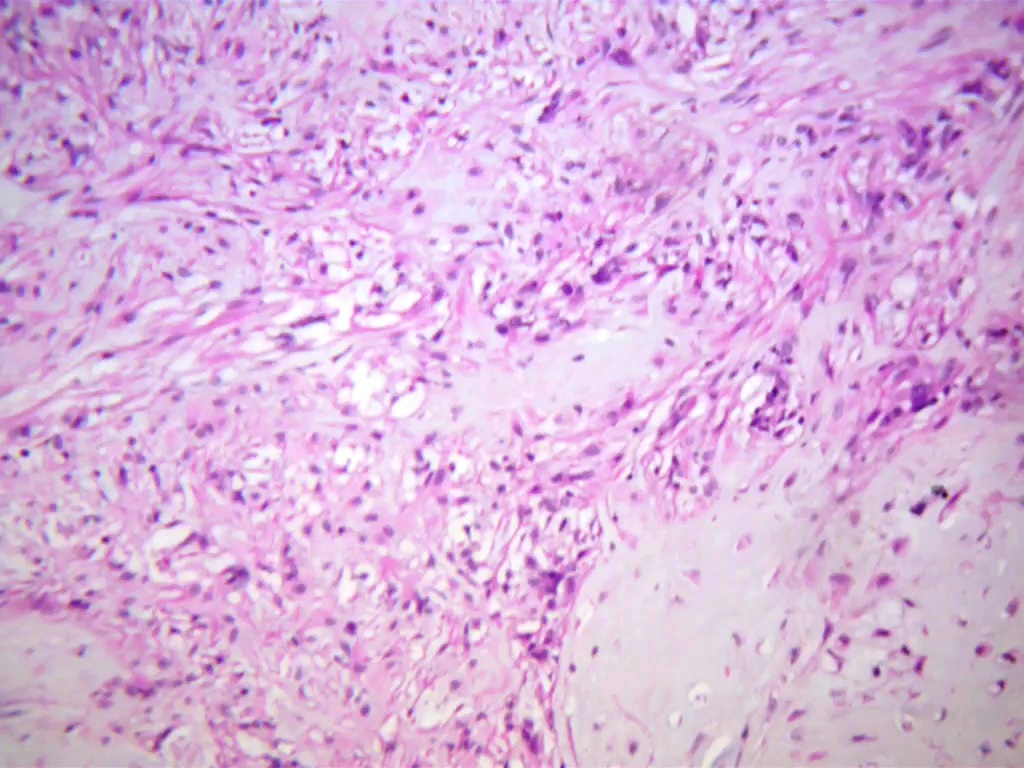

Supplement: Supplementary file 1 [file animals-13-01563-s001.zip › supplementary files/File S1 Canine Mammary Tumor Dataset/benign/Benign mixed tumor-72-13V2_FRM_000 (15).jpg]

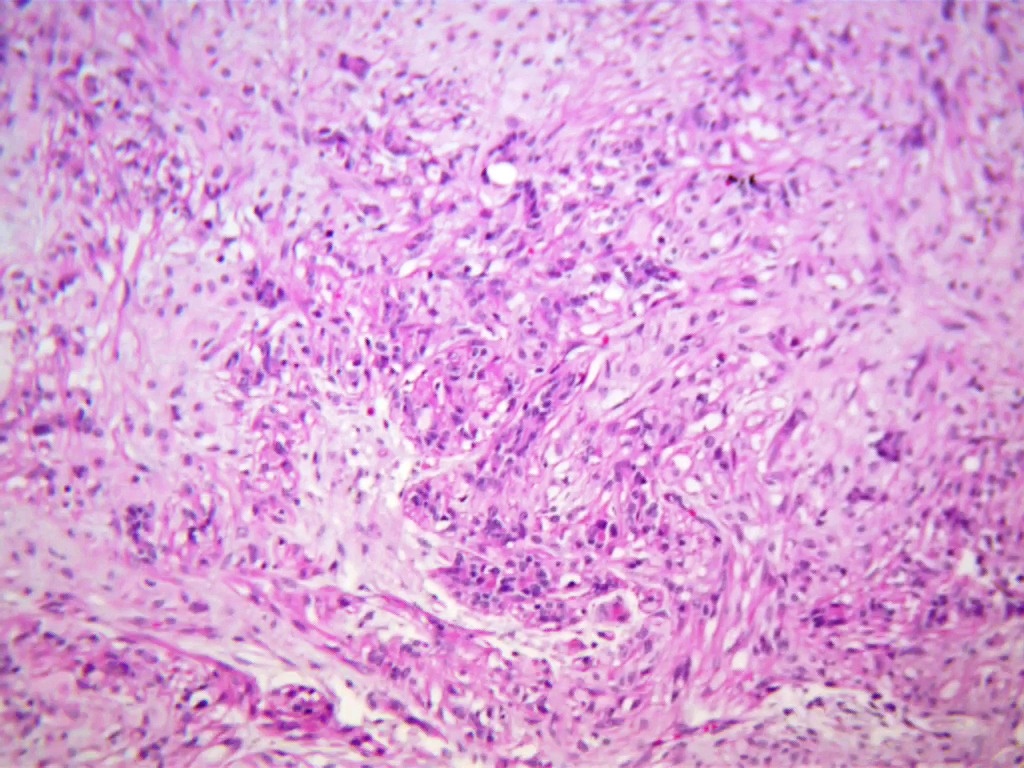

Supplement: Supplementary file 1 [file animals-13-01563-s001.zip › supplementary files/File S1 Canine Mammary Tumor Dataset/benign/Benign mixed tumor-72-13V2_FRM_000 (16).jpg]

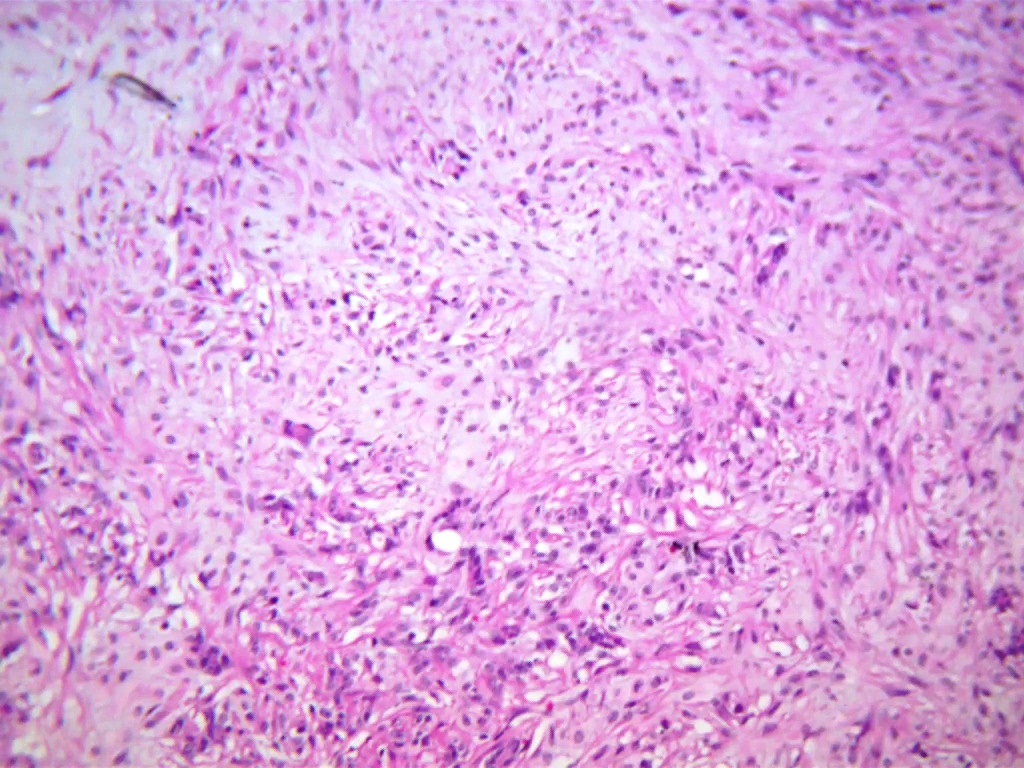

Supplement: Supplementary file 1 [file animals-13-01563-s001.zip › supplementary files/File S1 Canine Mammary Tumor Dataset/benign/Benign mixed tumor-72-13V2_FRM_000 (17).jpg]

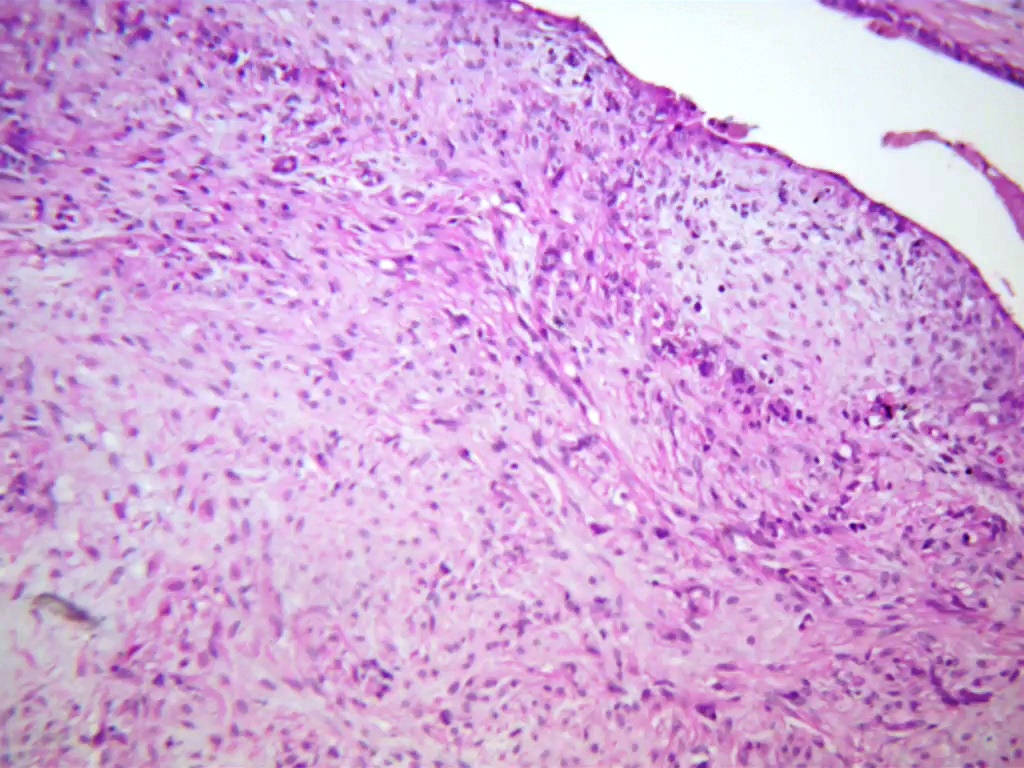

Supplement: Supplementary file 1 [file animals-13-01563-s001.zip › supplementary files/File S1 Canine Mammary Tumor Dataset/benign/Benign mixed tumor-72-13V2_FRM_000 (18).jpg]

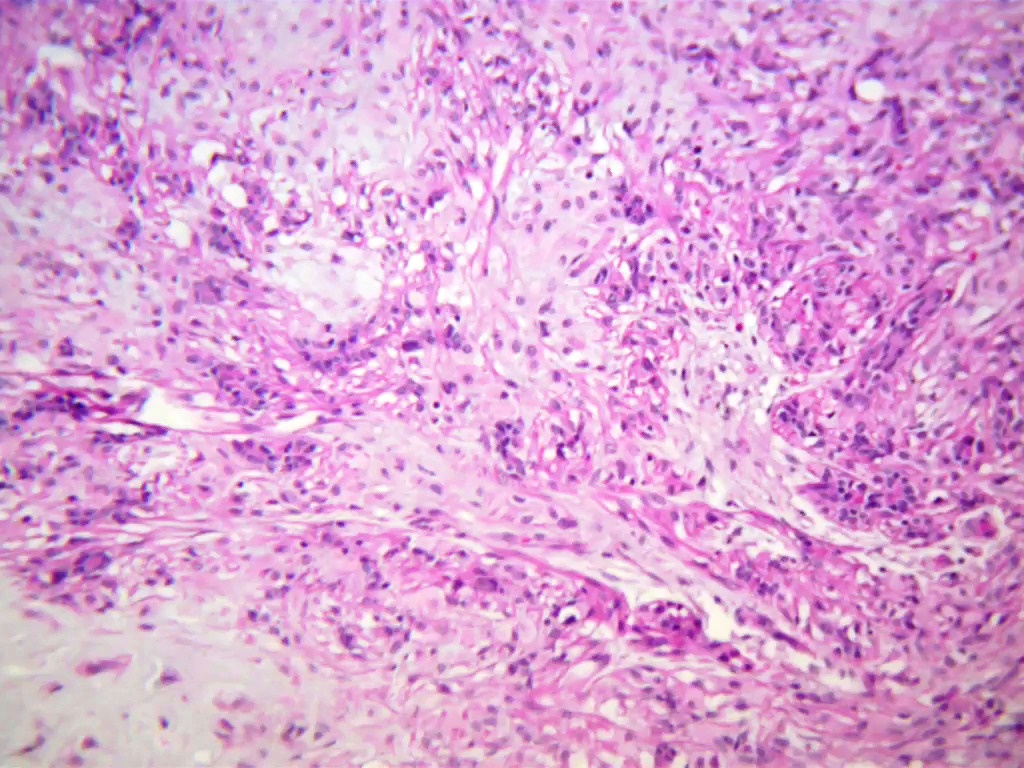

Supplement: Supplementary file 1 [file animals-13-01563-s001.zip › supplementary files/File S1 Canine Mammary Tumor Dataset/benign/Benign mixed tumor-72-13V2_FRM_000 (19).jpg]

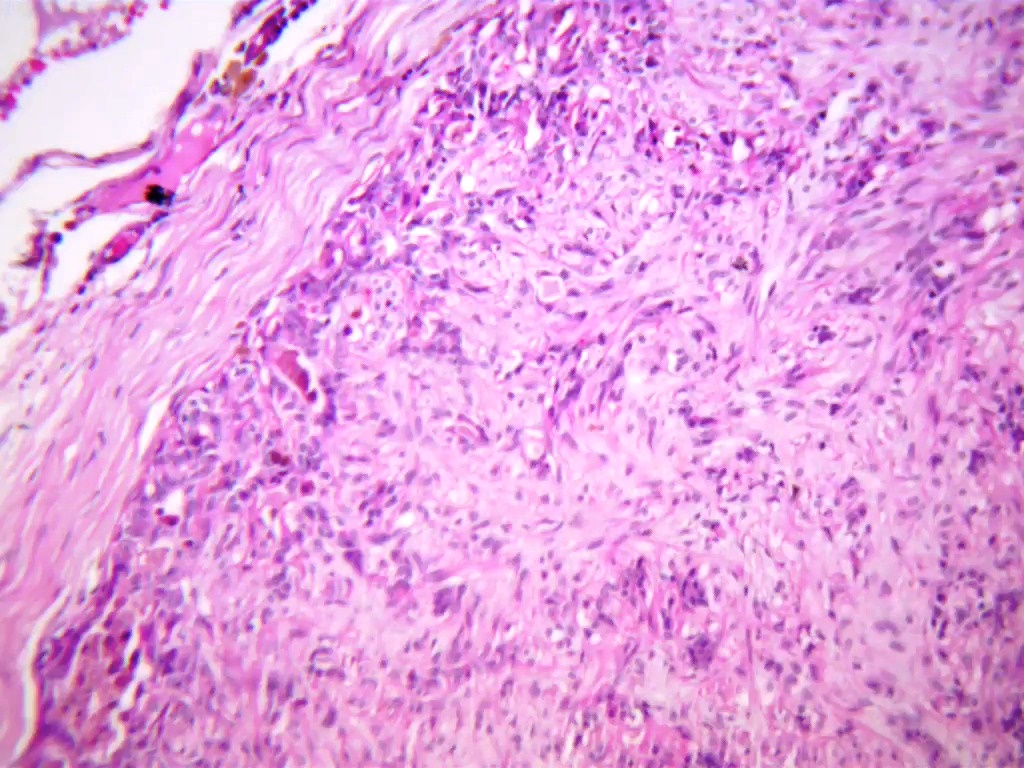

Supplement: Supplementary file 1 [file animals-13-01563-s001.zip › supplementary files/File S1 Canine Mammary Tumor Dataset/benign/Benign mixed tumor-72-13V2_FRM_000 (2).jpg]

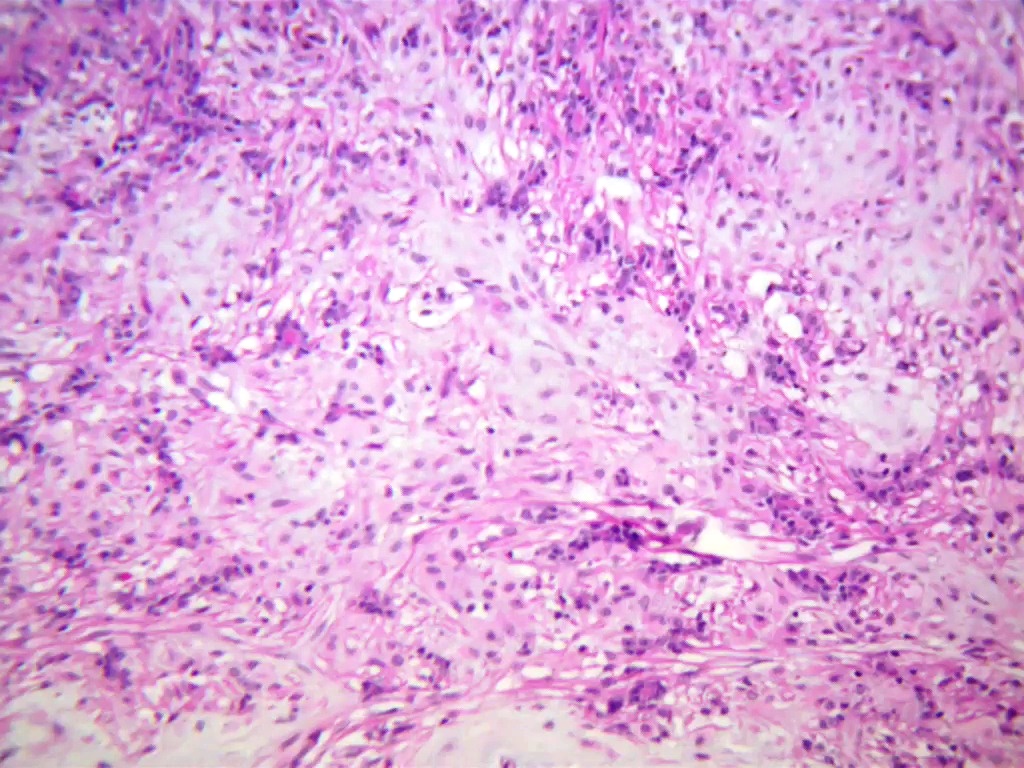

Supplement: Supplementary file 1 [file animals-13-01563-s001.zip › supplementary files/File S1 Canine Mammary Tumor Dataset/benign/Benign mixed tumor-72-13V2_FRM_000 (20).jpg]

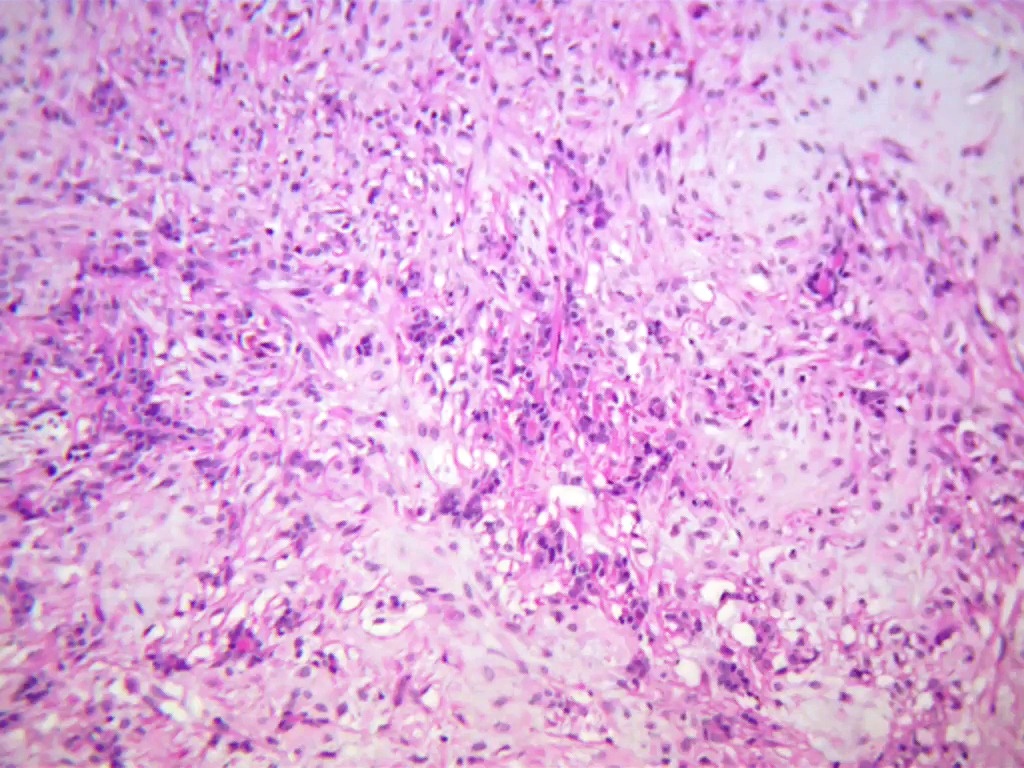

Supplement: Supplementary file 1 [file animals-13-01563-s001.zip › supplementary files/File S1 Canine Mammary Tumor Dataset/benign/Benign mixed tumor-72-13V2_FRM_000 (21).jpg]

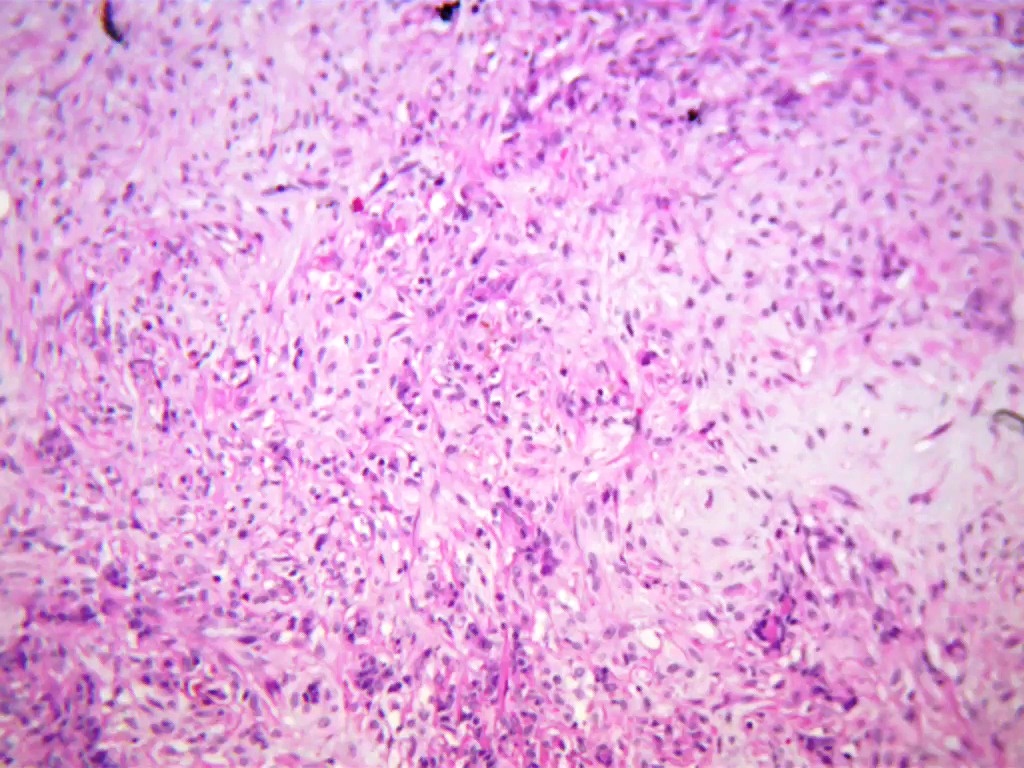

Supplement: Supplementary file 1 [file animals-13-01563-s001.zip › supplementary files/File S1 Canine Mammary Tumor Dataset/benign/Benign mixed tumor-72-13V2_FRM_000 (22).jpg]

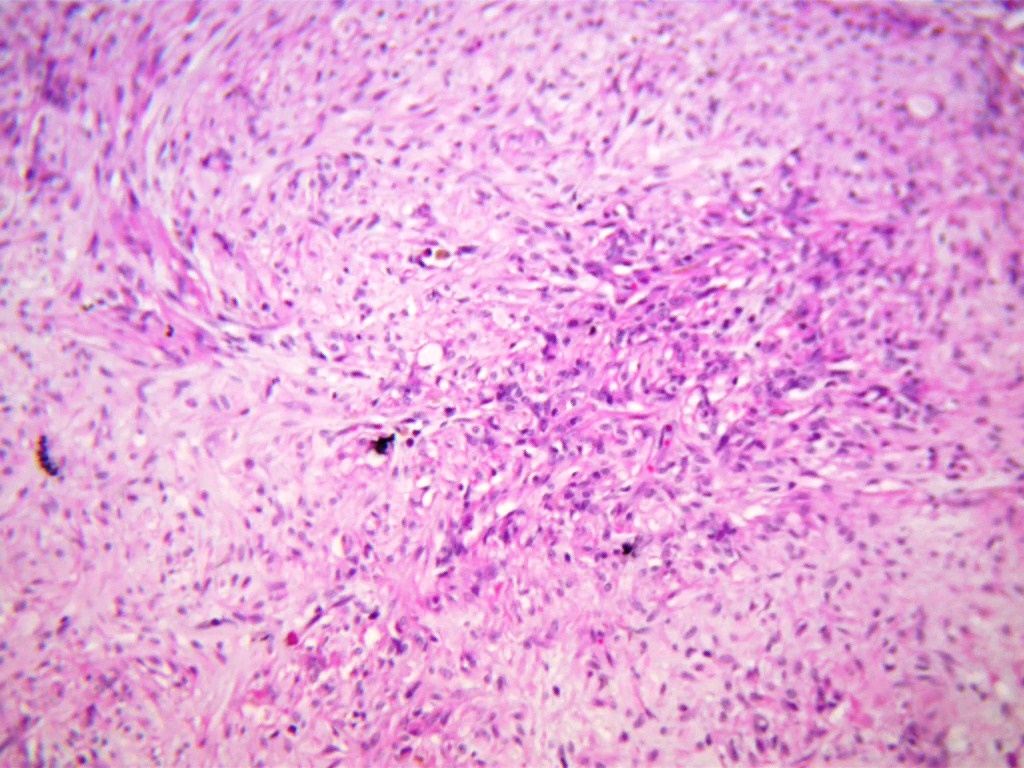

Supplement: Supplementary file 1 [file animals-13-01563-s001.zip › supplementary files/File S1 Canine Mammary Tumor Dataset/benign/Benign mixed tumor-72-13V2_FRM_000 (23).jpg]

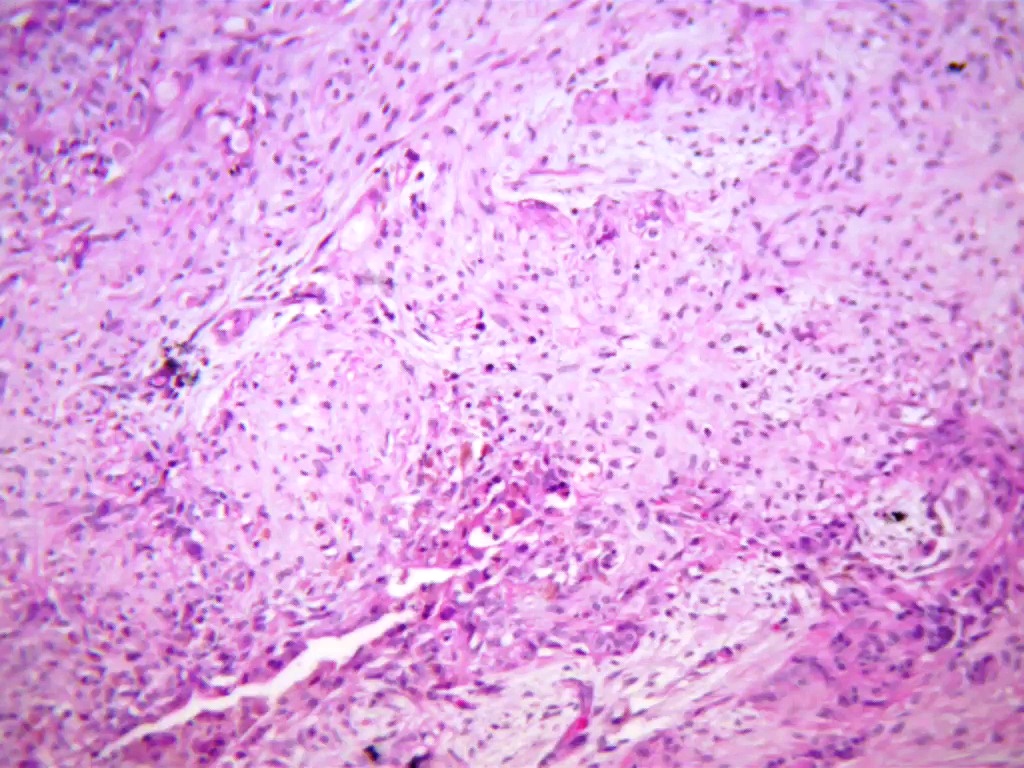

Supplement: Supplementary file 1 [file animals-13-01563-s001.zip › supplementary files/File S1 Canine Mammary Tumor Dataset/benign/Benign mixed tumor-72-13V2_FRM_000 (24).jpg]

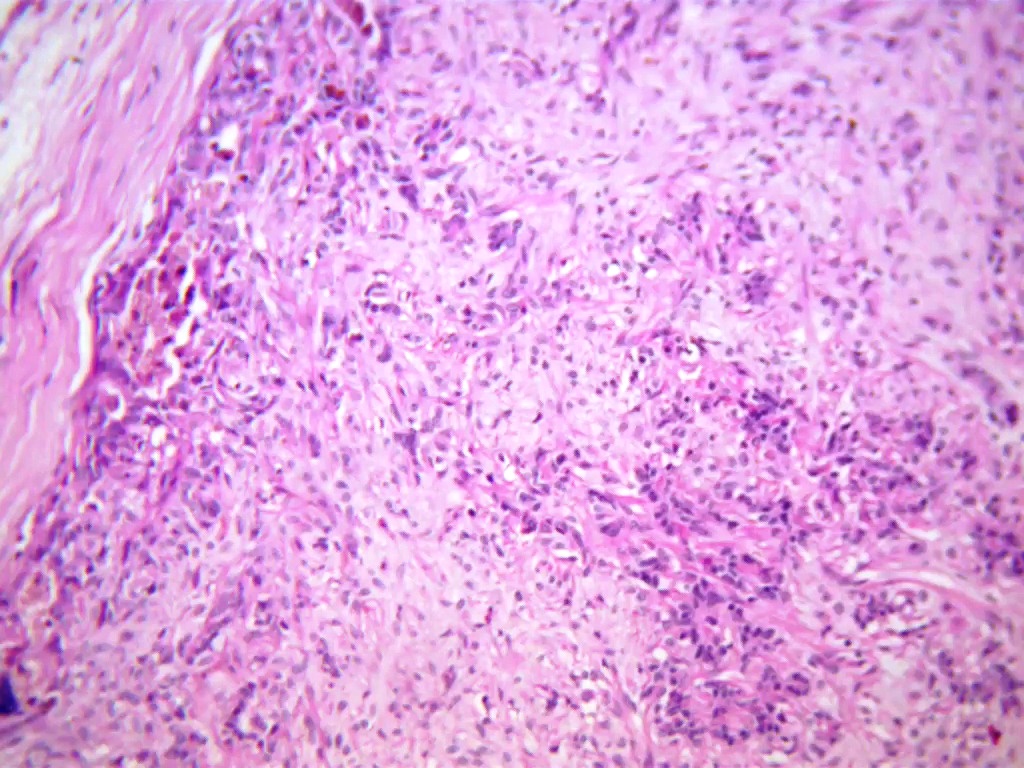

Supplement: Supplementary file 1 [file animals-13-01563-s001.zip › supplementary files/File S1 Canine Mammary Tumor Dataset/benign/Benign mixed tumor-72-13V2_FRM_000 (3).jpg]

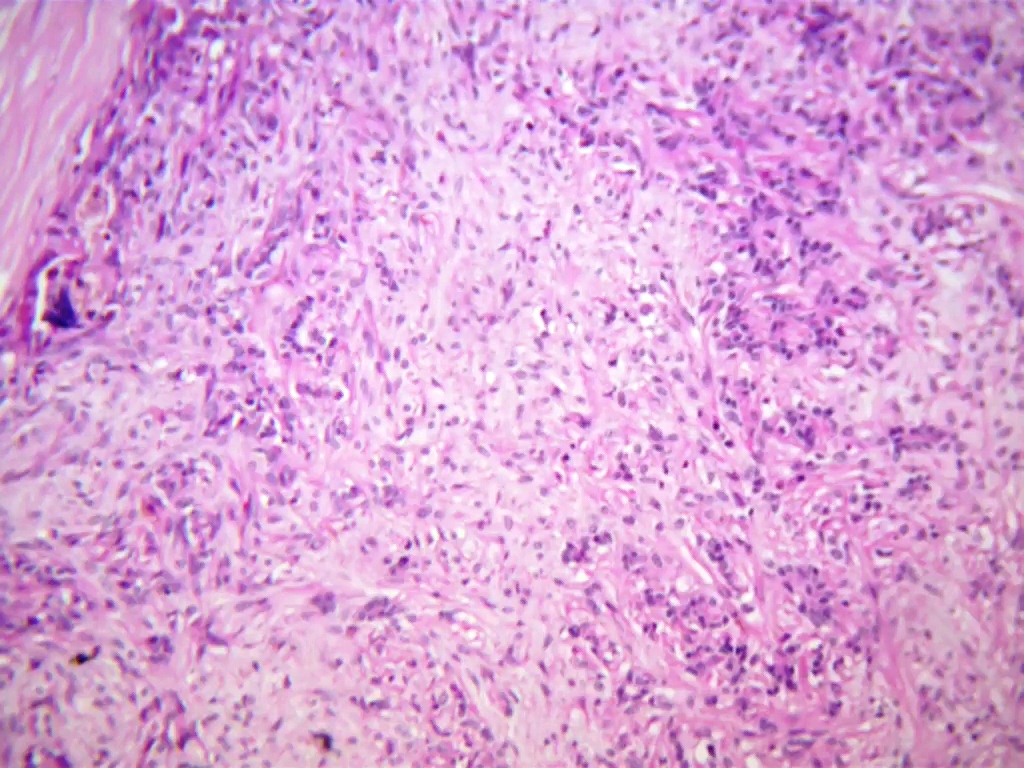

Supplement: Supplementary file 1 [file animals-13-01563-s001.zip › supplementary files/File S1 Canine Mammary Tumor Dataset/benign/Benign mixed tumor-72-13V2_FRM_000 (4).jpg]

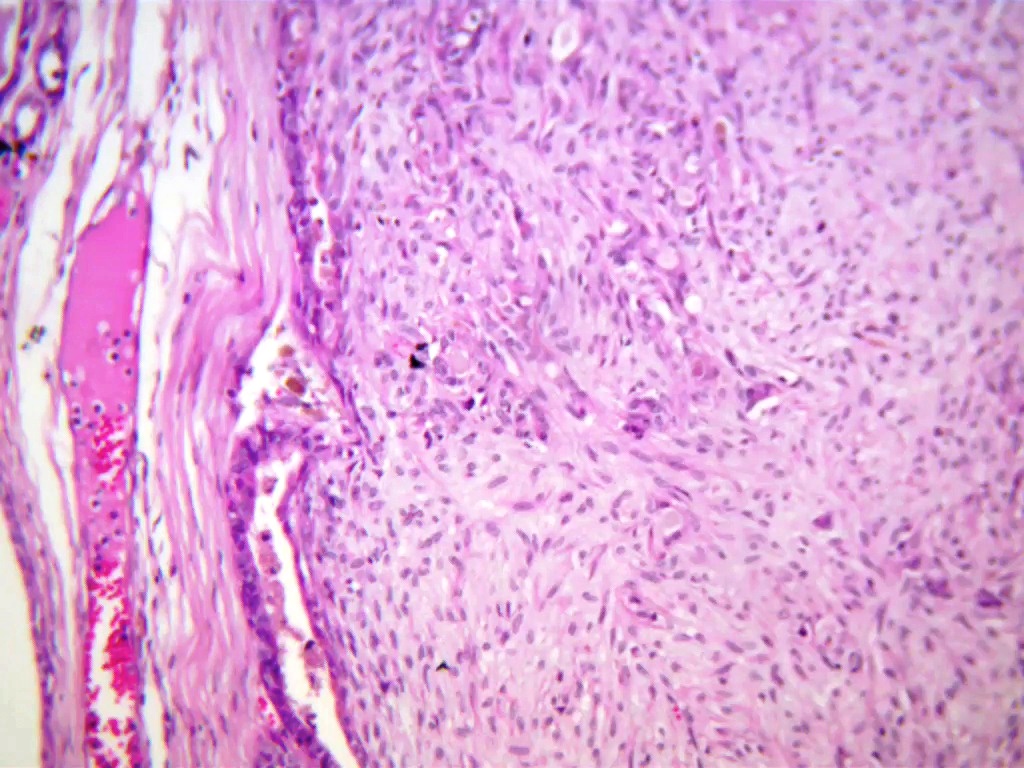

Supplement: Supplementary file 1 [file animals-13-01563-s001.zip › supplementary files/File S1 Canine Mammary Tumor Dataset/benign/Benign mixed tumor-72-13V2_FRM_000 (5).jpg]

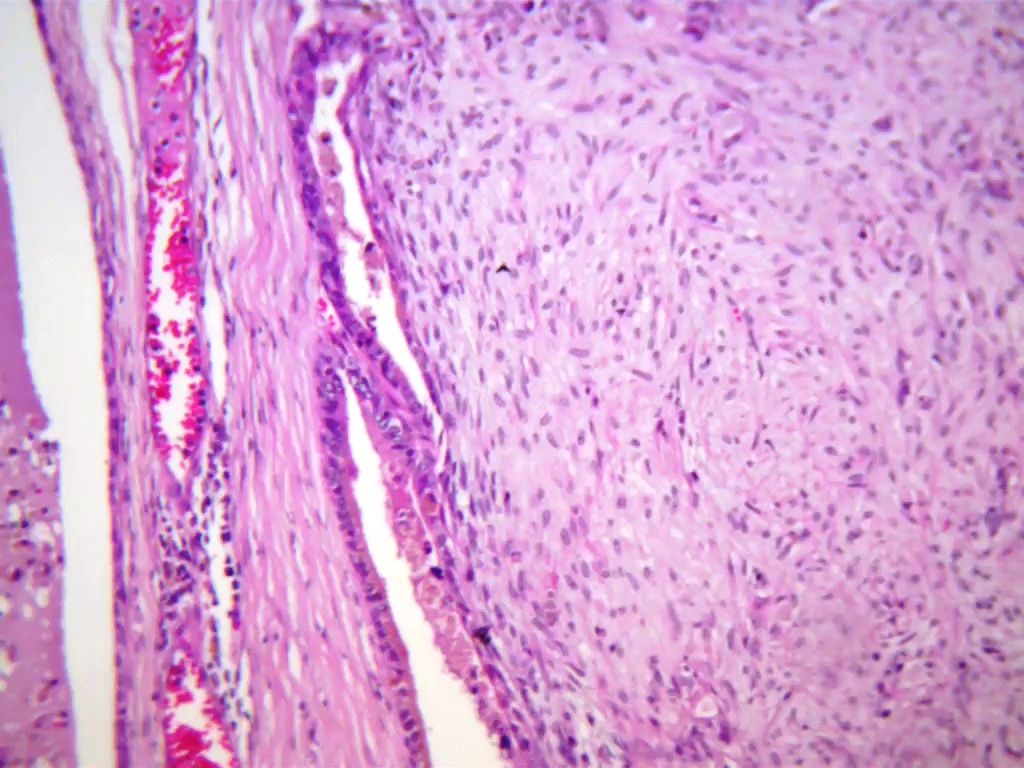

Supplement: Supplementary file 1 [file animals-13-01563-s001.zip › supplementary files/File S1 Canine Mammary Tumor Dataset/benign/Benign mixed tumor-72-13V2_FRM_000 (6).jpg]

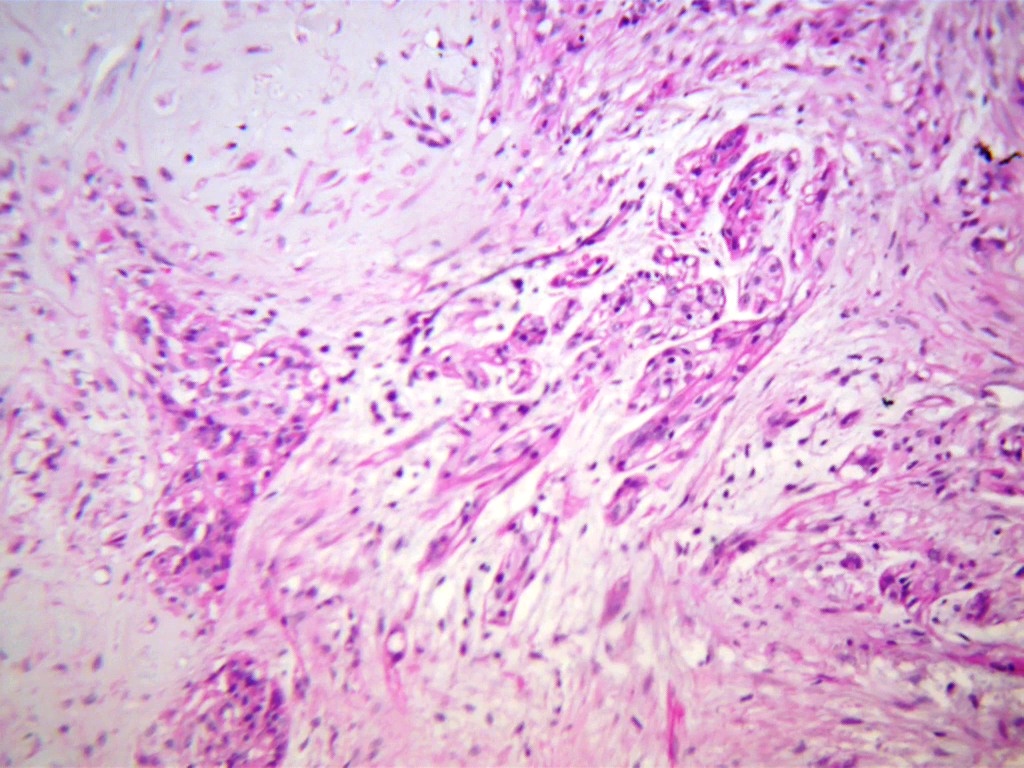

Supplement: Supplementary file 1 [file animals-13-01563-s001.zip › supplementary files/File S1 Canine Mammary Tumor Dataset/benign/Benign mixed tumor-72-13V2_FRM_000 (7).jpg]

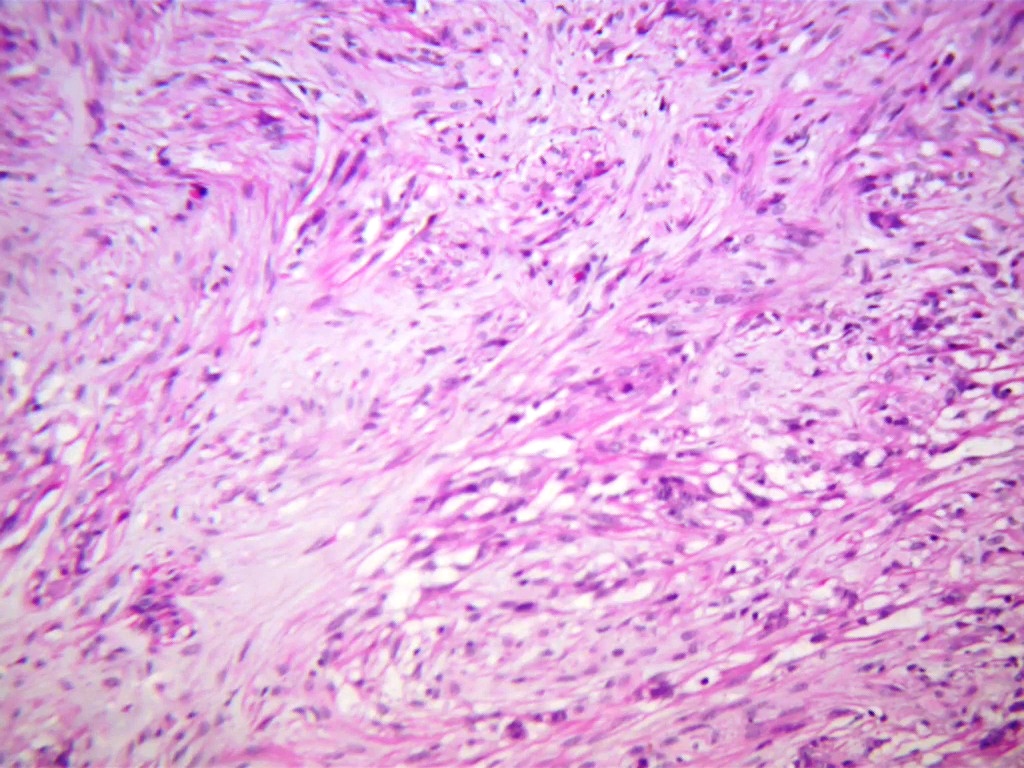

Supplement: Supplementary file 1 [file animals-13-01563-s001.zip › supplementary files/File S1 Canine Mammary Tumor Dataset/benign/Benign mixed tumor-72-13V2_FRM_000 (8).jpg]

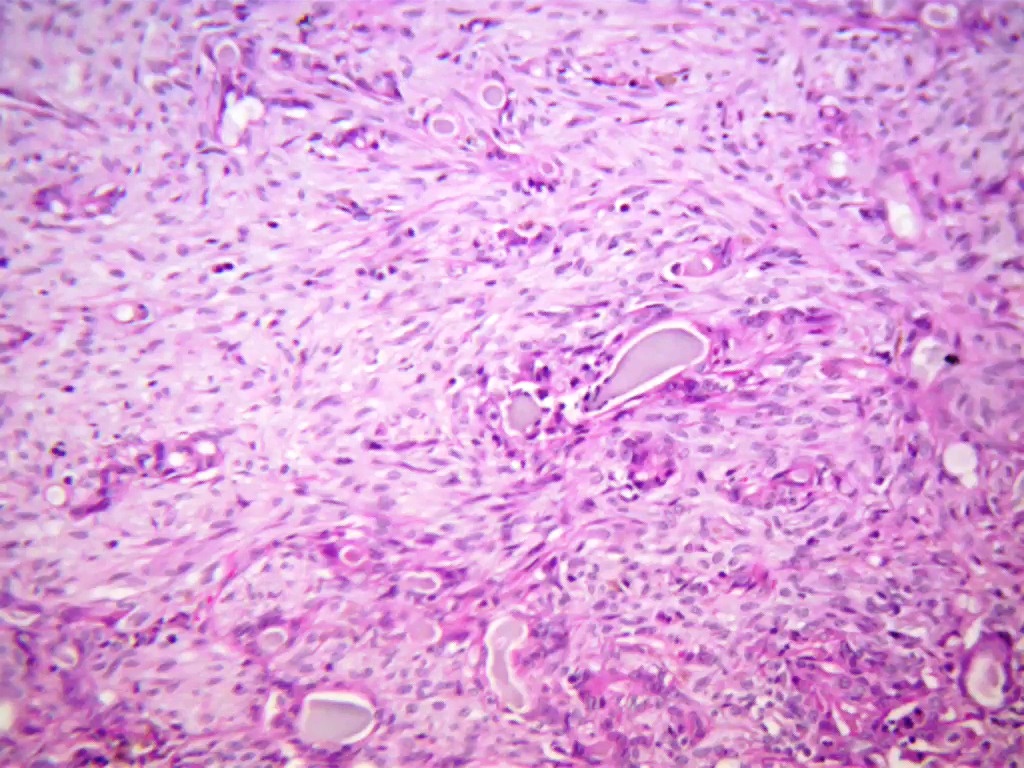

Supplement: Supplementary file 1 [file animals-13-01563-s001.zip › supplementary files/File S1 Canine Mammary Tumor Dataset/benign/Benign mixed tumor-72-13V2_FRM_000 (9).jpg]

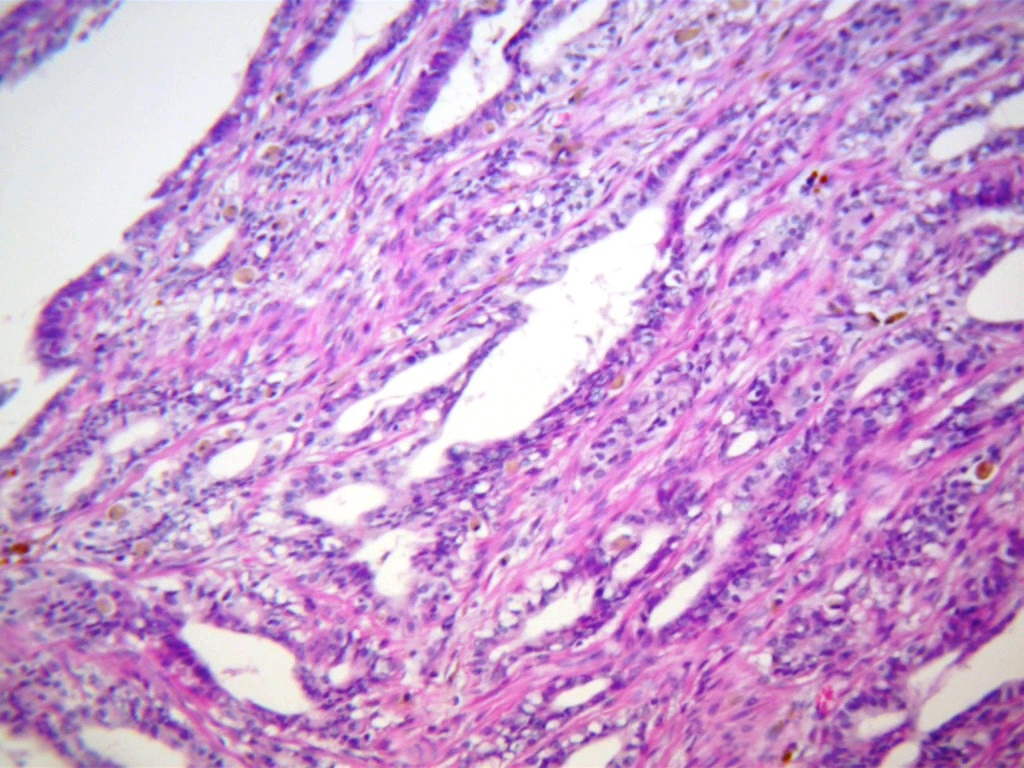

Supplement: Supplementary file 1 [file animals-13-01563-s001.zip › supplementary files/File S1 Canine Mammary Tumor Dataset/benign/Complex adenoma_208-12V2_FRM_065 (1).jpg]

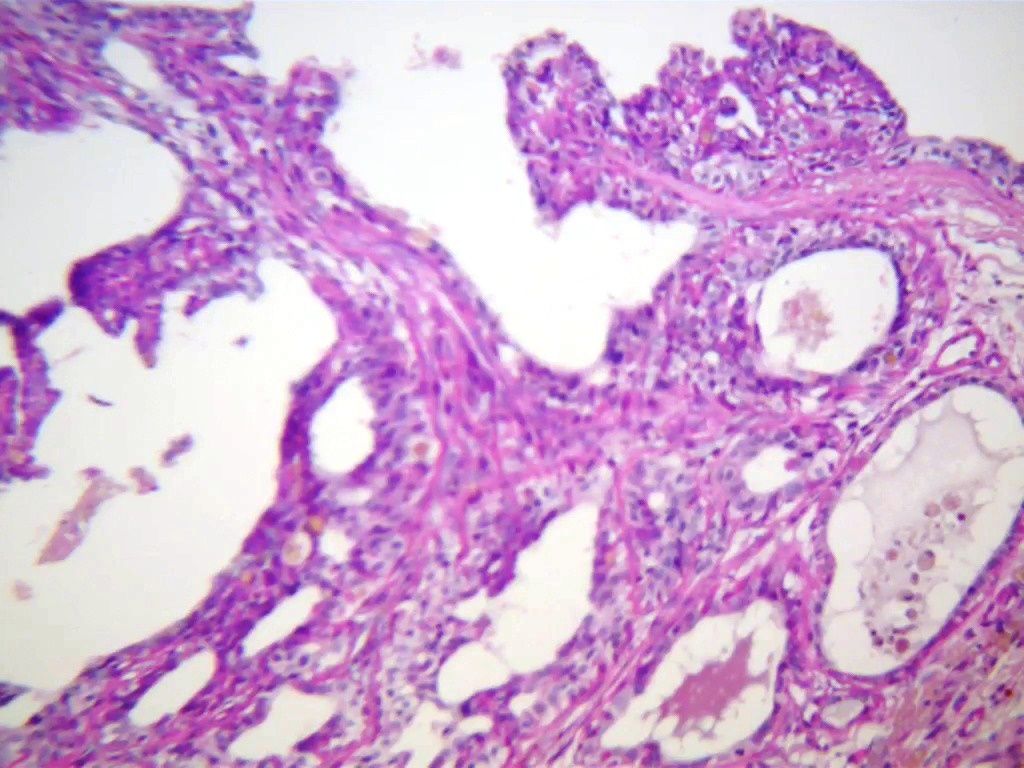

Supplement: Supplementary file 1 [file animals-13-01563-s001.zip › supplementary files/File S1 Canine Mammary Tumor Dataset/benign/Complex adenoma_208-12V2_FRM_065 (10).jpg]

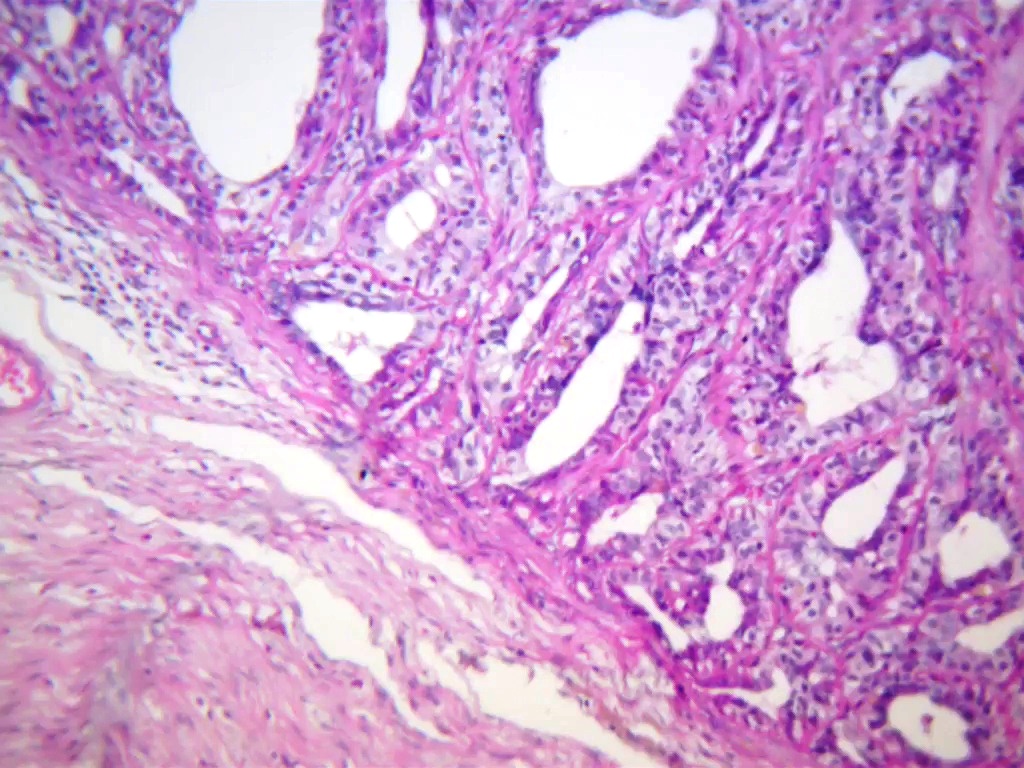

Supplement: Supplementary file 1 [file animals-13-01563-s001.zip › supplementary files/File S1 Canine Mammary Tumor Dataset/benign/Complex adenoma_208-12V2_FRM_065 (11).jpg]

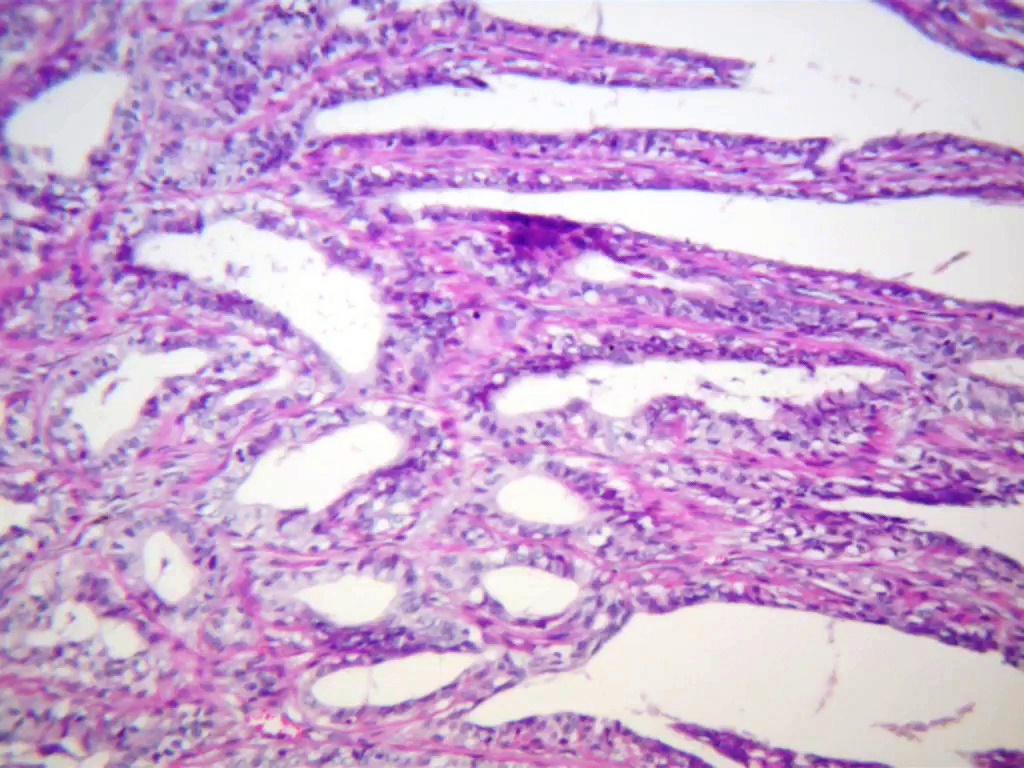

Supplement: Supplementary file 1 [file animals-13-01563-s001.zip › supplementary files/File S1 Canine Mammary Tumor Dataset/benign/Complex adenoma_208-12V2_FRM_065 (12).jpg]

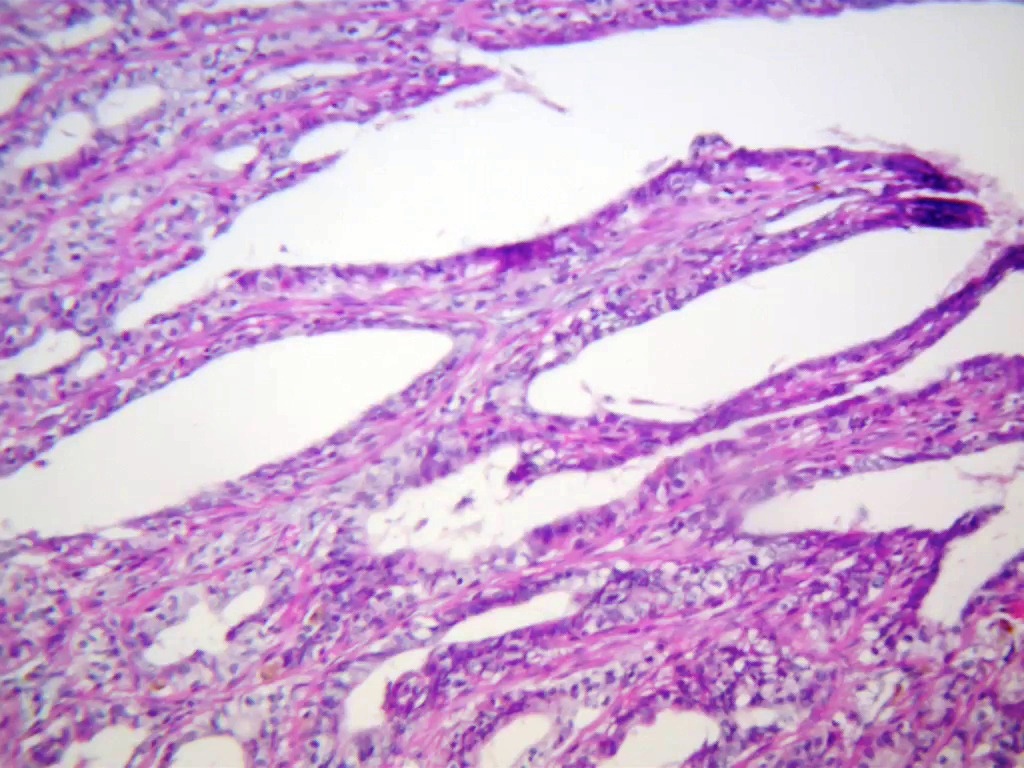

Supplement: Supplementary file 1 [file animals-13-01563-s001.zip › supplementary files/File S1 Canine Mammary Tumor Dataset/benign/Complex adenoma_208-12V2_FRM_065 (13).jpg]

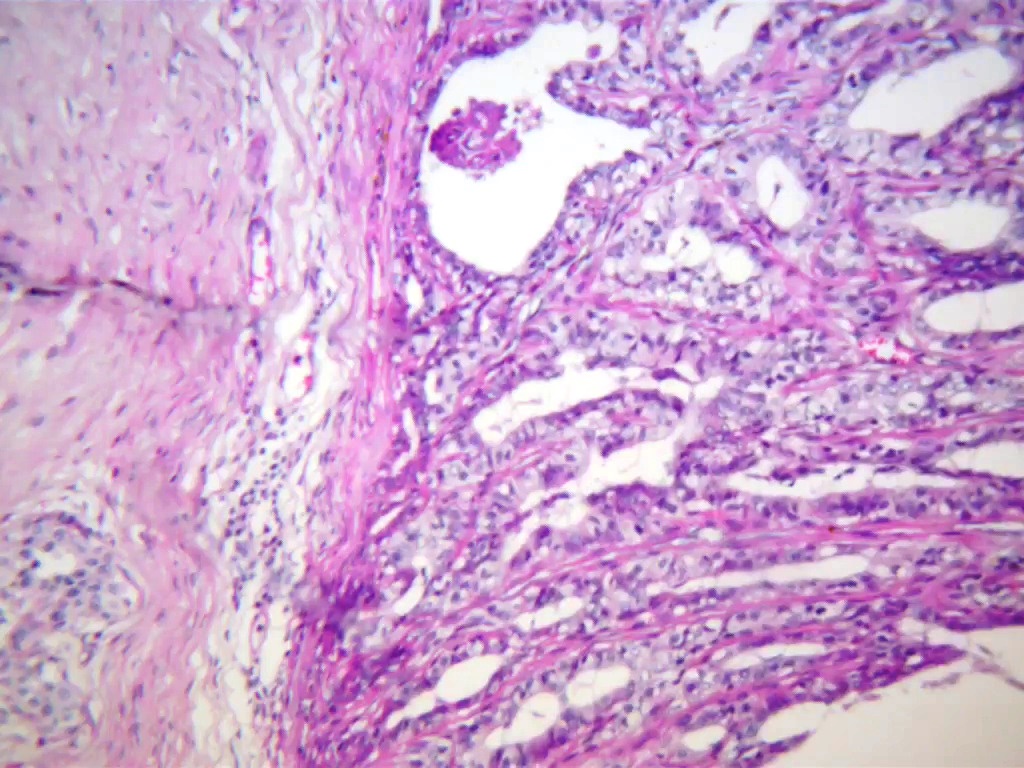

Supplement: Supplementary file 1 [file animals-13-01563-s001.zip › supplementary files/File S1 Canine Mammary Tumor Dataset/benign/Complex adenoma_208-12V2_FRM_065 (14).jpg]
