# Supplementary figures and images for: Canine Mammary Tumor Histopathological Image Classification via Computer-Aided Pathology: An Available Dataset for Imaging Analysis (part 1 of 2)
Source: Animals (Basel). 2023 May 6;13(9):1563. doi: 10.3390/ani13091563 (PMC10177203; doi:10.3390/ani13091563)

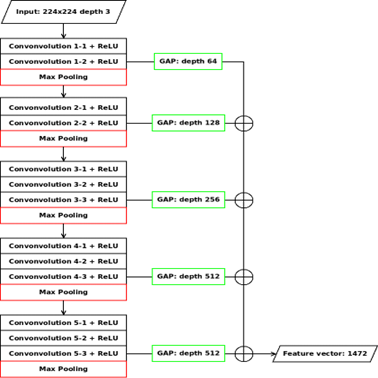

Supplement: Supplementary file 1 [file animals-13-01563-s001.zip › supplementary files/Figure S1 Depiction of the feature extraction process for VGG16 architecture..tif]

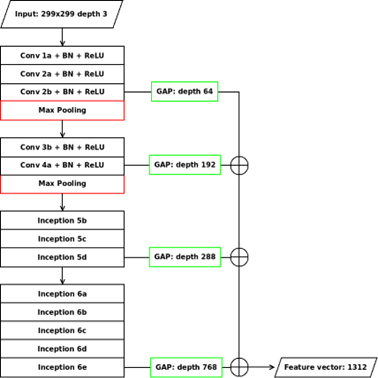

Supplement: Supplementary file 1 [file animals-13-01563-s001.zip › supplementary files/Figure S2 Depiction of the feature extraction process for Inception V3 architecture..tif]

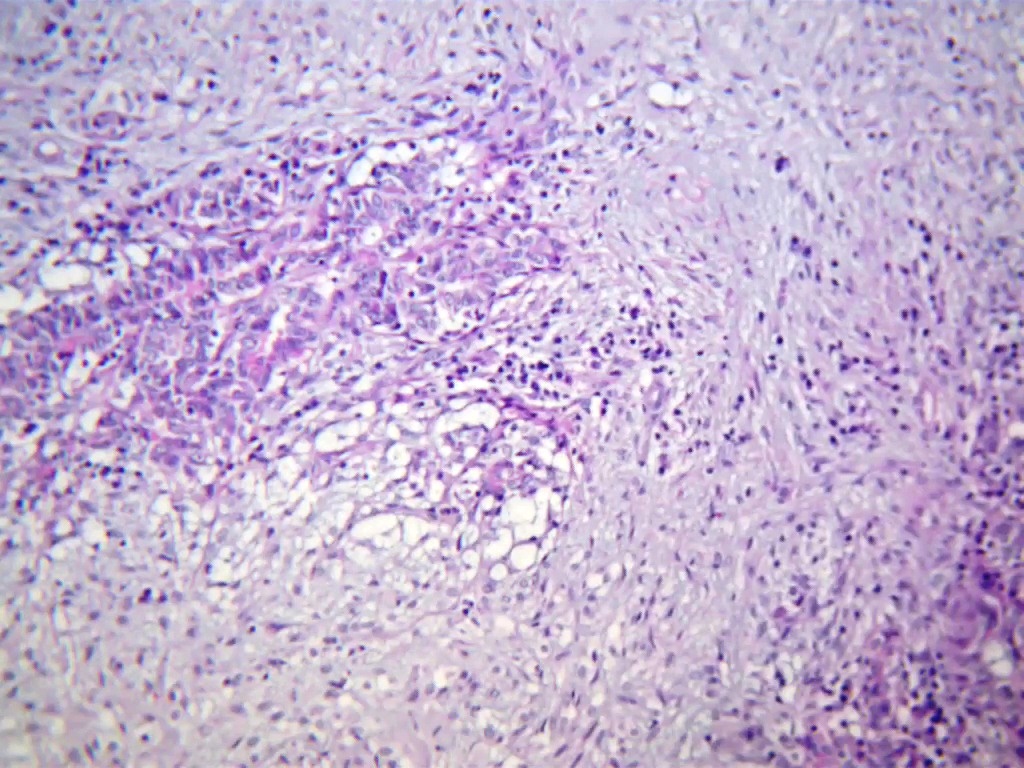

Supplement: Supplementary file 1 [file animals-13-01563-s001.zip › supplementary files/File S1 Canine Mammary Tumor Dataset/benign/Benign mixed tumor _465_V2_FRM_097 (66).jpg]

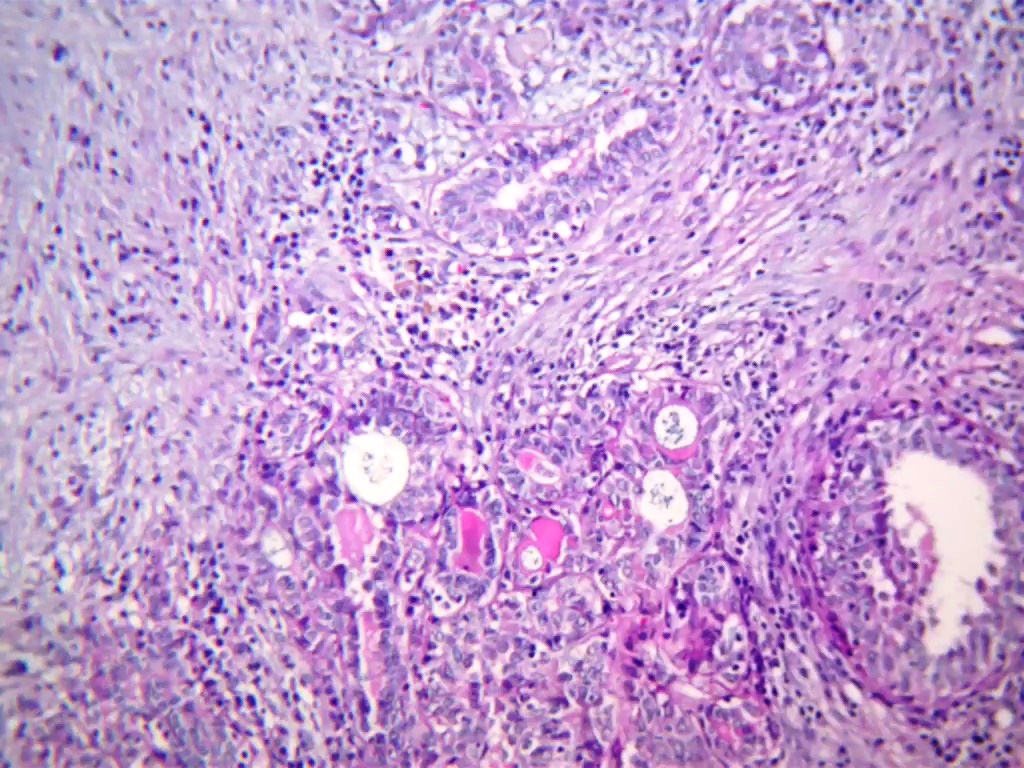

Supplement: Supplementary file 1 [file animals-13-01563-s001.zip › supplementary files/File S1 Canine Mammary Tumor Dataset/benign/Benign mixed tumor _465_V2_FRM_097 (67).jpg]

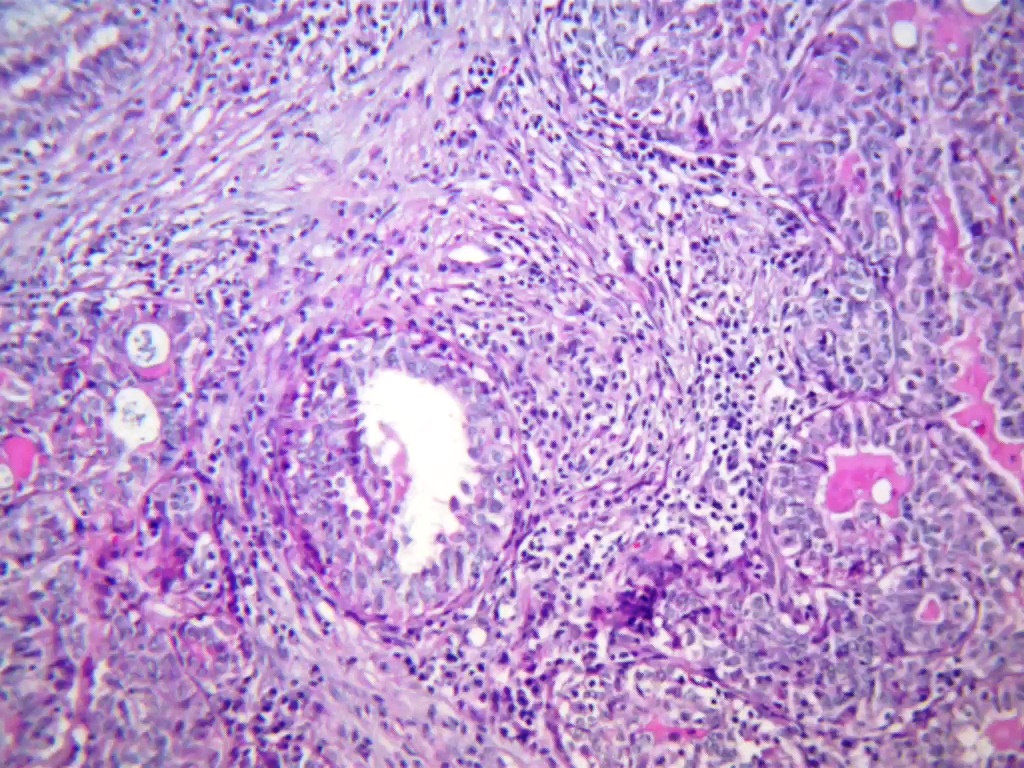

Supplement: Supplementary file 1 [file animals-13-01563-s001.zip › supplementary files/File S1 Canine Mammary Tumor Dataset/benign/Benign mixed tumor _465_V2_FRM_097 (68).jpg]

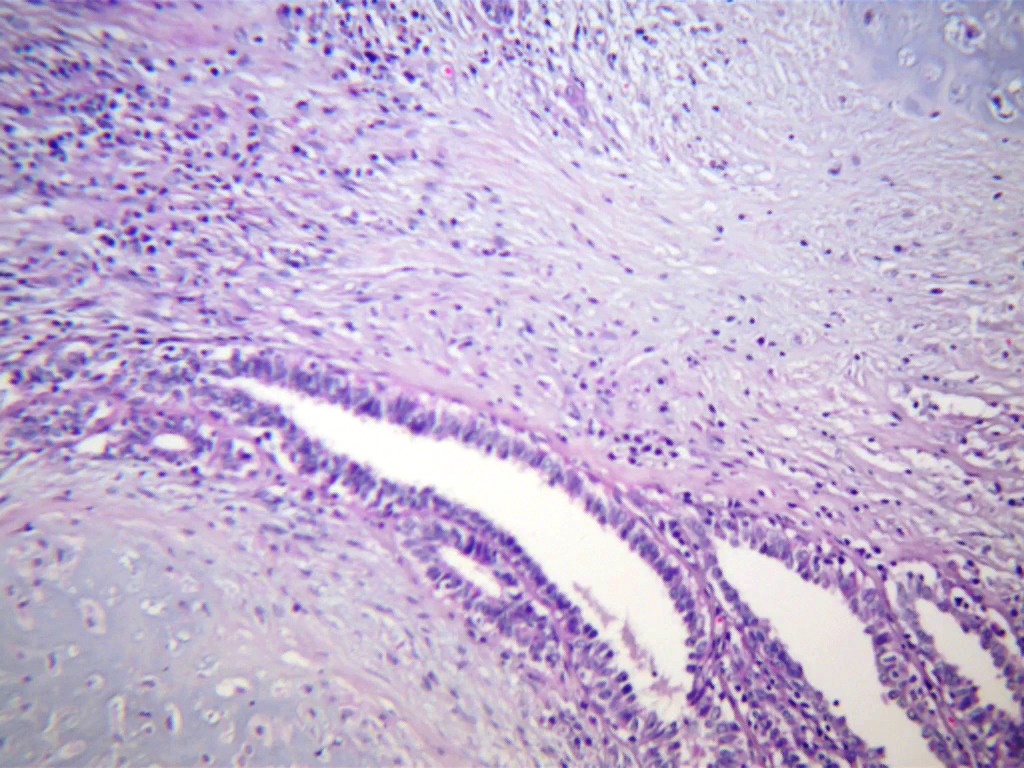

Supplement: Supplementary file 1 [file animals-13-01563-s001.zip › supplementary files/File S1 Canine Mammary Tumor Dataset/benign/Benign mixed tumor _465_V2_FRM_097 (69).jpg]

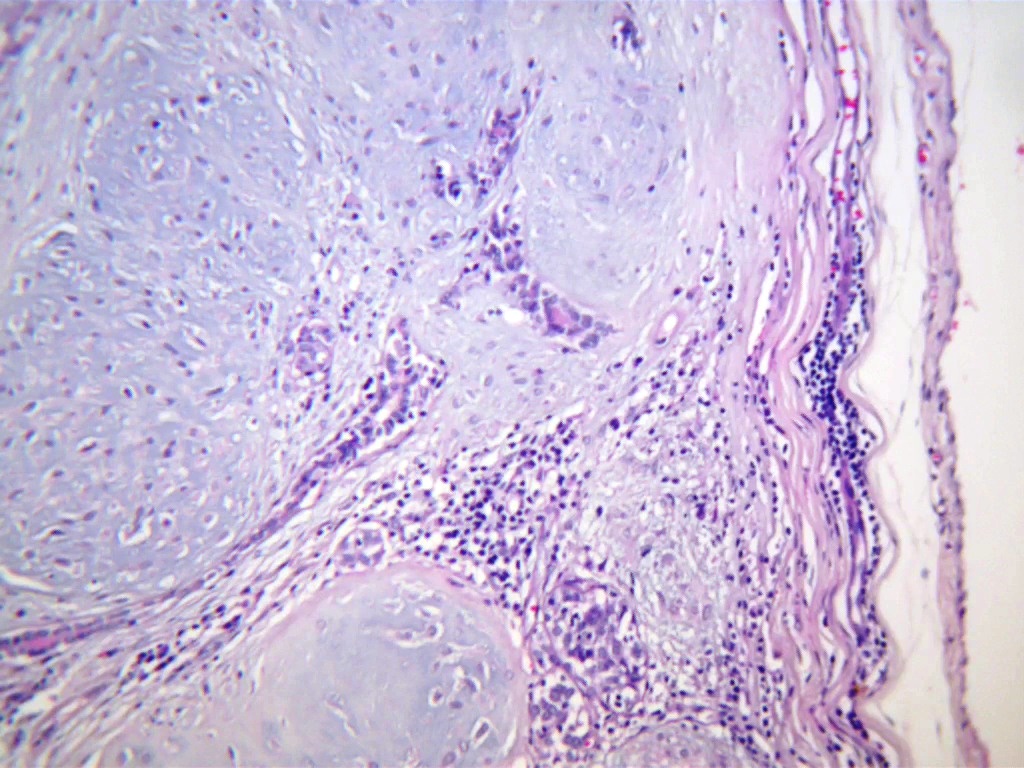

Supplement: Supplementary file 1 [file animals-13-01563-s001.zip › supplementary files/File S1 Canine Mammary Tumor Dataset/benign/Benign mixed tumor _465_V2_FRM_097 (70).jpg]

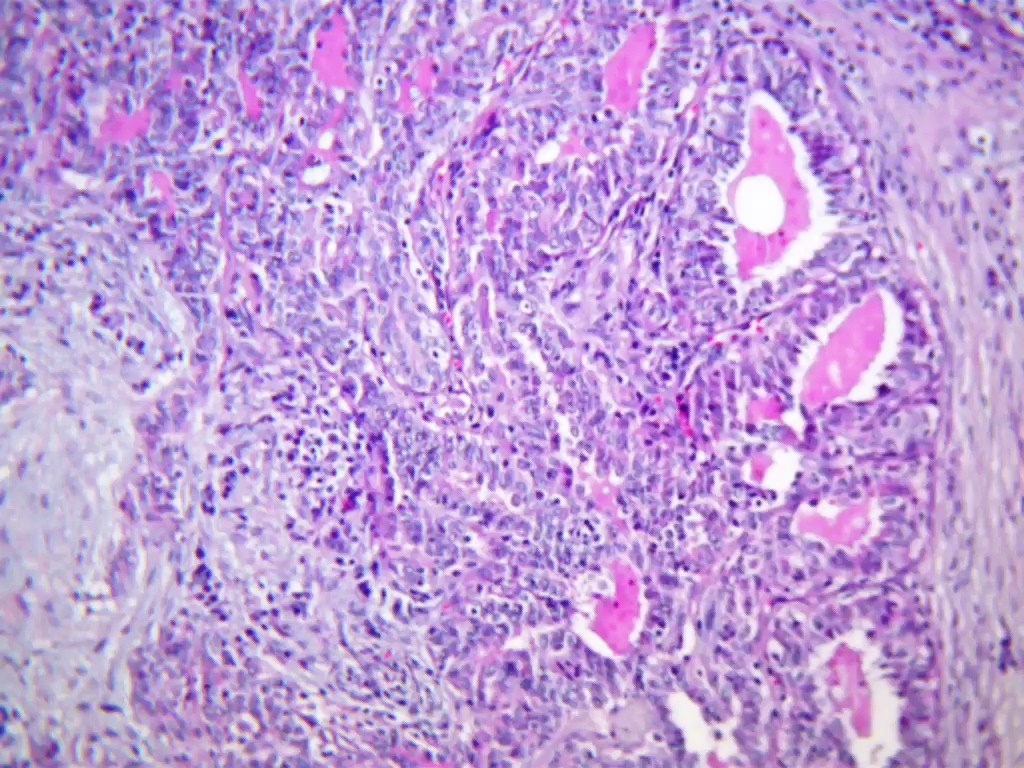

Supplement: Supplementary file 1 [file animals-13-01563-s001.zip › supplementary files/File S1 Canine Mammary Tumor Dataset/benign/Benign mixed tumor _465_V2_FRM_097 (71).jpg]

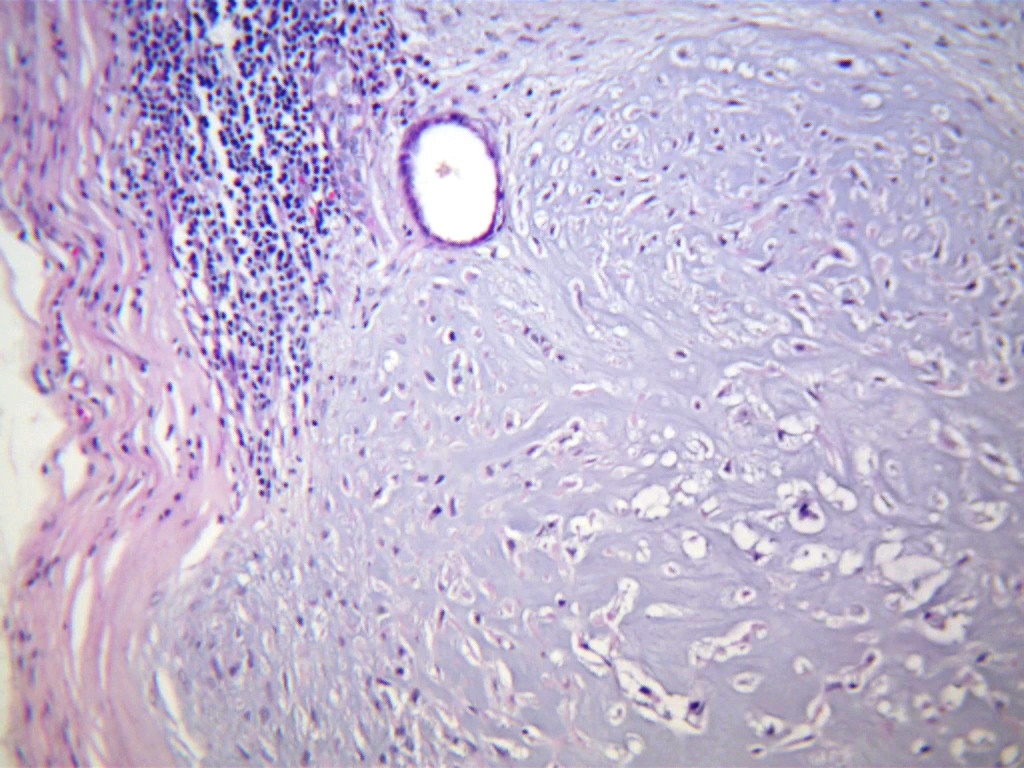

Supplement: Supplementary file 1 [file animals-13-01563-s001.zip › supplementary files/File S1 Canine Mammary Tumor Dataset/benign/Benign mixed tumor _465_V2_FRM_097 (72).jpg]

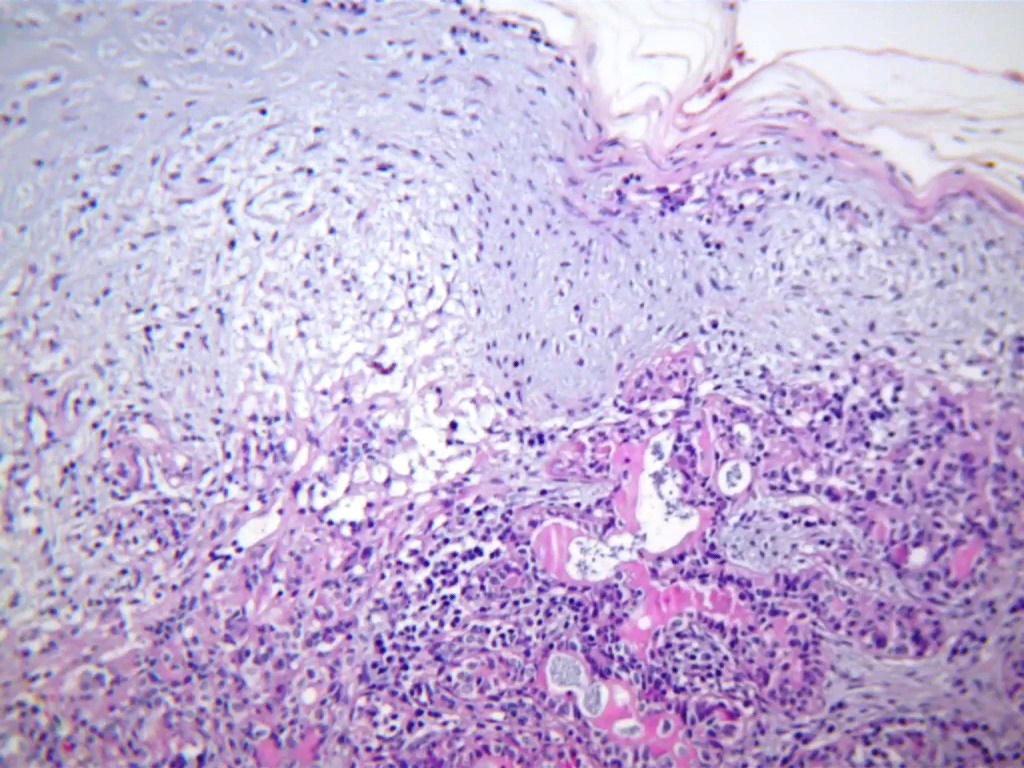

Supplement: Supplementary file 1 [file animals-13-01563-s001.zip › supplementary files/File S1 Canine Mammary Tumor Dataset/benign/Benign mixed tumor _465_V2_FRM_097 (73).jpg]

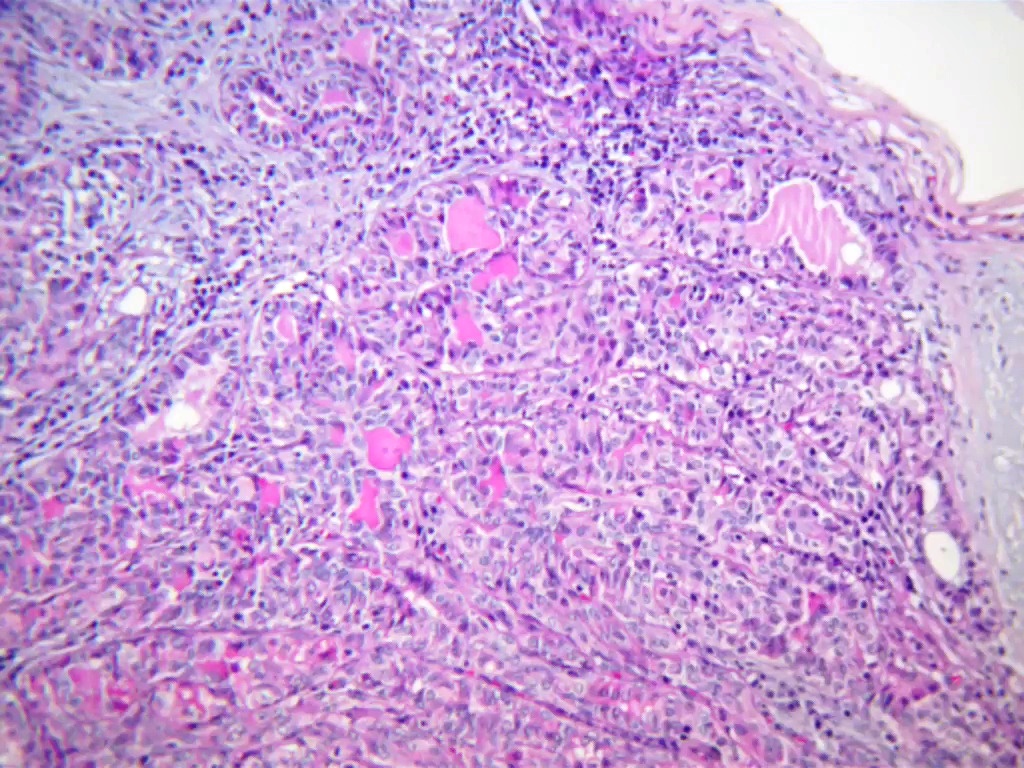

Supplement: Supplementary file 1 [file animals-13-01563-s001.zip › supplementary files/File S1 Canine Mammary Tumor Dataset/benign/Benign mixed tumor _465_V2_FRM_097 (74).jpg]

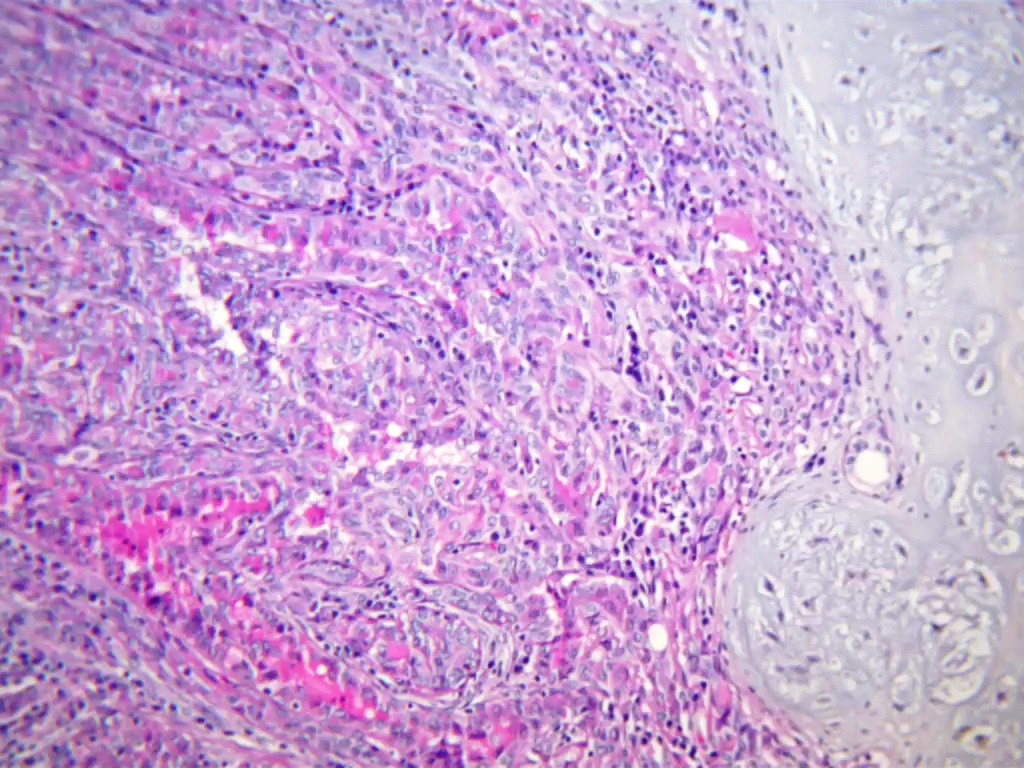

Supplement: Supplementary file 1 [file animals-13-01563-s001.zip › supplementary files/File S1 Canine Mammary Tumor Dataset/benign/Benign mixed tumor _465_V2_FRM_097 (75).jpg]

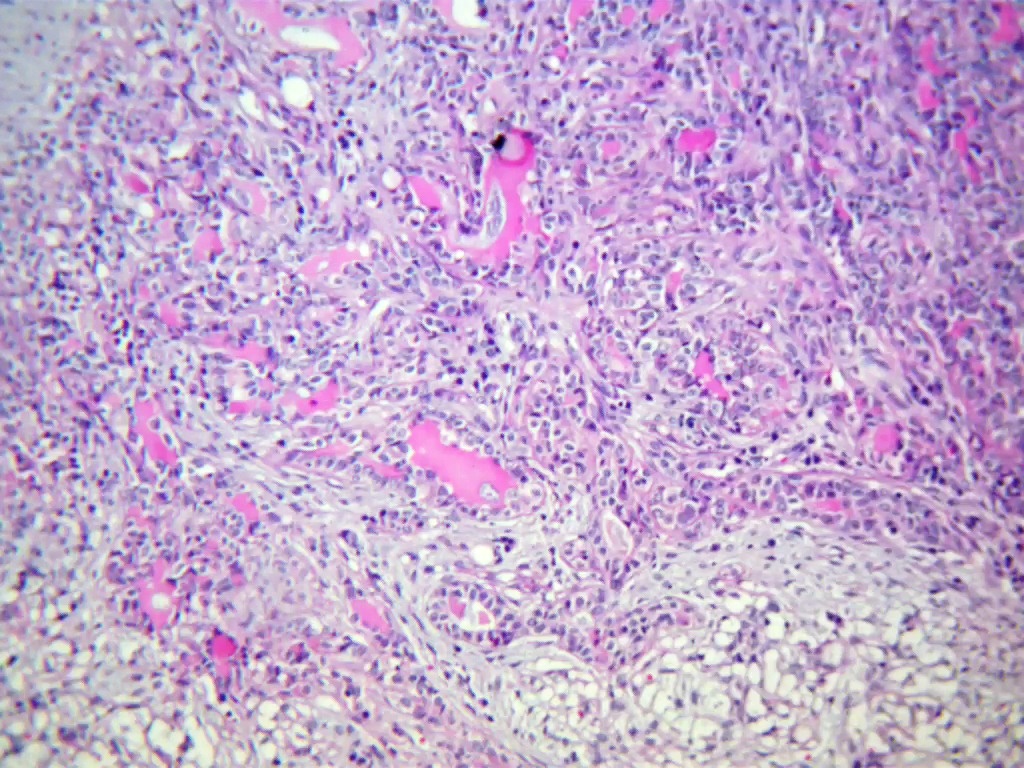

Supplement: Supplementary file 1 [file animals-13-01563-s001.zip › supplementary files/File S1 Canine Mammary Tumor Dataset/benign/Benign mixed tumor _465_V2_FRM_097 (76).jpg]

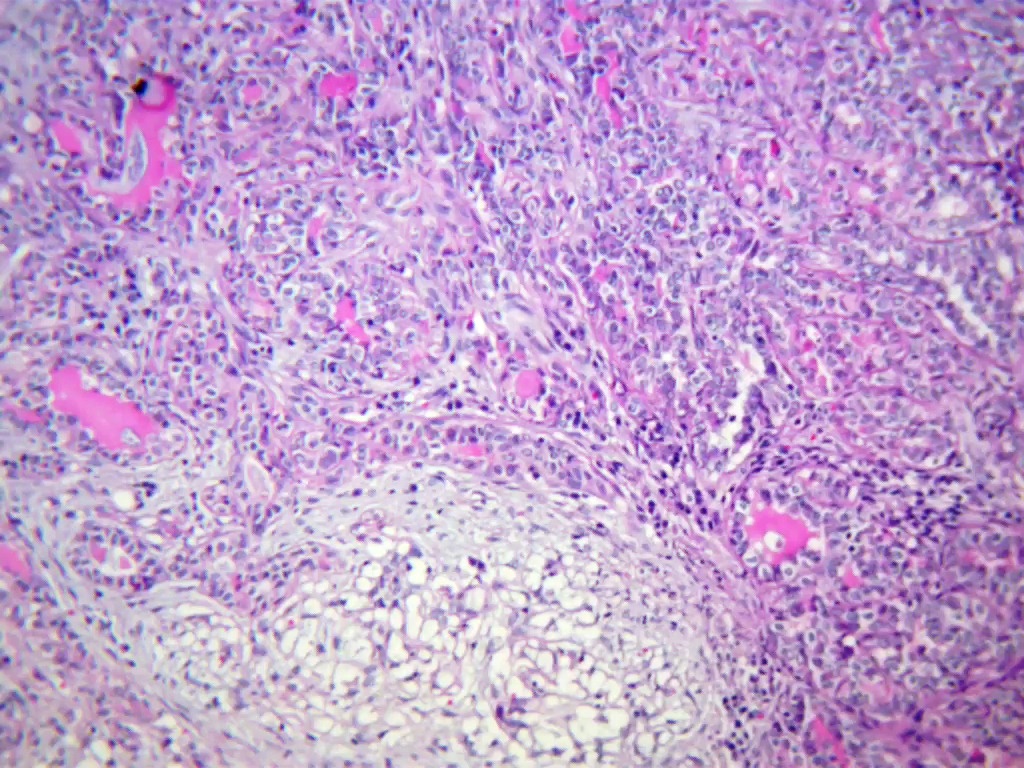

Supplement: Supplementary file 1 [file animals-13-01563-s001.zip › supplementary files/File S1 Canine Mammary Tumor Dataset/benign/Benign mixed tumor _465_V2_FRM_097 (77).jpg]

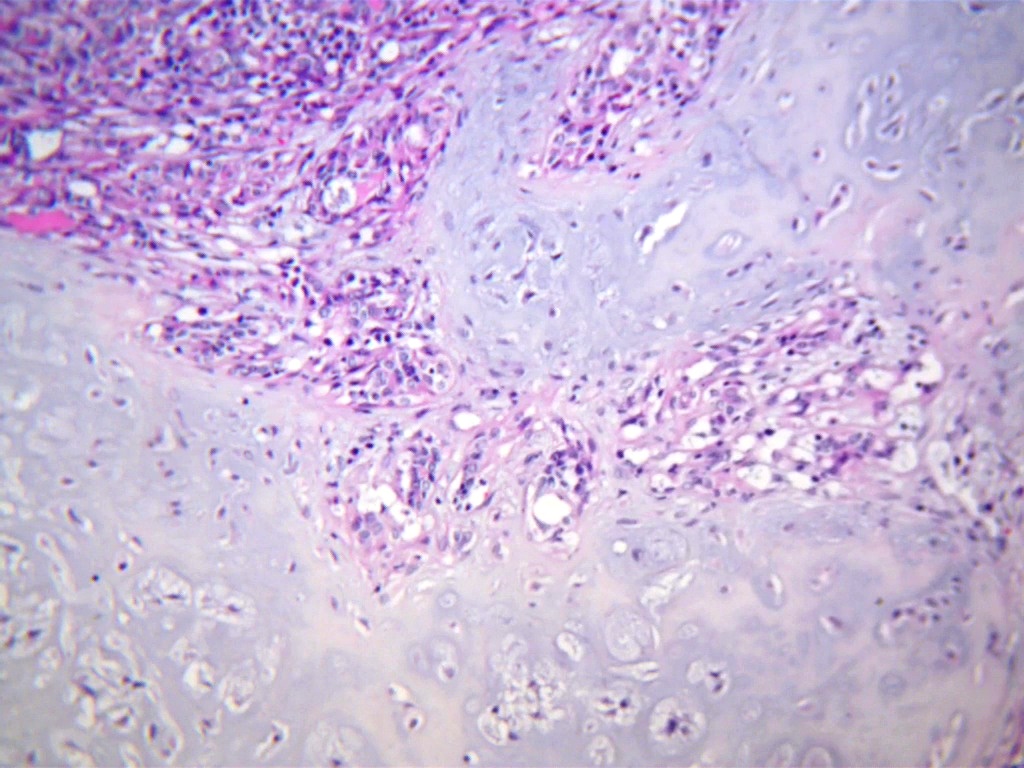

Supplement: Supplementary file 1 [file animals-13-01563-s001.zip › supplementary files/File S1 Canine Mammary Tumor Dataset/benign/Benign mixed tumor _465_V2_FRM_097 (78).jpg]

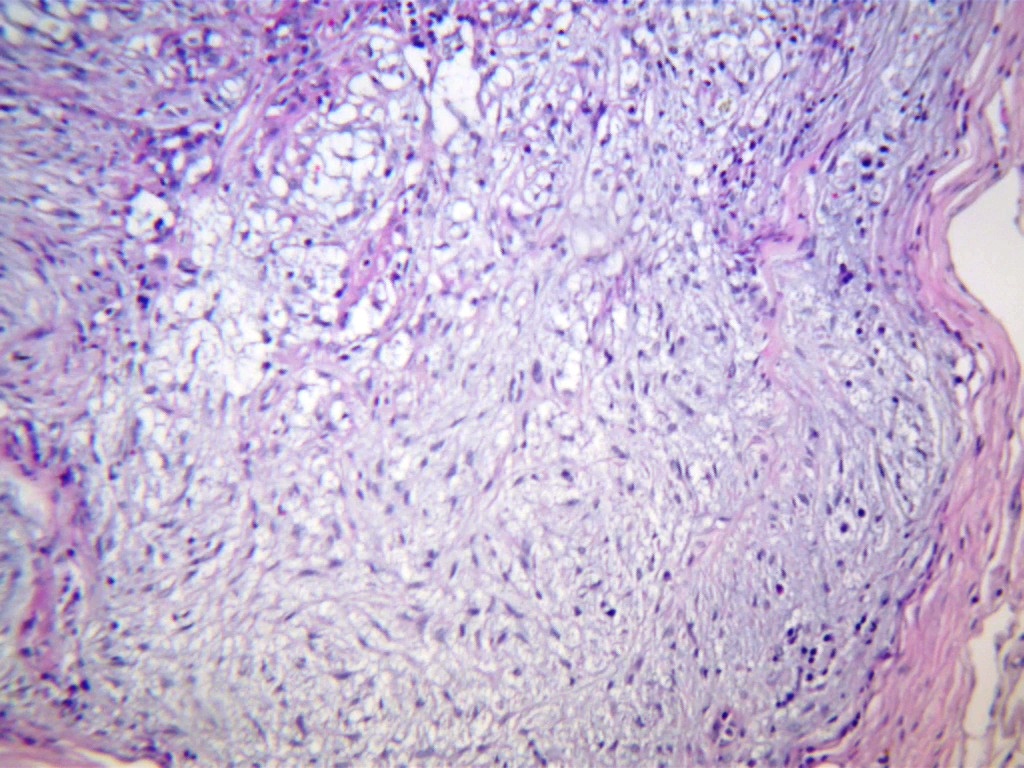

Supplement: Supplementary file 1 [file animals-13-01563-s001.zip › supplementary files/File S1 Canine Mammary Tumor Dataset/benign/Benign mixed tumor _465_V2_FRM_097 (79).jpg]

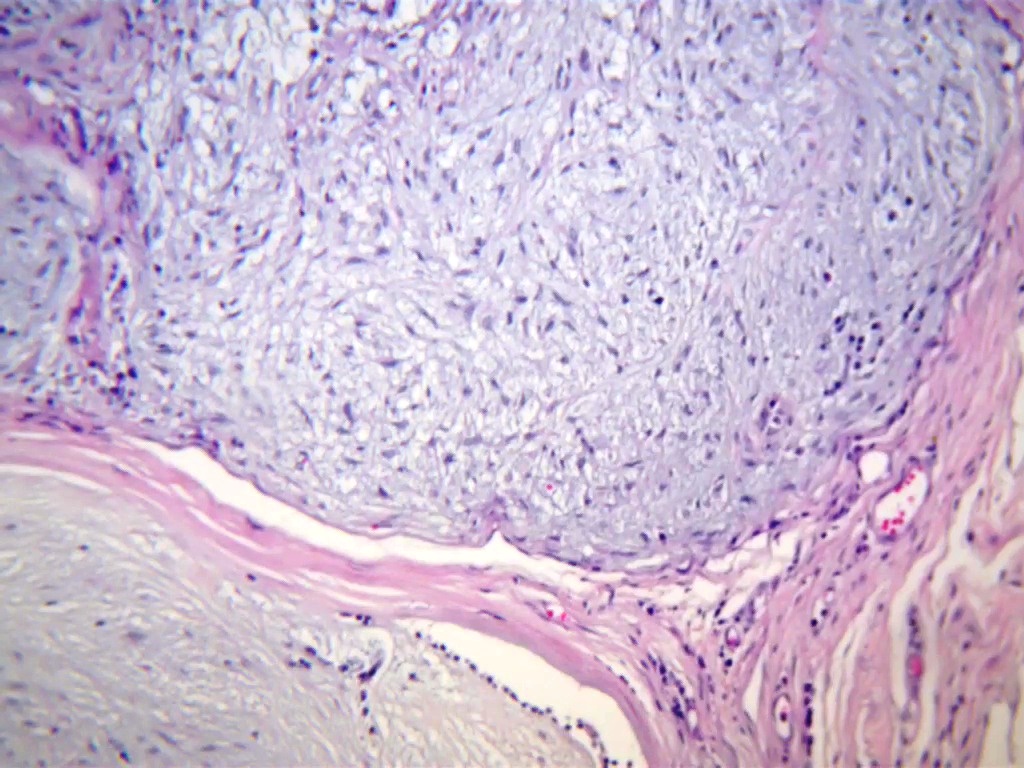

Supplement: Supplementary file 1 [file animals-13-01563-s001.zip › supplementary files/File S1 Canine Mammary Tumor Dataset/benign/Benign mixed tumor _465_V2_FRM_097 (80).jpg]

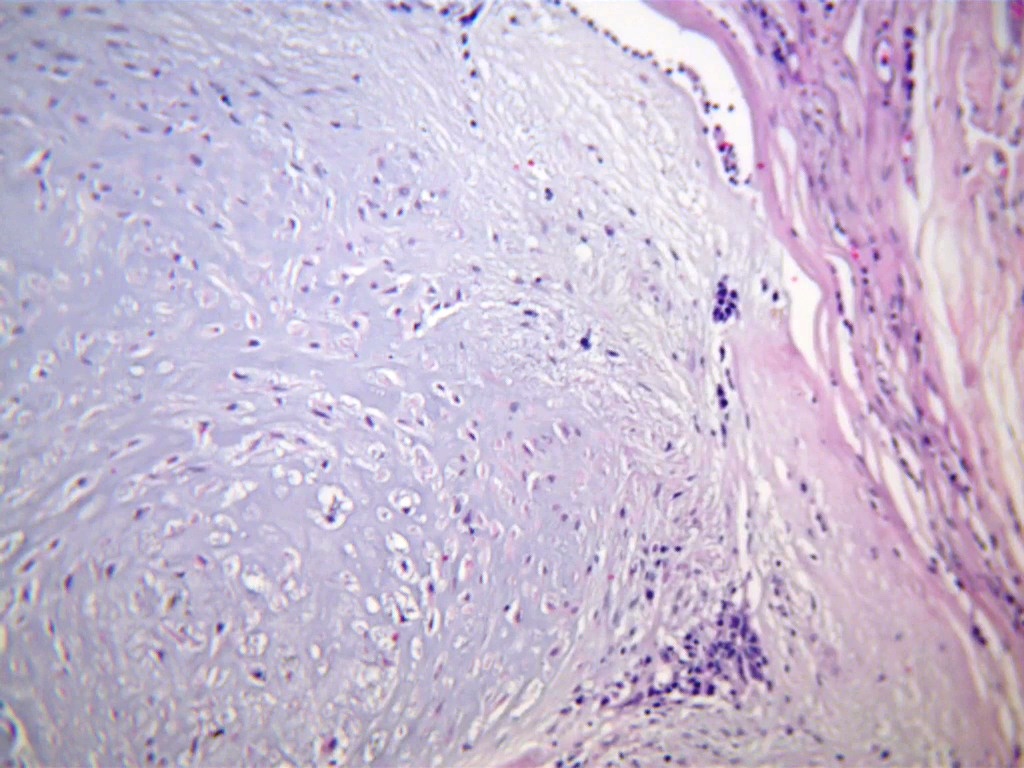

Supplement: Supplementary file 1 [file animals-13-01563-s001.zip › supplementary files/File S1 Canine Mammary Tumor Dataset/benign/Benign mixed tumor _465_V2_FRM_097 (81).jpg]

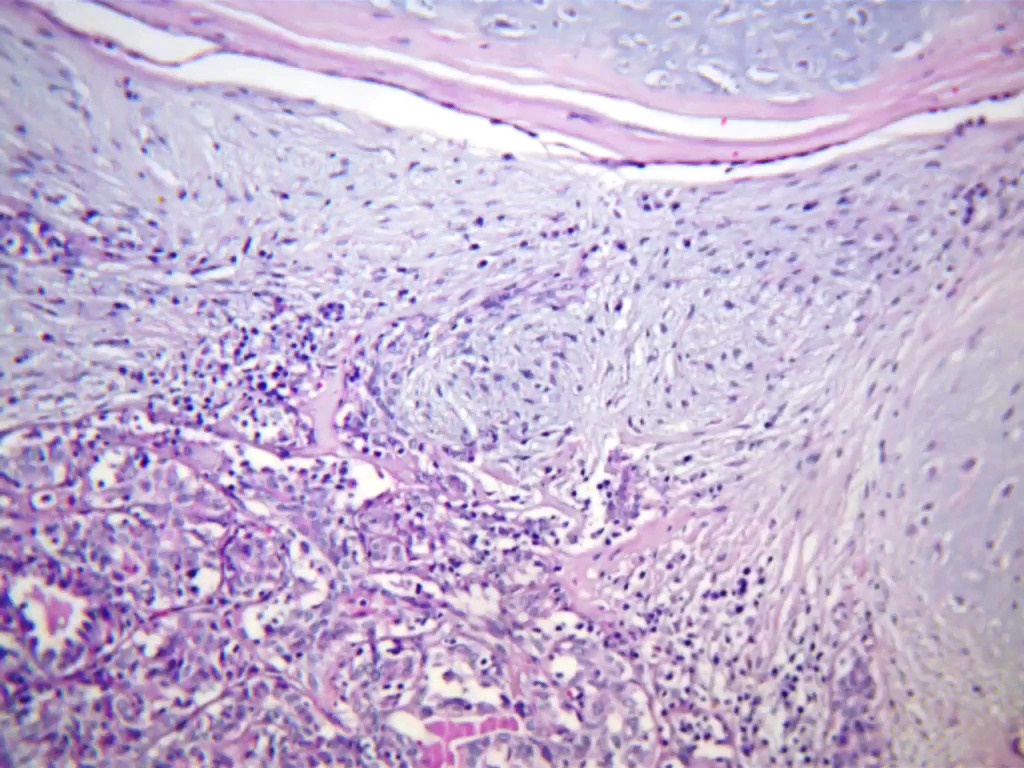

Supplement: Supplementary file 1 [file animals-13-01563-s001.zip › supplementary files/File S1 Canine Mammary Tumor Dataset/benign/Benign mixed tumor _465_V2_FRM_097 (82).jpg]

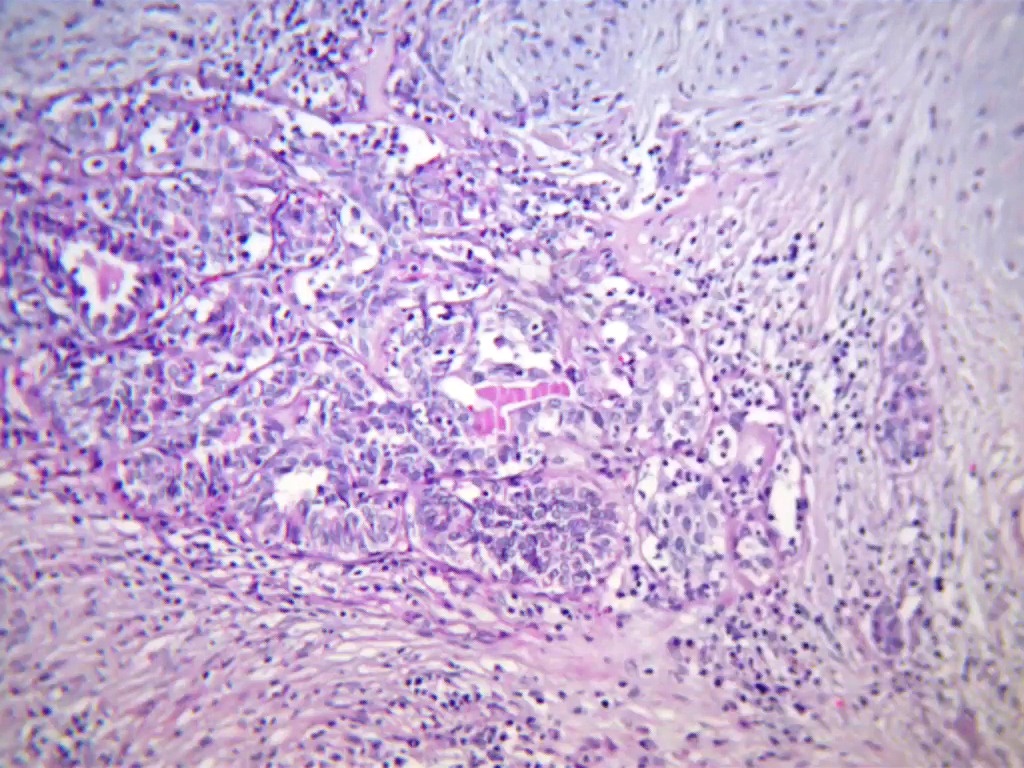

Supplement: Supplementary file 1 [file animals-13-01563-s001.zip › supplementary files/File S1 Canine Mammary Tumor Dataset/benign/Benign mixed tumor _465_V2_FRM_097 (83).jpg]

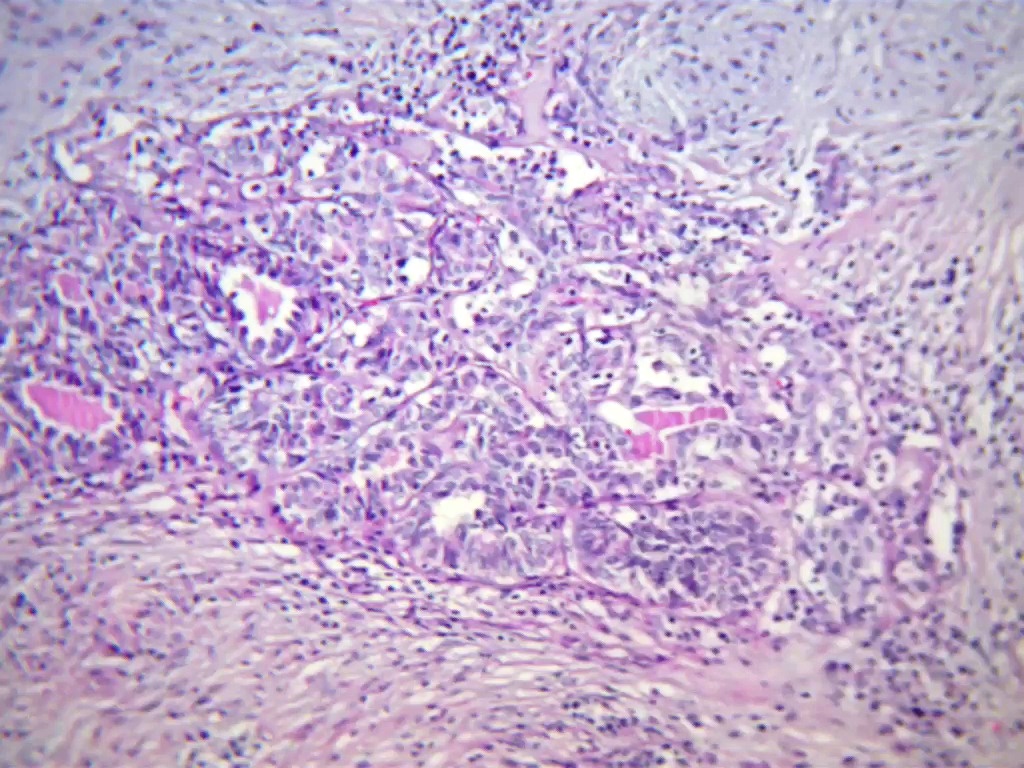

Supplement: Supplementary file 1 [file animals-13-01563-s001.zip › supplementary files/File S1 Canine Mammary Tumor Dataset/benign/Benign mixed tumor _465_V2_FRM_097 (84).jpg]

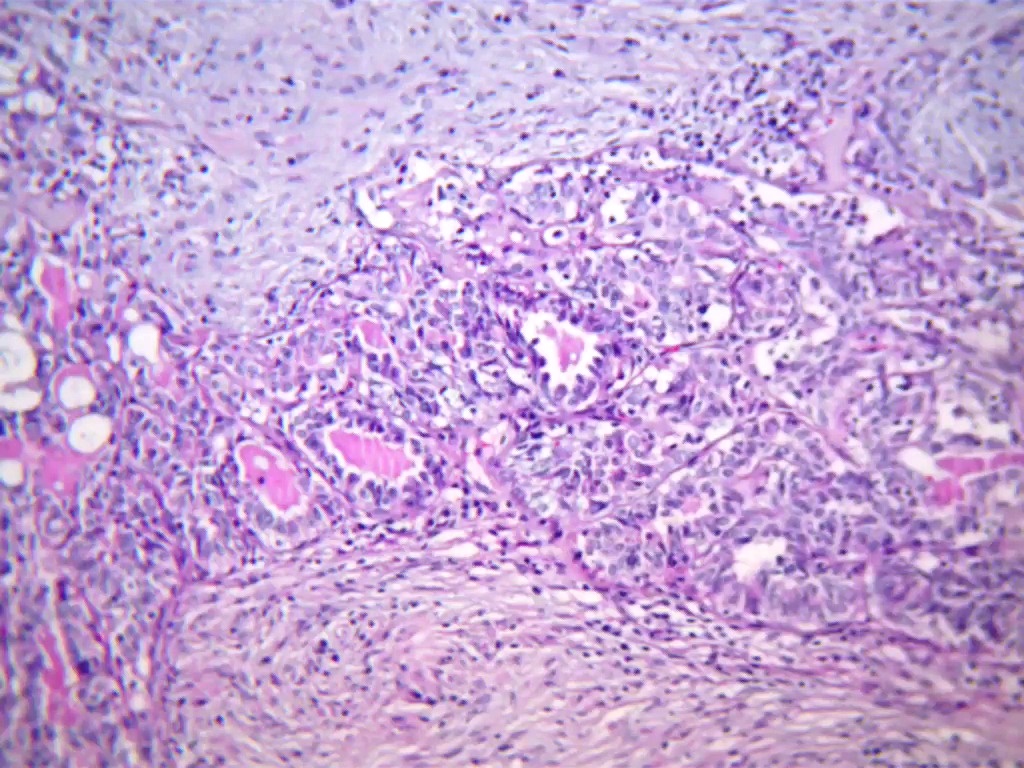

Supplement: Supplementary file 1 [file animals-13-01563-s001.zip › supplementary files/File S1 Canine Mammary Tumor Dataset/benign/Benign mixed tumor _465_V2_FRM_097 (85).jpg]

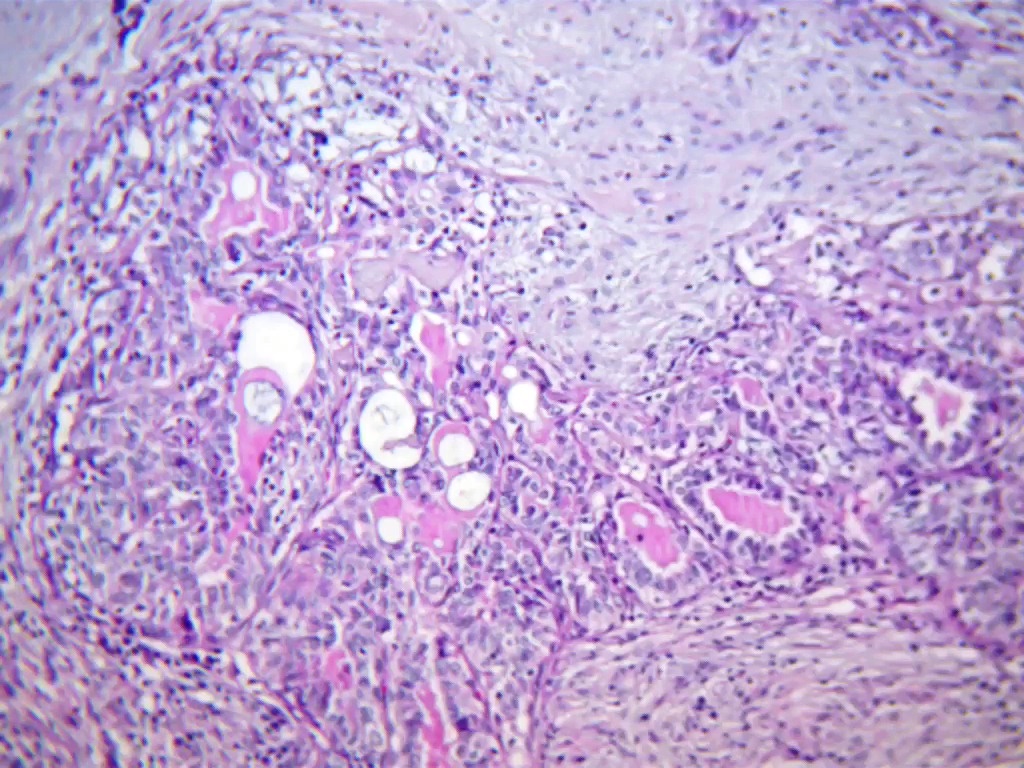

Supplement: Supplementary file 1 [file animals-13-01563-s001.zip › supplementary files/File S1 Canine Mammary Tumor Dataset/benign/Benign mixed tumor _465_V2_FRM_097 (86).jpg]

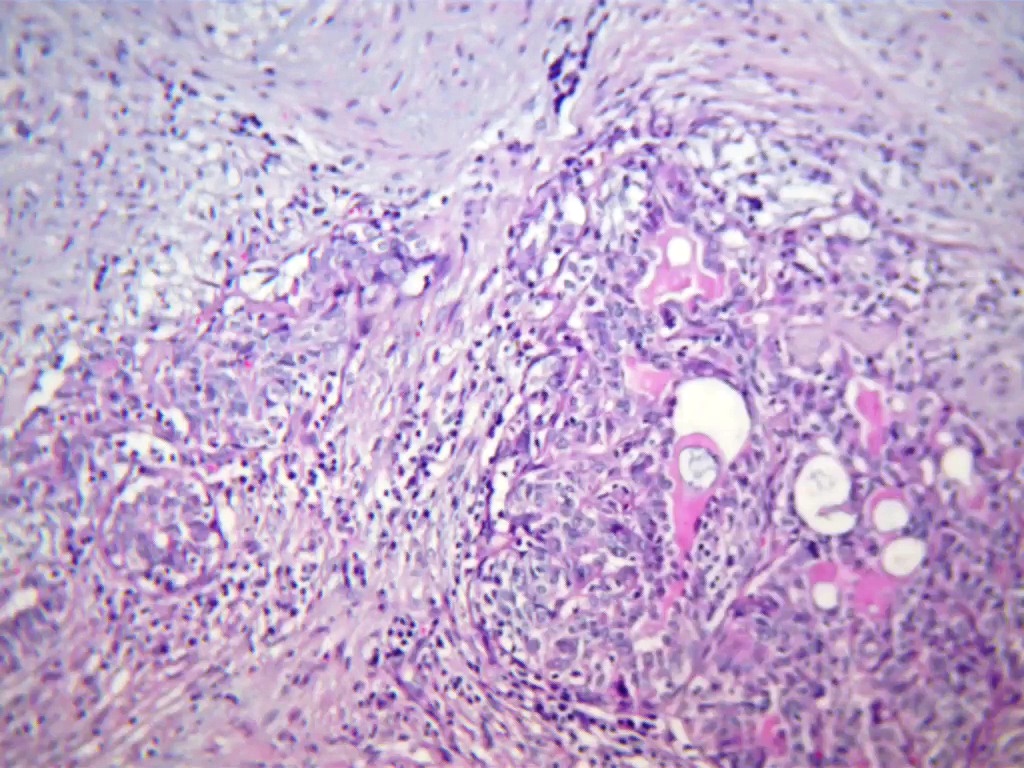

Supplement: Supplementary file 1 [file animals-13-01563-s001.zip › supplementary files/File S1 Canine Mammary Tumor Dataset/benign/Benign mixed tumor _465_V2_FRM_097 (87).jpg]

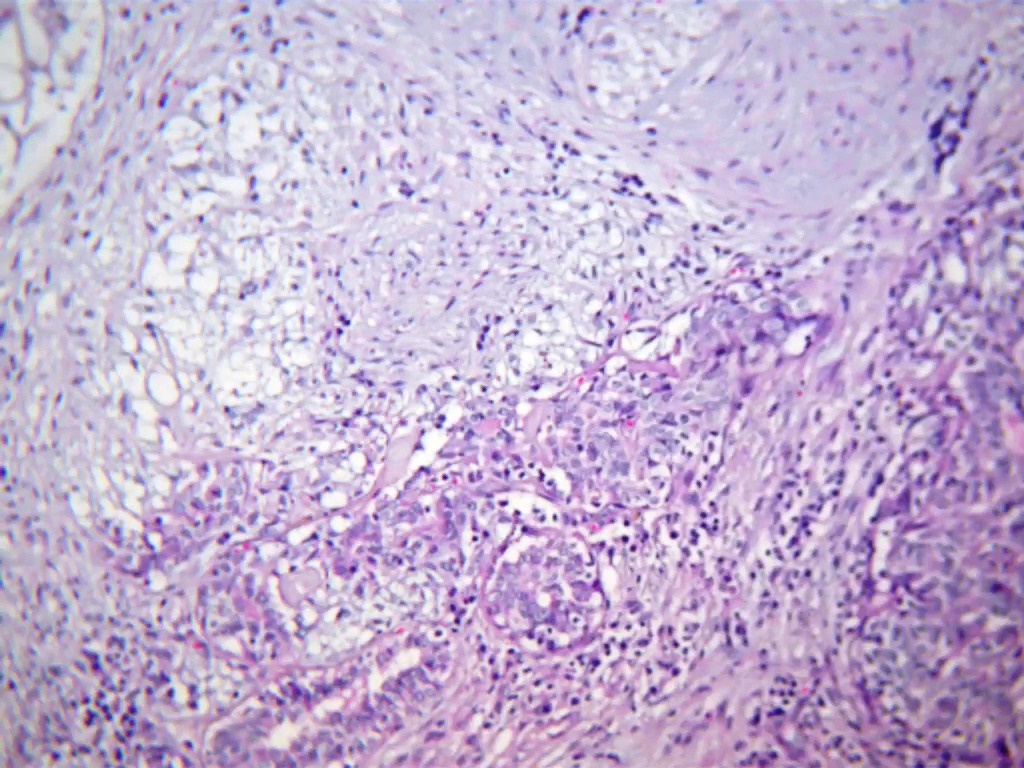

Supplement: Supplementary file 1 [file animals-13-01563-s001.zip › supplementary files/File S1 Canine Mammary Tumor Dataset/benign/Benign mixed tumor _465_V2_FRM_097 (88).jpg]

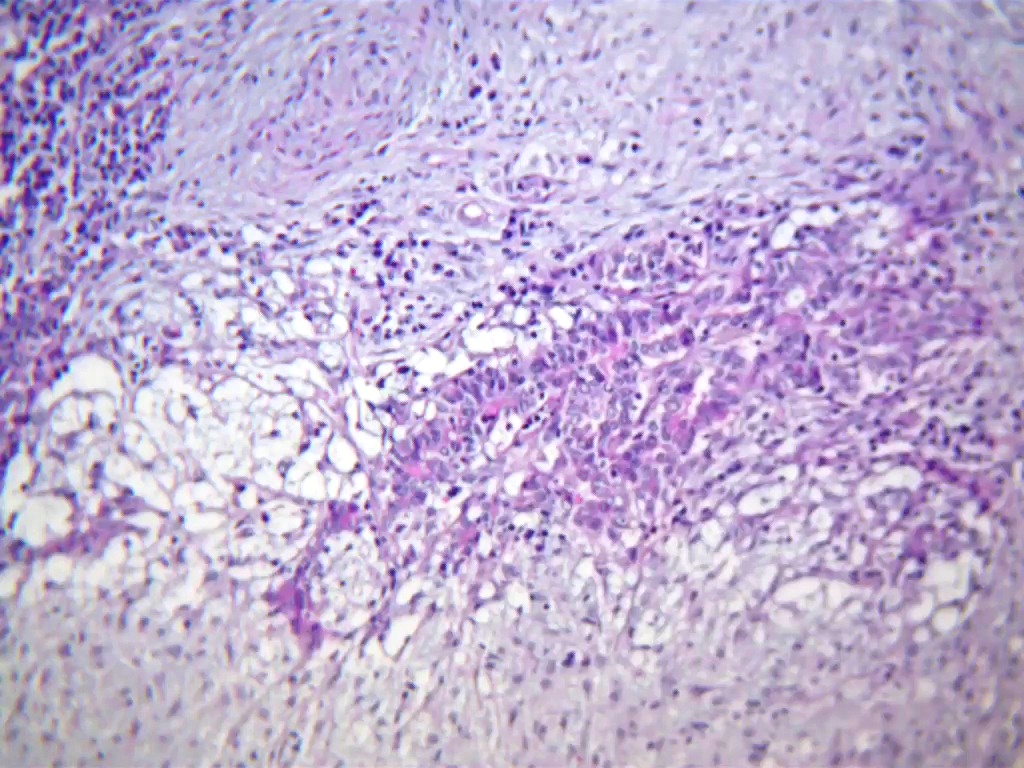

Supplement: Supplementary file 1 [file animals-13-01563-s001.zip › supplementary files/File S1 Canine Mammary Tumor Dataset/benign/Benign mixed tumor _465_V2_FRM_097 (89).jpg]

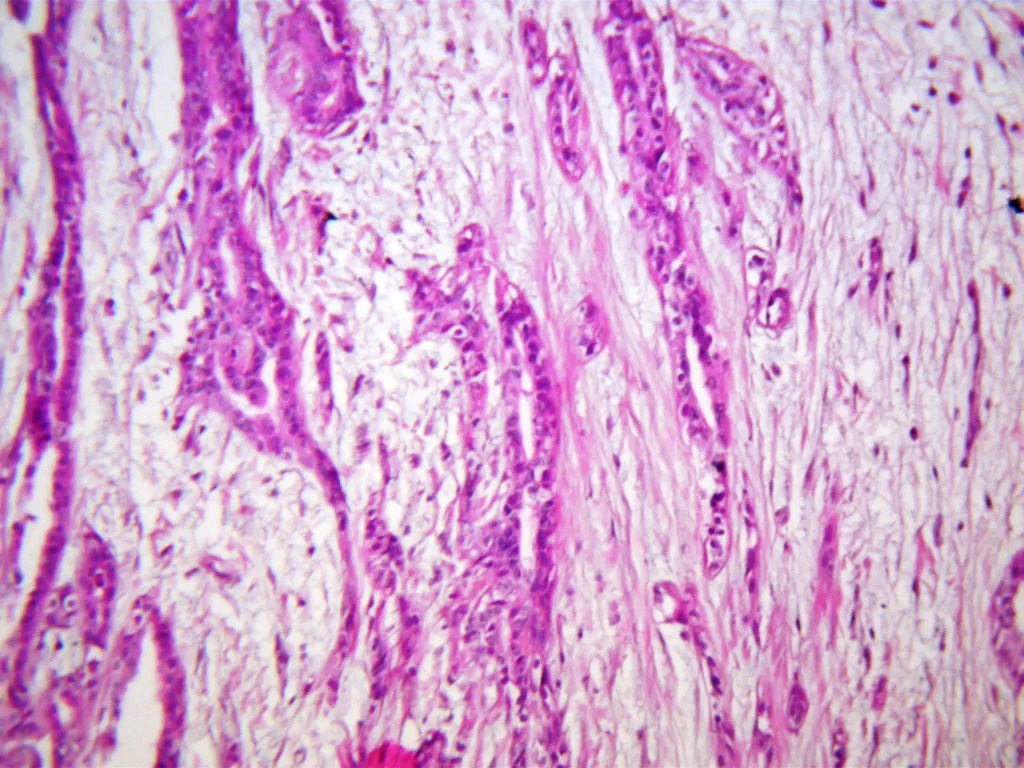

Supplement: Supplementary file 1 [file animals-13-01563-s001.zip › supplementary files/File S1 Canine Mammary Tumor Dataset/benign/Benign mixed tumor_28-12V2_FRM_006 (1).jpg]

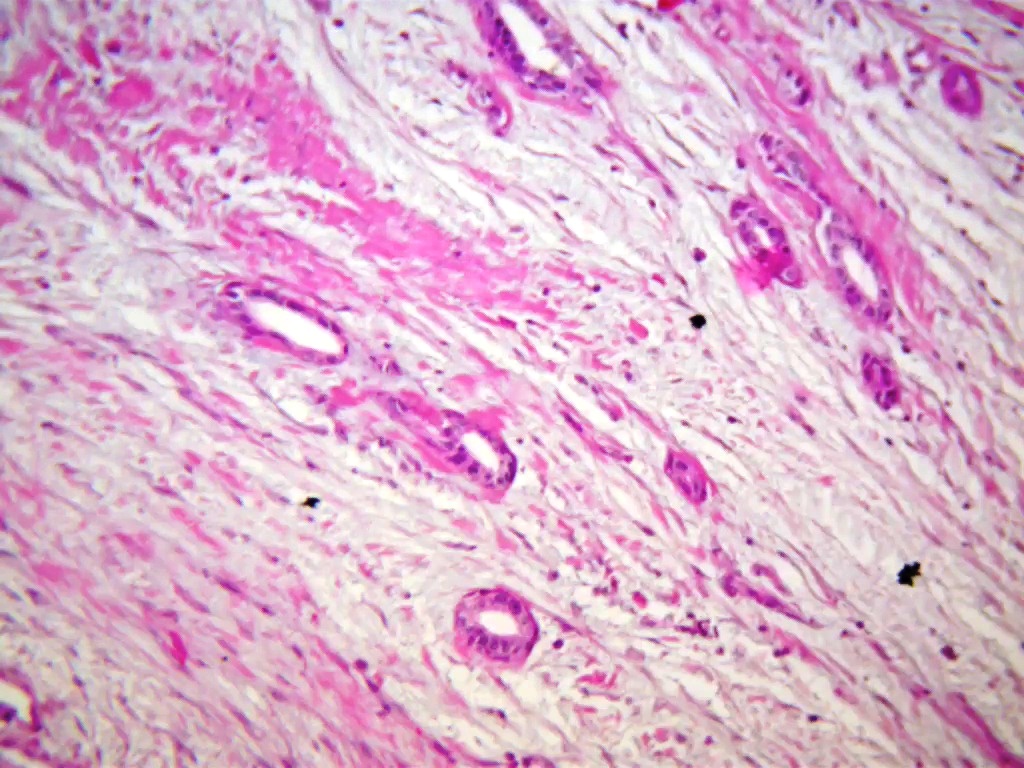

Supplement: Supplementary file 1 [file animals-13-01563-s001.zip › supplementary files/File S1 Canine Mammary Tumor Dataset/benign/Benign mixed tumor_28-12V2_FRM_006 (10).jpg]

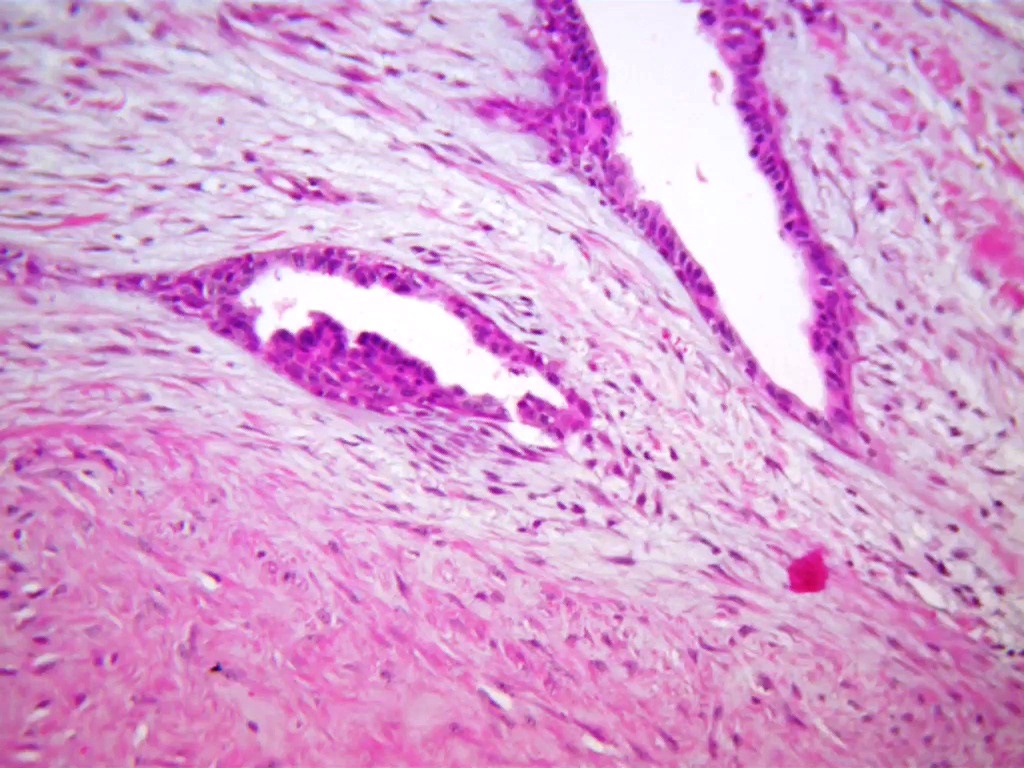

Supplement: Supplementary file 1 [file animals-13-01563-s001.zip › supplementary files/File S1 Canine Mammary Tumor Dataset/benign/Benign mixed tumor_28-12V2_FRM_006 (11).jpg]

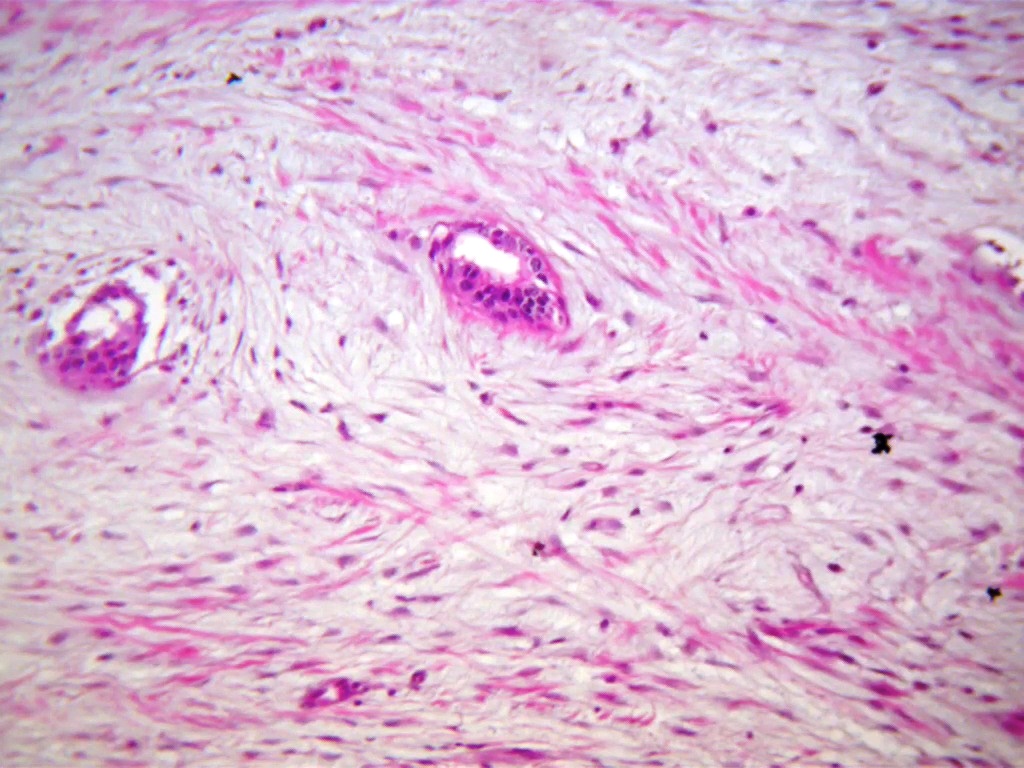

Supplement: Supplementary file 1 [file animals-13-01563-s001.zip › supplementary files/File S1 Canine Mammary Tumor Dataset/benign/Benign mixed tumor_28-12V2_FRM_006 (12).jpg]

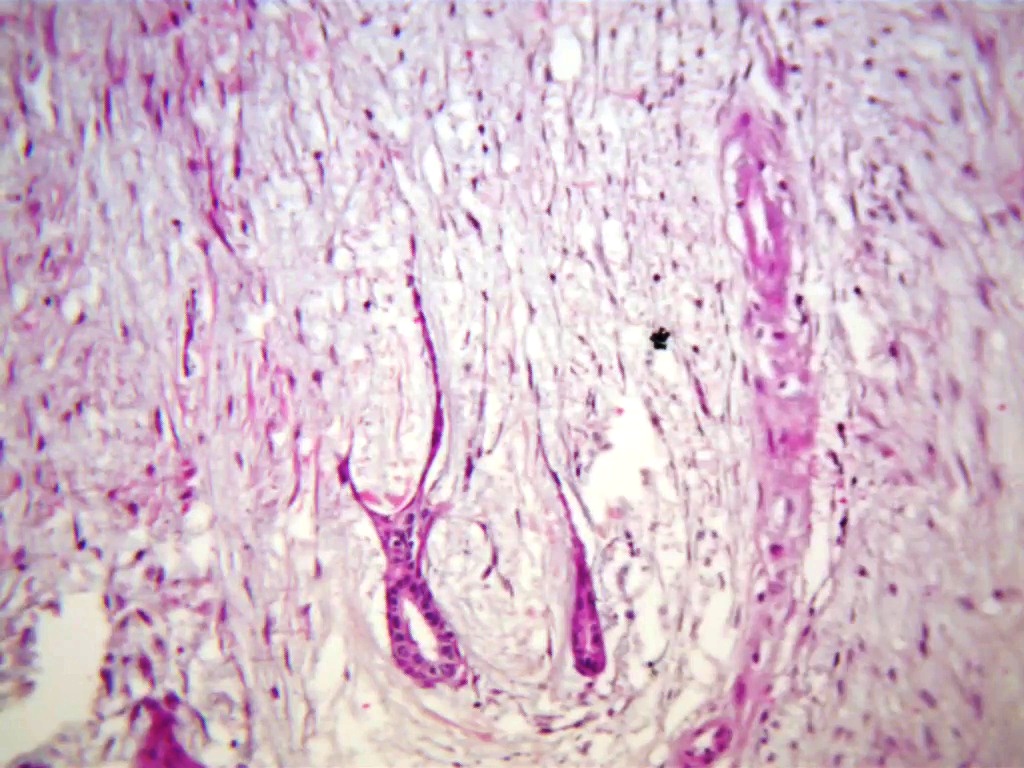

Supplement: Supplementary file 1 [file animals-13-01563-s001.zip › supplementary files/File S1 Canine Mammary Tumor Dataset/benign/Benign mixed tumor_28-12V2_FRM_006 (13).jpg]

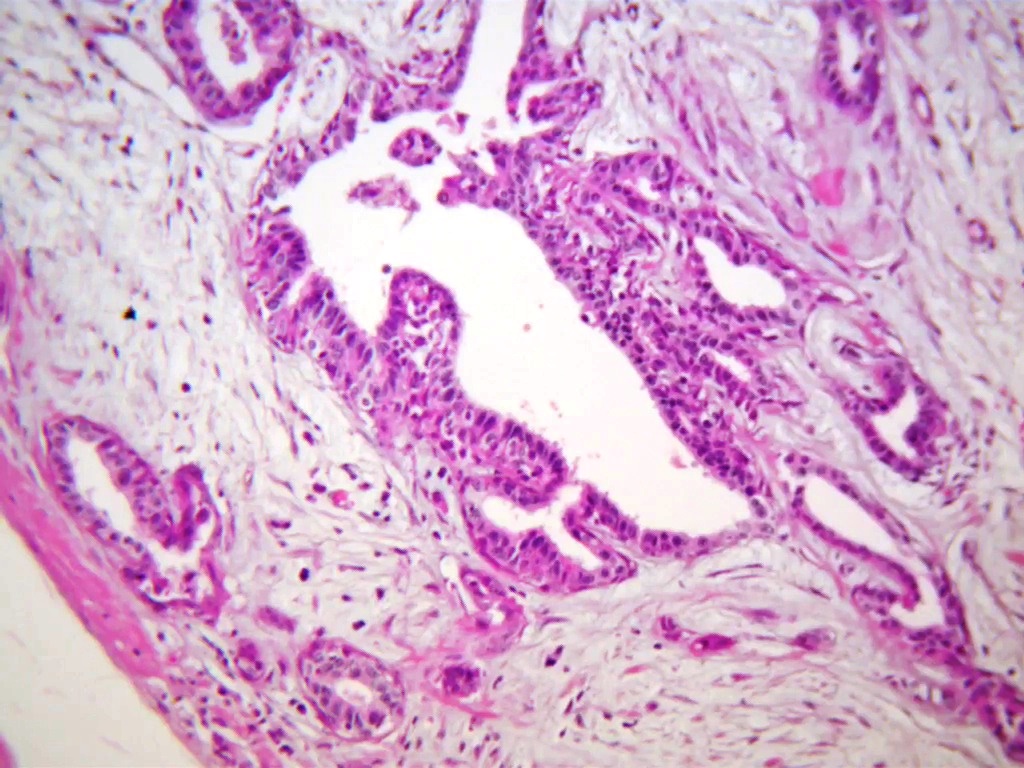

Supplement: Supplementary file 1 [file animals-13-01563-s001.zip › supplementary files/File S1 Canine Mammary Tumor Dataset/benign/Benign mixed tumor_28-12V2_FRM_006 (14).jpg]

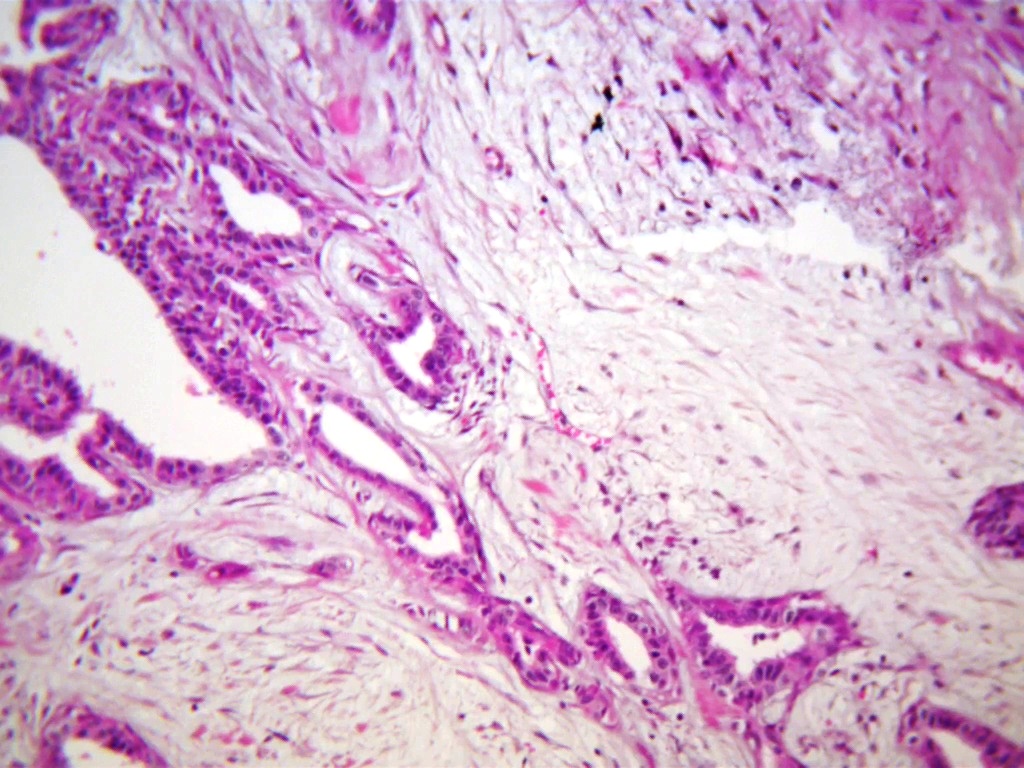

Supplement: Supplementary file 1 [file animals-13-01563-s001.zip › supplementary files/File S1 Canine Mammary Tumor Dataset/benign/Benign mixed tumor_28-12V2_FRM_006 (15).jpg]

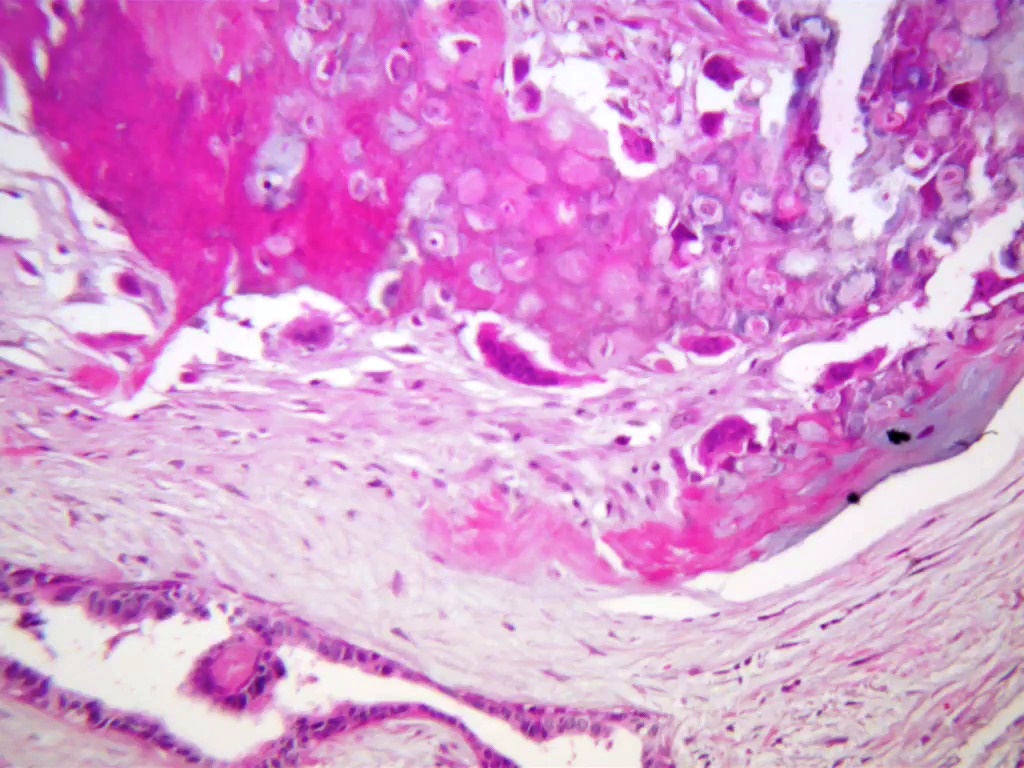

Supplement: Supplementary file 1 [file animals-13-01563-s001.zip › supplementary files/File S1 Canine Mammary Tumor Dataset/benign/Benign mixed tumor_28-12V2_FRM_006 (16).jpg]

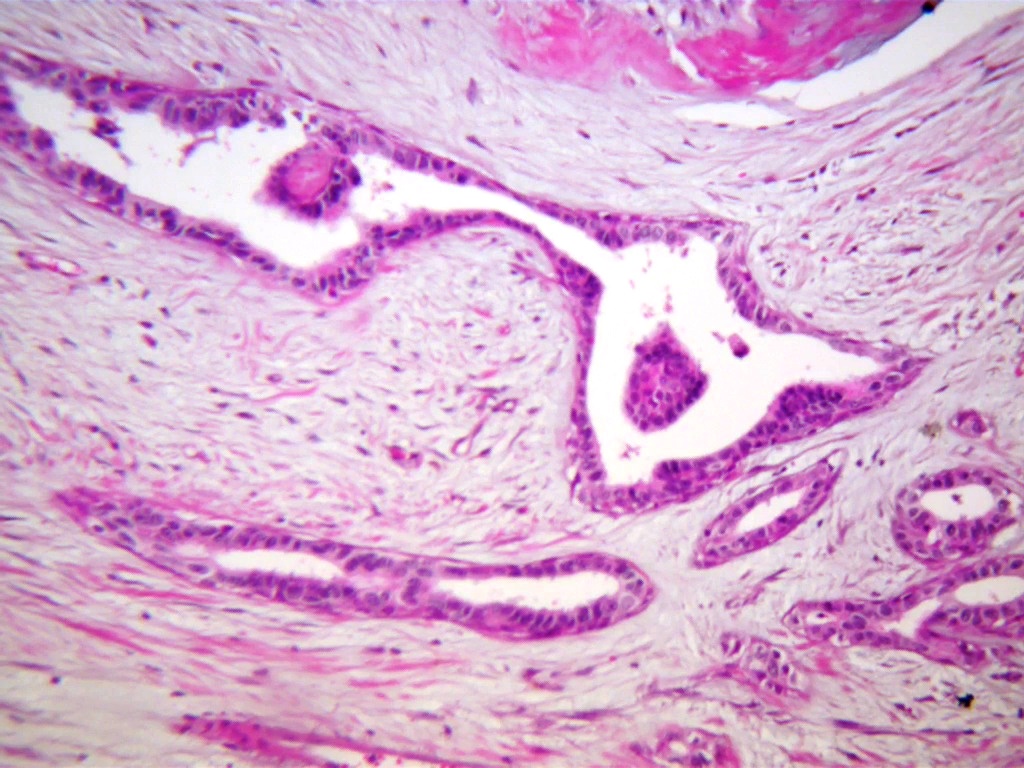

Supplement: Supplementary file 1 [file animals-13-01563-s001.zip › supplementary files/File S1 Canine Mammary Tumor Dataset/benign/Benign mixed tumor_28-12V2_FRM_006 (17).jpg]

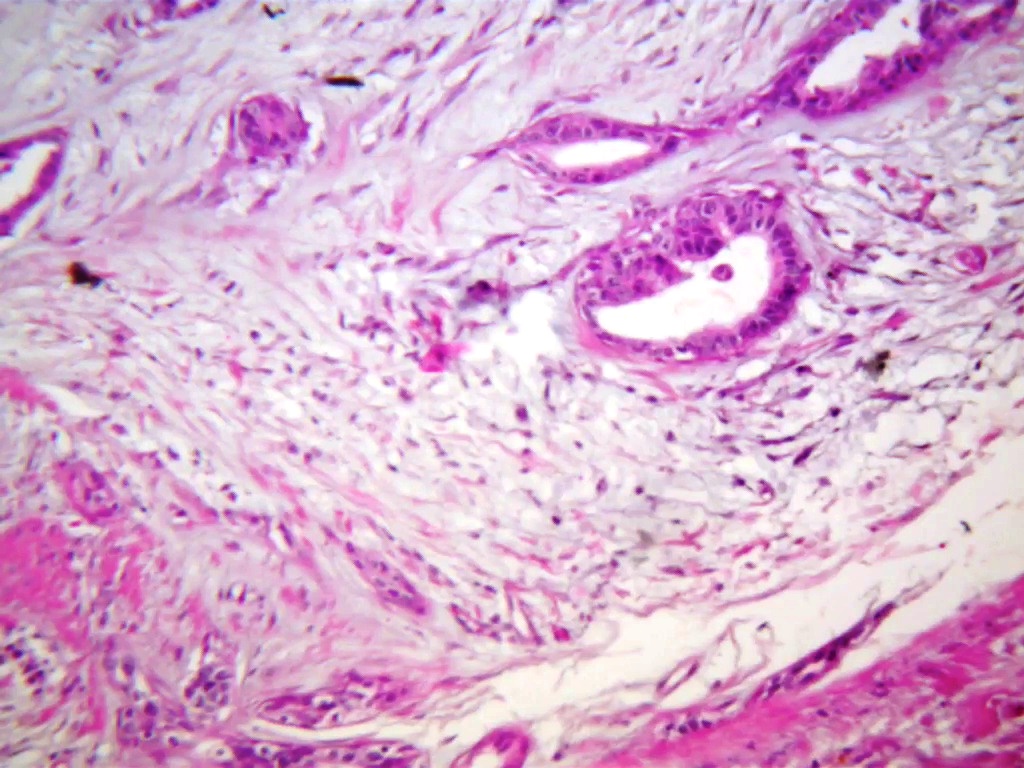

Supplement: Supplementary file 1 [file animals-13-01563-s001.zip › supplementary files/File S1 Canine Mammary Tumor Dataset/benign/Benign mixed tumor_28-12V2_FRM_006 (18).jpg]

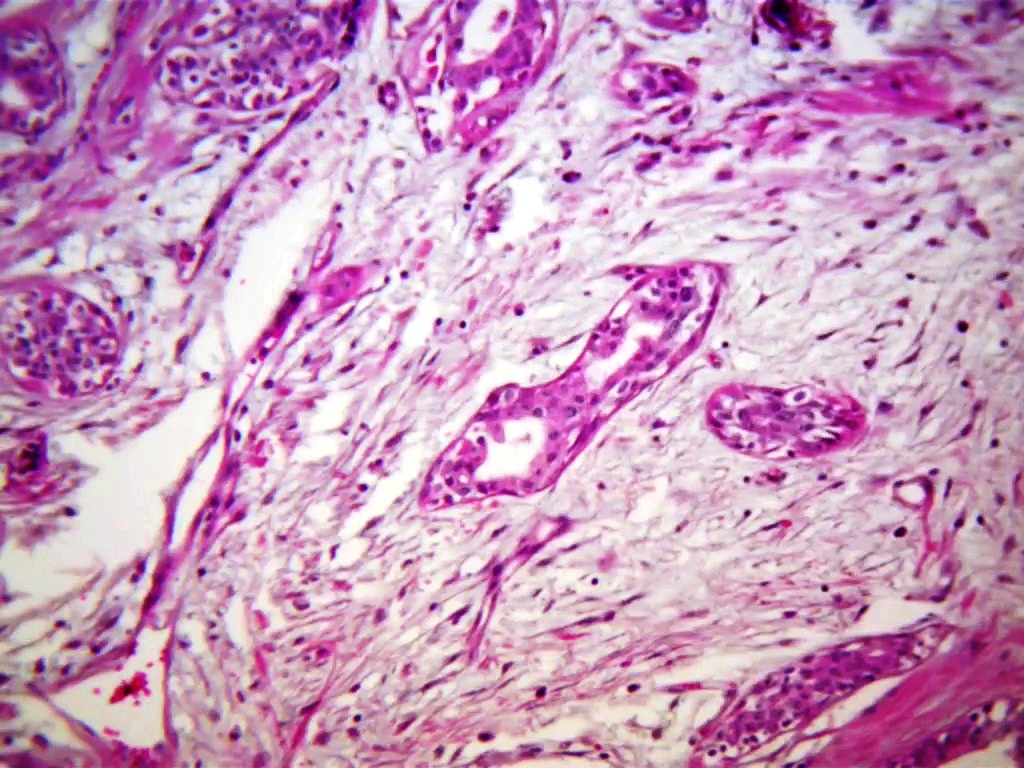

Supplement: Supplementary file 1 [file animals-13-01563-s001.zip › supplementary files/File S1 Canine Mammary Tumor Dataset/benign/Benign mixed tumor_28-12V2_FRM_006 (19).jpg]

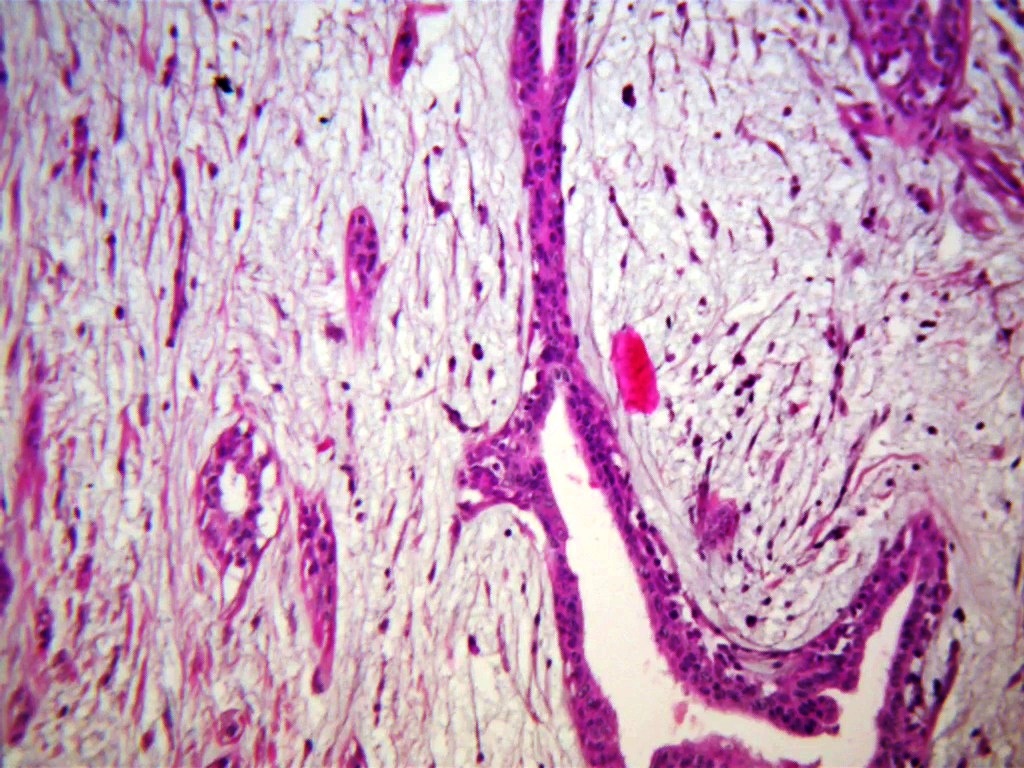

Supplement: Supplementary file 1 [file animals-13-01563-s001.zip › supplementary files/File S1 Canine Mammary Tumor Dataset/benign/Benign mixed tumor_28-12V2_FRM_006 (2).jpg]

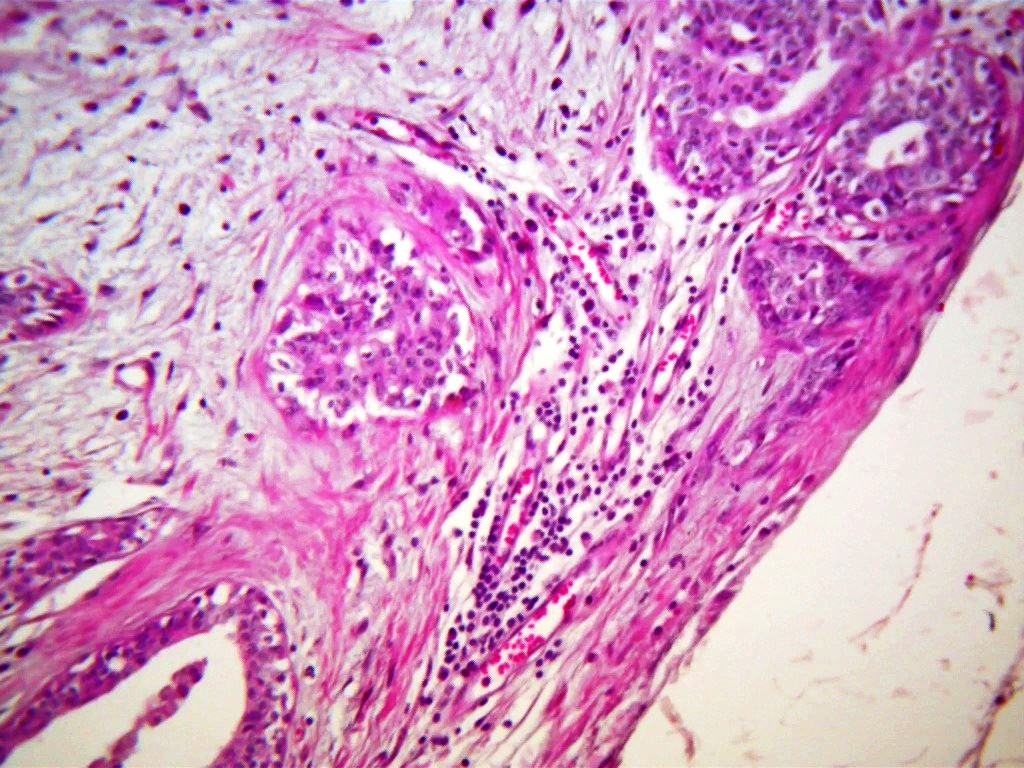

Supplement: Supplementary file 1 [file animals-13-01563-s001.zip › supplementary files/File S1 Canine Mammary Tumor Dataset/benign/Benign mixed tumor_28-12V2_FRM_006 (20).jpg]

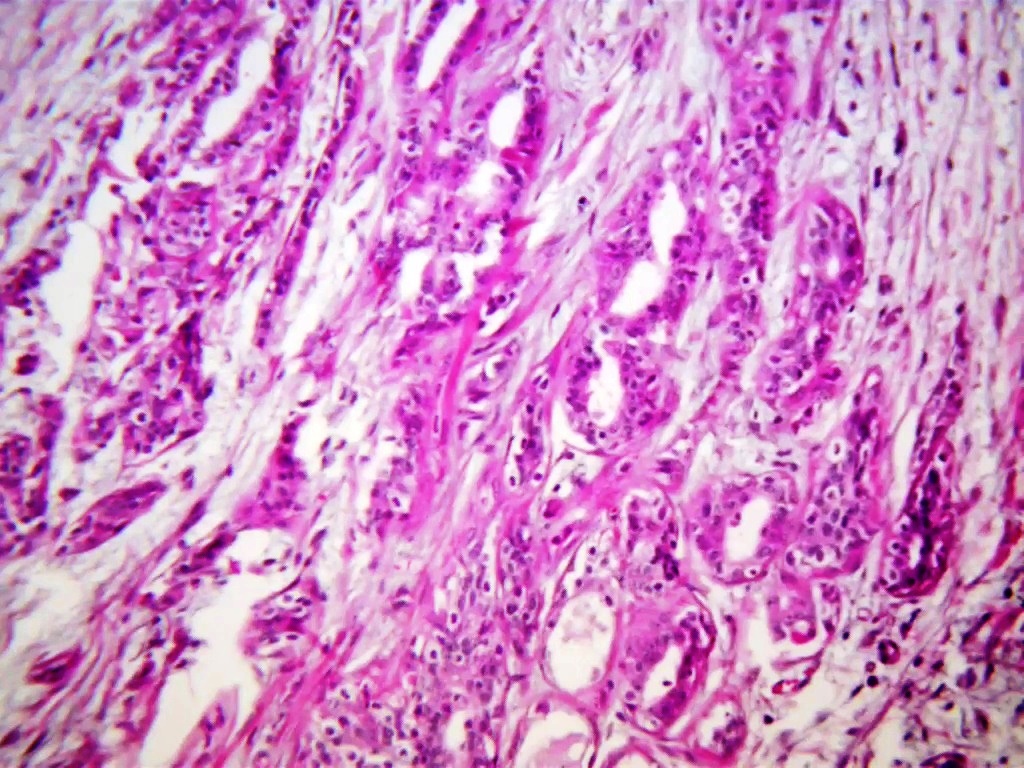

Supplement: Supplementary file 1 [file animals-13-01563-s001.zip › supplementary files/File S1 Canine Mammary Tumor Dataset/benign/Benign mixed tumor_28-12V2_FRM_006 (21).jpg]

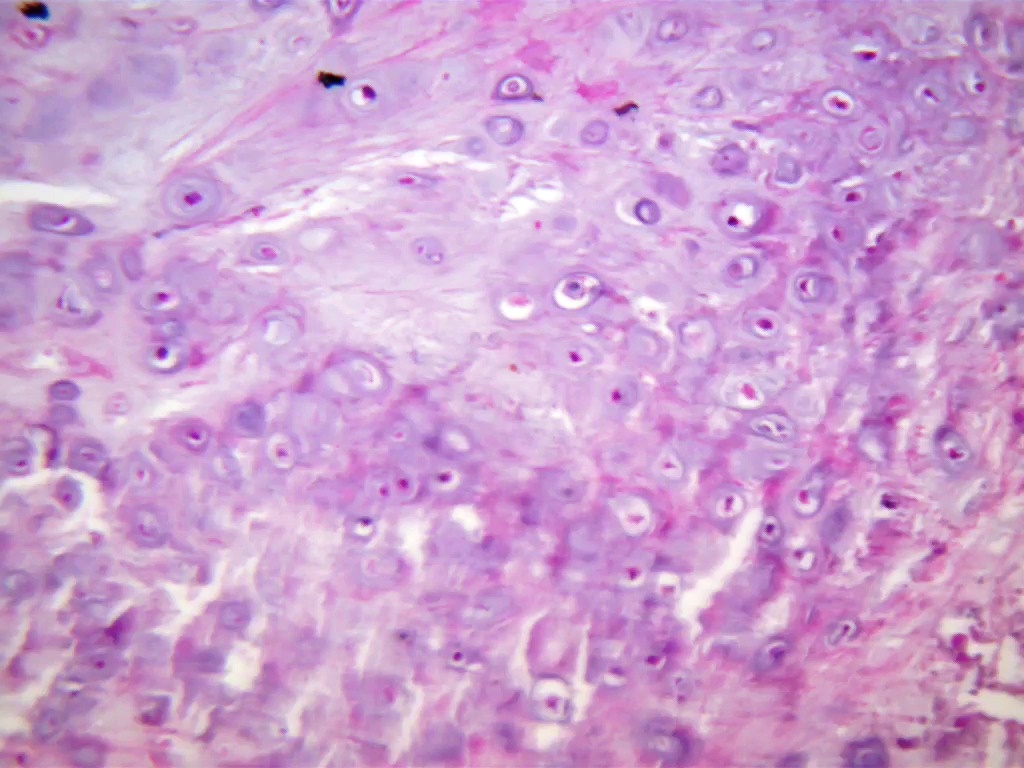

Supplement: Supplementary file 1 [file animals-13-01563-s001.zip › supplementary files/File S1 Canine Mammary Tumor Dataset/benign/Benign mixed tumor_28-12V2_FRM_006 (22).jpg]

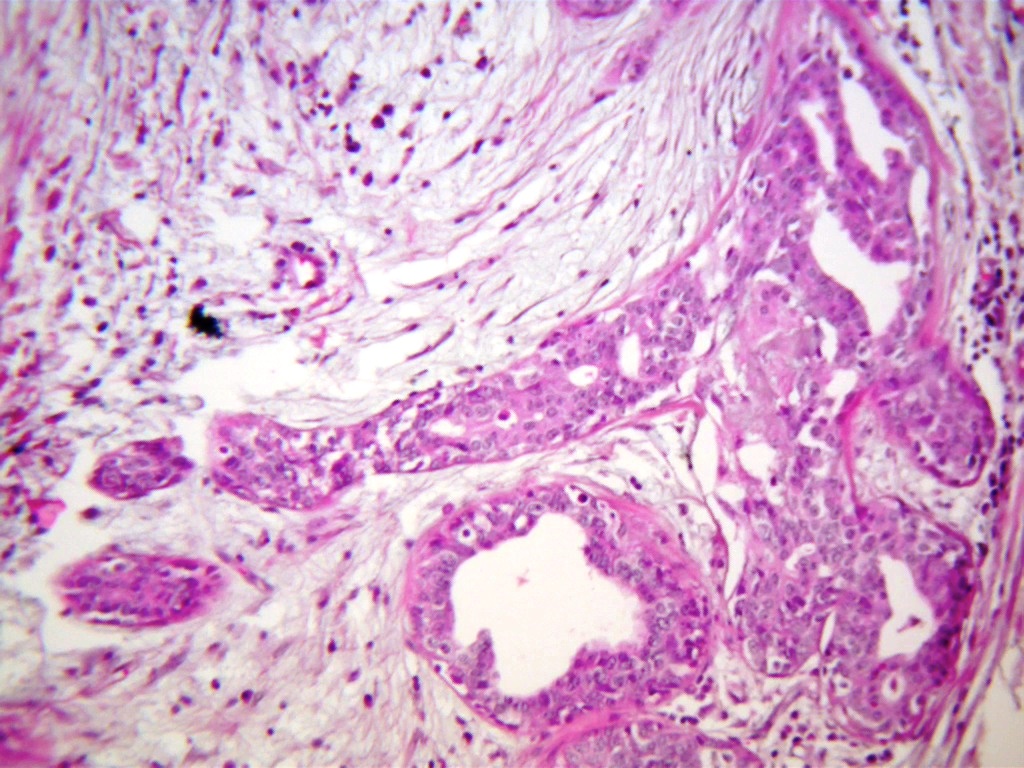

Supplement: Supplementary file 1 [file animals-13-01563-s001.zip › supplementary files/File S1 Canine Mammary Tumor Dataset/benign/Benign mixed tumor_28-12V2_FRM_006 (23).jpg]

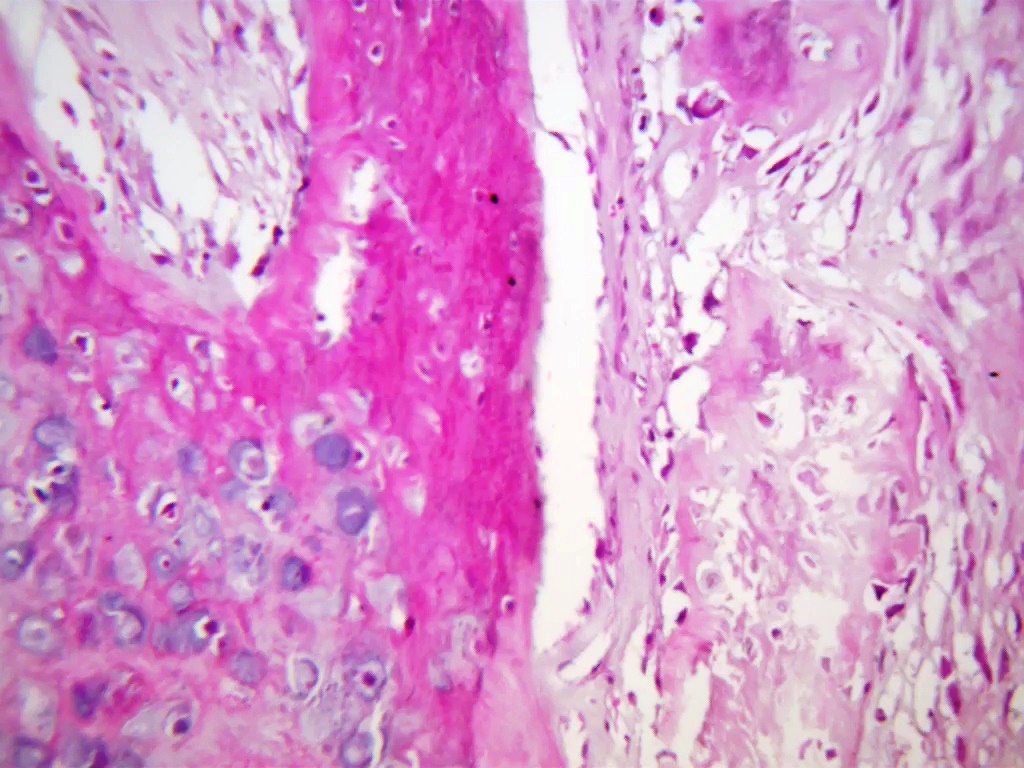

Supplement: Supplementary file 1 [file animals-13-01563-s001.zip › supplementary files/File S1 Canine Mammary Tumor Dataset/benign/Benign mixed tumor_28-12V2_FRM_006 (24).jpg]

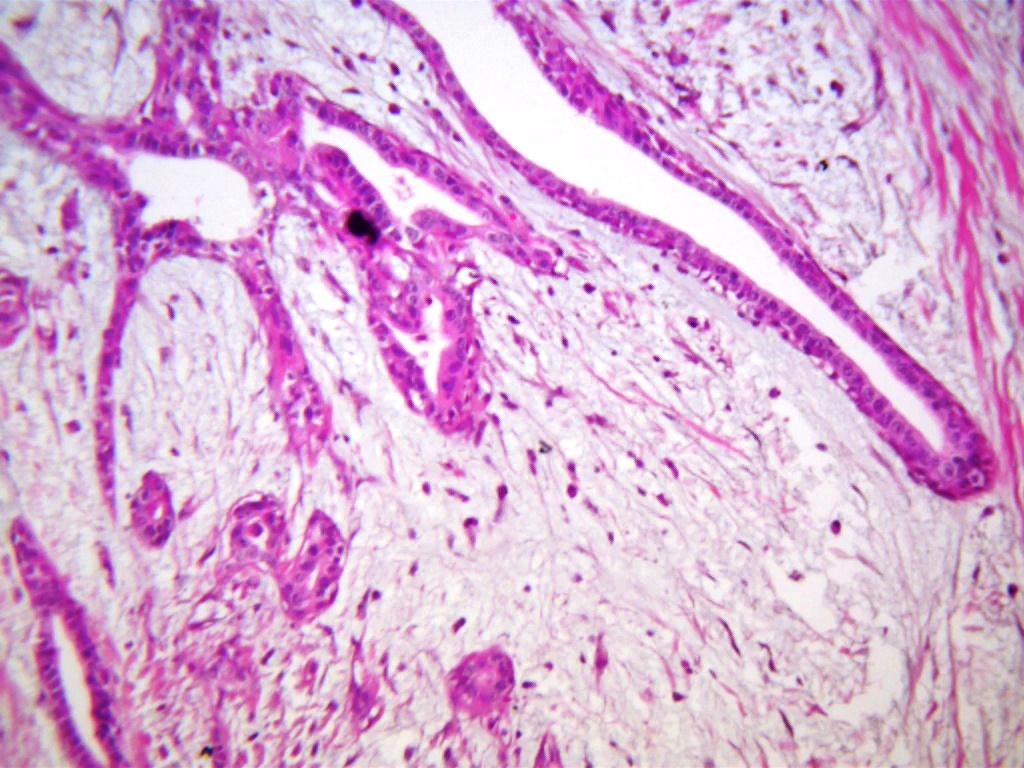

Supplement: Supplementary file 1 [file animals-13-01563-s001.zip › supplementary files/File S1 Canine Mammary Tumor Dataset/benign/Benign mixed tumor_28-12V2_FRM_006 (3).jpg]

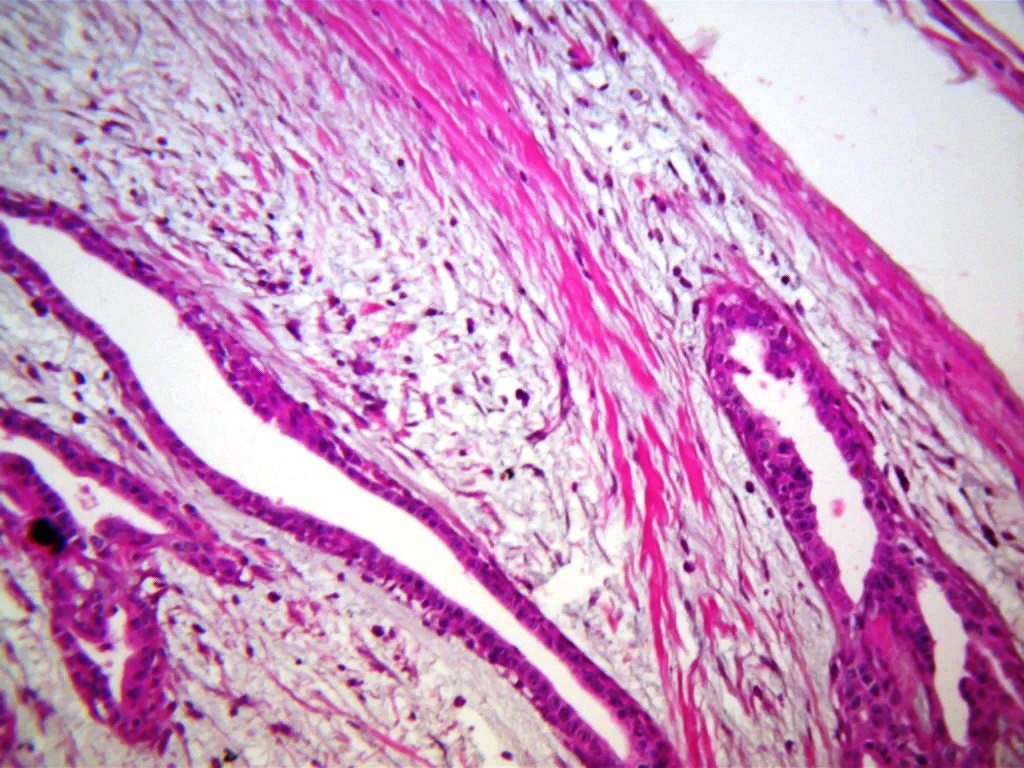

Supplement: Supplementary file 1 [file animals-13-01563-s001.zip › supplementary files/File S1 Canine Mammary Tumor Dataset/benign/Benign mixed tumor_28-12V2_FRM_006 (4).jpg]

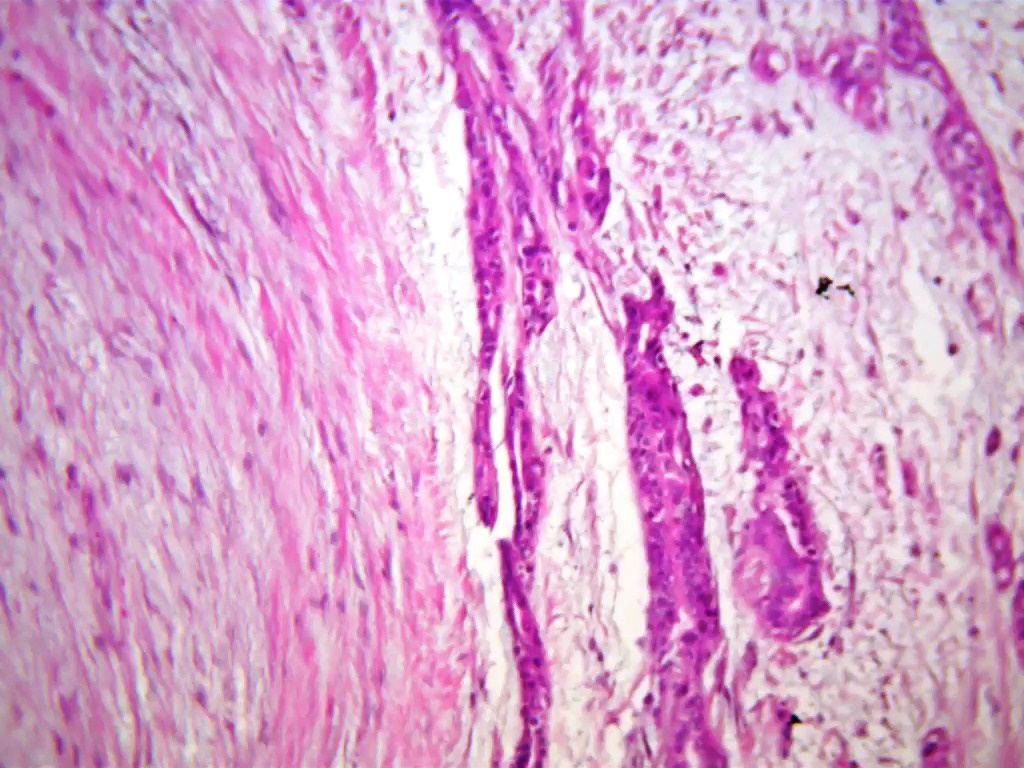

Supplement: Supplementary file 1 [file animals-13-01563-s001.zip › supplementary files/File S1 Canine Mammary Tumor Dataset/benign/Benign mixed tumor_28-12V2_FRM_006 (5).jpg]

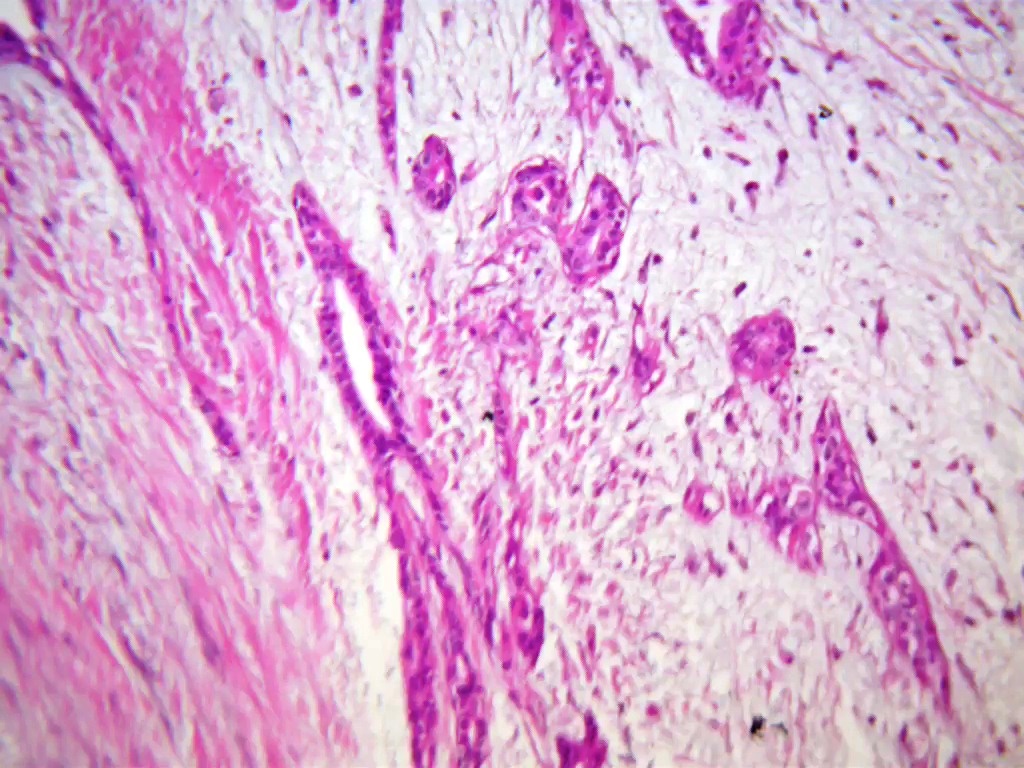

Supplement: Supplementary file 1 [file animals-13-01563-s001.zip › supplementary files/File S1 Canine Mammary Tumor Dataset/benign/Benign mixed tumor_28-12V2_FRM_006 (6).jpg]

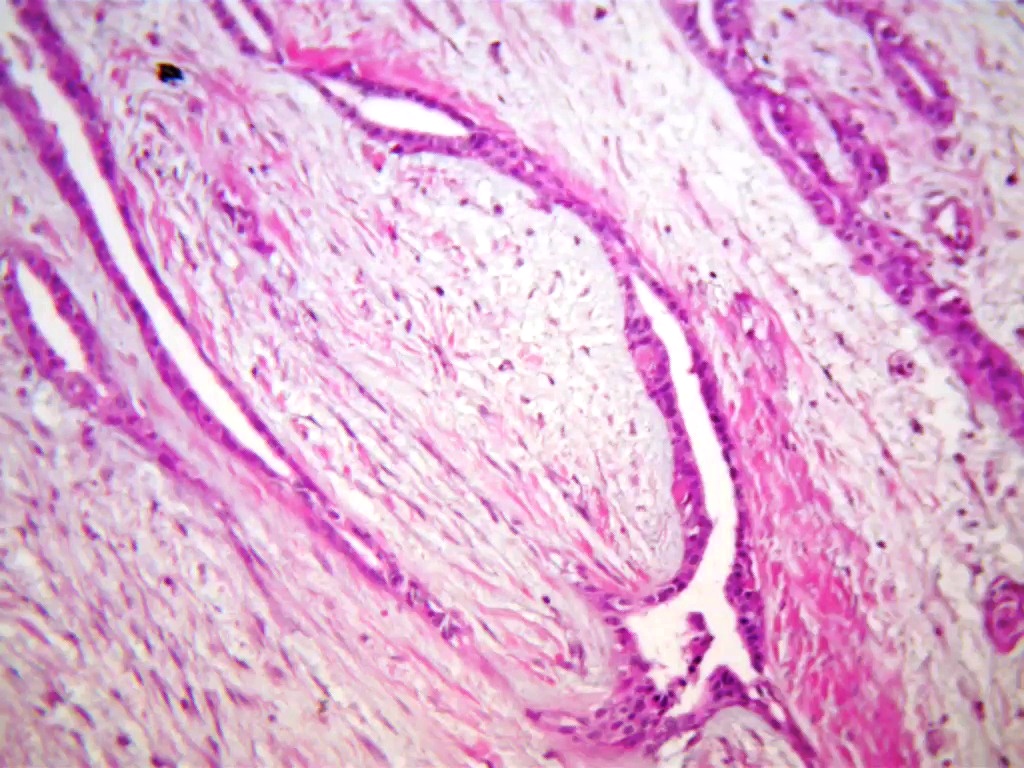

Supplement: Supplementary file 1 [file animals-13-01563-s001.zip › supplementary files/File S1 Canine Mammary Tumor Dataset/benign/Benign mixed tumor_28-12V2_FRM_006 (7).jpg]

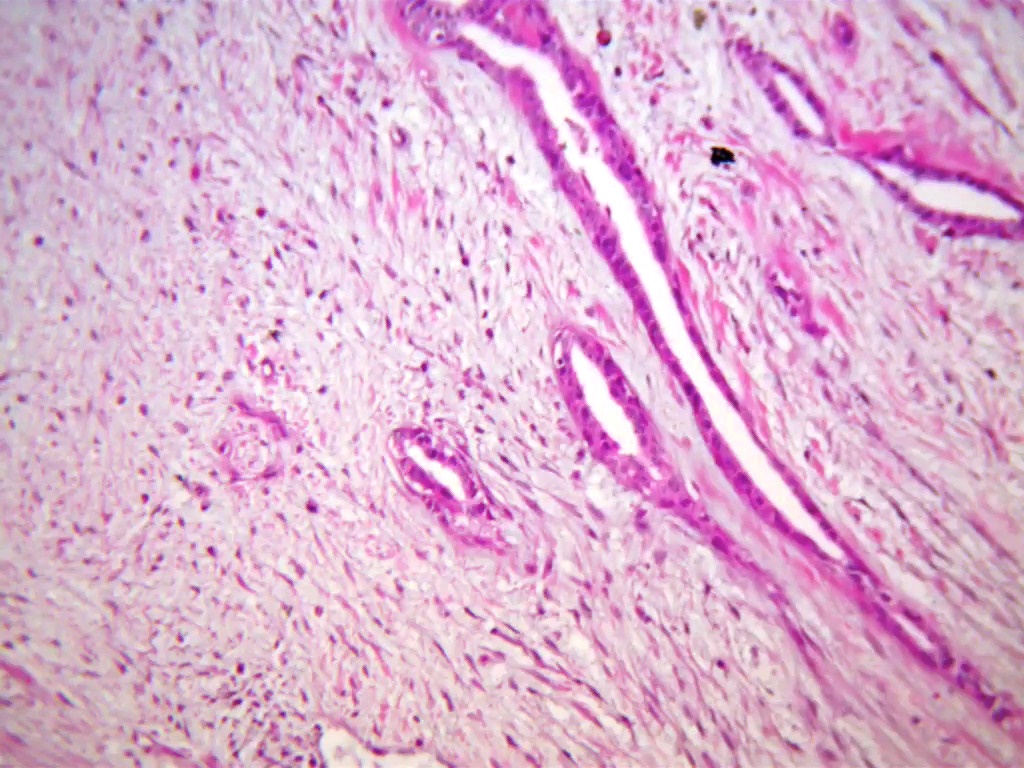

Supplement: Supplementary file 1 [file animals-13-01563-s001.zip › supplementary files/File S1 Canine Mammary Tumor Dataset/benign/Benign mixed tumor_28-12V2_FRM_006 (8).jpg]

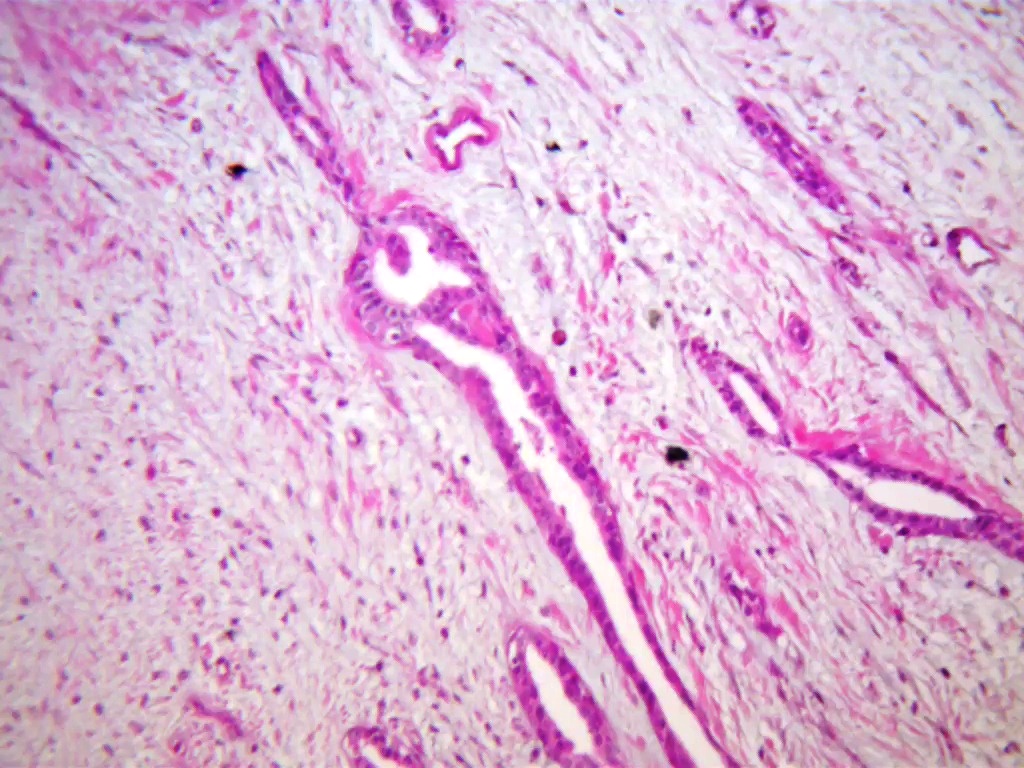

Supplement: Supplementary file 1 [file animals-13-01563-s001.zip › supplementary files/File S1 Canine Mammary Tumor Dataset/benign/Benign mixed tumor_28-12V2_FRM_006 (9).jpg]

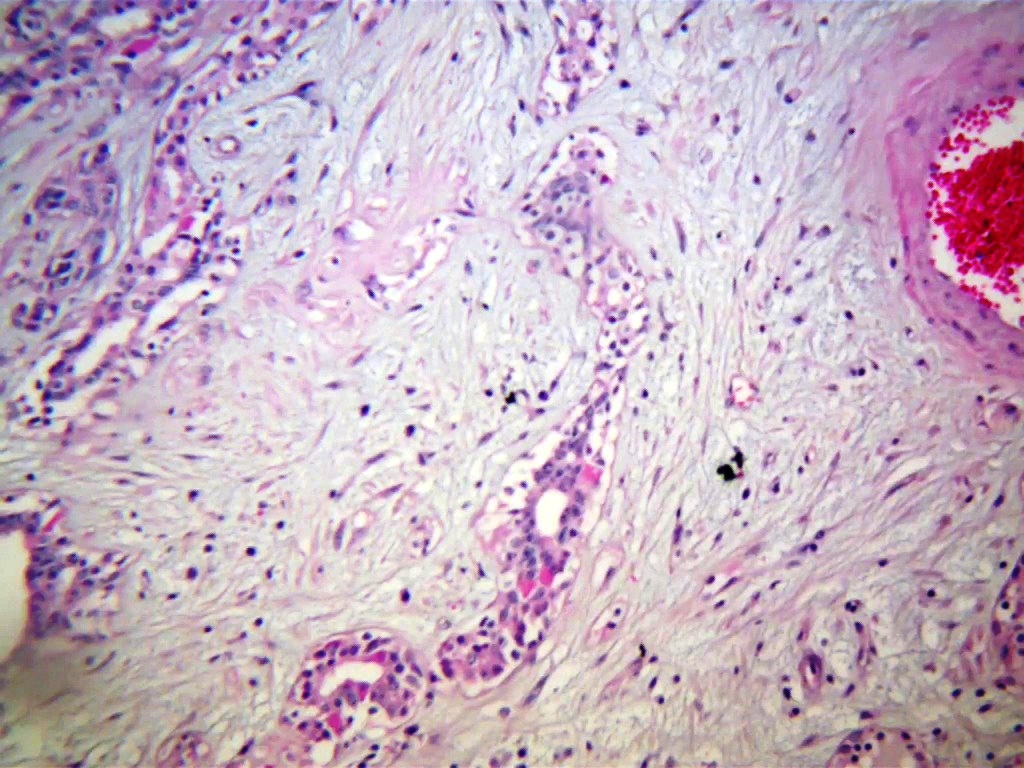

Supplement: Supplementary file 1 [file animals-13-01563-s001.zip › supplementary files/File S1 Canine Mammary Tumor Dataset/benign/Benign mixed tumor_28-13V2_FRM_008 (1).jpg]

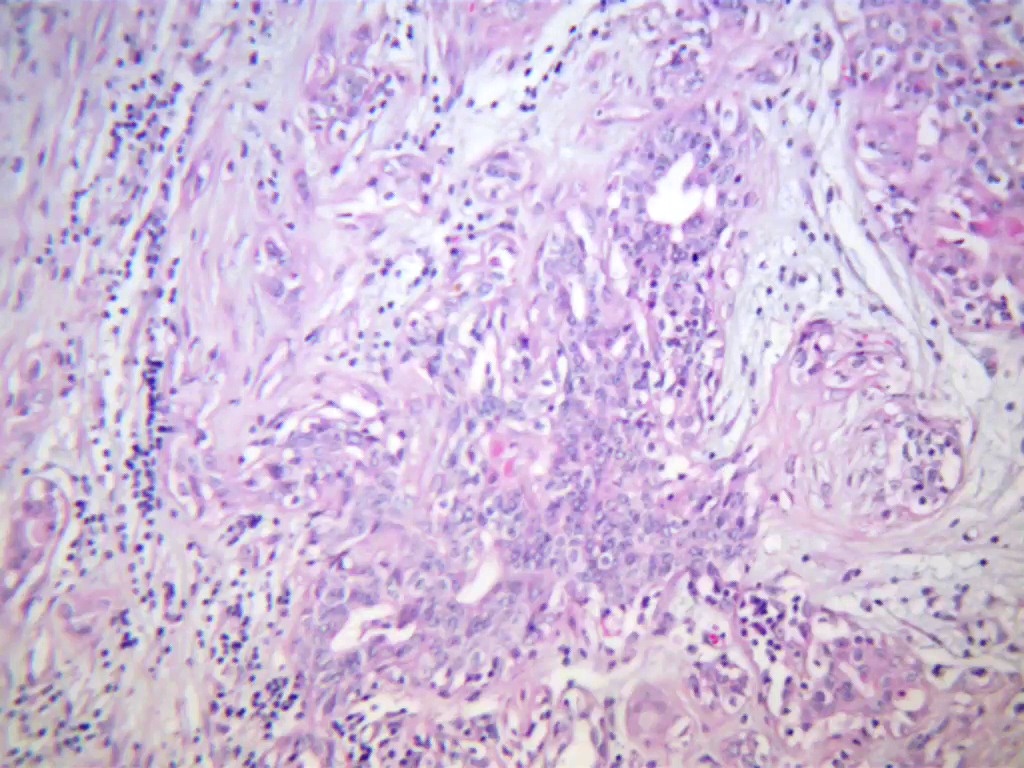

Supplement: Supplementary file 1 [file animals-13-01563-s001.zip › supplementary files/File S1 Canine Mammary Tumor Dataset/benign/Benign mixed tumor_28-13V2_FRM_008 (10).jpg]

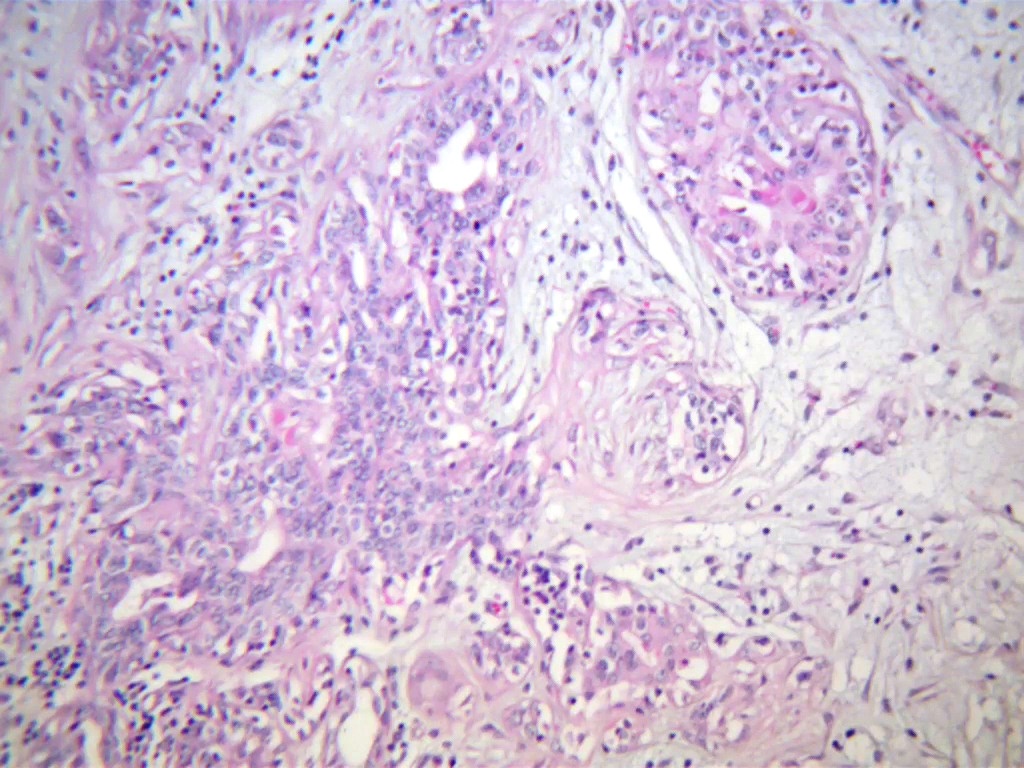

Supplement: Supplementary file 1 [file animals-13-01563-s001.zip › supplementary files/File S1 Canine Mammary Tumor Dataset/benign/Benign mixed tumor_28-13V2_FRM_008 (11).jpg]

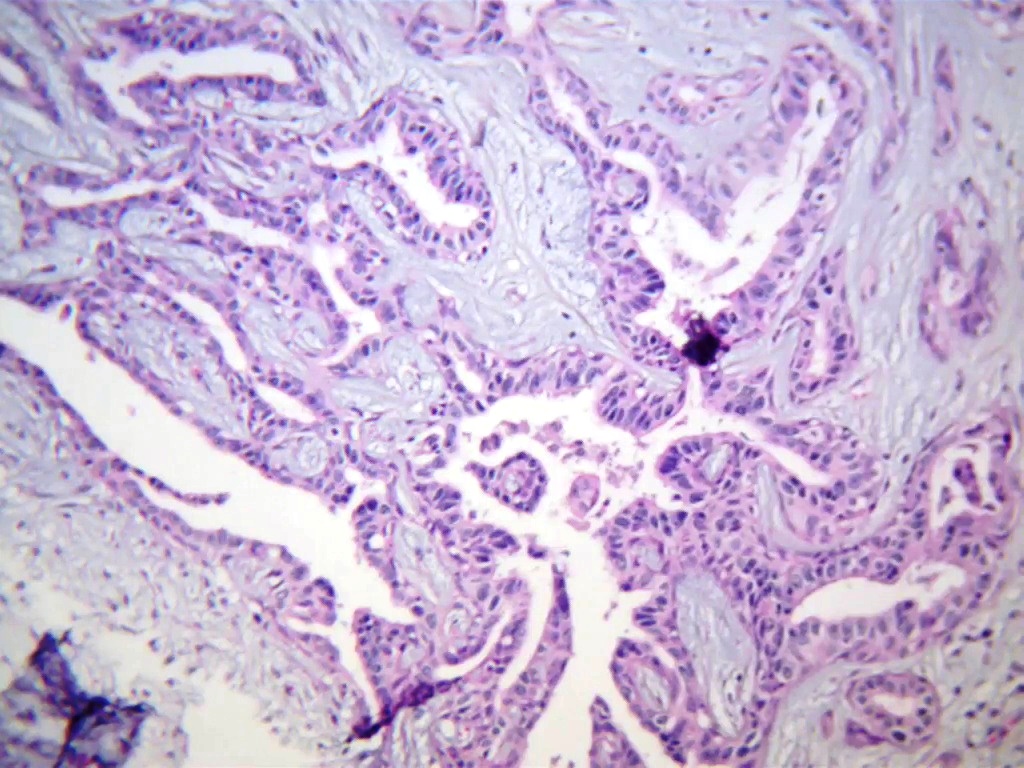

Supplement: Supplementary file 1 [file animals-13-01563-s001.zip › supplementary files/File S1 Canine Mammary Tumor Dataset/benign/Benign mixed tumor_28-13V2_FRM_008 (12).jpg]

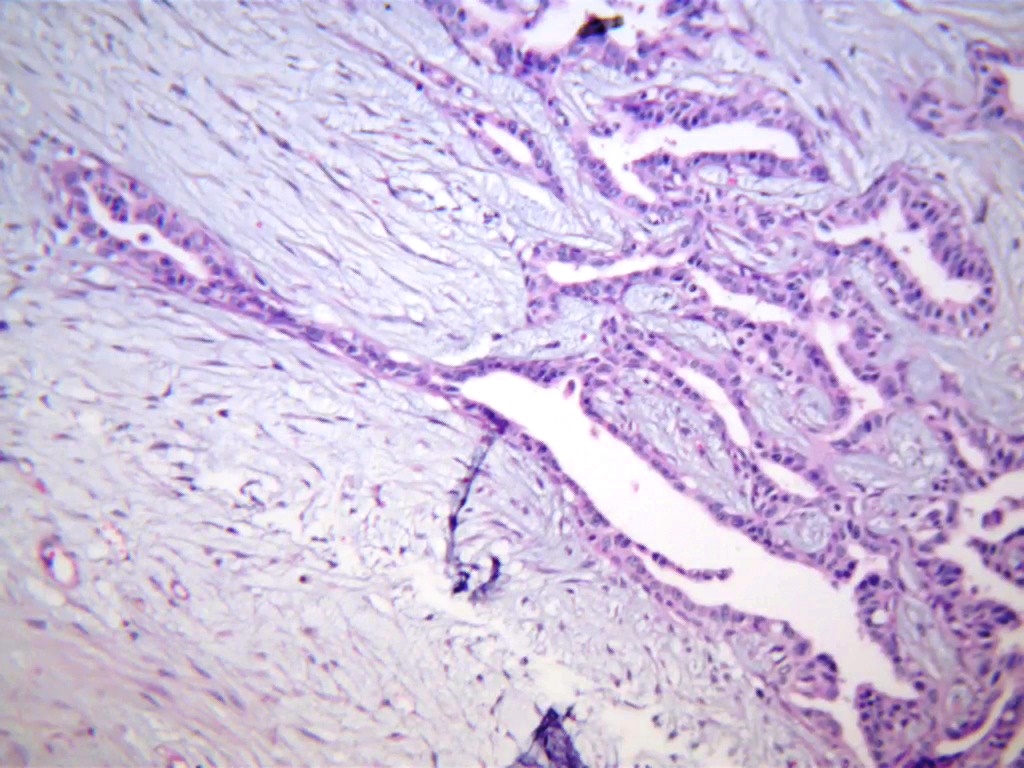

Supplement: Supplementary file 1 [file animals-13-01563-s001.zip › supplementary files/File S1 Canine Mammary Tumor Dataset/benign/Benign mixed tumor_28-13V2_FRM_008 (13).jpg]

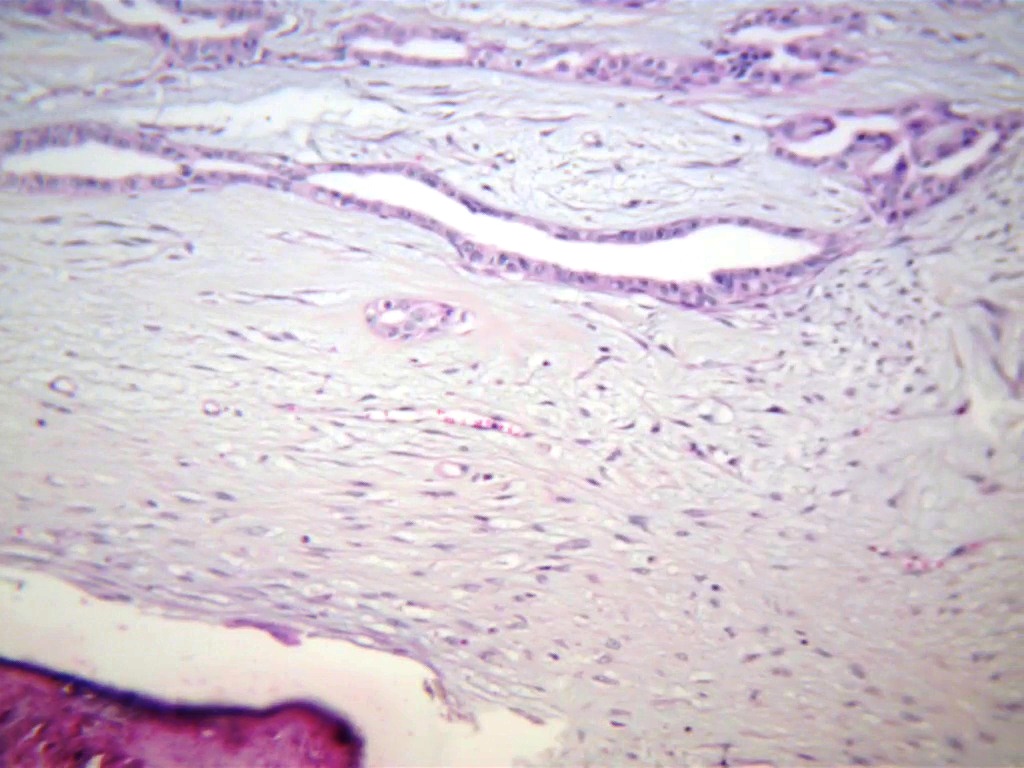

Supplement: Supplementary file 1 [file animals-13-01563-s001.zip › supplementary files/File S1 Canine Mammary Tumor Dataset/benign/Benign mixed tumor_28-13V2_FRM_008 (14).jpg]

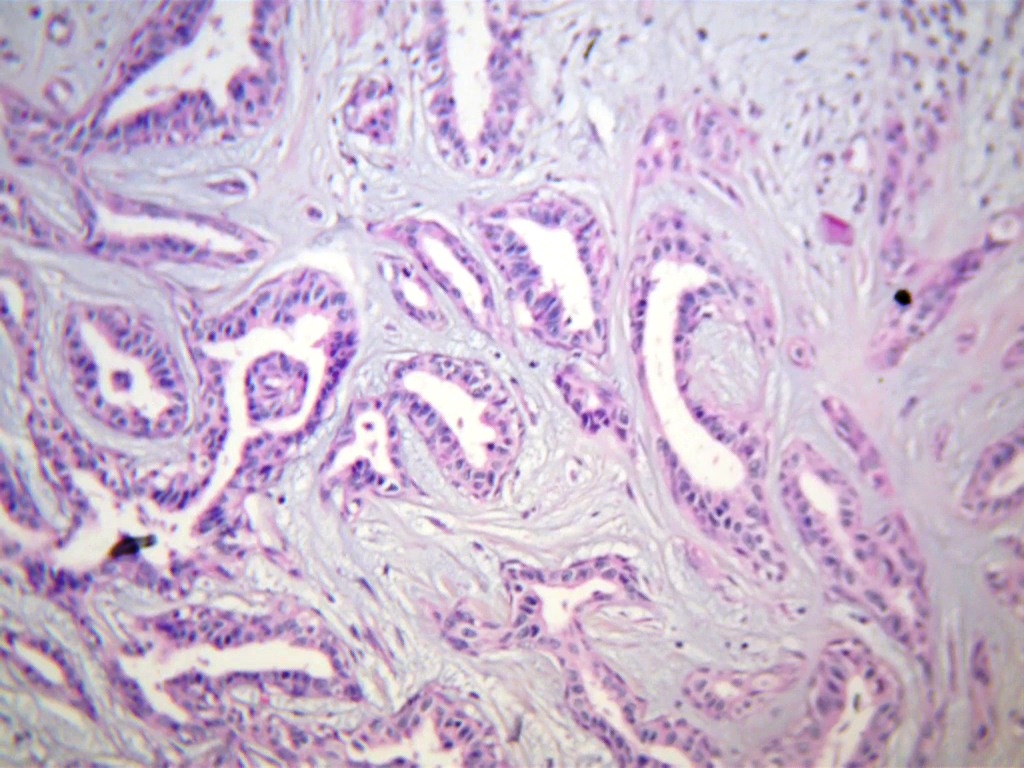

Supplement: Supplementary file 1 [file animals-13-01563-s001.zip › supplementary files/File S1 Canine Mammary Tumor Dataset/benign/Benign mixed tumor_28-13V2_FRM_008 (15).jpg]

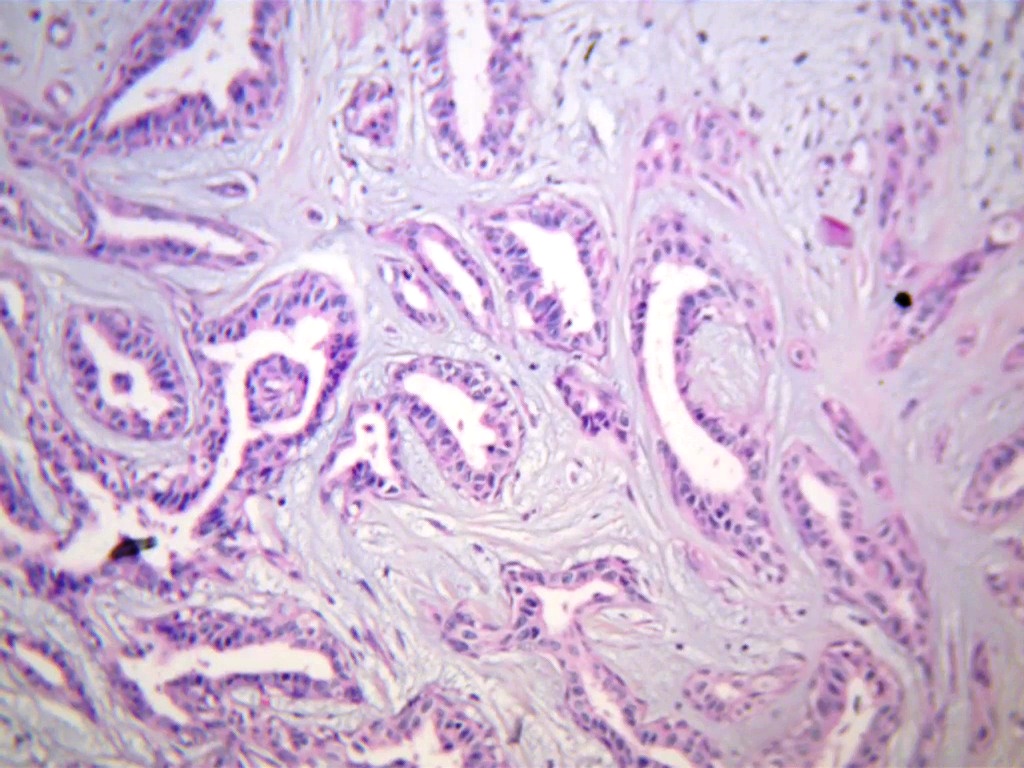

Supplement: Supplementary file 1 [file animals-13-01563-s001.zip › supplementary files/File S1 Canine Mammary Tumor Dataset/benign/Benign mixed tumor_28-13V2_FRM_008 (16).jpg]

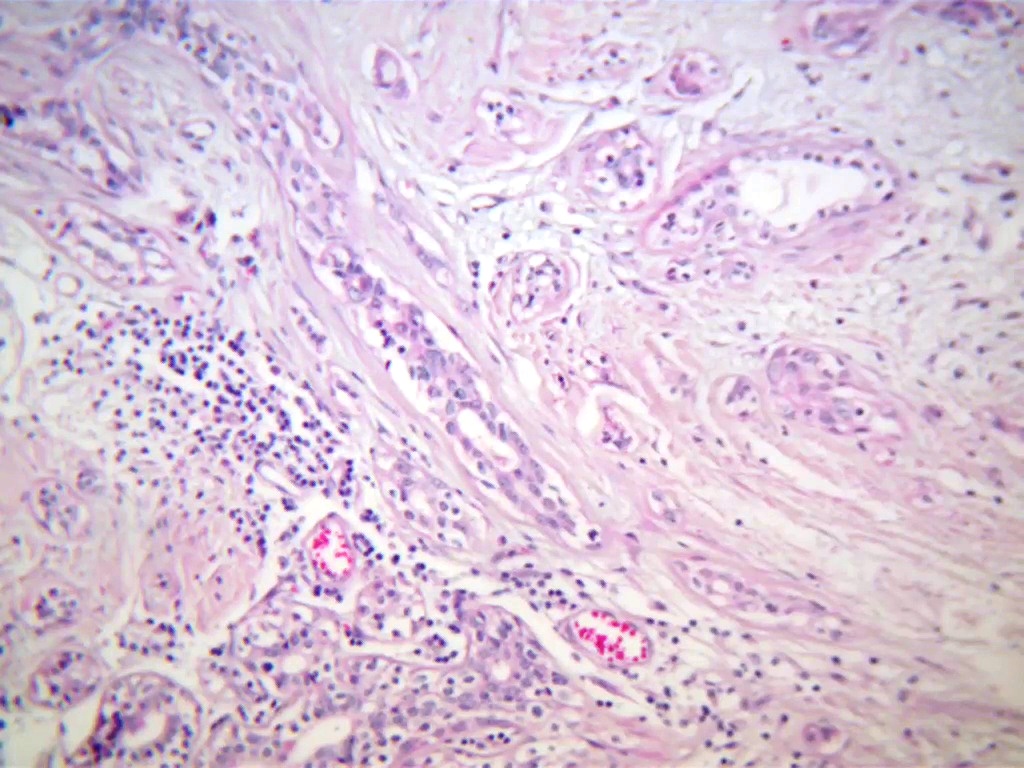

Supplement: Supplementary file 1 [file animals-13-01563-s001.zip › supplementary files/File S1 Canine Mammary Tumor Dataset/benign/Benign mixed tumor_28-13V2_FRM_008 (17).jpg]

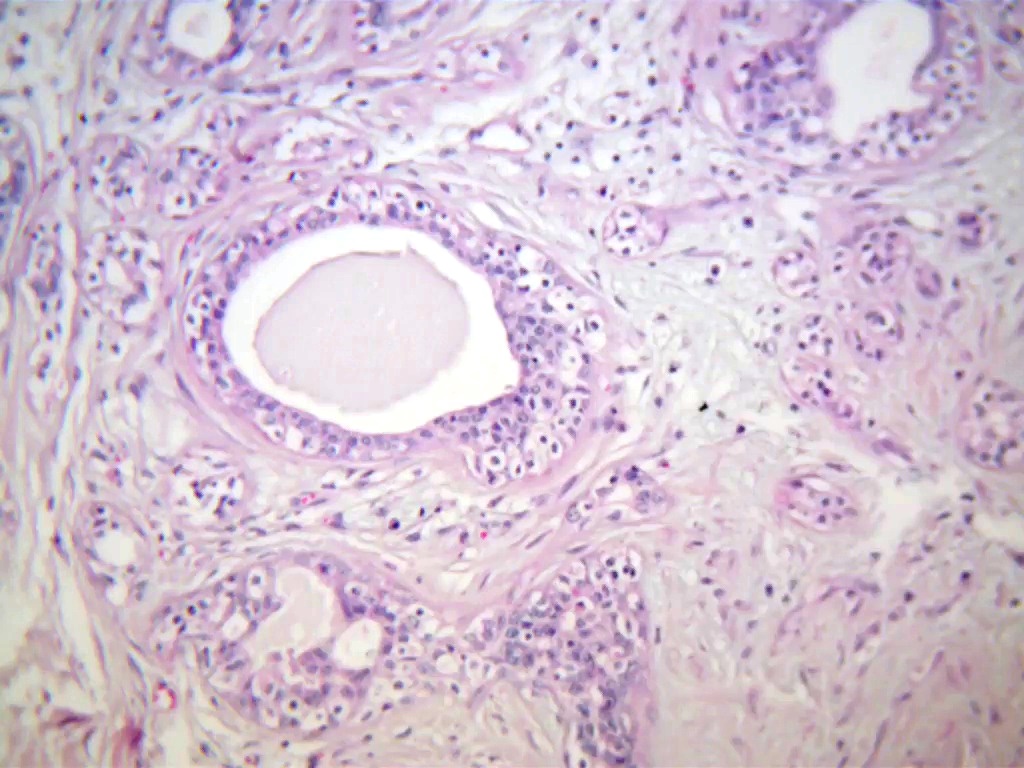

Supplement: Supplementary file 1 [file animals-13-01563-s001.zip › supplementary files/File S1 Canine Mammary Tumor Dataset/benign/Benign mixed tumor_28-13V2_FRM_008 (18).jpg]

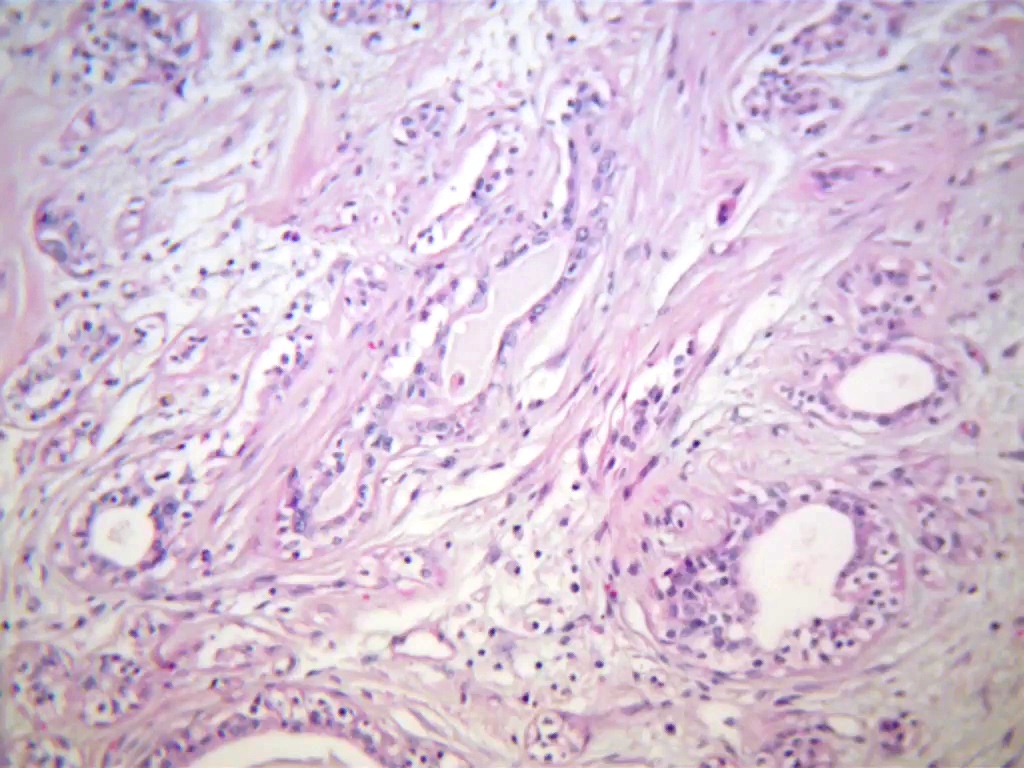

Supplement: Supplementary file 1 [file animals-13-01563-s001.zip › supplementary files/File S1 Canine Mammary Tumor Dataset/benign/Benign mixed tumor_28-13V2_FRM_008 (19).jpg]

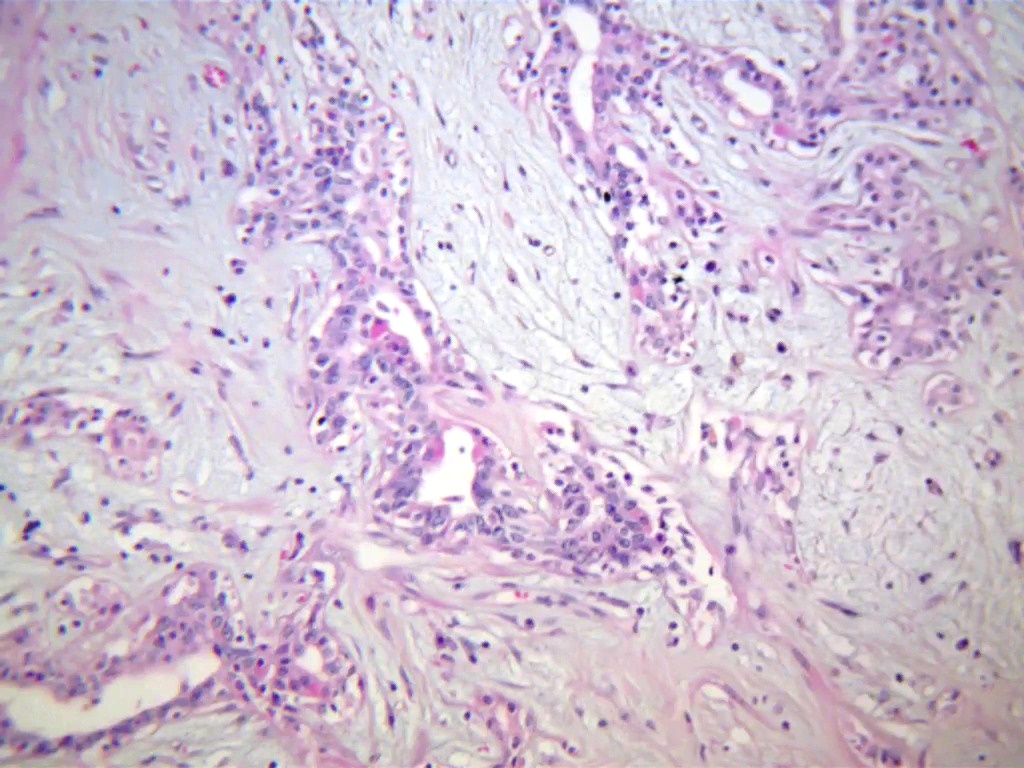

Supplement: Supplementary file 1 [file animals-13-01563-s001.zip › supplementary files/File S1 Canine Mammary Tumor Dataset/benign/Benign mixed tumor_28-13V2_FRM_008 (2).jpg]

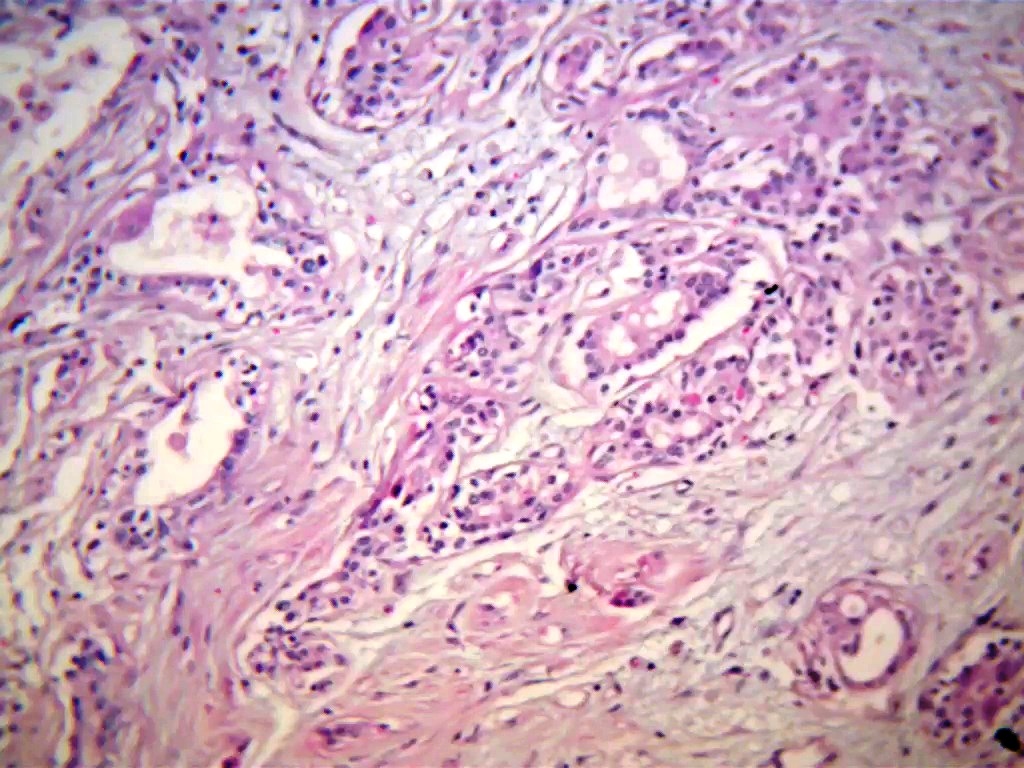

Supplement: Supplementary file 1 [file animals-13-01563-s001.zip › supplementary files/File S1 Canine Mammary Tumor Dataset/benign/Benign mixed tumor_28-13V2_FRM_008 (20).jpg]

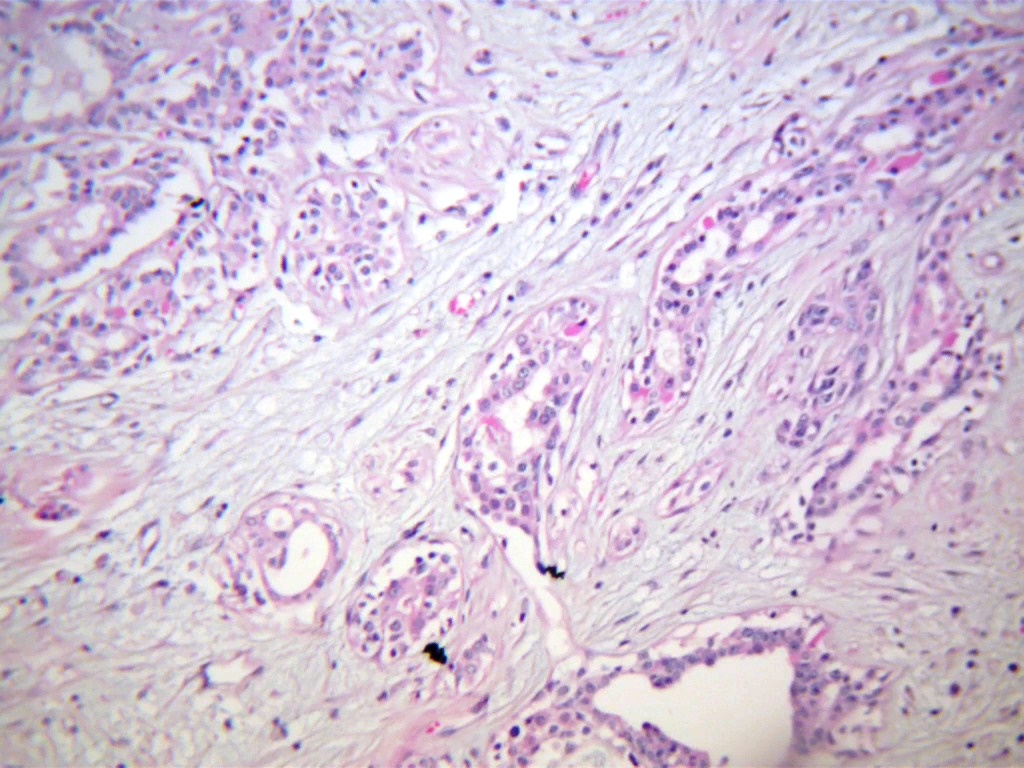

Supplement: Supplementary file 1 [file animals-13-01563-s001.zip › supplementary files/File S1 Canine Mammary Tumor Dataset/benign/Benign mixed tumor_28-13V2_FRM_008 (21).jpg]

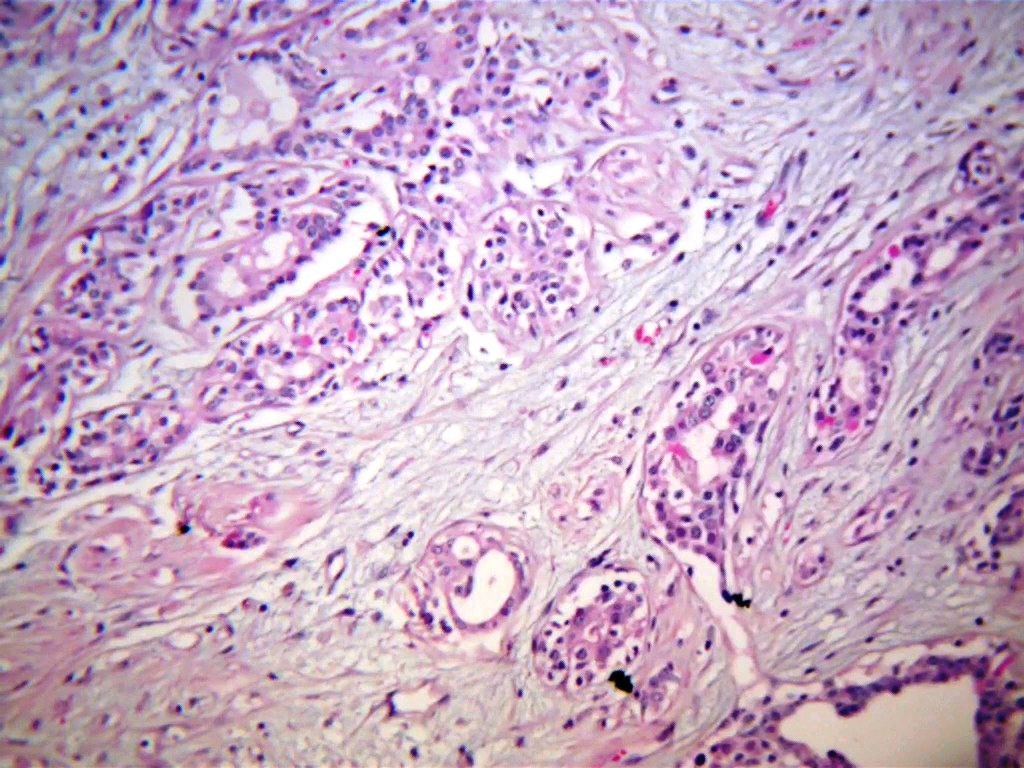

Supplement: Supplementary file 1 [file animals-13-01563-s001.zip › supplementary files/File S1 Canine Mammary Tumor Dataset/benign/Benign mixed tumor_28-13V2_FRM_008 (22).jpg]

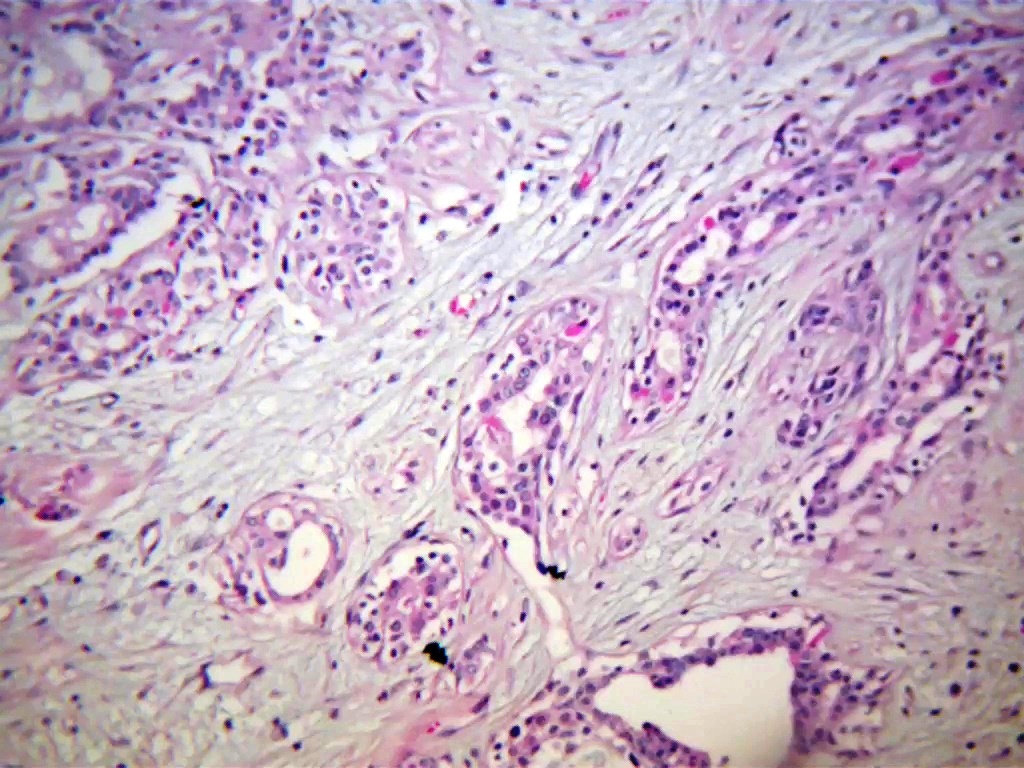

Supplement: Supplementary file 1 [file animals-13-01563-s001.zip › supplementary files/File S1 Canine Mammary Tumor Dataset/benign/Benign mixed tumor_28-13V2_FRM_008 (23).jpg]

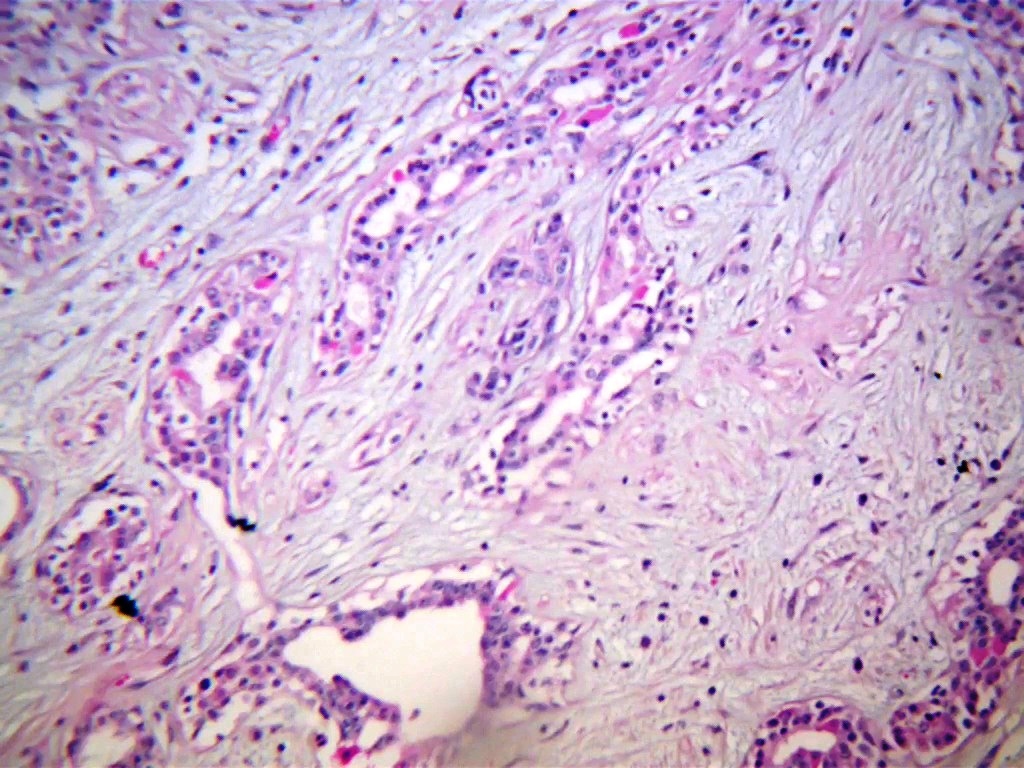

Supplement: Supplementary file 1 [file animals-13-01563-s001.zip › supplementary files/File S1 Canine Mammary Tumor Dataset/benign/Benign mixed tumor_28-13V2_FRM_008 (24).jpg]

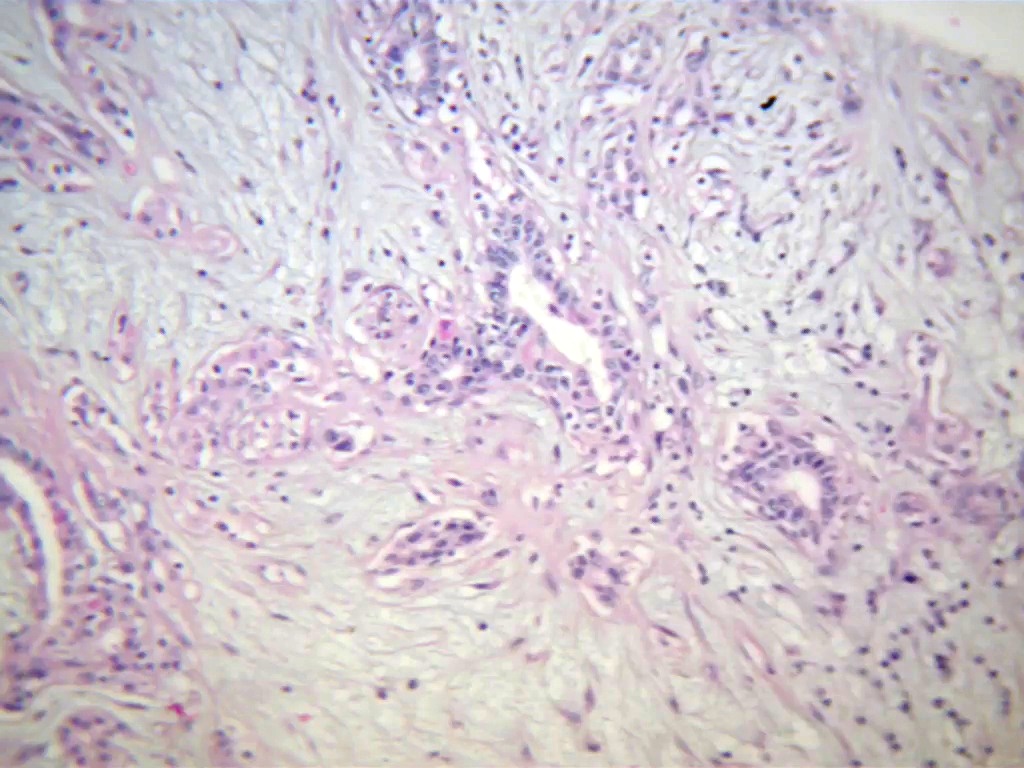

Supplement: Supplementary file 1 [file animals-13-01563-s001.zip › supplementary files/File S1 Canine Mammary Tumor Dataset/benign/Benign mixed tumor_28-13V2_FRM_008 (3).jpg]

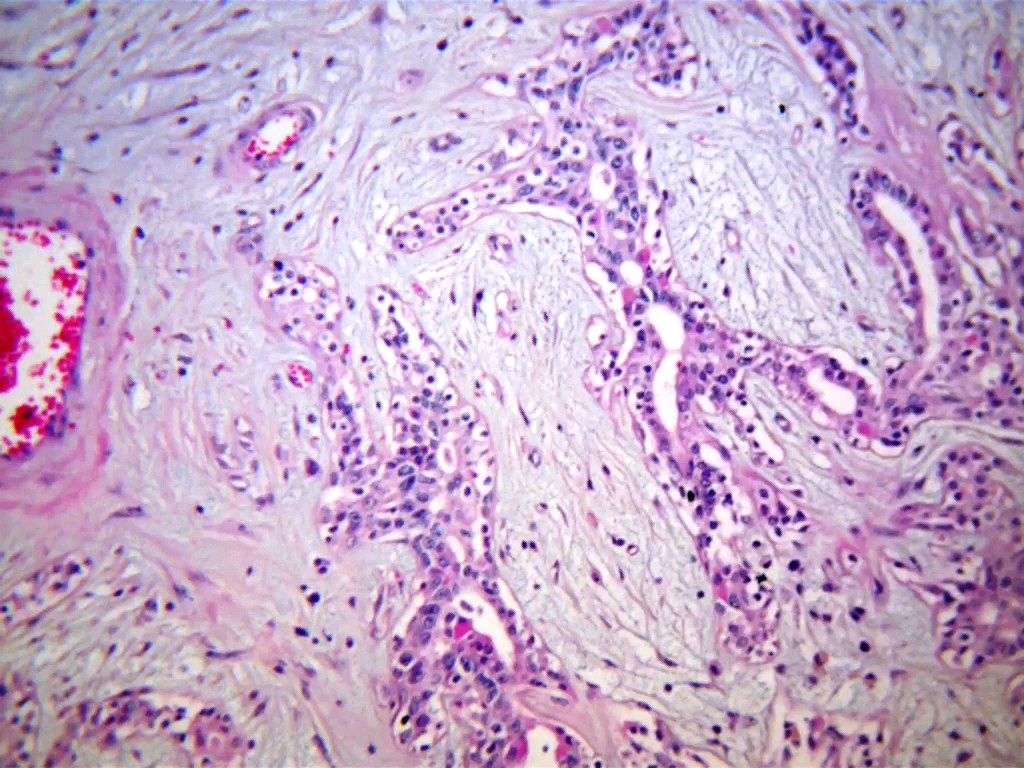

Supplement: Supplementary file 1 [file animals-13-01563-s001.zip › supplementary files/File S1 Canine Mammary Tumor Dataset/benign/Benign mixed tumor_28-13V2_FRM_008 (4).jpg]

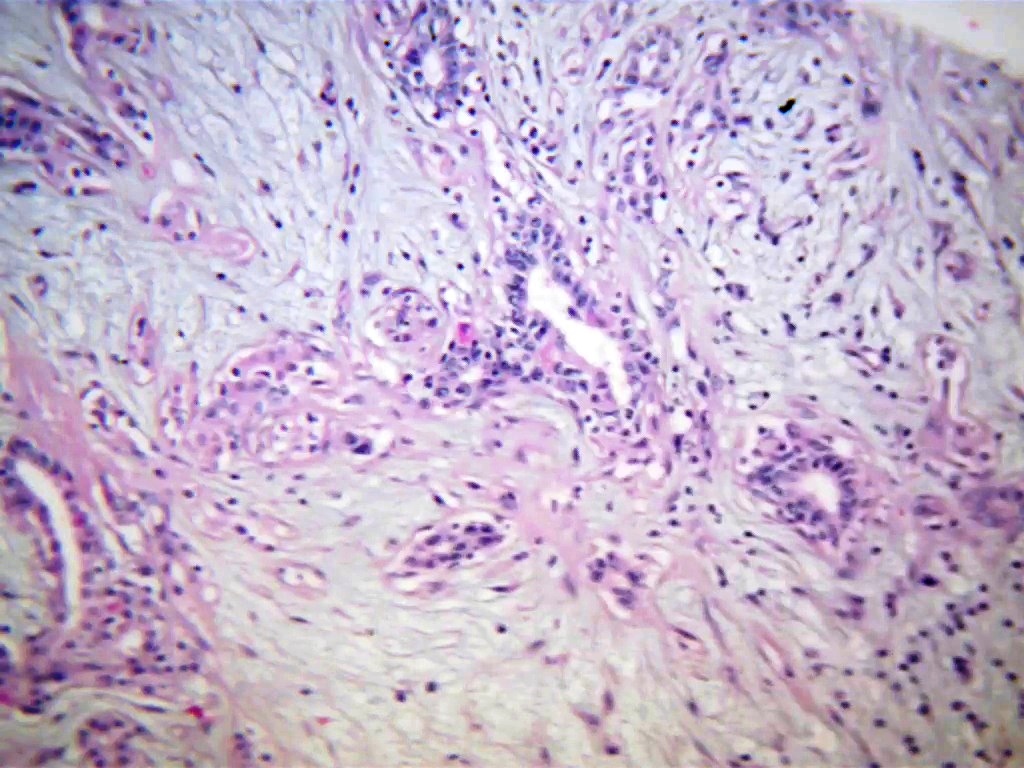

Supplement: Supplementary file 1 [file animals-13-01563-s001.zip › supplementary files/File S1 Canine Mammary Tumor Dataset/benign/Benign mixed tumor_28-13V2_FRM_008 (5).jpg]

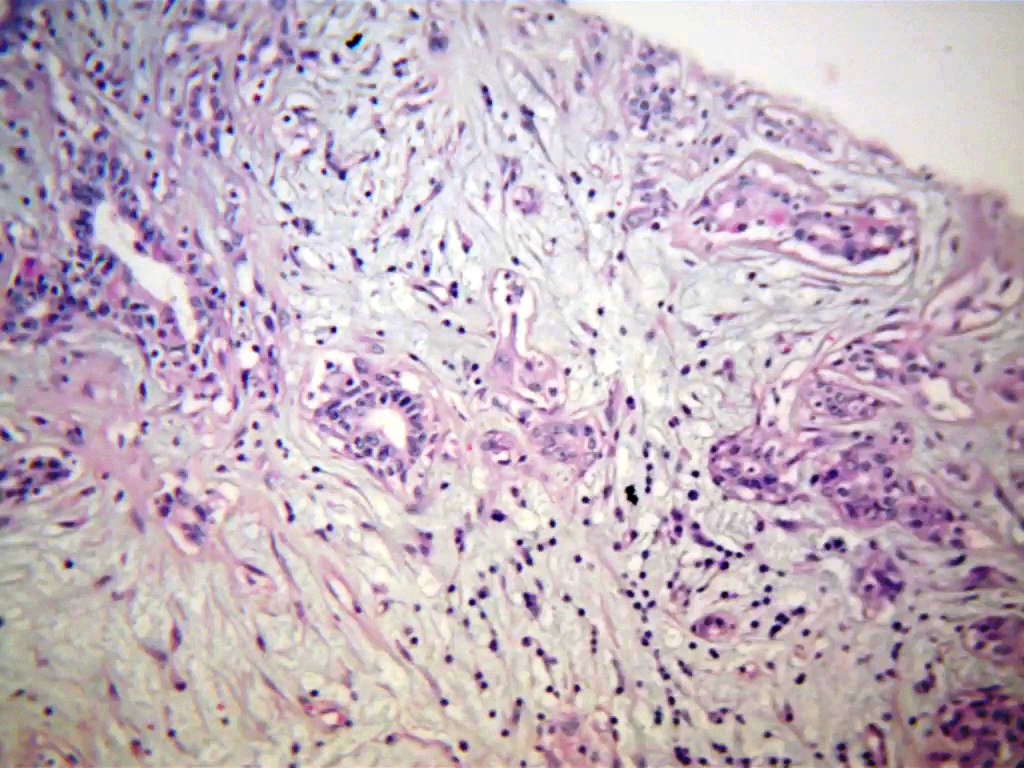

Supplement: Supplementary file 1 [file animals-13-01563-s001.zip › supplementary files/File S1 Canine Mammary Tumor Dataset/benign/Benign mixed tumor_28-13V2_FRM_008 (6).jpg]

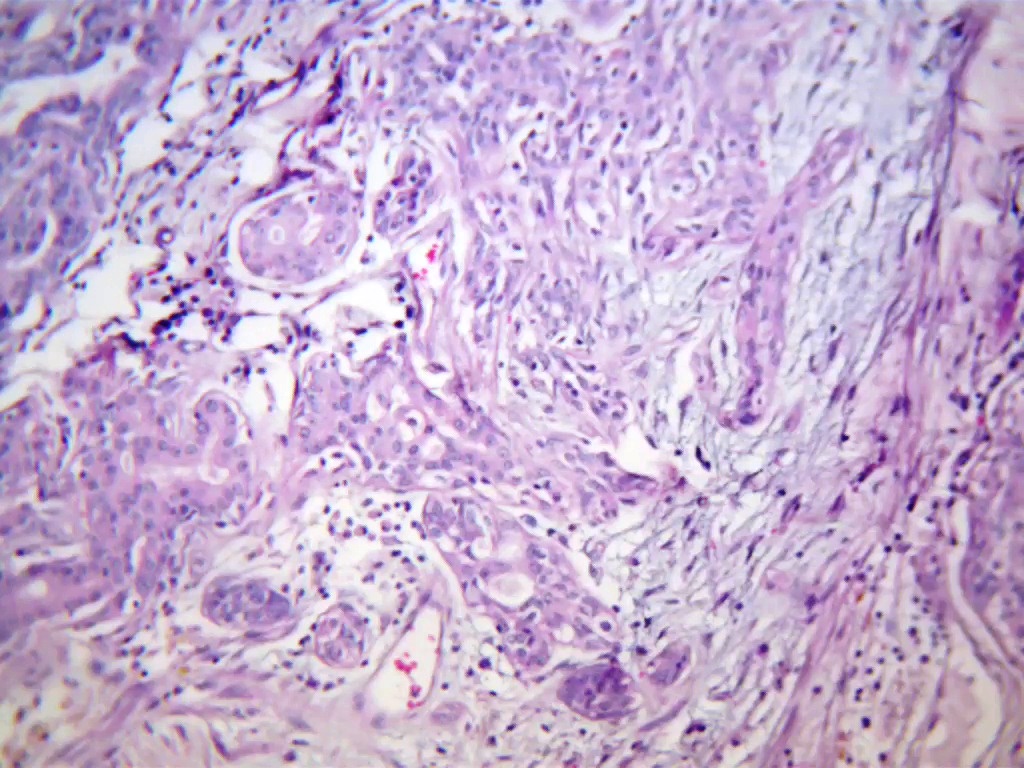

Supplement: Supplementary file 1 [file animals-13-01563-s001.zip › supplementary files/File S1 Canine Mammary Tumor Dataset/benign/Benign mixed tumor_28-13V2_FRM_008 (7).jpg]

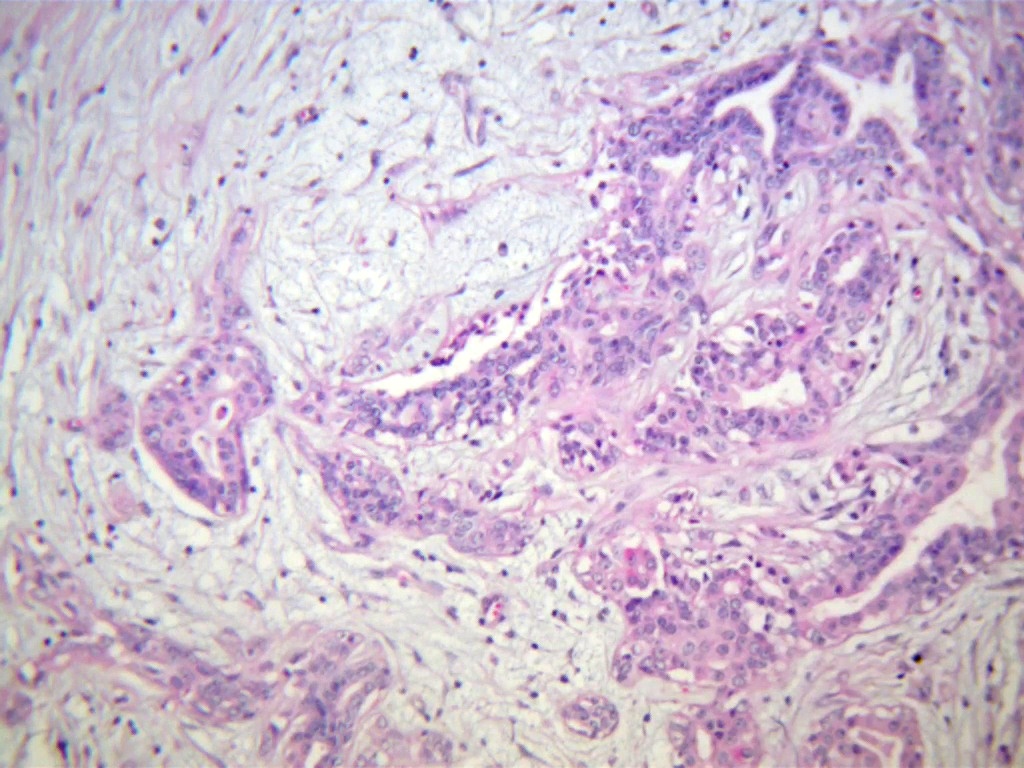

Supplement: Supplementary file 1 [file animals-13-01563-s001.zip › supplementary files/File S1 Canine Mammary Tumor Dataset/benign/Benign mixed tumor_28-13V2_FRM_008 (8).jpg]

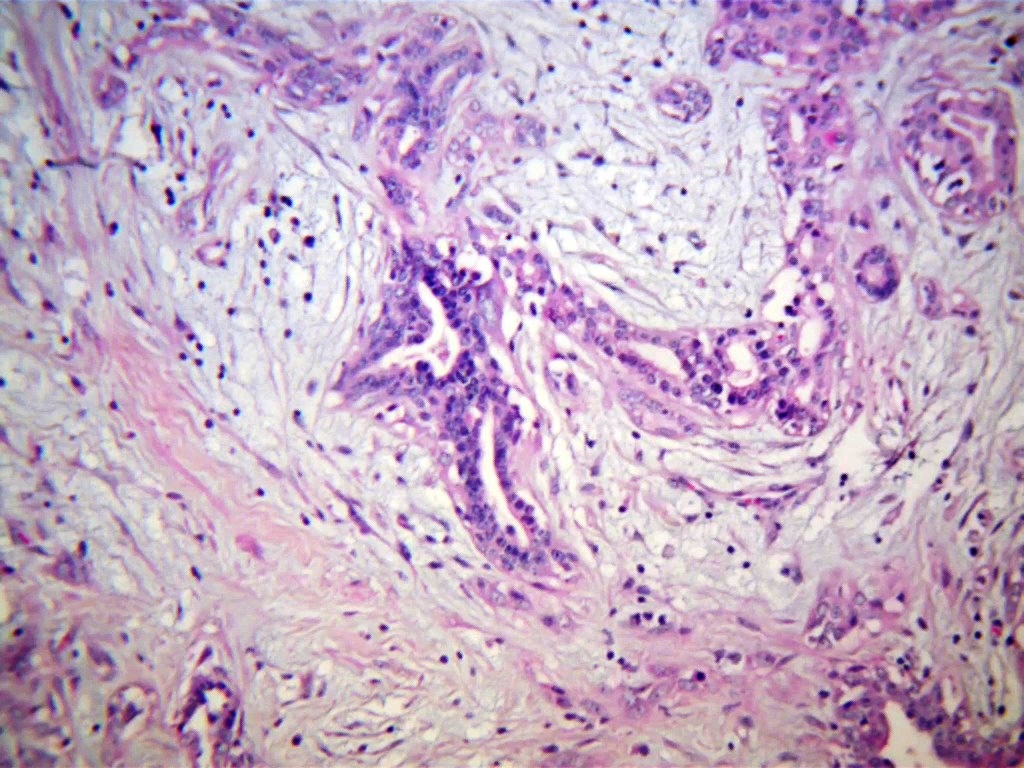

Supplement: Supplementary file 1 [file animals-13-01563-s001.zip › supplementary files/File S1 Canine Mammary Tumor Dataset/benign/Benign mixed tumor_28-13V2_FRM_008 (9).jpg]

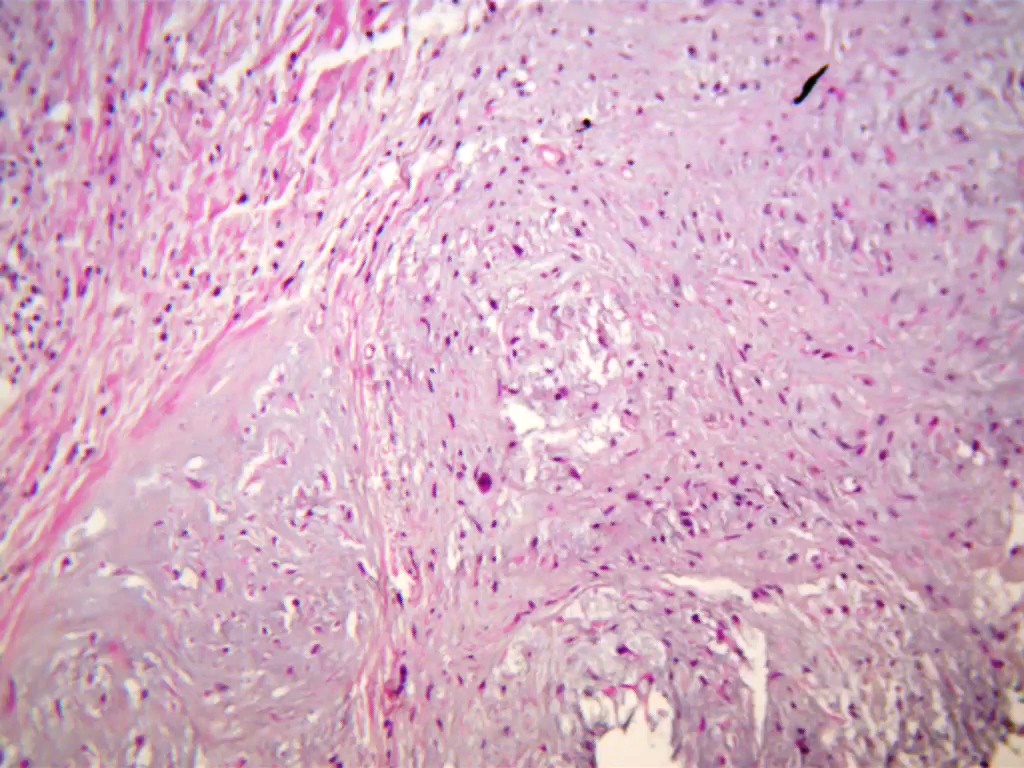

Supplement: Supplementary file 1 [file animals-13-01563-s001.zip › supplementary files/File S1 Canine Mammary Tumor Dataset/benign/Benign mixed tumor_465_11V2_FRM_011 (1).jpg]

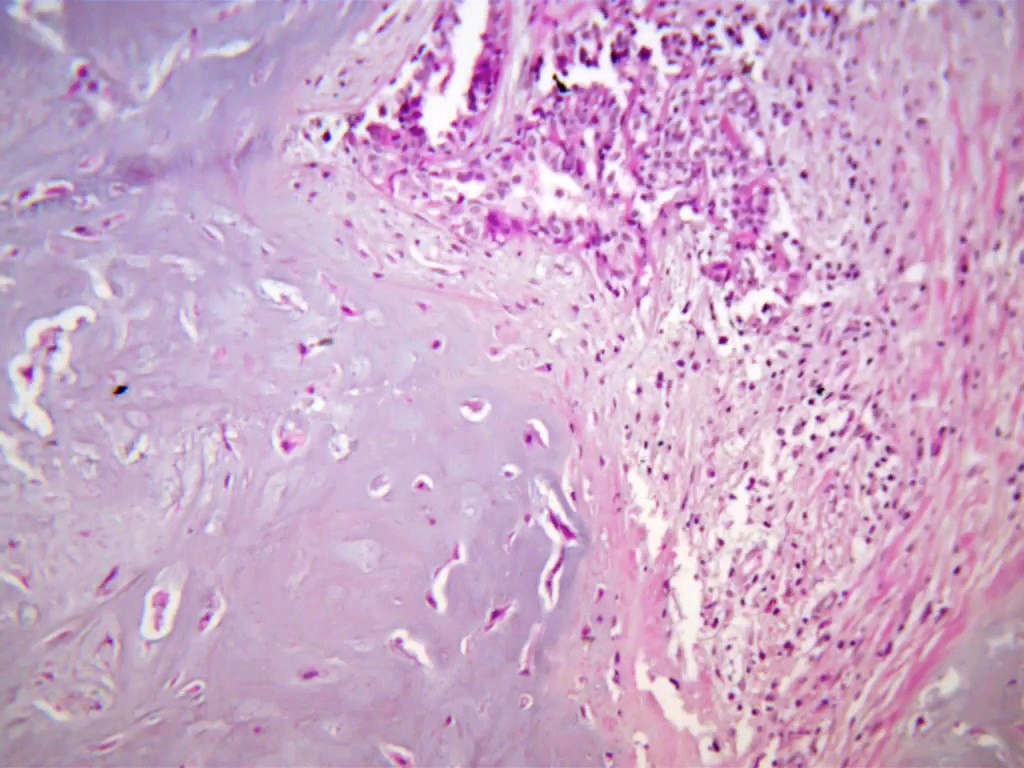

Supplement: Supplementary file 1 [file animals-13-01563-s001.zip › supplementary files/File S1 Canine Mammary Tumor Dataset/benign/Benign mixed tumor_465_11V2_FRM_011 (10).jpg]

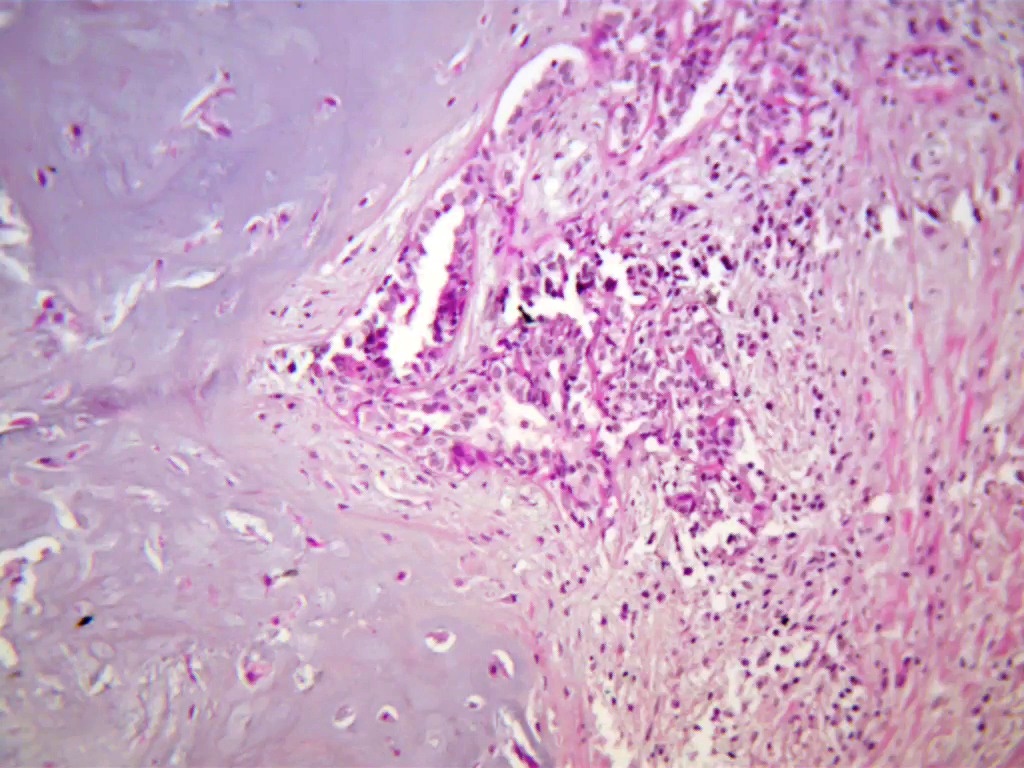

Supplement: Supplementary file 1 [file animals-13-01563-s001.zip › supplementary files/File S1 Canine Mammary Tumor Dataset/benign/Benign mixed tumor_465_11V2_FRM_011 (11).jpg]

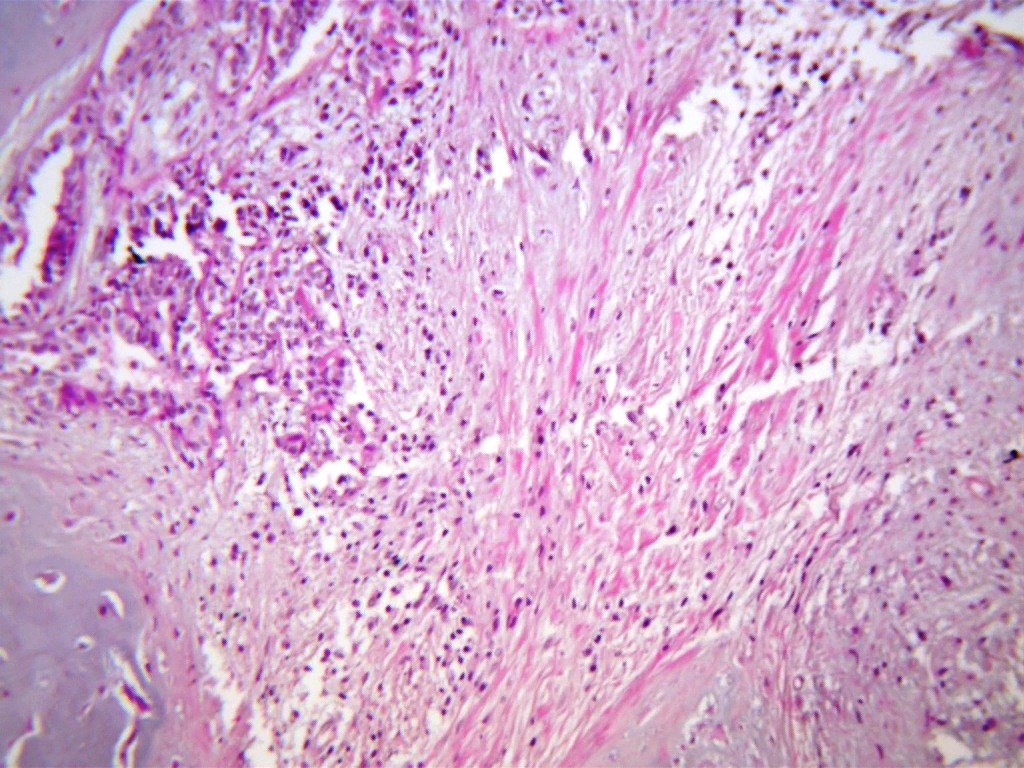

Supplement: Supplementary file 1 [file animals-13-01563-s001.zip › supplementary files/File S1 Canine Mammary Tumor Dataset/benign/Benign mixed tumor_465_11V2_FRM_011 (12).jpg]

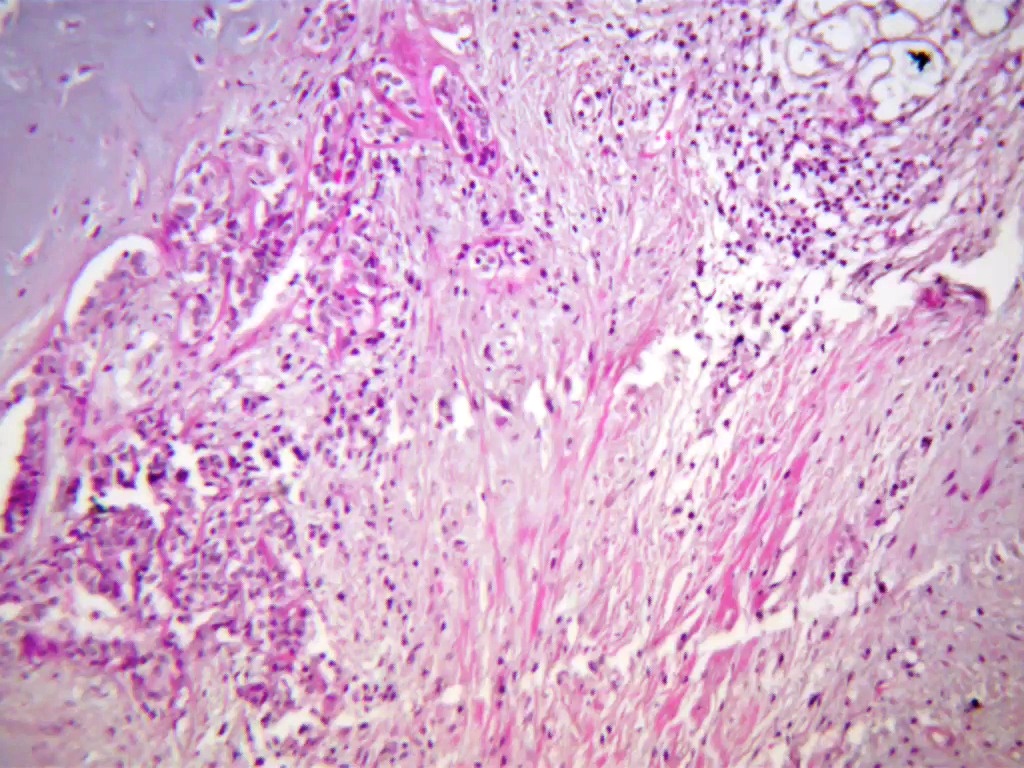

Supplement: Supplementary file 1 [file animals-13-01563-s001.zip › supplementary files/File S1 Canine Mammary Tumor Dataset/benign/Benign mixed tumor_465_11V2_FRM_011 (13).jpg]

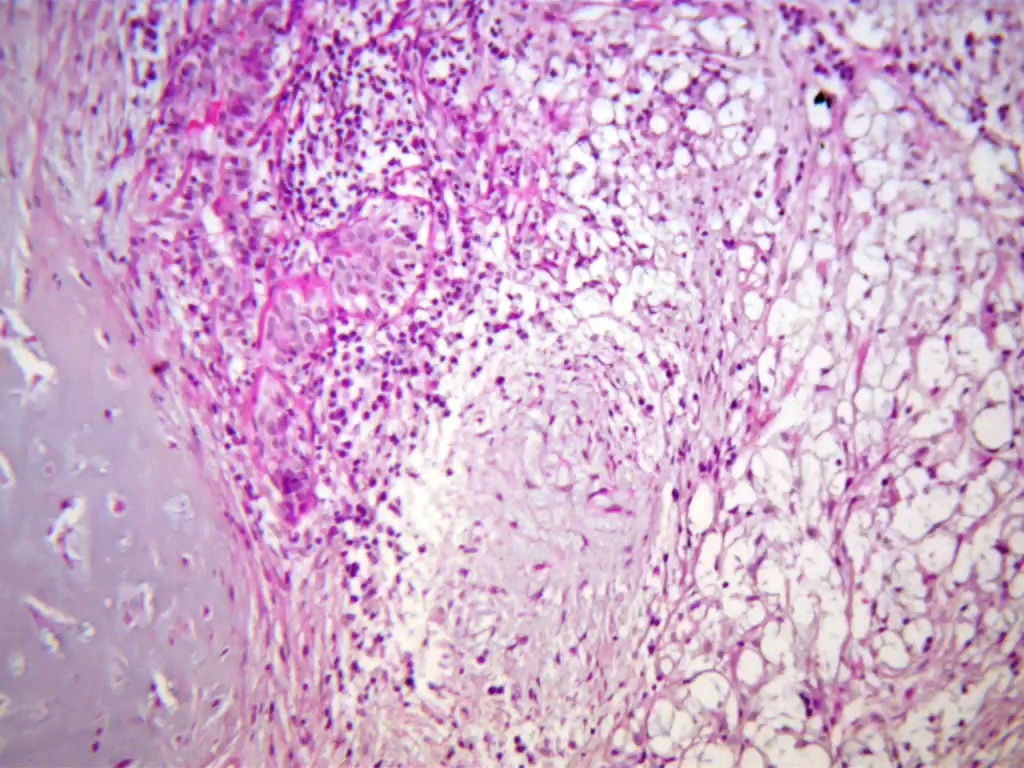

Supplement: Supplementary file 1 [file animals-13-01563-s001.zip › supplementary files/File S1 Canine Mammary Tumor Dataset/benign/Benign mixed tumor_465_11V2_FRM_011 (14).jpg]

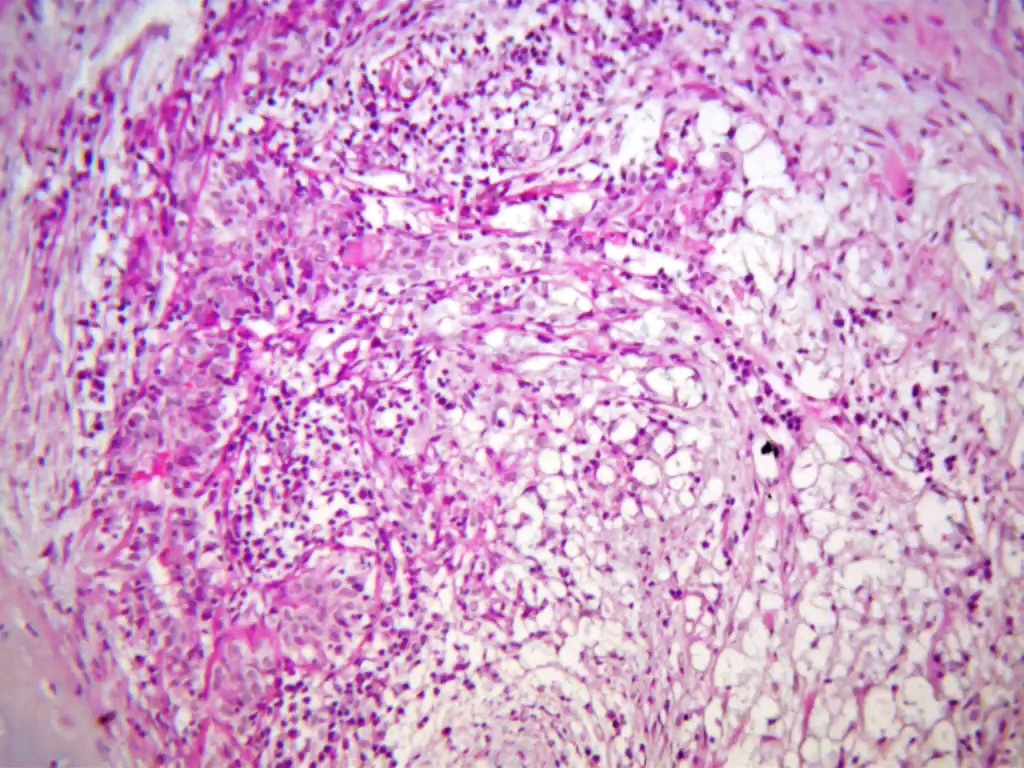

Supplement: Supplementary file 1 [file animals-13-01563-s001.zip › supplementary files/File S1 Canine Mammary Tumor Dataset/benign/Benign mixed tumor_465_11V2_FRM_011 (15).jpg]

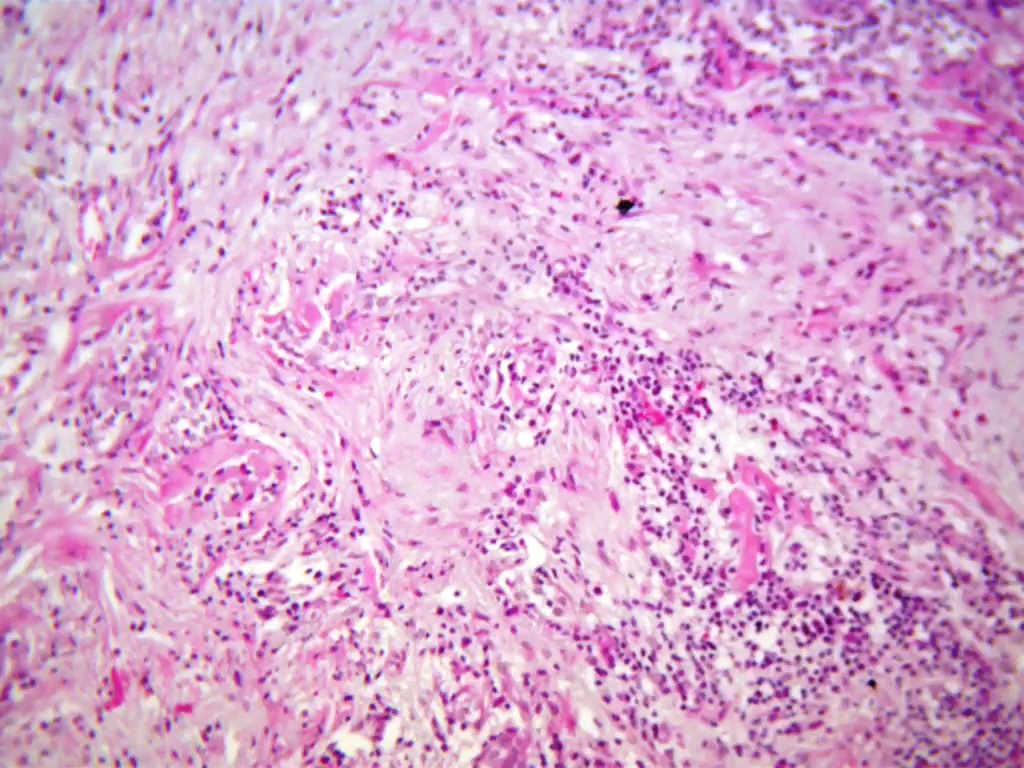

Supplement: Supplementary file 1 [file animals-13-01563-s001.zip › supplementary files/File S1 Canine Mammary Tumor Dataset/benign/Benign mixed tumor_465_11V2_FRM_011 (16).jpg]

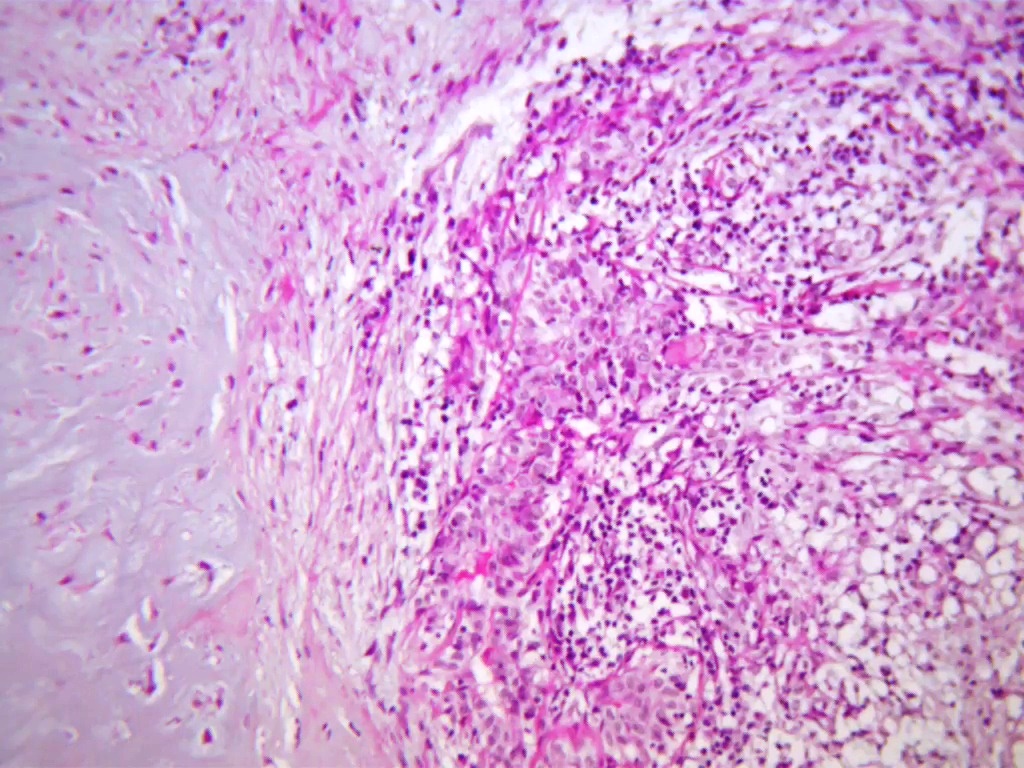

Supplement: Supplementary file 1 [file animals-13-01563-s001.zip › supplementary files/File S1 Canine Mammary Tumor Dataset/benign/Benign mixed tumor_465_11V2_FRM_011 (17).jpg]

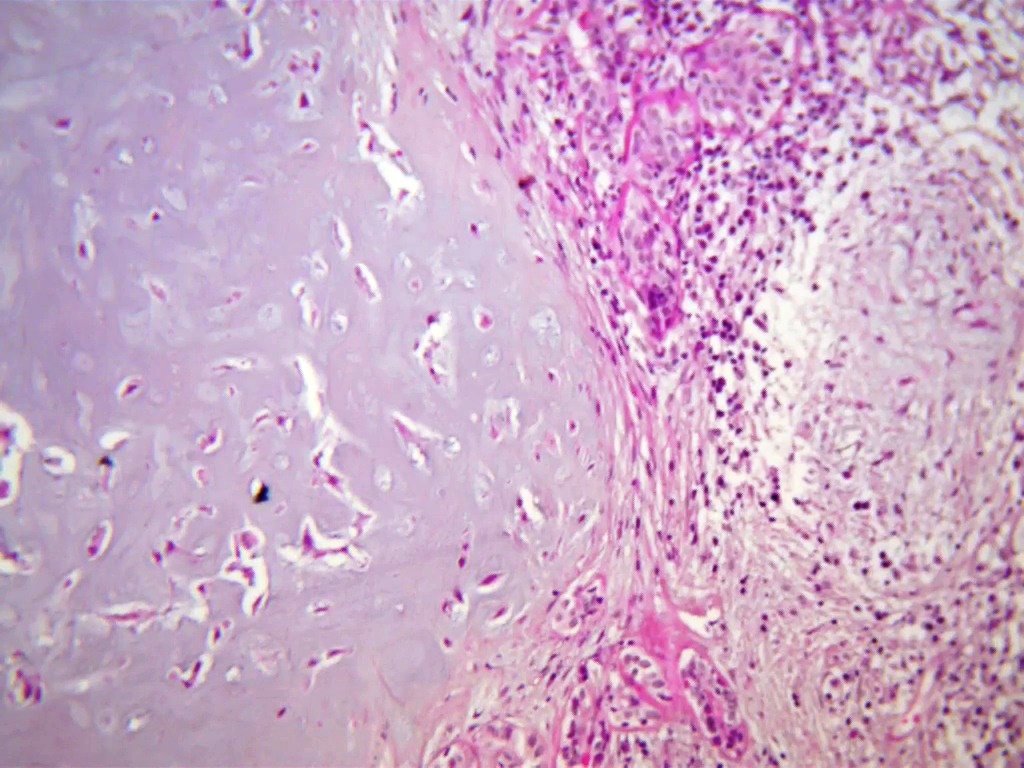

Supplement: Supplementary file 1 [file animals-13-01563-s001.zip › supplementary files/File S1 Canine Mammary Tumor Dataset/benign/Benign mixed tumor_465_11V2_FRM_011 (18).jpg]

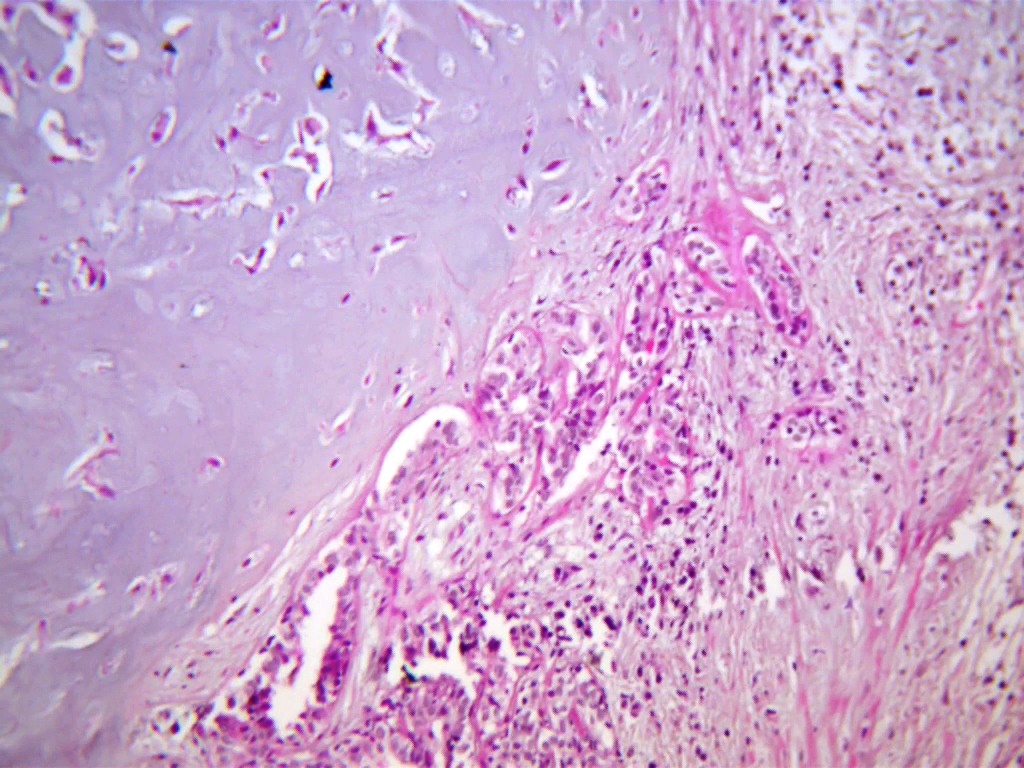

Supplement: Supplementary file 1 [file animals-13-01563-s001.zip › supplementary files/File S1 Canine Mammary Tumor Dataset/benign/Benign mixed tumor_465_11V2_FRM_011 (19).jpg]

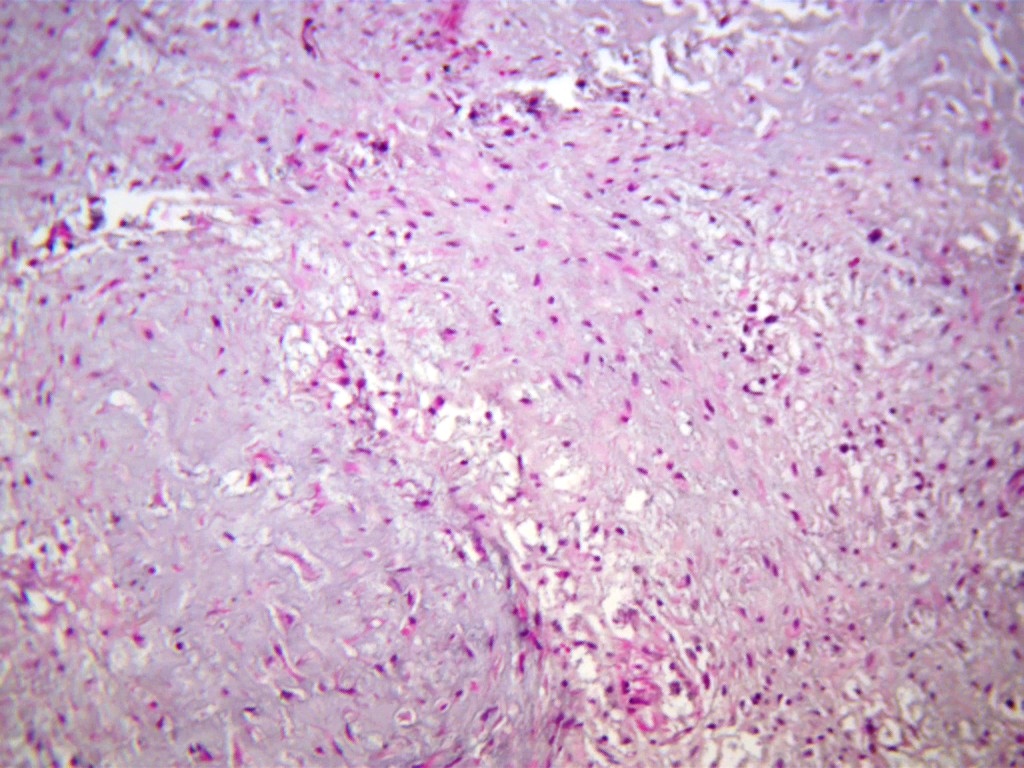

Supplement: Supplementary file 1 [file animals-13-01563-s001.zip › supplementary files/File S1 Canine Mammary Tumor Dataset/benign/Benign mixed tumor_465_11V2_FRM_011 (2).jpg]

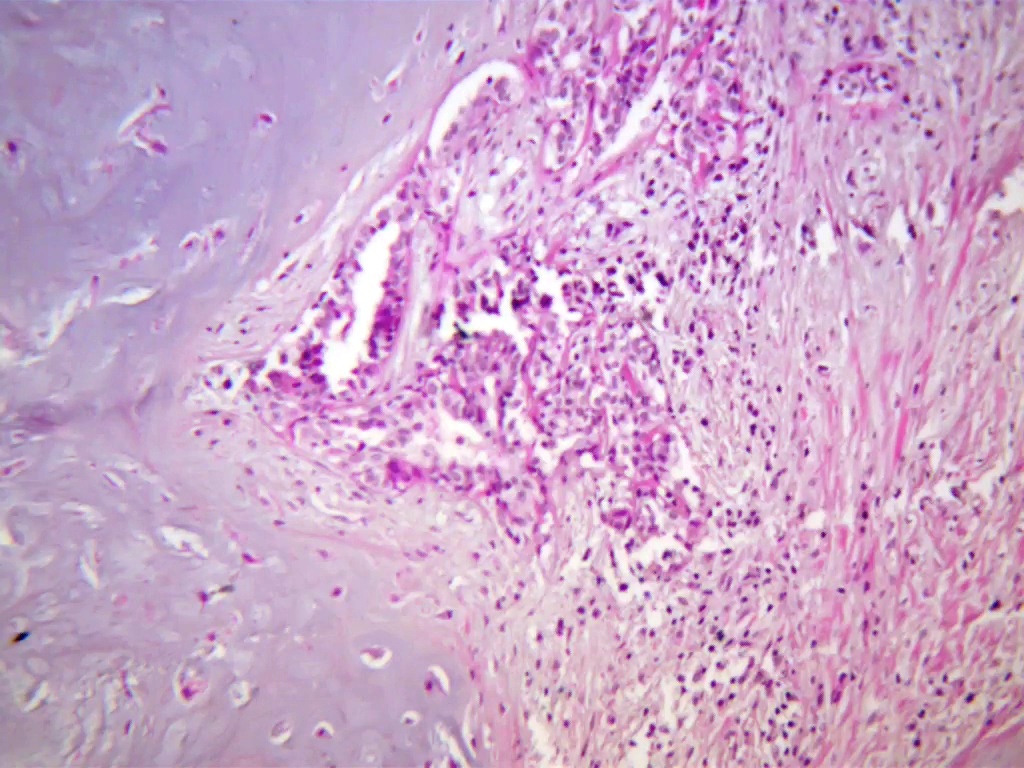

Supplement: Supplementary file 1 [file animals-13-01563-s001.zip › supplementary files/File S1 Canine Mammary Tumor Dataset/benign/Benign mixed tumor_465_11V2_FRM_011 (20).jpg]

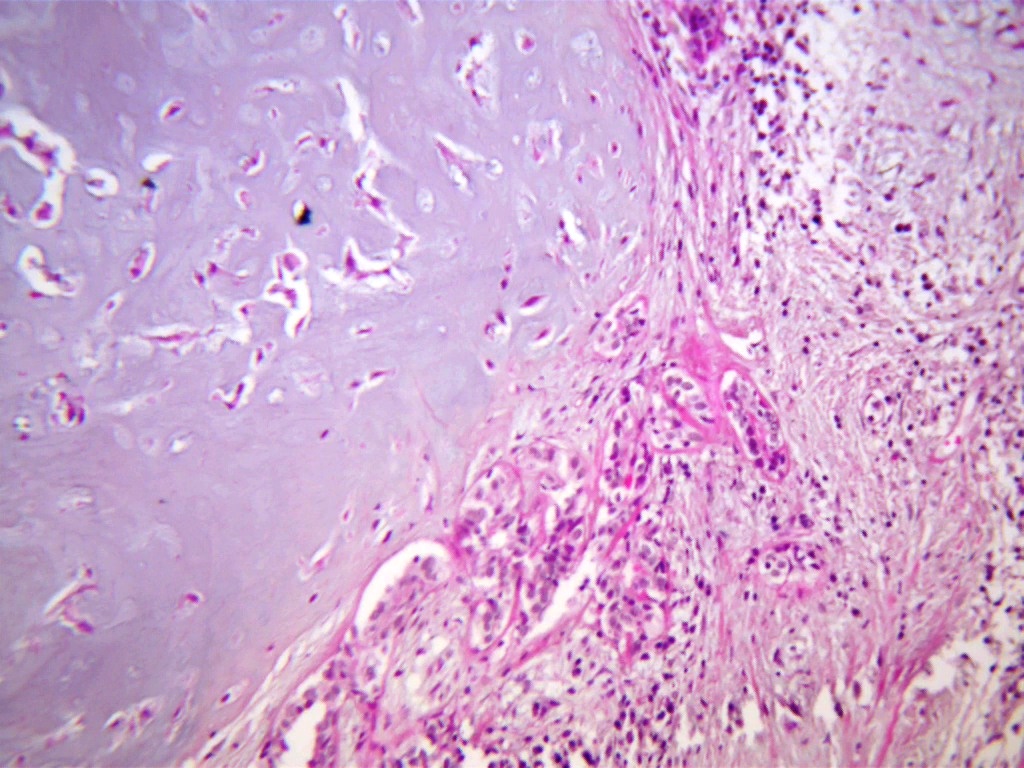

Supplement: Supplementary file 1 [file animals-13-01563-s001.zip › supplementary files/File S1 Canine Mammary Tumor Dataset/benign/Benign mixed tumor_465_11V2_FRM_011 (21).jpg]

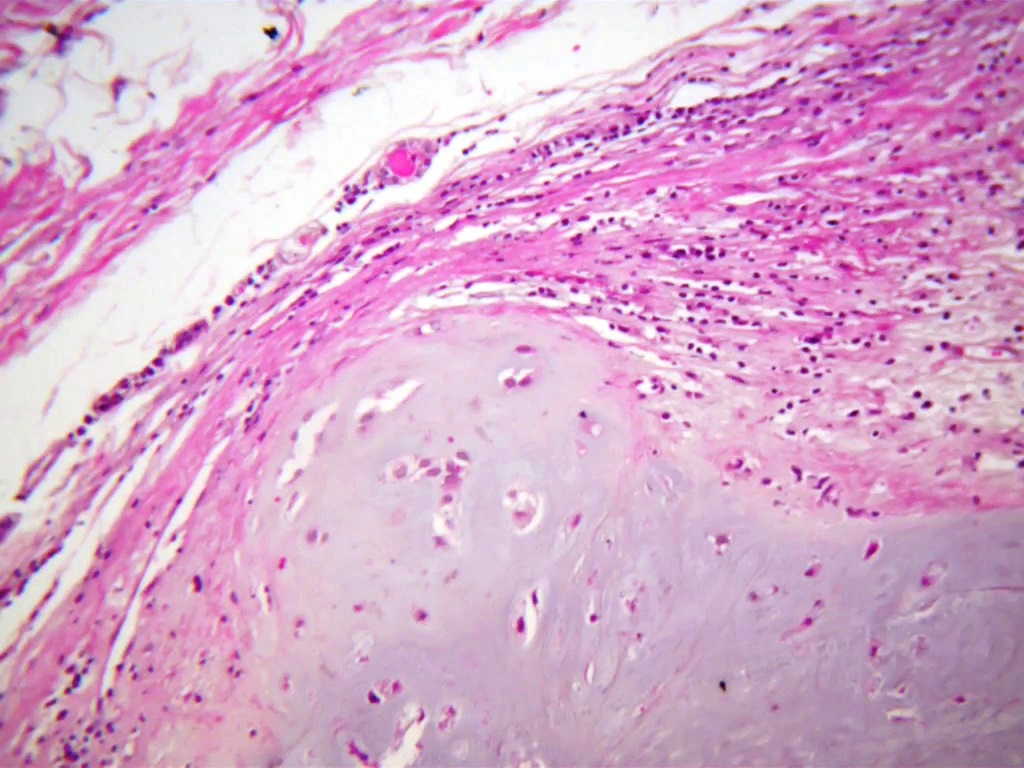

Supplement: Supplementary file 1 [file animals-13-01563-s001.zip › supplementary files/File S1 Canine Mammary Tumor Dataset/benign/Benign mixed tumor_465_11V2_FRM_011 (22).jpg]

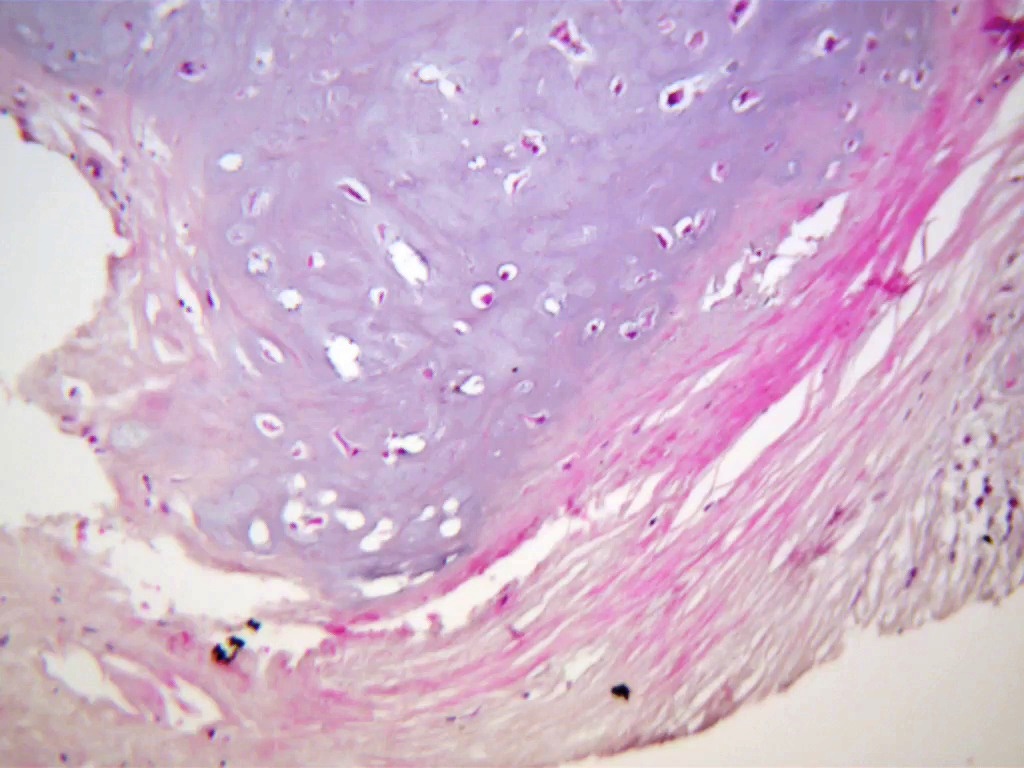

Supplement: Supplementary file 1 [file animals-13-01563-s001.zip › supplementary files/File S1 Canine Mammary Tumor Dataset/benign/Benign mixed tumor_465_11V2_FRM_011 (23).jpg]

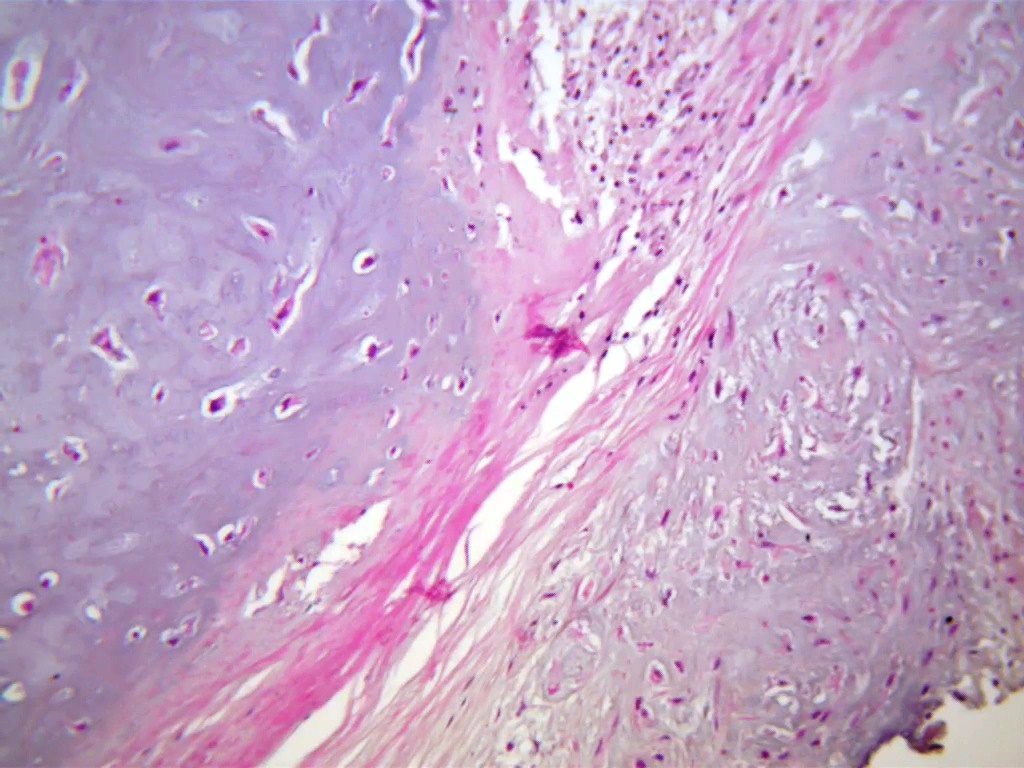

Supplement: Supplementary file 1 [file animals-13-01563-s001.zip › supplementary files/File S1 Canine Mammary Tumor Dataset/benign/Benign mixed tumor_465_11V2_FRM_011 (24).jpg]

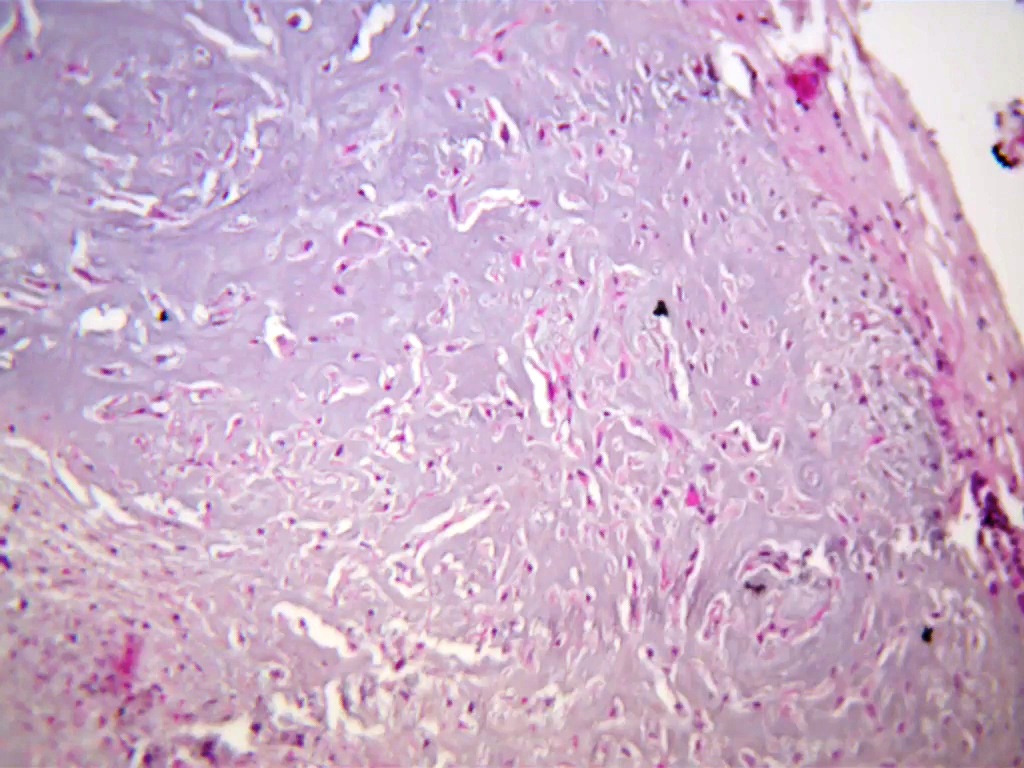

Supplement: Supplementary file 1 [file animals-13-01563-s001.zip › supplementary files/File S1 Canine Mammary Tumor Dataset/benign/Benign mixed tumor_465_11V2_FRM_011 (3).jpg]

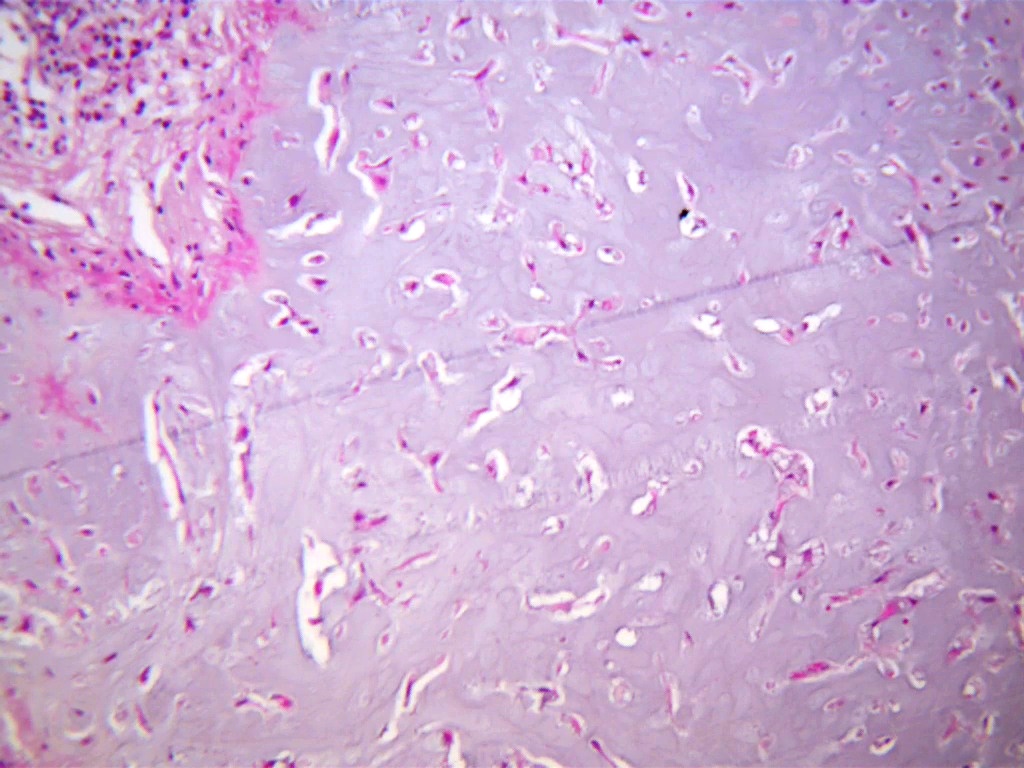

Supplement: Supplementary file 1 [file animals-13-01563-s001.zip › supplementary files/File S1 Canine Mammary Tumor Dataset/benign/Benign mixed tumor_465_11V2_FRM_011 (4).jpg]

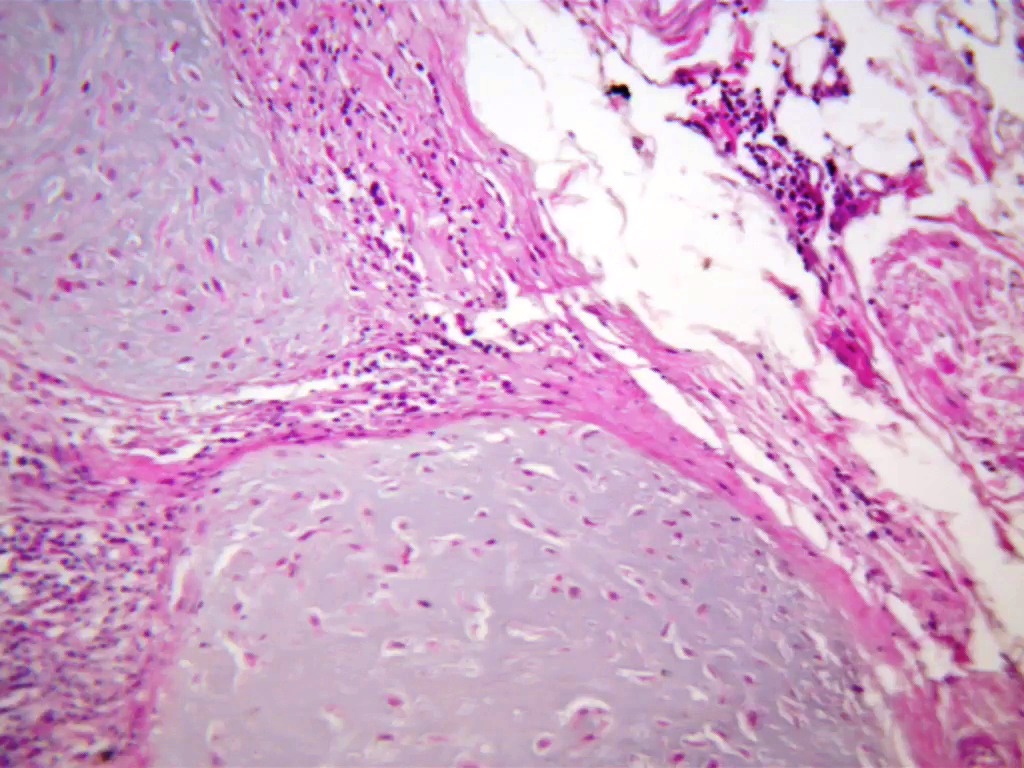

Supplement: Supplementary file 1 [file animals-13-01563-s001.zip › supplementary files/File S1 Canine Mammary Tumor Dataset/benign/Benign mixed tumor_465_11V2_FRM_011 (5).jpg]

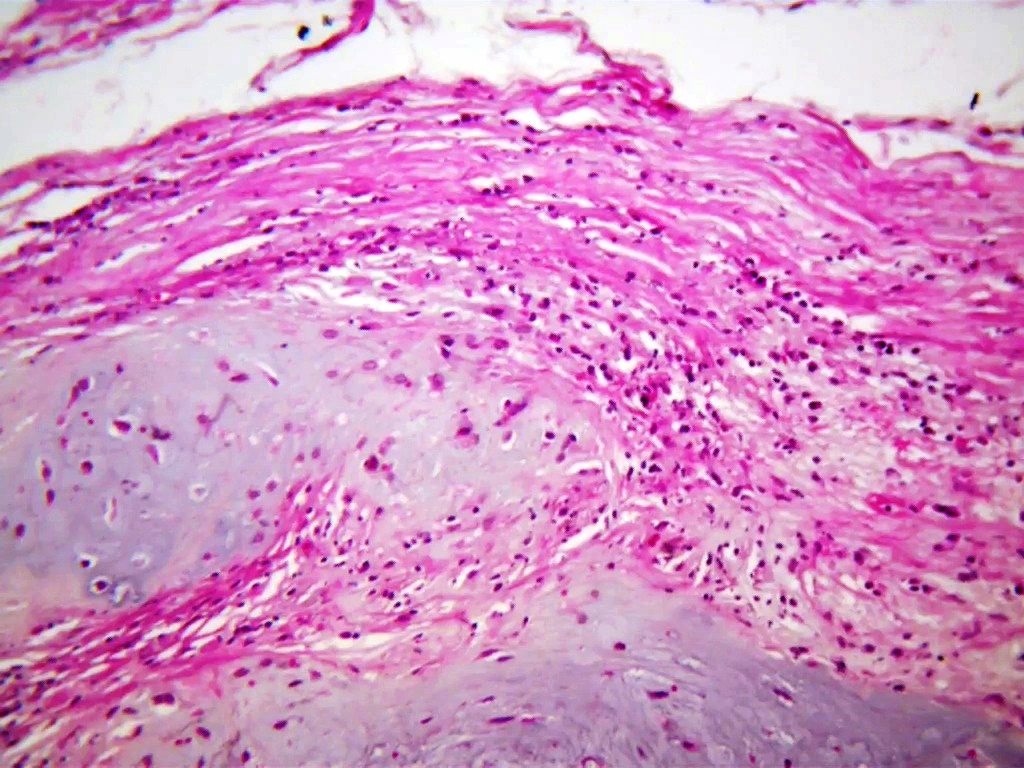

Supplement: Supplementary file 1 [file animals-13-01563-s001.zip › supplementary files/File S1 Canine Mammary Tumor Dataset/benign/Benign mixed tumor_465_11V2_FRM_011 (6).jpg]

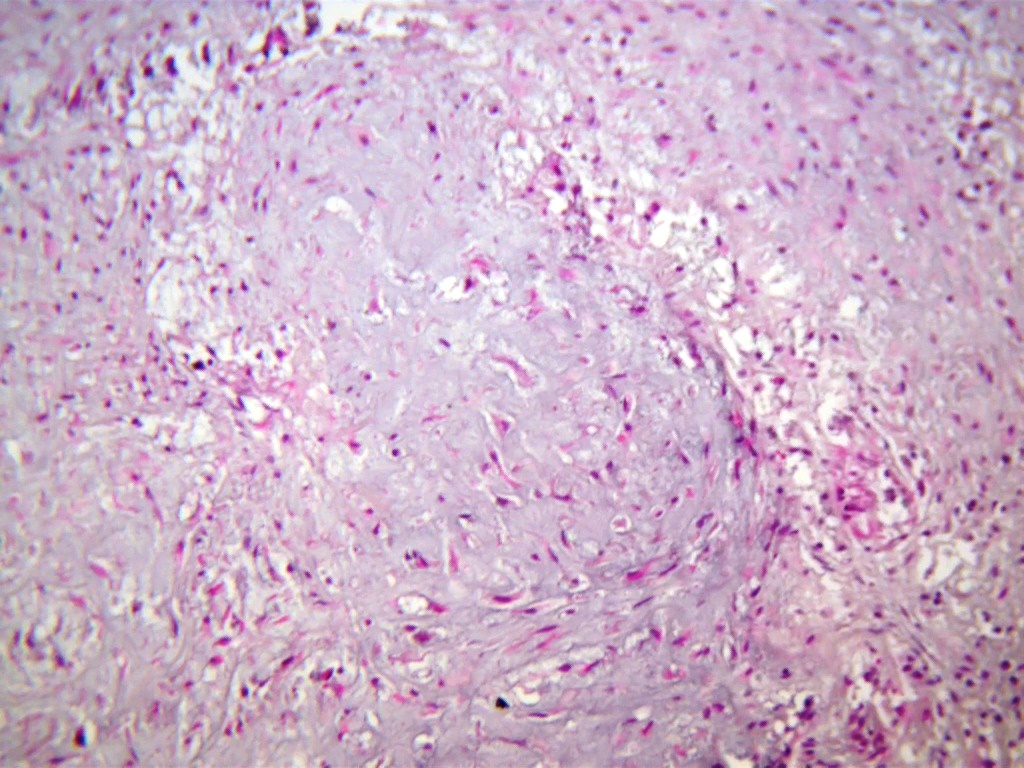

Supplement: Supplementary file 1 [file animals-13-01563-s001.zip › supplementary files/File S1 Canine Mammary Tumor Dataset/benign/Benign mixed tumor_465_11V2_FRM_011 (7).jpg]

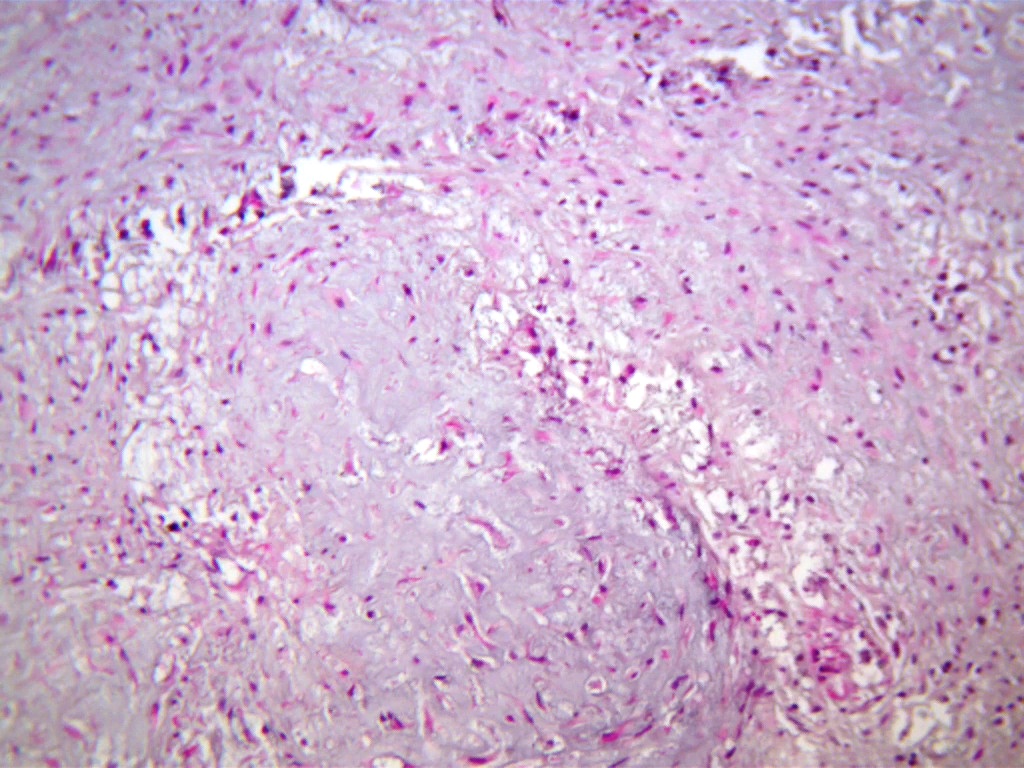

Supplement: Supplementary file 1 [file animals-13-01563-s001.zip › supplementary files/File S1 Canine Mammary Tumor Dataset/benign/Benign mixed tumor_465_11V2_FRM_011 (8).jpg]

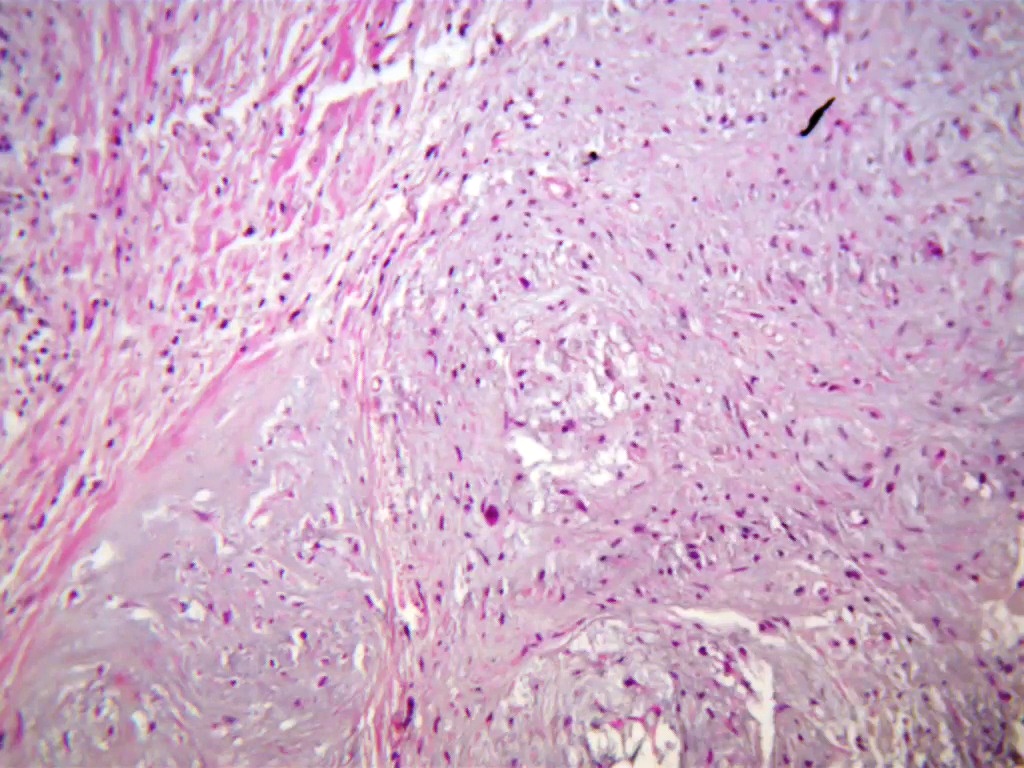

Supplement: Supplementary file 1 [file animals-13-01563-s001.zip › supplementary files/File S1 Canine Mammary Tumor Dataset/benign/Benign mixed tumor_465_11V2_FRM_011 (9).jpg]

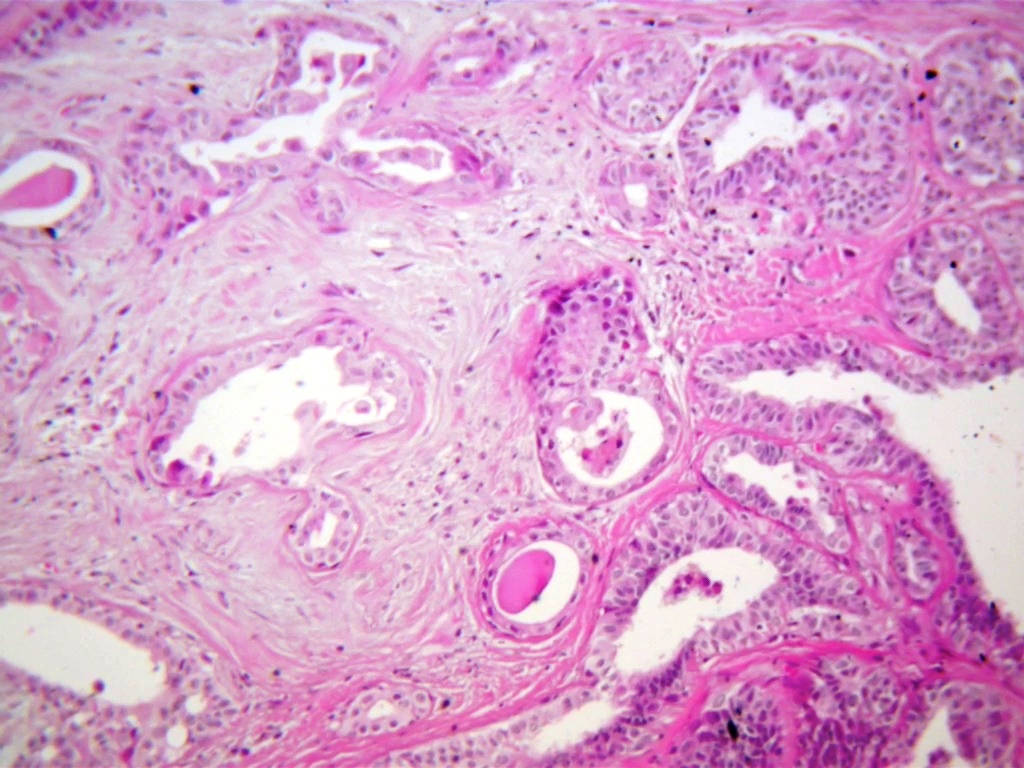

Supplement: Supplementary file 1 [file animals-13-01563-s001.zip › supplementary files/File S1 Canine Mammary Tumor Dataset/benign/Benign mixed tumor_99_369V2_FRM_000 (1).jpg]

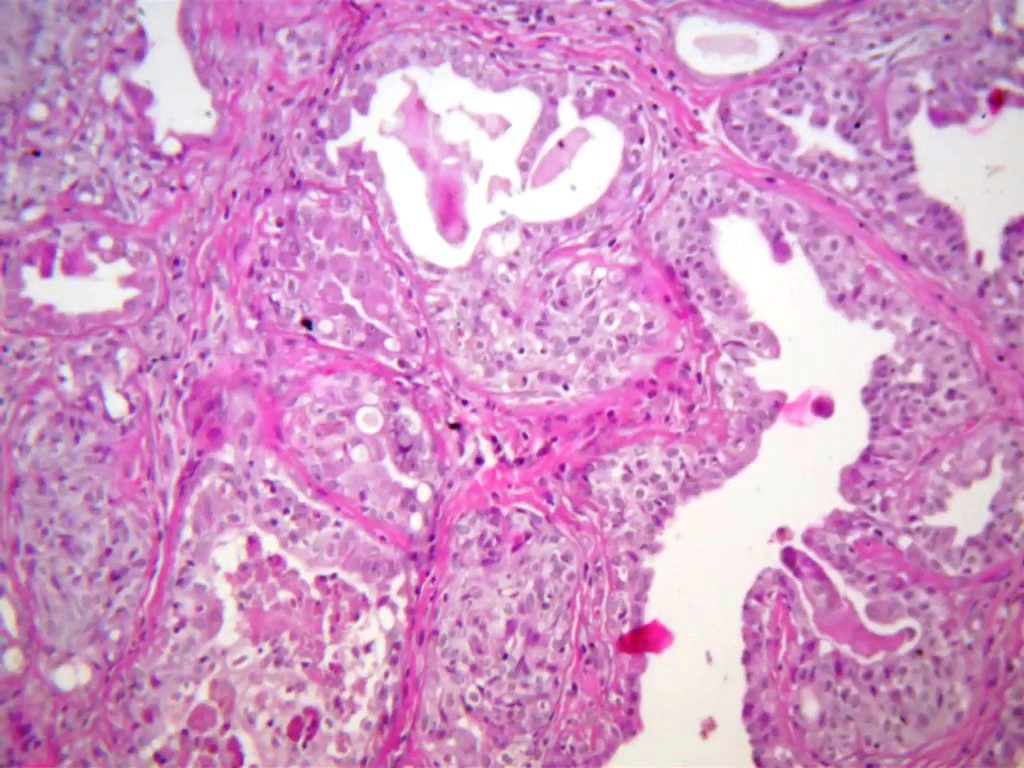

Supplement: Supplementary file 1 [file animals-13-01563-s001.zip › supplementary files/File S1 Canine Mammary Tumor Dataset/benign/Benign mixed tumor_99_369V2_FRM_000 (10).jpg]
